# Comparative Genomics of Emerging Human Ehrlichiosis Agents 

**DOI:** 10.1371/journal.pgen.0020021

**Published:** 2006-02-17

**Authors:** Julie C. Dunning Hotopp, Mingqun Lin, Ramana Madupu, Jonathan Crabtree, Samuel V Angiuoli, Jonathan Eisen, Rekha Seshadri, Qinghu Ren, Martin Wu, Teresa R Utterback, Shannon Smith, Matthew Lewis, Hoda Khouri, Chunbin Zhang, Hua Niu, Quan Lin, Norio Ohashi, Ning Zhi, William Nelson, Lauren M Brinkac, Robert J Dodson, M. J Rosovitz, Jaideep Sundaram, Sean C Daugherty, Tanja Davidsen, Anthony S Durkin, Michelle Gwinn, Daniel H Haft, Jeremy D Selengut, Steven A Sullivan, Nikhat Zafar, Liwei Zhou, Faiza Benahmed, Heather Forberger, Rebecca Halpin, Stephanie Mulligan, Jeffrey Robinson, Owen White, Yasuko Rikihisa, Hervé Tettelin

**Affiliations:** 1 The Institute for Genomic Research, Rockville, Maryland, United States of America; 2 Department of Veterinary Biosciences, College of Veterinary Medicine, The Ohio State University, Columbus, Ohio, United States of America; 3 J. Craig Venter Joint Technology Center, Rockville, Maryland, United States of America; The US DoE Joint Genome Institute, United States of America

## Abstract

*Anaplasma* (formerly *Ehrlichia*) *phagocytophilum, Ehrlichia chaffeensis,* and *Neorickettsia* (formerly *Ehrlichia*) *sennetsu* are intracellular vector-borne pathogens that cause human ehrlichiosis, an emerging infectious disease. We present the complete genome sequences of these organisms along with comparisons to other organisms in the Rickettsiales order. *Ehrlichia* spp. and *Anaplasma* spp. display a unique large expansion of immunodominant outer membrane proteins facilitating antigenic variation. All Rickettsiales have a diminished ability to synthesize amino acids compared to their closest free-living relatives. Unlike members of the Rickettsiaceae family, these pathogenic Anaplasmataceae are capable of making all major vitamins, cofactors, and nucleotides, which could confer a beneficial role in the invertebrate vector or the vertebrate host. Further analysis identified proteins potentially involved in vacuole confinement of the Anaplasmataceae, a life cycle involving a hematophagous vector, vertebrate pathogenesis, human pathogenesis, and lack of transovarial transmission. These discoveries provide significant insights into the biology of these obligate intracellular pathogens.

## Synopsis

Ehrlichiosis is an acute disease that triggers flu-like symptoms in both humans and animals. It is caused by a range of bacteria transmitted by ticks or flukes. Because these bacteria are difficult to culture, however, the organisms are poorly understood. The genomes of three emerging human pathogens causing ehrlichiosis were sequenced. A database was designed to allow the comparison of these three genomes to sixteen other bacteria with similar lifestyles. Analysis from this database reveals new species-specific and disease-specific genes indicating niche adaptations, pathogenic traits, and other features. In particular, one of the organisms contains more than 100 copies of a single gene involved in interactions with the host(s). These comparisons also enabled a reconstruction of the metabolic potential of five representative genomes from these bacteria and their close relatives. With this work, scientists can study these emerging pathogens in earnest.

## Introduction

*Anaplasma phagocytophilum, Ehrlichia chaffeensis,* and Neorickettsia sennetsu are small (approximately 0.4–1.5 μm), pleomorphic α-Proteobacteria. These bacteria are human pathogens that replicate in membrane-bound compartments inside host granulocytes *(A. phagocytophilum)* or monocytes/macrophages *(E. chaffeensis* and *N. sennetsu)* [1–3]. They are obligate intracellular pathogens with a life cycle that involves both vertebrate and invertebrate hosts. A. phagocytophilum and *E. chaffeensis* depend on hematophagous ticks as vectors and wild mammals as reservoir hosts (Table 1) [2,4]. Unknown trematodes are suspected to be the vector and reservoir of N. sennetsu [1]. No vaccine exists for any of these human pathogens.

**Table 1:**
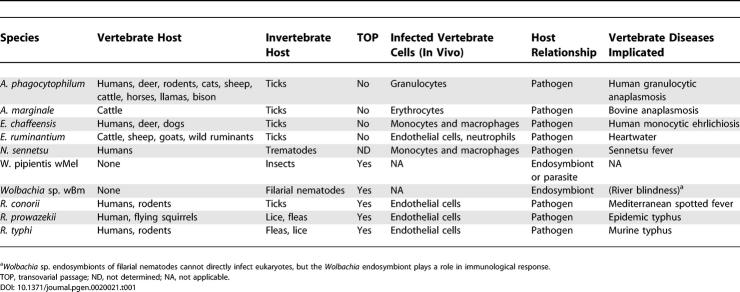
Biological Characteristics of the Rickettsiales

A. phagocytophilum is the causative agent of human granulocytic anaplasmosis (HGA), formerly recognized as human granulocytic ehrlichiosis (HGE) [5,6]. Infection with A. phagocytophilum causes fever, headache, myalgia, anorexia, and chills [7]. Prior to 1994, only ruminant and equine ehrlichiosis were known to be caused by this organism [1]. A. phagocytophilum is transmitted by *Ixodes* spp. Cases of HGA correspond to the distribution of *Ixodes* spp. being identified in New England, the mid-Atlantic region, the upper Midwest, and northern California in the United States, as well as in parts of Europe. A. phagocytophilum is one of the leading causes of ehrlichiosis in the world. Recent serological data suggest that as much as 15%–36% of the population in endemic areas has been infected [8]. Far fewer individuals are diagnosed with a symptomatic infection that varies in severity from fever to death [8]. Half of all symptomatic patients require hospitalization, and 5%–7% require intensive care [8].

Human monocytic ehrlichiosis (HME), caused by *E. chaffeensis,* was discovered in 1986 [9–11]. HME is a systemic disease indistinguishable from HGA [12]. E. chaffeensis has been most commonly identified in the Lone Star tick *(Amblyomma americanum),* with white-tailed deer considered to be the major reservoir. Over 500 cases of HME were diagnosed from 1986 to 1997, predominantly in the south-central and southeastern United States [12]. The recognition and increased prevalence of the disease has been proposed to be related to changes in the host-vector ecology [12]. As with all emerging diseases, it is likely outbreaks occurred in the preceding decades. Notably, 1,000 troops training in Texas contracted an unexplained disease with similar symptoms after exposure to the vector from 1942 to 1943 [12].

N. sennetsu is a monocytotropic species that causes sennetsu ehrlichiosis, an infectious mononucleosis-like disease with fever, fatigue, general malaise, and lymphadenopathy [1,13]. Less is known about the distribution of N. sennetsu when compared to *Anaplasma* and *Ehrlichia*. However, sequencing of its genome allows for interesting comparisons, since tissue tropism and clinical symptoms are similar but the vector (unknown trematodes) is different. Additionally, in the United States and Canada, domestic animals infected with the closely related N. risticii develop Potomac horse fever, an acute febrile disease accompanied by diarrhea with high morbidity and mortality [14,15]. The related N. helminthoeca causes acute and highly fatal salmon-poisoning disease of domestic and wild canines [14,16].

**Figure 1:**
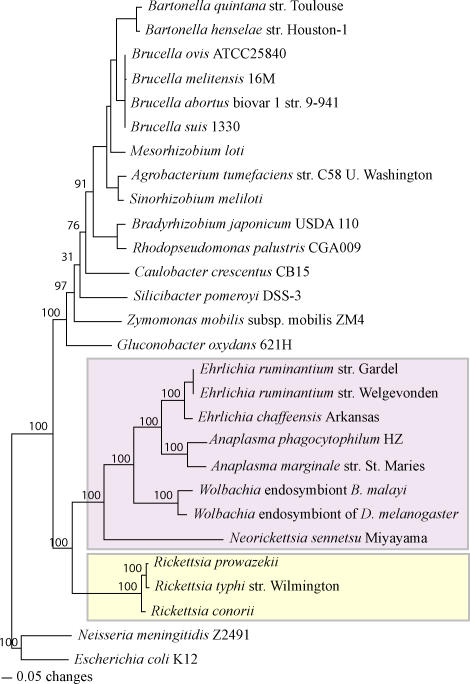
Phylogenetic Tree of the α-Proteobacteria The protein sequences of select conserved genes were concatenated and aligned, and a phylogenetic tree was inferred of all sequenced α-Proteobacteria (see Materials and Methods). The Anaplasmataceae (purple) and the Rickettsiaceae (yellow) are highlighted.

Along with *Wolbachia,* these bacteria are members of the Anaplasmataceae family (Figure 1) [3]. *Wolbachia* infect arthropods and filarial nematodes, but have not been shown to infect vertebrates directly.

Together with the Rickettsiaceae, the Anaplasmataceae are members of the order Rickettsiales (Figure 1) [3]. The Rickettsiaceae include the obligate intracellular *Rickettsia* spp. Like the Anaplasmataceae, the Rickettsiaceae are obligate intracellular pathogens with a life cycle that involves both vertebrate and invertebrate hosts, but they replicate directly in the cytosol of endothelial cells. All organisms in the order Rickettsiales have relatively small genomes (0.8–1.5 Mb) that have arisen through reductive evolution as they developed dependence on the host cell for necessary functions [17]. The Rickettsiales and other α-Proteobacteria also have an unresolved evolutionary relationship with the progenitor of the mitochondria [18,19].

Three Rickettsiaceae genomes have been published: *Rickettsia prowazekii, R. conorii,* and R. typhi [18,20,21]. Four Anaplasmataceae genomes have been published: the insect parasite *W. pipientis w*Mel, the filarial nematode endosymbiont *Wolbachia* sp. *w*Bm, the bovine pathogen *Anaplasma marginale,* and the bovine pathogen Ehrlichia ruminantium [19,22–24].

We present here a comparison of the previously completed Rickettsiales genomes to the first complete genomes of three representative Anaplasmataceae human pathogens: *A. phagocytophilum, E. chaffeensis,* and N. sennetsu. The complete genome sequence of these human pathogens will enhance the opportunities for investigation of virulence factors, pathogenesis, immune modulation, and novel targets for antimicrobial therapy and vaccines.

## Results/Discussion

### Genome Anatomy

*A. phagocytophilum, E. chaffeensis,* and N. sennetsu each have a single circular chromosome (Figure S1). Most genomic features are typical of the sequenced Rickettsiales (Table 2). *W. pipientis w*Mel, *Ehrlichia* spp., and *Anaplasma* spp., which are most closely related, all have numerous repeats in their genomes. In contrast, N. sennetsu and R. prowazekii have only six repeats in their respective genomes (Table 2). The repetitive nature of the *Ehrlichia* and *Anaplasma* genomes is exemplified by the expansion of outer membrane proteins of the OMP-1/P44/Msp2 family (discussed below). In addition numerous other functionally important genes are duplicated including those involved in type IV secretion and vitamin/cofactor biosynthesis.

**Table 2:**
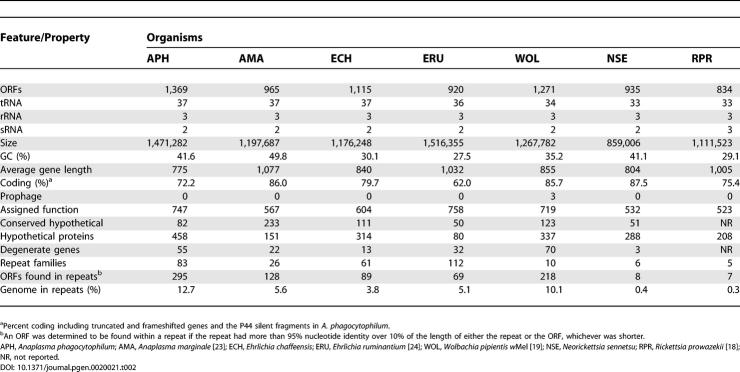
Genome Properties

**Figure 2:**
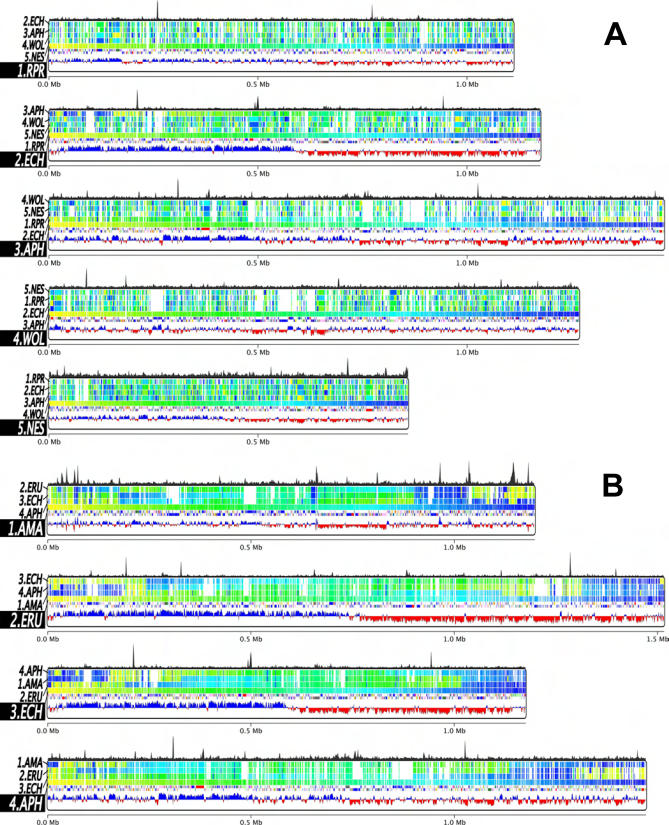
Synteny of the Rickettsiales Regions of conserved synteny were identified using the ortholog clusters (see Materials and Methods) and visualized with Sybil. The genes along each ordered chromosome were colored on a gradient from yellow to blue. The ortholog clusters for each query genome were then plotted relative to the order of the reference genome. Regions of synteny are then seen as continuous gradients across large regions of the genome. Above the synteny gradient display is the atypical nucleotide composition. Below the gradient display are the predicted coding regions on the plus strand and the minus strand, and the GC-skew. Representatives of all the Rickettsiales (A) and representative *Ehrlichia* spp. and *Anaplasma* spp. (B) were compared separately. AMA, A. marginale St. Maries; APH, A. phagocytophilum HZ; ECH, E. chaffeensis Arkansas; ERU, E. ruminantium Welgevonden; NES, N. sennetsu Miyayama; RPR, R. prowazekii Madrid E; WOL, *W. pipientis w*Mel.

The origin of replication was not experimentally determined in any of the genomes. As with other Rickettsiales [18], genes typically clustered near the origin *(dnaA, gyrA, gyrB, rpmH, dnaN, parA,* and *parB)* were dispersed throughout the genomes. For E. chaffeensis and *N. sennetsu,* a clear shift in GC-skew occurs near *parA* and *parB* (Figure 2). Therefore, basepair 1 was set in the intergenic region between the two genes. In *A. phagocytophilum,* none of these genes were found near the GC-skew transition. Therefore, basepair 1 was set in the intergenic region near *polA*. For E. chaffeensis and *A. phagocytophilum,* these predictions coincide with the predictions for E. ruminantium [24] and A. marginale [23].

Only three islands of synteny over 10 kb in length are conserved among all the sequenced Anaplasmataceae, and these islands are shared among all the Rickettsiales (Figure 2). They include two operons of ribosomal proteins and one operon of proteins encoding portions of the type IV secretion system. Similar to the other Rickettsiales sequenced, all three genomes have the equivalent of a single rRNA operon with the 16S rRNA separated from the 23S-5S gene pair, as previously described for this order of bacteria [18].

Of genes typically clustered near the origin, *parA* and *parB* were not identified in A. phagocytophilum. Likewise, *parA* and *parB* are truncated in the *Wolbachia* sp. *w*Bm. In various mutational studies in free-living prokaryotes, the effects of inactivation of *parA* or *parB* range from lethality to production of anucleated cells at low copy number [25,26]. Without *parA* and *parB, A. phagocytophilum* and the *Wolbachia* sp. *w*Bm may have random chromosome partitioning, may require an alternate partitioning factor, or may have inefficient chromosome partitioning.

**Figure 3:**
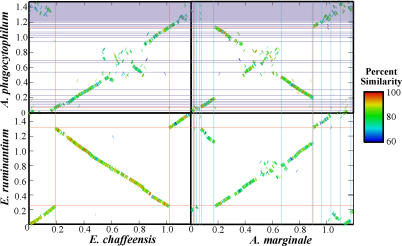
Synteny between *Anaplasma* spp. and *Ehrlichia* spp. *Anaplasma* spp. and *Ehrlichia* spp. share conserved gene order (synteny) across their chromosomes. E. ruminantium and E. chaffeensis have a single symmetrical inversion near two duplicate Rho termination factors (approximate positions shown in pink). Genomic rearrangements between these Rho termination factors are also apparent in A. marginale (pink). In addition to the synteny breaks near the Rho termination factors, A. marginale has rearrangements located near the *msp2-* and *msp3-* expression locus and pseudogenes (approximate positions shown in light blue). Likewise, in *A. phagocytophilum,* numerous changes in genome arrangement are located near the homologous *p44* expression locus and silent genes (approximate positions shown in lavender).

Of all the sequenced Anaplasmataceae, only the *Anaplasma* spp. and *Ehrlichia* spp. share conserved gene order (synteny) across their chromosome (Figure 2). E. ruminantium and E. chaffeensis have a single symmetrical inversion near two duplicate Rho termination factors (Figure 3). Symmetrical inversions around the origin are the most common large-scale rearrangements in microbial genomes [27]. Genomic rearrangements between these Rho termination factors are also apparent in A. marginale. The presence of the same break in both the *Anaplasma* and *Ehrlichia* lineages suggests that the duplicate Rho termination factors allow for repeated inversions across this region of the genome.

In addition to the synteny breaks near the Rho termination factors, A. marginale has rearrangements located near the *msp2* and *msp3* expression loci and their corresponding pseudogenes (Figure 3). Likewise, numerous boundaries of genome rearrangements are located near the homologous *p44* expression locus (*p44ES*/APH_1221) and silent genes. In both *Anaplasma* spp., the silent *p44* and *msp2* genes stored in reserve in the genome can recombine into the corresponding expression locus to generate antigenic variation in the immunodominant surface protein (discussed in detail below). These exact, repeated sequences throughout the genome facilitate recombination for antigenic variation and may also provide sites where chromosomal inversions occur.

### Genome Comparisons

In order to compare the genomic content of the Rickettsiales to that of other intracellular bacteria, ortholog clusters were delineated for 19 representatives of obligate and facultative intracellular pathogens and endosymbionts (see Materials and Methods). Such comparisons show conservation of 176 ortholog clusters across these intracellular bacteria (Table S1), most of which correspond to housekeeping functions.

Eleven ortholog clusters present in all the Rickettsiales distinguish the Rickettsiales from other intracellular bacteria examined (Table S2). These include a type I secretion system ATPase, a pyridine nucleotide-disulfide oxidoreductase family protein, a putative transporter, and type IV secretion system proteins VirB9 and VirB8. Thirteen ortholog clusters composed of 12 conserved hypothetical proteins and a GNAT family acetyltransferase distinguish all the Anaplasmataceae from the Rickettsiales (Table S3).

**Figure 4:**
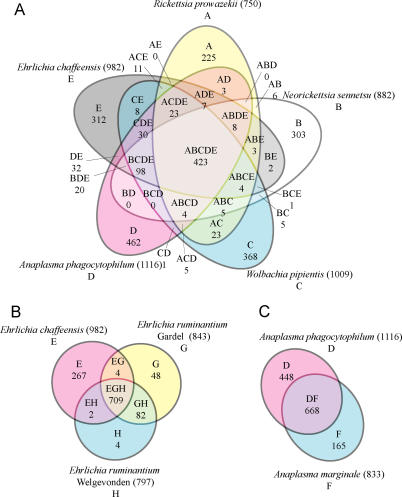
Comparison of the Rickettsiales Gene Sets The composition of ortholog clusters (see Materials and Methods) of representative Rickettsiales (A), *Ehrlichia* spp. (B), and *Anaplasma* spp. (C) were compared. Numbers within the intersections of different ovals indicate ortholog clusters shared by 2, 3, 4, or 5 organisms. Species compared are indicated in diagram intersections as follows. A, *R. prowazekii;* B, *N. sennetsu;* C, *W. pipientis;* D, *A. phagocytophilum;* E, *E. chaffeensis;* F, *A. marginale;* G, E. ruminantium Gardel; and H, E. ruminantium Welgevonden.

Five genera in the Rickettsiales order have at least one representative sequenced. In order to compare these five genera, the following genomes were compared: *R. prowazekii, N. sennetsu, W. pipientis, A. phagocytophilum,* and *E. chaffeensis.* This comparison shows conservation of 423 ortholog clusters (Table S4) generally associated with housekeeping functions. Most genes in the five compared genomes are either conserved among all genomes or unique to a given genome. Indeed, 60% of the two-, three-, and four-way comparisons shared fewer than ten ortholog clusters (Figure 4). In the three-way comparisons, the BDE *(A. phagocytophilum, E. chaffeensis,* and *N. sennetsu)* and CDE *(A. phagocytophilum, E. chaffeensis,* and *W. pipientis)* intersections harbor more than 20 ortholog clusters (Figure 4). The BDE intersection includes the organisms sequenced here and represents the human pathogens with very similar disease outcomes. Ortholog clusters conserved between these organisms include those for vitamin and cofactor biosynthesis enzymes, a monovalent cation/proton antiporter, a dicarboxylate transporter, and a DNA-binding protein (Table S5). Vitamin and cofactor biosynthesis is specific to the human ehrlichiosis agents, suggesting a niche adaptation or pathogenic trait. The CDE intersection is composed of the most closely related organisms. These ortholog clusters include genes for amino acid, fatty acid and nucleotide biosynthesis, an M48 family peptidase, a cytochrome c-type biogenesis protein, and the type IV secretion system protein VirB4 (Table S6).

In two-way comparisons, the AC *(R. prowazekii* and *W. pipientis)* and DE *(A. phagocytophilum* and *E. chaffeensis)* intersections contain more than twenty ortholog clusters. Genes shared only by R. prowazekii and W. pipientis include those for cell wall biosynthesis, subunits of cytochrome D ubiquinol oxidase, a biotin transporter, a dinucleoside polyphosphate hydrolase, and an amino acid permease (Table S7). The presence of genes for cell wall biosynthesis in only R. prowazekii and W. pipientis likely reflects differences in the cell surface; *A. phagocytophilum, E. chaffeensis,* and N. sennetsu do not synthesize peptidoglycan [28]. The peptidoglycan biosynthesis genes are also found in *A. marginale,* which suggests that if these genes are expressed, A. marginale may have a peptidoglycan layer [23]. Since the peptidoglycan genes are present in A. marginale and W. pipientis but not in the other Anaplasmataceae, these genes have either been horizontally acquired in these organisms or have been lost numerous times in the Anaplasmataceae. Peptidoglycan binding to the Toll-like receptor 2 activates leukocytes. Neither A. marginale nor W. pipientis infects the immune cells of a vertebrate host. The peptidoglycan layer may have been lost to allow the organism to successfully infect vertebrate immune cells.

Genes shared only by A. phagocytophilum and E. chaffeensis include those encoding thiamine biosynthetic proteins, a potassium transporter, a peptide deformylase, and an ankyrin repeat protein (Table S8). Thiamine biosynthesis is distinctly absent from *N. sennetsu,* suggesting a possible trematode niche-specific adaptation.

*A. phagocytophilum, E. chaffeensis,* and N. sennetsu have 462, 312, and 303 open reading frames (ORFs) or paralog clusters that are unique with respect to the five-organism ortholog cluster analysis, respectively. The vast majority of these unique genes encode hypothetical, conserved hypothetical, and conserved domain proteins, as well as uncharacterized membrane proteins and lipoproteins. Other A. phagocytophilum-specific genes include those encoding the P44 outer membrane proteins and the HGE-14 and HGE-2 antigenic proteins (Table S9). E. chaffeensis-specific genes include those for the OMP-1 family of proteins, arginine biosynthesis, a major facilitator family transporter, and a variable-length PCR target protein (Table S10). N. sennetsu-specific genes include those for an F-type ATPase beta subunit, a cyclophilin-type peptidyl-prolyl cis-trans isomerase, a branched-chain amino acid transporter, a sensor histidine kinase, a strain-specific surface antigen, thioredoxin, and the type IV secretion system proteins VirB2 and VirB4 (Table S11).

Of the organism-specific genes detected in this five-way comparison, over half were hypothetical proteins, many of which formed genomic islands of hypothetical proteins (Figure 2). The majority of the genes identified as unique were not just unique to the genus, but to the species. Of the 462 A. phagocytophilum-unique genes in this comparison, 448 are also unique when compared with A. marginale. The 21 ortholog clusters shared only between *Anaplasma* spp. include conserved hypothetical proteins, OMP-1 proteins, membrane proteins, and HGE-2 (Table S12). Likewise, of the 312 E. chaffeensis-unique ORFs or paralog clusters in the five-way comparison, 267 are unique upon comparison with either E. ruminantium strain. The 52 ortholog clusters shared only between the *Ehrlichia* spp. include OMP-1 proteins, arginine biosynthetic proteins, a pyrroline-5-carboxylate reductase, a major facilitator protein, conserved hypothetical proteins, membrane proteins, and lipoproteins (Table S13).

Only one ortholog cluster containing conserved hypothetical proteins is shared between the animal pathogens E. ruminantium (Erum1840, ERGA_CDS_01780) and A. marginale (AM279) and are absent from the human pathogens *E. chaffeensis, A. phagocytophilum,* and N. sennetsu. In addition, a homolog of these proteins is present in the Ehrlichia canis Jake publicly available shotgun sequence. Since A. phagocytophilum and E. chaffeensis are maintained in animal reservoirs, presence of this gene is not associated with animal infection. Instead, loss of this protein could be required to establish infection in humans. These conserved hypothetical proteins have some homology to the eukaryotic patatin family of phospholipases. Patatin has been characterized to have phospholipase A-like activity [29].

Except for *N. sennetsu,* all of the sequenced pathogenic Anaplasmataceae require an arthropod-vector that feeds on blood (Table 1). Three ortholog clusters, including one for bacterioferritin and two for conserved hypothetical proteins, are absent in all of the tick-, flea-, and louse-borne Rickettsiales, but are present in *Wolbachia* spp. and N. sennetsu (Table S14). The proteins in these ortholog clusters may be correlated to the lack of a blood-sucking arthropod in the life cycles of these organisms.

The tick-borne Anaplasmataceae (*Ehrlichia* spp. and *Anaplasma* spp.) are the only Rickettsiales that are not transmitted transovarially in the invertebrate host. One ortholog cluster containing a class II aldolase/adducing domain protein (NSE_0849, RC0678, RP493, RT0479, WD0208) is absent only from *Ehrlichia* spp. and *Anaplasma* spp. Lack of this aldolase/adducing domain protein may prevent transovarial transmission in the arthropod vector.

Four ortholog clusters of conserved hypothetical proteins are present in all the pathogenic Rickettsiales but none of the endosymbionts. These proteins, which remain to be characterized, may be essential for pathogenesis or survival in the vertebrate host (Table S15).

### A. phagocytophilum Strain Comparison

As an initial effort to use these genome sequences to identify the conserved genomic content of unsequenced members of these species, we conducted microarray-based comparative genome hybridization analyses with two A. phagocytophilum strains. Except for four *p44* hypervariable regions (discussed below), the genomic content across all three strains is conserved (ratio < 3). Although A. phagocytophilum and A. marginale have very different complements of unique genes, the genomic content within the strains of A. phagocytophilum is highly conserved. Conservation of the gene content of the strains may explain the similarity of clinical signs of HGA from two geographic regions (New York, Minnesota) and equine ehrlichiosis in California [7].

### Free-Living and Obligate-Intracellular α-Proteobacteria

In order to understand the differences between these obligate intracellular pathogens and a closely related free-living organism, the number of genes in each role category was compared between representative Anaplasmataceae and Caulobacter crescentus (Table 3). C. crescentus is a closely related and sequenced free-living α-Proteobacteria to the Rickettsiales [30]. The scope of this comparison was limited to only these five α-Proteobacteria, as only these organisms had role categories assigned in an identical manner.

**Table 3:**
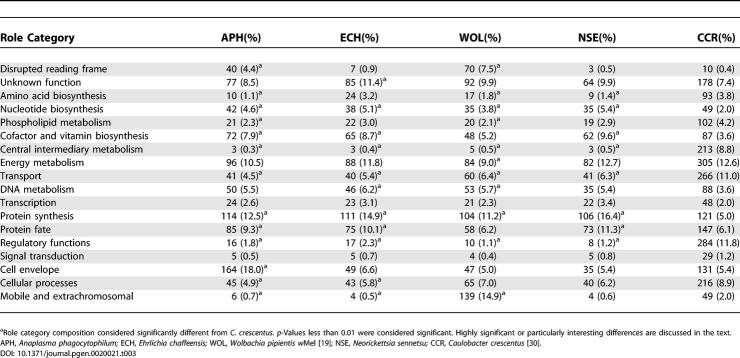
Comparison of Role Category Breakdown

All of the Anaplasmataceae examined have significantly higher percentages of their genomes involved in nucleotide biosynthesis, cofactor and vitamin biosynthesis, and protein synthesis. Enzymes in these biosynthetic pathways are likely to play an important role in interactions with their hosts and intracellular survival, as discussed below. The protein synthesis category includes many essential genes such as those encoding ribosomal proteins, tRNA synthetases, RNA modification enzymes, and translation factors. These genes are essential and cannot be sacrificed as the genome reduces. Therefore, as the genome size decreases, the proportion of genes involved in protein synthesis increases.

All of the Anaplasmataceae examined have a significantly lower coding capacity for central intermediary metabolism, transport, and regulatory functions. The decrease in central intermediary metabolism and transport reflects the differences in acquiring nutrients and energy. Since intracellular bacteria are exposed to a relatively restricted complement of nutrients and energy sources, they have evolved to be specialists in acquiring specific compounds from their hosts.

Likewise, these intracellular bacteria live in a homeostatic environment and have fewer regulatory genes. ORFs encoding σ^70^ and σ^32^ were identified (*rpoD* and *rpoH,* respectively), but σ^24^ and σ^54^ were not detected (*rpoE* and *rpoN,* respectively). Several two-component regulatory systems are retained and may be employed as these bacteria transition between their vertebrate and invertebrate hosts. Despite being identified in *Rickettsia* spp. [21], stringent response (a global regulatory response) may not be expected in the Anaplasmataceae, since neither RelA nor SpoT proteins were identified.

There are several role categories in which only specific organisms have significant differences from, or similarities to, *C. crescentus.* All the bacteria except E. chaffeensis have a statistically significant decrease in amino acid biosynthesis. The difference between *Ehrlichia* spp. and the other Anaplasmataceae is due to the presence of lysine and arginine biosynthesis pathways in *Ehrlichia* spp., as discussed below. A. phagocytophilum has a significant increase in the percentage of genes dedicated to the cell envelope due to expansion of the OMP-1 family in *Anaplasma* spp. (discussed below). W. pipientis has a significantly higher percentage of its genome involved in mobile and extrachromosomal functions due to the unique presence of phage and transposons in its genome [19]. *E. chaffeensis, A. phagocytophilum,* and N. sennetsu have a significant decrease in mobile elements, as they have no intact prophage, no transposable elements, and only a few phage core components (HK97-like portal, major capsid, and prohead protease) scattered throughout their genomes. Lastly, A. phagocytophilum and W. pipientis both have an increased number of disrupted reading frames.

Based on comparisons of the intracellular and free-living α-Proteobacteria, the only overall theme that emerges is the conservation of housekeeping genes and the shuffling of the genomes resulting in the loss of many operon structures.

### Pathogenesis

Little is known about the genetic determinants required for the Rickettsiales to invade a host and cause disease. Putative pathogenesis genes were identified, including enzymes to neutralize reactive oxygen species, outer membrane proteins, and protein secretion systems.

#### Oxidative stress response.

Reactive oxygen species have been implicated in both host defense to infection and host cell injury [31–33]. All of the Rickettsiales contain *sodB,* an iron superoxide dismutase. This superoxide dismutase may have an important role in pathogenesis since *sodB* is cotranscribed with components of the type IV secretion system in E. chaffeensis and A. phagocytophilum [34].

Further examination of conserved genes without functional annotation (e.g., conserved hypothetical proteins, conserved domain proteins) shows two other ortholog clusters of proteins that may be involved in response to oxidative stress—a putative heme copper oxidase and a putative flavohemoglobin. In both cases, there is no significant similarity to a protein of known function, but several conserved domains were identified. From a particular combination of domains and conservation of metal/cofactor ligands, a function of response to oxidative stress can be proposed for these proteins [35].

Indeed, ECH_1079, NSE_0121, and APH_1205 each contain the 12 transmembrane segments and six conserved histidine residues consistent with members of the heme-copper oxidase family. Members of this protein family include cytochrome oxidase subunit I, FixN for nitrogen fixation, and NorB for nitric oxide reduction [36]. Each of these organisms is unlikely to be fixing nitrogen and already has a functional subunit I of cytochrome oxidase (ECH_1003, NSE_0622, and APH_1085), so these orthologs may be nitric oxide reductases. Alternatively, there may be another, as yet to be identified, role for this oxidase, which was identified in all the Rickettsiales genomes except the *Wolbachia* sp. *w*Bm where it is truncated (an ORF that was not annotated but has genomic coordinates from 536343 to 536534).

APH_0545, NSE_0661, and ECH_0778 encode proteins with three functional motifs similar to flavohemoglobins—a heme binding site, an FAD binding domain, and an NAD binding domain. The biological function of the Escherichia coli flavohemoglobin has not been elucidated, but it has been shown to be an efficient alkylhydroperoxide reductase [37] and a nitric oxide reductase [38]. This putative flavohemoglobin is conserved among the Anaplasmataceae, but *Wolbachia* spp. are missing the NAD oxidoreductase domain, and R. prowazekii is missing the heme ligands. Although the speculation of a role for these genes in pathogenicity is intriguing, the precise function of each of these proteins will need to be elucidated experimentally.

#### The OMP-1/MSP2/P44 protein superfamily.

The Anaplasmataceae all have a diverse complement of outer membrane proteins. Many of these outer membrane proteins (OMPs) are members of Pfam PF01617 [39] and constitute the OMP-1/MSP2/P44 family. *Anaplasma, Ehrlichia,* and *Wolbachia* have each undergone variable levels of expansion of their *omp-1/msp2* gene families (Figure S2). The *N. sennetsu* genome has only one uncharacterized protein from this family (NSE_0875). *W. pipientis w*Mel and the *Wolbachia* sp. *w*Bm have the smallest expansion with three *wsp* genes scattered throughout each genome. The largest expansion of this family is in *Ehrlichia* spp. and *Anaplasma* spp. These organisms cannot be transovarially inherited in their arthropod hosts. Instead, ticks acquire *Ehrlichia* or *Anaplasma* by feeding on an infected vertebrate reservoir animal. The expansion of this family may allow persistence in the vertebrate reservoir by providing antigenic variation, thus allowing for effective tick transmission.

*E. chaffeensis, E. canis,* and E. ruminantium have 17–22 paralogous tandemly arranged genes from this family that are flanked by a transcription regulator *(tr1)* and a preprotein translocase *(secA)* [40–42]. These genes all have signal peptides and are likely to be secreted across the cytoplasmic membrane by SecA [42]. They encode immunodominant major outer membrane proteins that are differentially expressed in ticks and experimentally infected animals [43].

A. marginale St. Maries is reported to have 56 genes that have been placed into this superfamily, including eight *msp2,* eight *msp3,* one *msp4,* three *opag,* 15 *omp-1,* 12 *orfX,* seven *orfY,* and two *msp3* remnants [23]. These genes are scattered throughout the genome with a bias in location toward the origin of replication. MSP2 and MSP3 are the immunodominant proteins [44]. The *msp2* and *msp3* gene subsets each include one full-length expression locus and seven reserve/silent sequences that are thought to recombine into the expression locus to generate antigenic variation [23].

The A. phagocytophilum genome has three *omp-1,* one *msp2,* two *msp2* homologs, one *msp4,* and 113 *p44* loci belonging to the OMP-1/MSP2/P44 superfamily. Although both *Anaplasma* spp. *msp2* genes are members of PF01617 and the OMP1/MSP2/P44 superfamily, the *A. marginale msp2* gene is distinct from the *A. phagocytophilum msp2* gene. In addition, the previously identified *omp-1N* is not a member of this Pfam, but is homologous to *E. chaffeensis omp-1N* and the *msp2* operon-associated gene 3 of A. marginale [45].

The largest expansion of this family is that of *p44* genes in A. phagocytophilum. Only 36 copies of *p44* are in this Pfam, but many smaller regions were identified, resulting in a total of 113 annotated *p44* loci (Table S16). The *p44*s consist of a central hypervariable region of approximately 280 bp containing a signature of four conserved amino acid regions (C, C, WP, A) and conserved flanking sequences longer than 50 bp. Diverse *p44* paralogs *(p44–1* to *p44–65)* are expressed in mammals and ticks and confer antigenic environmental adaptation, especially during tick transmission [46–49]. The genomic loci of all 65 previously described *p44* genes were determined in the present study (Figure S3). Twenty-three novel *p44* genes *(p44*-*66* to *p44–88)* were identified by genome sequencing, but have not yet been experimentally identified as being expressed.

**Figure 5:**
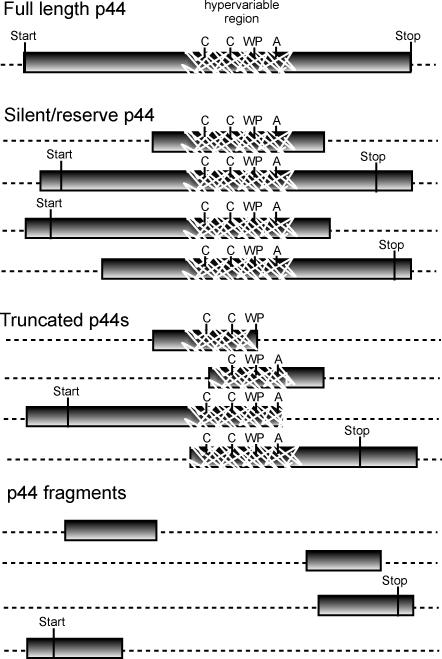
Representative Illustrations of *p44* Genes Full-length *p44* genes contain conserved start and stop codons, an ORF longer than 1,000 bp, and a central hypervariable region of approximately 280 bp containing a signature of four conserved amino acid regions (C, C, WP, A). These genes can be expressed at their respective current genome location or can recombine into the expression locus (*p44ES*/APH_1221). A silent/reserve *p44* is less than 1,000 bp. It may have either the conserved or alternative start and/or stop codons. A silent/reserve *p44* is not likely to be expressed at its current genome location, but can recombine into the expression locus (*p44ES*/APH_1221). Truncated *p44*s carry the complete hypervariable region, or a portion thereof, but only one of the two conserved regions. Fragments of *p44* have only a conserved region and no hypervariable region. Each annotated *p44* is longer than 60 bp. It should be noted that smaller fragments can be identified throughout the genome. These, as well as *p44* truncations and fragments, are likely to be nonfunctional remnants of previous recombination events.

The *p44*s were annotated as full-length, silent/reserve, truncated, and fragments (Figure 5). There are 22 full-length *p44*s identified that have ORFs longer than 1.0 kb with conserved start and stop codons. By locating highly conserved 5′ and 3′ flanking sequences and signature sequences within the hypervariable region, 64 shorter *p44*s were identified. These ORFs lack a translational start codon and likely serve as reserve/silent *p44*s that can be expressed after recombining into the previously described *p44*-expression locus (*p44ES*/APH_1221) [45,50]. The full-length and silent/reserve *p44* genes are preferentially located near the replication origin (Figure S3) and symmetrically located around the *p44* expression locus. Localization near the origin, where multiple replication forks coexist, may facilitate recombination between the expression locus and the reserve/silent *p44* genes.

In addition to the full-length and silent/reserve *p44* genes, 21 5′ and 3′ fragments and six truncations of *p44* genes larger than 60 nucleotides have been identified in the genome. Truncations include portions of a hypervariable region; fragments did not. The *p44*s annotated as truncated and fragments do not contain both conserved regions flanking the hypervariable region. These *p44*s are not expected to recombine through the homologous recombination model deduced by previous analyses of recombined *p44*s [49–52].

Microarray-based comparative genomic hybridization reveals that expansion of the *p44* family is a common feature in A. phagocytophilum strains. All but four of the *p44* unique hypervariable sequences used as targets on the microarray are present in the human isolate A. phagocytophilum MN and the horse isolate A. phagocytophilum California MRK (Figure S3; Table 4). The *p44*-*12* and *p44*-*9* unique regions are either absent or divergent only in strain MN. The *p44*-*4* and *p44*-*1* unique regions are absent or divergent in strains MN and MRK. This confirms previous results demonstrating that the *p44–1* unique region is absent/divergent in MN and MRK [52].

**Table 4:**
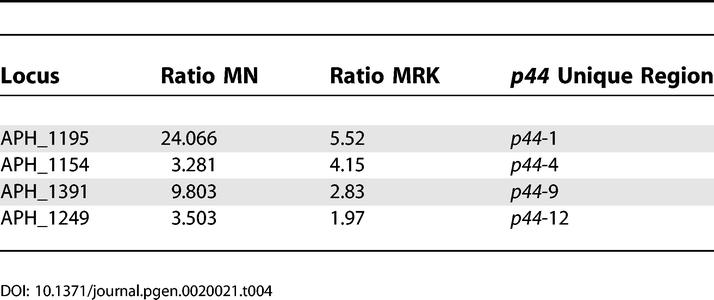
Selected A. phagocytophilum CGH Results

#### Other important outer membrane proteins.

*N. sennetsu* has a single *p51* gene (NSE_0242) encoding its immunodominant P51 major outer membrane protein [14]. The *p51* gene is highly conserved among *N. risticii, N. sennetsu,* and the Stellantchasmus falcatus fluke agent, but not in *N. helminthoeca,* the agent causing an acute, highly fatal salmon-poisoning disease of domestic and wild canines [14]. Although a full-length, highly conserved homolog for P51 was not found in the Rickettsiales genome sequences, P51 was placed in an ortholog cluster of genes conserved among all the Rickettsiales due to short regions of similarity, particularly in a C-terminal region that may include a secretion peptide motif.

Other outer membrane proteins have been reported in *A. marginale,* including *msp5, msp1a,* and *msp1b*. The *msp5* gene (a SCO1/SenC family protein) is found in all the Rickettsiales, whereas *msp1a* and *msp1b* are unique to A. marginale.

Only E. chaffeensis and E. canis encode a 120-kDa immunodominant surface protein (ECH_0039) [53]. The variable-length PCR target useful in distinguishing various strains of E. chaffeensis [54] is present only in the genome of E. chaffeensis Arkansas (ECH_0170).

#### Protein secretion systems.

All of the strains sequenced here contain both a Sec-dependent and Sec-independent protein export pathway for secretion of proteins across the inner membrane. The Sec-independent pathway (Tat pathway) has been implicated in the transport of phospholipases in Pseudomonas aeruginosa [55]. All of the strains sequenced here also contain two components of a putative type I secretion system, potentially for transporting toxins or proteases carrying a C-terminal secretion signal.

All of the Rickettsiales have a type IVa secretion system that uses a complex of transmembrane proteins and a pilus to deliver effector macromolecules from prokaryotic to eukaryotic cells. The reference Type IVa secretion system is that of *Agrobacterium tumefaciens,* which contains 11 genes in the *vir*B locus and one gene in the *vir*D locus. Several components of the A. tumefaciens type IVa secretion system are conserved in *A. phagocytophilum, E. chaffeensis,* and N. sennetsu. Like R. prowazekii and *W. pipientis,* the three organisms sequenced here are lacking *vir*B1, *vir*B5, and *vir*B7. All but N. sennetsu lack *vir*B2.

The *vir*B3, *vir*B4, and *vir*B6 homologs are contiguous at one locus (Figure S4). Neighboring this locus in all of these organisms are three or four *vir*B6 homologs. Contiguous at a second locus are *vir*B8, *vir*B9, *vir*B10, *vir*B11, and *vir*D4. The type IV secretion system is one of the few sets of genes syntenic between all of the Rickettsiales sequenced, suggesting that tight coordination of expression of these genes is critical.

In *A. tumefaciens,* translocated type IV effector proteins have the consensus sequence R-X_7_-R-X-R-X-R-X-X_n_, where lysine can substitute for arginine with no noticeable effect [56]. In addition, effector molecules are often localized to a region of the chromosome near the type IV secretion apparatus. Examination of the regions around the type IV operons in A. phagocytophilum revealed numerous genes encoding HGE-14, which contain C-terminal sequences similar, but not identical, to this motif (Table S17), suggesting that it may be an excreted effector molecule. Subsequent searches of the Anaplasmataceae genomes with motifs like that found in HGE-14 did not reveal other potential effector molecules.

### Metabolism

**Figure 6:**
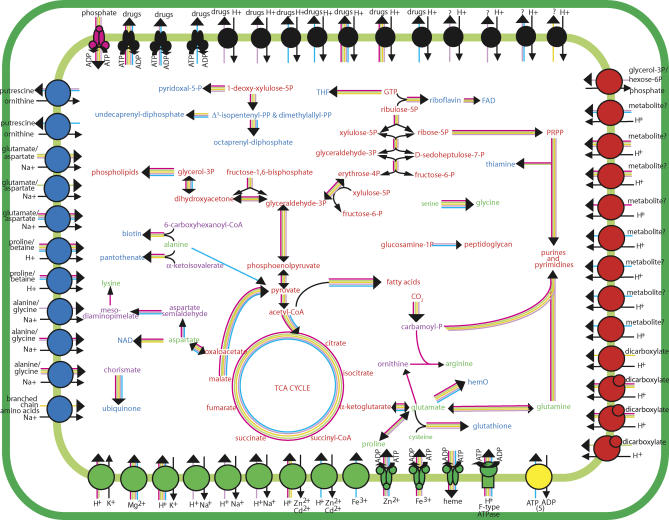
Comparative Metabolic Potential of Select Rickettsiales Metabolic pathways of E. chaffeensis (magenta arrows), A. phagocytophilum (green arrows), N. sennetsu (gold arrows), W. pipientis (lavender arrows), and R. prowazekii (cyan arrows) were reconstructed and compared. The networks of some of the more important pathways are shown with metabolites color coded: red and purple, central and intermediary metabolites; blue, cofactors; green, amino acids; and black, cell structures. Transporters are shown in the membrane and are grouped by predicted substrate specificity: green, inorganic cations; magenta, inorganic anions; red, carbohydrates and carboxylates; blue, amino acids/peptides/amines; yellow, nucleotides/nucleosides; and black, drug/polysaccharide efflux or unknown.

The metabolic potentials of *A. phagocytophilum, E. chaffeensis,* and N. sennetsu were compared to that of R. prowazekii and W. pipientis [18,19]. Overall, the Anaplasmataceae have very similar metabolic pathways but are quite distinct from those of R. prowazekii (Figure 6). W. pipientis differs from the other Anaplasmataceae in its inability to synthesize some cofactors.

#### Nucleotide and cofactor biosynthesis.

*E. chaffeensis, A. phagocytophilum, N. sennetsu,* and W. pipientis have the ability to synthesize all nucleotides. This differs from *R. prowazekii,* which cannot make purines or pyrimidines, and therefore must rely on nucleotide translocases and interconversion of the bases to obtain the full complement of nucleotides [18]. *E. chaffeensis, A. phagocytophilum,* and N. sennetsu are able to synthesize most vitamins and cofactors. In contrast to the other Anaplasmataceae, W. pipientis has lost some of its ability to synthesize cofactors, and it has completely lost the biosynthetic pathways for biotin, thiamine, and NAD. In addition, it may be in the process of losing the ability to synthesize folate. R. prowazekii has also lost the ability to synthesize these cofactors as well as FAD, pantothenate, and pyridoxine-phosphate.

Biotin is one of the essential cofactors only synthesized by the vertebrate-infecting Anaplasmataceae. In most organisms, biotin is required for many carboxylation reactions, but is not synthesized by many multicellular eukaryotes. RT-PCR analysis showed that all four genes in the biotin biosynthesis pathway *(BioA/B/D/F)* were expressed by E. chaffeensis and A. phagocytophilum in THP-1 and HL-60 cells, respectively, at both 2 d and 3 d post infection (Figure S5).

The presence of nucleotide, vitamin, and cofactor biosynthetic pathway in *E. chaffeensis, A. phagocytophilum,* and *N. sennetsu* suggests that they do not need to compete with the host cell for, and may even supply host cells with, essential vitamins and nucleotides. It has been previously proposed that *Wigglesworthia glossinidia* supplies its host with vitamins that are rare in the blood meal of its arthropod host (tsetse fly) [57]. Interestingly, *Ehrlichia* spp. and *Anaplasma* spp., the two tick-borne intracellular pathogens sequenced, both have a complement of pathways for cofactor and amino acid biosynthesis similar to *W.* *glossinidia* (Table 5). This raises the possibility that these pathogens may currently be, or historically have been, able to provide a benefit to their tick hosts by providing necessary cofactors.

**Table 5:**
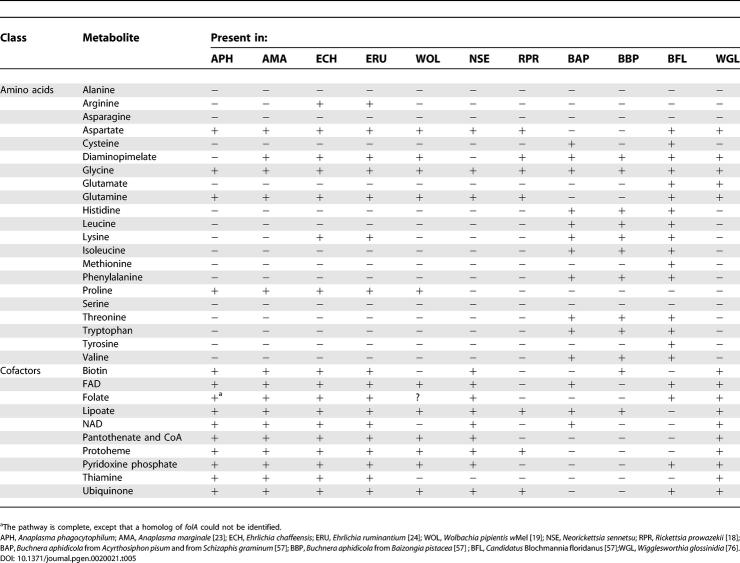
Amino Acid and Cofactor Biosynthesis in Intracellular Bacteria

#### Amino acid biosynthesis.

The Rickettsiales have a very limited ability to synthesize amino acids and must rely on transporting them from the host (Figure 6). All four of the Anaplasmataceae sequenced have the ability to make glycine, glutamine, glutamate, and aspartate. Additionally, E. chaffeensis is predicted to be able to synthesize arginine and lysine like E. ruminantium [24]. One possible role for arginine biosynthesis may be to recover an intracellular arginine pool after exposure to inducible host nitric oxide. Nitric oxide is synthesized by nitric oxide synthases that convert arginine to citrulline and nitric oxide [58]. The production of nitric oxide is likely to deplete the intracellular pool of arginine, further hampering intracellular growth. The presence of an arginine biosynthesis pathway and putative nitric oxide reductase(s) may allow *Ehrlichia* spp. to recover more rapidly and subvert the host immune response. This would be similar to the proposed retention of select tryptophan biosynthetic genes in *Chlamydia* spp. in order to replenish tryptophan pools after host enzymatic degradation of tryptophan in response to IFN-γ [59].

#### Glycolysis, tricarboxylic acid cycle, pentose phosphate, and respiration.

A complete pyruvate dehydrogenase, tricarboxylic acid cycle, F_0_F_1_-ATPase, and electron transport chain were found in all of the organisms. All five organisms are likely to use host-derived carboxylates and amino acids, but none of these organisms can obtain carbon or energy from fatty acids or actively carry out glycolysis. The glycolysis enzymes present are limited to those that produce glyceraldehyde-3-phosphate and dihydroxyacetone phosphate from phosphoenolpyruvate (Figure 6). The glyceraldehyde-3-phosphate produced in this manner is used in the nonoxidative pentose phosphate pathway, resulting in the production of pentoses needed for cofactor and nucleotide biosynthesis. Consistent with this role for the glycolytic enzymes, R. prowazekii and R. conorii retain neither the glycolytic enzymes nor the enzymes needed for the biosynthesis of nucleotides or cofactors from pentose. Similarly, dihydroxyacetone phosphate from these glycolytic enzymes can be converted to glycerol-3-phosphate for phospholipid biosynthesis in the Anaplasmataceae. Without the glycolytic enzymes, *Rickettsia* spp. must obtain glycerol-3-phosphate from the host via a glycerol-3-phosphate transporter.

### Evolution and DNA Repair

A genome-scale phylogenetic analysis using a concatenated alignment of core proteins is consistent with rRNA studies and current taxonomic assignments. This indicates that *Anaplasma* and *Ehrlichia* are sister genera that share a common ancestor with *Wolbachia* (Figure 1). *Neorickettsia* is the deepest-branching lineage in the group.

The branch lengths on the whole genome tree can be used to get an indication of the relative rates of evolution of these organisms. In general, the branch lengths for these intracellular organisms are longer than those of their free-living relatives. This may be due to either differences in DNA repair or population genetic and selection-related force. For example, many intracellular organisms go through more stringent population bottlenecks, which in turn increase the amount of genetic drift and possibly the rate of accumulation of deleterious mutations.

Analysis of the genome of *W. pipientis w*Mel revealed that it had a longer branch length than the closely related *Rickettsia;* the *Rickettsia* have higher rates of evolution than free-living organisms [19]. Wu et al. [19] ascribed this increase to features of *Wolbachia* biology. However, there appears to be a general increase in the rate for all of the Anaplasmataceae (Figure 1). Thus, the increase reported for *Wolbachia* [19] is not likely due to the specific biology of *Wolbachia,* but instead to some feature shared by all Anaplasmataceae.

Examination of the putative DNA-repair capabilities of the different species does not reveal any significant differences between the Anaplasmataceae and the *Rickettsia* spp. (Table S18). Interestingly, within the Anaplasmataceae, N. sennetsu appears to have the longest branch length and the most limited suite of DNA repair genes within the group. For example, N. sennetsu is missing various glycosylases and exonucleases that contribute to repair, including *uvrABC,* which is involved in nucleotide excision repair. It is possible that the faster rate of evolution in this organism is related to the absence of some of these repair pathways.

The absence of *uvrABC* in N. sennetsu and the absence of *uvrBC* in the *Ehrlichia* spp. suggest that these species do not have nucleotide excision repair (NER). NER is used by other organisms, including bacteria, archaea, and eukaryotes, as a general repair process to remove sections of DNA with gross abnormalities. One important role of NER is in the repair of UV-induced DNA damage, and defects in NER in other species lead to great increases in UV sensitivity. It appears that *Neorickettsia* has compensated for this by acquiring a gene homologous to DNA photolyases, an alternative mechanism for repairing UV damage. The *Neorickettsia* photolyase is not particularly closely related to known photolyases from α-Proteobacteria but is instead most closely related to a photolyase from Coxiella burnetii, a γ-Proteobacteria. The *Ehrlichia* spp., however, do not encode a photolyase homolog, and thus these species may be highly UV-sensitive.

### Conclusions

The dual existence of members of *Anaplasma* spp. and *Ehrlichia* spp. as invertebrate symbionts or commensals and effective human and animal pathogen requires flexibility, a fact reflected in the genome. Both organisms display an expansive inventory of paralogous genes encoding diverse functions that promote survival and success in different environments when compared to *Neorickettsia* spp. and *Wolbachia* spp., which do not require a mammalian host. This capacity is evident from the large repertoire of outer membrane proteins, and partial duplication of some of the virulence determinants (e.g., components of the type IV protein secretion system).

The large number of paralogous genes encoding immunodominant outer membrane proteins in *Anaplasma* spp. and *Ehrlichia* spp. has important implications for the study of pathogenesis and in the development of vaccination strategies. Adaptability in the human host may underlie significant disease manifestations. Genomic-level characterization of the full complement of variable antigens will facilitate the future development of more specific and sensitive diagnostic targets. In light of the growing recognition of the increased global burden of ehrlichiosis, development of such diagnostic targets will impact public health.

Between pairwise comparisons of different species within a single genus, there are hundreds of genes that are not shared. Often these gene differences are immunodominant outer membrane proteins, but the vast majority are genes that are not functionally characterized in any organism. Some are likely to be involved in zoonosis or specific disease characteristics. For instance, A. phagocytophilum is the only sequenced Rickettsiale that infects neutrophils. Therefore, some of the A. phagocytophilum-unique genes (e.g., genes encoding P44 and HGE-14) may be involved in neutrophil invasion.

Many pathogens are obligate intracellular bacteria. But since they are difficult or impossible to culture and tools for genetic manipulation are limited, they are less well characterized than the facultative intracellular bacteria or extracellular pathogens. The analysis of the genome sequences provides critical insights into the biology of these intracellular pathogens and will facilitate manipulation of the emerging human ehrlichiosis agents and leukocytotropic pathogens.

## Materials and Methods

### Intracellular bacteria purification and DNA preparation.

Organisms (infecting ~1 × 10^9^ host cells; 50–100 175-cm^2^ flasks) were cultured in synchrony in respective host cells (E. chaffeensis in DH82 cells, A. phagocytophilum in HL-60 cells, and N. sennetsu in P388D_1_ cells). Bacterial cells were liberated from the infected host cells using Dounce homogenization, differential centrifugation, and Percoll density gradient centrifugation [60]. Any specimens with host nuclei contamination were excluded. From these isolated bacteria, phenol extraction was used to purify DNA that was minimally fragmented and free of host-cell DNA. Levels of host DNA contamination were verified to be less than 0.001% by PCR using host G3PDH-specific primers. This method was highly successful, with only 14 sequencing reads identified as being of human origin from a total of over 57,000 good sequencing reads.

### Sequencing and annotation.

The complete genome sequences were determined using the whole-genome shotgun sequencing approach [61], sequences were assembled into contigs using the Celera Assembler [62], and all gaps were closed [63]. ORFs from each genome were predicted and annotated using a suite of automated tools that combine Glimmer gene prediction [64,65], ORF and non-ORF feature identification (e.g., protein motifs), and assignment of database matches and functional role categories to genes [63]. Frameshifts and point mutations were detected and corrected where appropriate; those remaining were annotated as “authentic frameshift” or “authentic point mutation.” Repeats were identified using RepeatFinder [66,67] and were manually curated. The complete genome sequences for A. phagocytophilum HZ, E. chaffeensis Arkansas, and N. sennetsu Miyayama have been deposited in GenBank.

### Annotation of the *p44* genes.

Full-length *p44*s were defined as having ORFs greater than 1,000 bp with conserved start codon and stop codons. For shorter silent/reserve *p44*s, the ORFs were initially identified by locating highly conserved 5′ and 3′ sequences and signature sequences within the hypervariable region. Since these silent/reserve *p44*s lack a start and stop codon, the 5′ and 3′ ends were annotated on the basis of conserved genome features found in full-length *p44* genes [50,68]. The annotated *p44* fragments are at least 60 nucleotides in length, have either 5′ or 3′ conserved sequences, and may contain a partial hypervariable region (Figure 5).

### Genome comparisons.

Ortholog clusters were delineated for R. prowazekii Madrid E [18], R. typhi Wilmington [21], R. conorii Malish 7 [20], N. sennetsu Miyayama, *Wolbachia* sp. *w*Bm [22], *W. pipientis w*Mel [19], E. chaffeensis Arkansas, E. ruminantium Gardel (GenBank CR925677.1), E. ruminantium Welgevonden [24], A. marginale St. Maries [23], A. phagocytophilum HZ, Brucella suis 1330 [69], Bartonella henselae Houston-1 [70], Coxiella burnetii RSA 493 [71], Tropheryma whipplei Twist [72], Blochmannia floridanus [73], *Buchnera* sp. APS [74], Chlamydia pneumoniae AR39 [75], and *W. glossinidia brevipalpis* [76]. For *Wolbachia* sp. *w*Bm, the ORFs used in these comparisons were uncurated ORFs predicted and annotated using a suite of automated tools that combine Glimmer gene prediction [64,65], ORF and non-ORF feature identification (e.g., protein motifs), and assignment of database matches and functional role categories to genes [63]. Upon release of the annotated genome [22], these uncurated ORFs were paired with the corresponding curated ones where possible, with exceptions noted in the text.

Paralog clusters within each of the genomes were identified using the Jaccard algorithm with the following parameters: 80% or greater identity and Jaccard coefficient 0.6 or higher [77, Text S1]. Members of paralog clusters were then organized into ortholog clusters by allowing any member of a paralog cluster to contribute to the reciprocal best matches used to construct the ortholog clusters. The conservation of ortholog clusters across the various genomes analyzed was determined using Sybil, a web-based software package for comparative genomics developed at TIGR (http://sybil.sourceforge.net). The database of these clusters and corresponding tools can be accessed through TIGR (http://www.tigr.org/sybil/rcd). Metabolic pathways and transporters were compared across genomes using (1) these calculated ortholog clusters, (2) Genome Properties [78], (3) TransportDB [79], and (4) Biocyc [80].

Significant differences in the role category composition was determined using χ^2^ calculated using the Yates continuity correction. A *p*-value less than 0.01 was considered significant.

### GC-skew and origin prediction.

The GC-skew was calculated as (C − G)/(C + G) in windows of 1,000 bp along the chromosome [81]. The origin of replication was not experimentally determined in any of the genomes. For E. chaffeensis and *N. sennetsu,* a clear shift in GC-skew occurs near *parA* and *parB*. Therefore basepair 1 was set in the intergenic region between the two genes. In *A. phagocytophilum,* a GC-skew transition occurs near *polA.* Therefore, basepair 1 was set in the intergenic region near *polA*.

### Atypical nucleotide composition.

Regions of atypical nucleotide composition were identified by the χ^2^ analysis: the distribution of all 64 trinucleotides was computed for the complete genome in all six reading frames, followed by the trinucleotide distribution in 5,000-bp windows overlapping by 500 bp. For each window, the χ^2^ statistic was computed based on the difference between the trinucleotide content in that window and that of the whole genome. Peaks indicate regions of atypical nucleotide composition.

### Genome tree construction.

Protein sequences of 31 housekeeping genes *(frr, infC, nusA, pgk, pyrG, rplA, rplB, rplC, rplD, rplE, rplF, rplK, rplL, rplM, rplN, rplP, rplS, rplT, rpmA, rpoB, rpsB, rpsC, rpsE, rpsI, rpsJ, rpsK, rpsM, rpsS, smpB,* and *tsf)* from complete α-Proteobacteria genomes were aligned to predefined HMM models and ambiguous regions were autotrimmed according to an embedded mask. Concatenated alignments were then used to build a maximum likelihood tree with bootstrapping using PHYML [82]. The γ-Proteobacteria E. coli and the β-Proteobacteria Neisseria meningitidis were used as outgroups to root the tree.

### Array construction and hybridizations.

Oligonucleotides (70-mer) were designed from the unique ORFs of each of the three genomes. The oligonucleotides (Illumina, San Diego, California, United States) were diluted to 25 μM in DMSO and spotted in quadruplicate onto UltraGap slides (Corning, Acton, Massachusetts, United States). Cy3 and Cy5 probes were synthesized from genomic DNA as previously described [83]. In order to obtain enough DNA for microarray analysis, small amounts of DNA were prepared in the manner described above for genome sequencing. This DNA was then quantitatively amplified using GenomiPhi (Amersham, Piscataway, New Jersey, United States).

Appropriately labeled query and reference probes were hybridized overnight, washed, and scanned using an Axon GenePix 4000B scanner (Axon Instruments, Union City, California, United States). The corresponding images were analyzed with TIGR Spotfinder [84]. Log mode centering was used to normalize the data alleviating the bias of expression microarray normalization methods, which expect a normal distribution of data. Briefly, a Perl script was designed to construct the histogram of the log_2_ of the ratio and adjust the histogram mode to zero. The data presented are the geometric means of the normalized ratios from at least two slides with different reference Cy dyes and with oligonucleotides printed in quadruplicate.

### Transcript analysis of biotin biosynthetic genes.

Total RNA was extracted from E. chaffeensis or A. phagocytophilum-infected THP-1 or HL-60 cells at 2 d or 3 d postinfection using RNeasy (Qiagen, Valencia, California, United States). RNA was DNase I treated (Invitrogen, Carlsbad, California, United States) in the presence of 40 U of RNaseOUT (Invitrogen) for 15 min at room temperature, followed by inactivation at 65 °C in the presence of 2.5 mM EDTA for 10 min. For cDNA synthesis, total RNA (0.5 μg) was reverse-transcribed at 42 °C for 1 h in 50 mM Tris-HCl (pH 8.3), 75 mM KCl, 3 mM MgCl_2_, 0.5 mM of each dNTP, 1 U of RNase inhibitor (Invitrogen), 1.5 μM random hexamers (Invitrogen), and 10 U of Superscript II reverse transcriptase (Invitrogen). The reaction was terminated by heat inactivation at 70 °C for 15 min. To ensure the absence of DNA contamination in the RNA preparations, the assay was duplicated without reverse transcriptase. The subsequent amplification was conducted with standard conditions for 25 cycles of 95 °C for 45 s, 54 °C for 45 s, and 72 °C for 1 min and with the PCR primer pair (Table S19).

## Supporting Information

Figure S1 Linear Representation of E. chaffeensis Arkansas, A. phagocytophilum HZ, and N. sennetsu Miyayama Genomes ORFs are oriented along the molecule and are color-coded by role category: violet, amino acid biosynthesis; light blue, biosynthesis of cofactors, prosthetic groups, and carriers; light green, cell envelope; red, cellular processes; brown, central intermediary metabolism; yellow, DNA metabolism; light gray, energy metabolism; black, mobile/extrachromosomal functions and truncated ORFs; magenta, fatty acid and phospholipid metabolism; pink, protein synthesis and fate; orange, purines, pyrimidines, nucleosides, and nucleotides; olive, regulatory functions and signal transduction; dark green, transcription; teal, transport and binding proteins; gray, unknown function; crosshatched, conserved hypothetical proteins; white, hypothetical proteins; salmon, other categories. The tRNA genes and rRNA genes are represented by their secondary structure. Repeats are shown by dual-arrowed line segments; paralogs are represented by arrows.
(10.4 MB PDF)

Figure S2 Phylogenetic Tree of OMP1 Proteins The protein sequences of all the members of PFAM01617 were aligned and a phylogenetic tree inferred. The divergence of the OMP1/MSP2/P44 proteins in this superfamily did not permit robust inferences about the evolution of these proteins, but allowed classification of the proteins into superfamilies as reflected in their annotation. Particular families within this superfamily are highlighted, including the P44 proteins (pink), the OMP-1s (blue), and the Wsp (yellow).
(876 KB PDF)

Figure S3 Distribution of the *p44* Genes in A. phagocytophilum Strains Rims 1 and 2: predicted coding regions on the plus and minus strands, respectively, color-coded by role categories. Rim 3: atypical nucleotide composition. Rim 4: distribution of *p44* silent genes (green), expression locus (cyan), full-length genes (magenta), fragments (brown), and truncations (blue). Rims 5 and 6: microarray-based comparative genome hybridization results for A. phagocytophilum strain HZ against strains MRK and MN. The ratios [(HZ normalized intensity)/(query normalized intensity)] were divided into three categories: ratio > 10 (red; absent); ratio 3–10 (blue; absent/divergent); and ratio < 3 (not plotted; present).
(1.0 MB PDF)

Figure S4 Type IV Secretion Systems in Rickettsiales Genes encoding the type IV secretion system components can be found at two distinct regions of the Rickettsiales genome. At the larger of these regions, *virB3, virB4,* and *virB6* show a typical arrangement. These are followed by a series of genes in the *virB6* family that have been shown to be cotranscribed in *W. pipientis w*Mel. Each of these regions is presented with ortholog clusters (see Materials and Methods) and color coded: cyan, *virB3;* orange, *virB4;* green, *virB6;* and purple, a *virB6* family of genes. Orthologs conserved in location are connected with gray bars. The *virB3* gene is not always annotated, due to its small size, but it is present in all Rickettsiales genomes examined.
(616 KB PDF)

Figure S5 Transcript Analysis of Biotin Biosynthetic Genes DNase-treated total RNA was reverse-transcribed and subsequently PCR amplified using primers specific to each biotin biosynthesis gene. RT-PCR analysis showed that all four genes in the biotin biosynthesis pathway *(BioA/B/D/F)* were expressed by E. chaffeensis and A. phagocytophilum in THP-1 and HL-60 cells, respectively, at 2 d (unpublished data) and 3 d postinfection.
(61 KB JPG)

Table S1 Ortholog Clusters Conserved across All Representative Obligate and Facultative Intracellular Pathogens and Endosymbionts Presented in a Tab-Delimited Format (640 KB DOC)

Table S2 Ortholog Clusters Present in All the Rickettsiales but Not in Any Other Intracellular Bacterium Examined (49 KB DOC)

Table S3 Ortholog Clusters Present in All Anaplasmataceae but Not the Rickettsiales or Other Intracellular Bacterium Examined (47 KB DOC)

Table S4 Ortholog Clusters Present in All of Five Representatives of the Genera in the Rickettsiales That Have at Least One Representative Sequenced The following genomes were compared: *Rickettsia prowazekii, Neorickettsia sennetsu, Wolbachia pipientis, Anaplasma phagocytophilum,* and Ehrlichia chaffeensis.
(424 KB DOC)

Table S5 Ortholog Clusters Present in *Neorickettsia sennetsu, Anaplasma phagocytophilum,* and *Ehrlichia chaffeensis,* but Not in Wolbachia pipientis and Rickettsia prowazekii
(44 KB DOC)

Table S6 Ortholog Clusters Present in *Wolbachia pipientis, Anaplasma phagocytophilum, *and *Ehrlichia chaffeensis,* but Not in Neorickettsia sennetsu and Rickettsia prowazekii
(54 KB DOC)

Table S7 Ortholog Clusters Present in Rickettsia prowazekii and *Wolbachia pipientis,* but Not in *Neorickettsia sennetsu, Anaplasma phagocytophilum,* or Ehrlichia chaffeensis
(41 KB DOC)

Table S8 Ortholog Clusters Present in Anaplasma phagocytophilum and *Ehrlichia chaffeensis,* but Not in *Rickettsia prowazekii, Wolbachia pipientis,* or Neorickettsia sennetsu
(46 KB DOC)

Table S9 Individual Genes (Based on Ortholog Cluster Analysis) Present in *Anaplasma phagocytophilum,* but Not in *Ehrlichia chaffeensis, Rickettsia prowazekii, Wolbachia pipientis,* or Neorickettsia sennetsu
(394 KB DOC)

Table S10 Individual Genes (Based on Ortholog Cluster Analysis) Present in Ehrlichia chaffeensis but Not in *Anaplasma phagocytophilum, Rickettsia prowazekii, Wolbachia pipientis,* or Neorickettsia sennetsu
(244 KB DOC)

Table S11 Individual Genes (Based on Ortholog Cluster Analysis) Present in Neorickettsia sennetsu but Not in *Anaplasma phagocytophilum, Rickettsia prowazekii, Wolbachia pipientis,* or Ehrlichia chaffeensis
(223 KB DOC)

Table S12 Ortholog Clusters Present Only in Anaplasma phagocytophilum and Anaplasma marginale
(57 KB DOC)

Table S13 Ortholog Clusters Present Only in *Ehrlichia chaffeensis, Ehrlichia ruminantium* Welgevonden, and Ehrlichia ruminantium Gardel (82 KB DOC)

Table S14 Ortholog Clusters That Are Absent in All of the Tick-, Flea-, and Louse-Borne Rickettsiales, but Are Present in *Wolbachia* spp. and Neorickettsia sennetsu
(27 KB DOC)

Table S15 Ortholog Clusters Present in All the Pathogenic Rickettsiales but None of the Endosymbionts (30 KB DOC)

Table S16 *Anaplasma phagocytophilum p44* Genes (246 KB DOC)

Table S17 Putative Anaplasma phagocytophilum Type IV Effector Motifs in HGE-14 Proteins (27 KB DOC)

Table S18 DNA Repair Genes (110 KB DOC)

Table S19 Sequences of Oligonucleotides Used for RT-PCR (31 KB DOC)

Text S1 PDF copy of Jaccard et al. reference (4578 KB PDF)

### Accession Numbers

The GenBank (http://www.ncbi.nlm.nih.gov) accession number for the Ehrlichia canis Jake shotgun sequence is ZP_00210380; the complete GenBank genome sequences for A. phagocytophilum HZ, E. chaffeensis Arkansas, and N. sennetsu Miyayama are CP000235, CP000236, and CP00237, respectively.

The ArrayExpress database (http://www.ebi.ac.uk/arrayexpress) accession numbers for the microarray slide type and study are E-TIGR-125 and A-TIGR-21.

## Abbreviations

HGA: human granulocytic anaplasmosis
HGE: human granulocytic ehrlichiosis
HME: human monocytic ehrlichiosis
NER: nucleotide excision repair
OMP: outer membrane protein
ORF: open reading frame
