# Correction: Comparative Genomics of Emerging Human Ehrlichiosis Agents

**DOI:** 10.1371/journal.pgen.0020213

**Published:** 2006-12-29

**Authors:** Julie C Dunning Hotopp, Mingqun Lin, Ramana Madupu, Jonathan Crabtree, Samuel V Angiuoli, Jonathan A Eisen, Rekha Seshadri, Qinghu Ren, Martin Wu, Teresa R Utterback, Shannon Smith, Matthew Lewis, Hoda Khouri, Chunbin Zhang, Hua Niu, Quan Lin, Norio Ohashi, Ning Zhi, William Nelson, Lauren M Brinkac, Robert J Dodson, M. J Rosovitz, Jaideep Sundaram, Sean C Daugherty, Tanja Davidsen, Anthony S Durkin, Michelle Gwinn, Daniel H Haft, Jeremy D Selengut, Steven A Sullivan, Nikhat Zafar, Liwei Zhou, Faiza Benahmed, Heather Forberger, Rebecca Halpin, Stephanie Mulligan, Jeffrey Robinson, Owen White, Yasuko Rikihisa, Hervé Tettelin

In *PLoS Genetics,* volume 2, issue 2: 10.1371/journal.pgen.0020021

The original research article mistakenly listed Jonathan A. Eisen without his middle initial.

The original copyright did not indicate that the article is part of the public domain; the correct copyright statement is:

This is an open-access article distributed under the terms of the Creative Commons Public Domain declaration which stipulates that, once placed in the public domain, this work may be freely reproduced, distributed, transmitted, modified, built upon, or otherwise used by anyone for any lawful purpose.

Additionally, in the Accession Numbers section, the *Neorickettsia sennetsu* accession number is CP000237 instead of the published CP00237.

