# Supplementary material for: Comparative Genomics of Emerging Human Ehrlichiosis Agents
Source: PLoS Genet. 2006 Feb 17;2(2):e21. doi: 10.1371/journal.pgen.0020021 (PMC1366493; doi:10.1371/journal.pgen.0020021)

Tableau No 1. Répartition de 92 espèces sur 52 m² groupés en 9 localités (Prairie subalpine).

No des espèces	No des localités →	Nombre [illegible]	I.								II.						III.								IV.						V.						VI.				VII.					VIII.							IX.	
	Nos des m² explorés →		1	2	3	4	5	6	7	8	1	2	3	4	5	6	1	2	3	4	5	6	7	8	1	2	3	4	5	6	1	2	3	4	5	6	1	2	3	4	1	2	3	4	5	1	2	3	4	5	6	7	1	2
1	Achillea millefolium	9																X	X	X	X				X			X	X	X							X																	
2	Agrostis canina	12					X		X	X																	X	X	X	X											X	X	X	X	X									
3	Agropyrum repens	1														X																																						
4	Ajuga reptans	3																																				X	X	X														
5	Alchemilla pratensis	45	X	X			X	X	X	X	X	X	X	X	X		X	X	X	X	X	X	X	X	X	X	X	X	X	X	X	X	X	X	X	X	X	X	X	X	X	X	X	X	X	X	X	X	X			X		
6	Alectorolophus hirsutus	26	X	X	X	X	X	X	X	X	X	X	X	X	X	X																										X	X	X	X	X	X	X	X	X	X	X	X		
7	— " — minor	8						X				X																										X	X	X	X													X	X
8	Anthoxanthum odoratum	28			X	X	X	X	X	X			X		X	X	X	X	X	X	X	X	X	X			X	X	X	X		X	X		X	X				X								X	X					
9	Anthyllis vulneraria	6	X																																			X	X	X													X	X
10	Anthriscus sylvestris	27			X		X	X	X		X	X		X	X		X	X	X	X		X	X	X	X	X	X	X	X	X	X	X	X	X	X	X																	
11	Aposeris foetida	1															X																																			
12	Astrantia major	1																					X																													
13	Avena pubescens	28	X	X				X	X	X	X	X	X	X	X														X				X			X	X	X	X	X	X	X		X		X	X	X	X	X			X	X
14	Bellis perennis	6	X		X	X	X		X	X																																											
15	Biscutella laevigata	1																																																			X
16	Briza media	18	X	X	X	X	X	X				X	X	X		X														X											X			X	X		X	X			X	X	
17	Brachypodium pinnatum	1																																																	X	
18	Bromus erectus	13										X	X						X	X		X		X	X	X	X	X	X																					X	X		
19	Brunella vulgaris	28	X	X	X	X		X	X	X	X	X	X	X	X	X	X	X	X		X	X	X	X	X	X											X	X	X	X								X				X		
20	Campanula rhomboidalis	40	X	X	X	X				X	X	X	X		X	X	X	X	X	X	X	X	X	X			X	X	X	X	X	X	X	X	X	X			X	X	X	X	X	X	X	X		X		X	X	X		
21	— " — rotundifolia	7					X	X	X	X																											X	X	X															
22	Carex pallescens	5															X	X						X														X	X														
23	— " — sempervirens	10	X	X	X	X	X	X		X						X																																					X	X
24	Carlina acaulis	2																																																		X	X
25	Carum carvi	26	X	X		X		X			X	X	X	X	X	X																					X	X	X	X	X	X	X	X	X	X	X	X	X	X	X	X		
26	Cerastium caespitosum	17	X				X	X		X							X	X	X	X		X	X	X								X													X	X	X	X	X				
27	Centaurea jacea	9						X			X																											X			X							X		X	X		X	X
28	— " — montana	4										X		X		X													X																								
29	Cirsium oleraceum	2								X																															X												
30	Colchicum autumnalis	33	X	X	X	X	X	X	X	X	X	X	X	X	X	X									X												X	X	X	X	X	X	X	X	X	X	X	X	X	X	X	X	X	X
31	Crepis taraxacifolia	19					X	X		X				X	X	X							X		X	X	X	X	X	X	X	X	X	X	X	X																	
32	— " — aurea	1																																						X														
33	— " — paludosa	2															X	X																																		
34	Cynosurus cristatus	26	X	X	X	X	X	X		X	X		X	X	X					X				X	X	X	X	X	X	X	X	X	X	X	X	X															X			
35	Chrysanthem. leucanthem.	44	X	X		X	X		X	X	X	X	X	X		X	X	X		X	X	X	X	X	X	X	X	X	X	X	X	X	X	X		X	X	X	X	X	X		X	X	X	X	X		X	X	X	X		X
36	Dactylis glomerata	42		X	X	X	X	X		X	X	X	X	X	X	X		X	X	X	X	X	X	X	X		X	X	X	X	X	X	X	X	X	X					X	X	X	X	X	X	X	X	X	X	X	X		
37	Deschampsia caespitosa	11															X		X	X	X	X	X	X																					X				X	X	X		
38	Equisetum arvense	6	X	X	X	X			X	X																																										
39	Festuca pratensis	43	X	X	X	X		X	X	X			X	X	X	X		X	X	X	X	X	X	X	X	X	X	X		X	X	X	X	X	X	X					X	X	X	X	X	X	X	X	X	X	X	X	X	X
40	— " — ovina	9								X								X	X	X	X																X	X	X	X													
41	Galium asperum (silvestre)	6							X				X	X		X								X						X																							
42	Gentiana campestris	6																																		X	X	X	X													X	X
43	— " — verna	2																																		X	X															
44	Geranium silvaticum	30						X	X	X	X	X	X	X	X	X	X	X	X	X	X	X	X	X	X	X	X	X	X	X	X	X	X	X	X	X	X																
45	Geum urbanum	3					X		X	X																																										
46	Hieracium pilosella	2																																																	X	X
47	Hippocrepis comosa	2																X		X																																
48	Hypericum quadrangul.	1														X																																				
49	Juncus lamprocarpus	1																					X																													
50	Knautia arvensis	14									X	X	X	X	X	X	X	X		X	X	X	X	X																							X					
[illegible]	[illegible]	[illegible]	X	X		X	X	X				X	X			X	X					X	X	X	X	X	X	X	X		X	X	X	X		X	X	X	X	X	X		X		X	X	X	X	X	X		X		

No.	Species	Total	Occurrences
13	Avena pubescens	26	^^ ^^^^^^^^ ^ ^ ^^^^^^X X ^
14	Bellis perennis	6	X XXX XX X
15	Biscutella laevigata ..	1	X XX XX XX
16	Briza media	18	XXXXXX XXX X X X X
17	Brachypodium pinnatum	1	XX XX X XXXXXX XX
18	Bromus erectus	13	XXXX XXXXXXXXXXXX XXXXXX XXXX X X
19	Brunella vulgaris	28	XXXX XXXXXXXXXXXX XXXXXX XXXX X X XXX
20	Campanula rhomboïdalis	40	XXXX XXXX XXXXXXXXXX XXXXXXXXX XXXXXXX X XXX
21	—— " —— rotundifolia	7	XXXX XXX
22	Carex pallescens	5	XX X XX XX
23	—— " —— sempervirens ..	10	XXXXXX X X XX
24	Carlina acaulis	2	XXXXXXXXXXXXXXX
25	Carum carvi	26	XX X X XXXXXX XXXXX
26	Cerastium caespitosum .	17	X XX X XXXX XXX X X X X XX XX
27	Centaurea jacea	9	X X
28	—— " —— montana	4	X X X X
29	Cirsium oleraceum	2	X X
30	Colchicum autumnalis ...	33	XXXXXXXXXXXXXX X XXXXXXXXXXXXXXXXXX
31	Crepis taraxacifolia	19	XX X XXX X XXXXXXXXXXXX X
32	—— " —— aurea	1	XX
33	—— " —— paludosa	2	X
34	Cynosurus cristatus	26	XXXXXX XX XXX X XXXXXXXXXXXX
35	Chrysanthem. leucanthem.	44	XX XX XXXXXXX XXX XXXXXXXXXXXXXXX XXXXXX XXXXX XXXX X
36	Dactylis glomerata	42	XXXXX XXXXXXX XXXXXXXX XXXXXXXXX XXXXXXXXXXXXX
37	Deschampsia caespitosa	11	X XXXXXX X XXX
38	Equisetum arvense	6	XXXX XX
39	Festuca pratensis	43	XXXX XXX XXXX XXXXXXXXXXX XXXXXX XXXXXXXXXXXXXX
40	—— " —— ovina	9	X XXXX XXXX
41	Galium asperum (silvestre)	6	X XX X X X XXXX XX
42	Gentiana campestris ...	6	XX
43	—— " —— verna	2	
44	Geranium silvaticum ..	30	XXXXXXXXXXXXXXXXXXXXXXXXXXXXXXX
45	Geum urbanum	3	X XX
46	Hieracium pilosella ..	2	X X XX
47	Hippocrepis comosa ..	2	
48	Hypericum quadrangul.	1	X
49	Juncus lamprocarpus ..	1	X X
50	Knautia arvensis	14	XXXXXXXX XXXXX
51	Lathyrus pratensis	35	XX XXX XX XX XXXXXXX XXXX XXXXXX X XXXXXX X XX
52	Linum catharticum ..	7	XX X XX X
53	Listera ovata	1	
54	Leontodon hispidus	42	XXX XXXX XX XXXXXXX X XXXXXXXXXXXXXXXXXXXXX XXXX
55	Lotus corniculatus	21	X X XX XX XXXX XXXXXX XXXX X
56	Luzula silvatica	2	X X XXX XXXX XXX
57	Medicago lupulina ..	20	XX XXX XXXXX
58	Melandrium silvestre .	13	XX XXXXXXXXXX X
59	Myosotis silvatica ...	12	XXXXX X XX X XX X XXX
60	Picea excelsa	3	XX X XXX X X X
61	Plantago lanceolata ..	17	XXXXXXXX XXXXXXXXXXXXXXXXXXX
62	—— " —— media	20	X X XXXX X X
63	Poa pratensis	26	X XXX XX X XXXXXXXX X X XXX X X XX
64	Polygala vulgaris	5	X XXX
65	Polygonum aviculare .	3	
66	—— " —— bistorta	23	XXXX XXXXXXXXXXXXXXXXX XX
67	Potentilla silvestris (Tormentilla)	5	XX X XX
68	Primula elatior	11	XXX XX X X X X X XX
69	Phyteuma orbiculaire .	3	X
70	—— " —— spicatum ..	15	X XXX X XXXX XX X X XX
71	Ranunculus acer	35	XX XXXX X XXXX XX X XXXXX XXXXXXXXXXXXXXX

NOUVELLES RECHERCHES SUR LA DISTRIBUTION FLORALE[1]

PAR

Paul JACCARD,
Professeur à l'Ecole polytechnique fédérale.

Pl. X—XX.

Les recherches que j'ai poursuivies pendant plusieurs années en vue de déterminer la distribution locale de la flore dans la zone alpine, m'ont permis d'établir qu'il existe entre la composition spécifique d'une prairie alpine et le nombre des espèces qui la constituent, ainsi qu'entre le degré de fréquence relatif de ces espèces, leur répartition suivant les grandes subdivisions du règne végétal et suivant les genres auxquels elles appartiennent, certaines relations constantes, ayant pour la région considérée le caractère de lois[2].

Afin de vérifier le degré de généralité des résultats obtenus pour la zone alpine, j'ai étendu mes recherches à la distribution de la flore dans la prairie subalpine.

Le territoire étudié se trouve dans la partie supérieure de la vallée de la Grande-Eau (commune d'Ormont-dessus, Alpes vaudoises), à l'altitude de 1200 mètres environ. La prairie qui le recouvre repose en entier sur un terrain d'alluvion, qui constitue un substratum très uniforme.

[1] Travail présenté au Congrès international de géographie, à Genève, juillet 1908.

[2] Voir entr'autres : *Lois de Distribution florale dans la zone alpine* (Bullet. soc. vaud. sc. nat. Vol. XXXVIII, 1902) et *Gesetze der Pflanzenverteilung in der alpinen Region*. Flora Bd. 90. 1902.

Au lieu d'envisager comme dans mes précédents travaux des localités de plus de 1 ha. de superficie, j'ai fait cette fois-ci le relevé floristique de 52 m² groupés par 4 à 8 carrés contigus de 1 m. de côté en 9 localités distantes l'une de l'autre de 1 km. environ.

Voici la liste de ces 9 localités avec l'indication de leurs caractères particuliers :

A. *Prairies en pente.*

I. Pente uniforme, inclinée d'environ 25 à 30°. Exubérance moyenne. Hauteur moyenne des tiges fleuries : 50 centimètres. Exposition sud. Altitude : 1190 m.

Relevé floristique de 8 m² contigus et disposés de la façon ci-contre (fig. 1). Nombre total des espèces : 53. Coefficient moyen de communauté = 47 %.
(moyenne de 28 rapports)
extrêmes : 35 % et 61 %

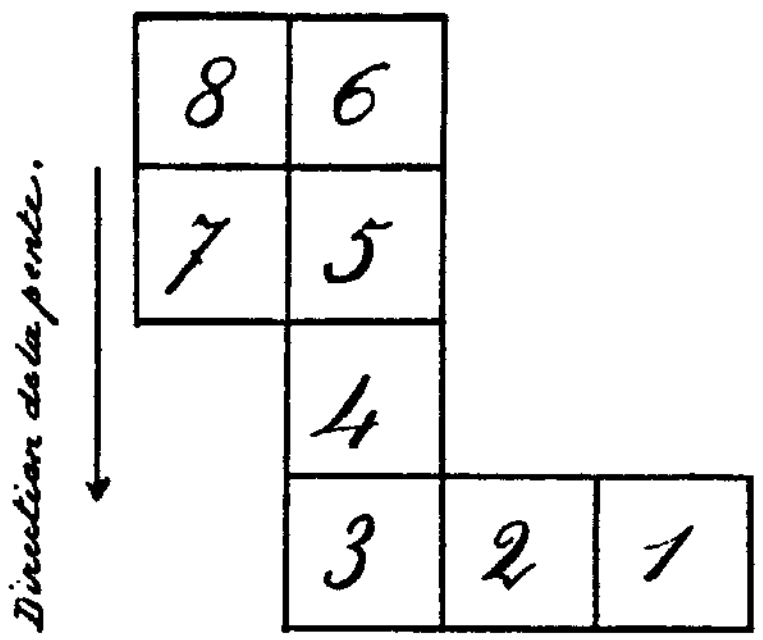

Fig. 1.

II. Pente uniforme; inclinaison approximative : 25°. Prairie exubérante. Hauteur moyenne des tiges fleuries : 80 cm. Exposition sud. Altitude : 1200 m. Relevé floristique de 6 m² groupés comme ci-contre (fig. 2). Nombre total des espèces : 44. Coefficient de com. 54 %.
(moyenne de 15 rapports)
extrêmes : 35 % et 64 %

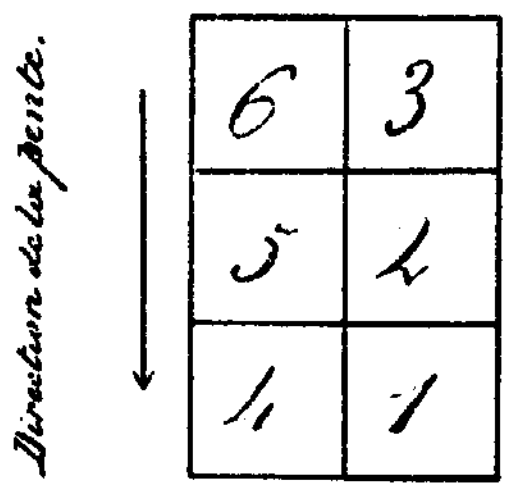

Fig. 2.

III. Pente variant de bas en haut de 35° à 10° environ. Exubérance variant, de bas en haut, de faible à moyenne. Exposition nord. Altitude : 1050 m. Relevé floristique de

8 m², contigus dans le sens de la pente (fig. 3). Nombre total des espèces : 45. Coeff. de comm. 57 %.
(moyenne de 28 rapports)
extrêmes : 34 % et 70 %

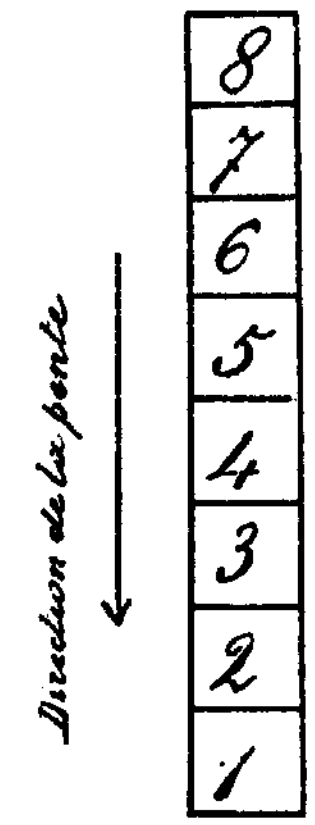

Fig. 3.

IV. Pente uniforme de 10° environ. Prairie exubérante ayant reçu de l'engrais; hauteur moyenne des tiges fleuries : 80 cm. Exposition ouest. Altitude 1160 m. environ. Relevé floristique de 6 m² disposés comme suit, parallèlement à la pente (fig. 4). Nombre total des espèces : 39. Coeff. de comm. 59 %.
extrêmes 39 % et 76 %

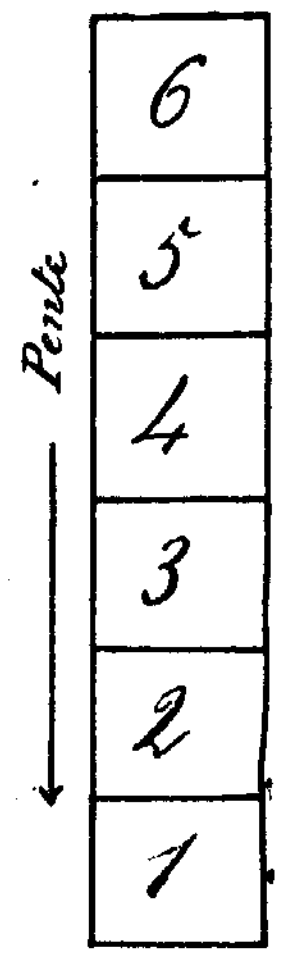

Fig. 4.

V. Même prairie que N° IV. Relevé floristique de 6 m² disposés comme suit, perpendiculairement à la pente (fig. 5).
Total des espèces : 29. Coeff. de comm. 73 %.
extrêmes 63 % et 91 %

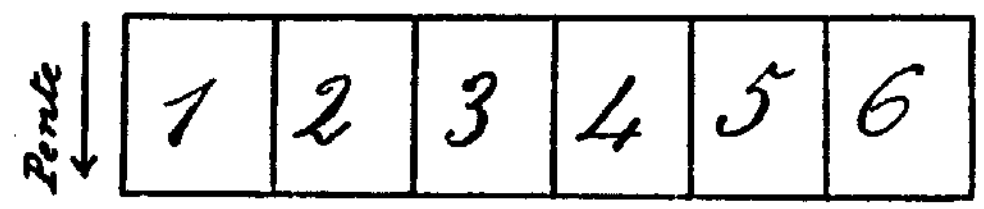

Fig. 5.

B. *Prairies plates.*

VI. Prairie maigre située à 100 m. de la lisière d'une forêt. Relevé floristique de 4 m² disposés comme suit (fig. 6) : Nombre des espèces : 38. Coeff. moyen de comm. 66 %.
extrêmes 51 % et 74 %

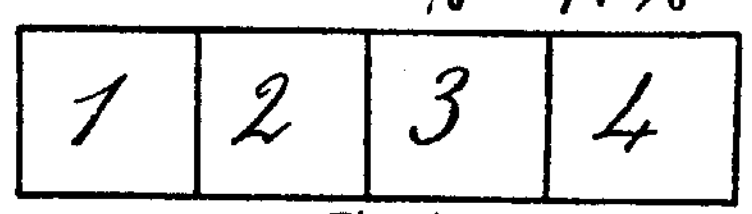

Fig. 6.

VII. Prairie d'apparence très homogène ; exubérance moyenne. Hauteur moyenne des tiges fleuries : 50 cm. Relevé floristique de 5 m² groupés comme suit (fig. 7): Total des espèces : 26. Coeff. moyen de comm. 76 %.

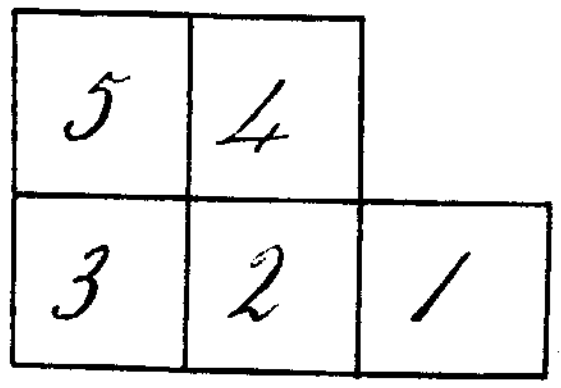

Fig. 7.

extrêmes : 71 % et 83 %

VIII. Prairie d'exubérance moyenne. Hauteur des tiges fleuries : 50-60 cm. Relevé floristique de 7 m² disposés comme suit (fig. 8) :

Total des espèces : 36. Coeff. moyen de comm. 63 %. Extrêmes 50 % et 78 %

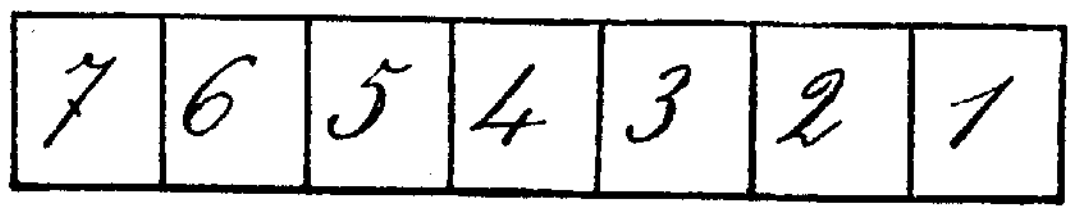

Fig. 8.

IX. Prairie maigre située à 30 m. de distance de la précédente. Relevé de 2 m² contigus. Total des espèces : 25. Coeff. de comm. 80 %.

Voir au tableau nº 12 les documents relatifs aux coeff. de comm.

Diversité florale élémentaire.

La distribution des 92 espèces notées sur les localités I à IX, ainsi que sur chacun des 52 mètres carrés explorés est transcrite sur le tableau ci-joint (Pl. X.) lequel indique également le nombre des espèces recueillies sur chaque m² et celui noté sur chaque localité (groupe de 2 à 8 m²). D'après ces indications, il est facile de calculer le coefficient de communauté florale, c'est-à-dire le rapport du nombre des *espèces distinctes* croissant sur 2 m² ou sur 2 localités comparées à celui du nombre des *espèces communes* croissant à la fois sur les deux termes de com-

Schéma N° 1,

montrant la distribution des espèces spéciales et des espèces communes aux carrés 1 et 2 de la localité I.
(Prairie des Ormonts).

Carré 1.

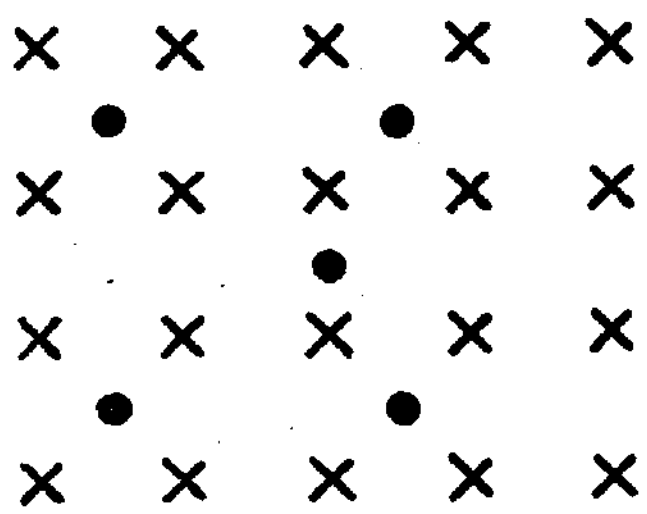

Carré 2.

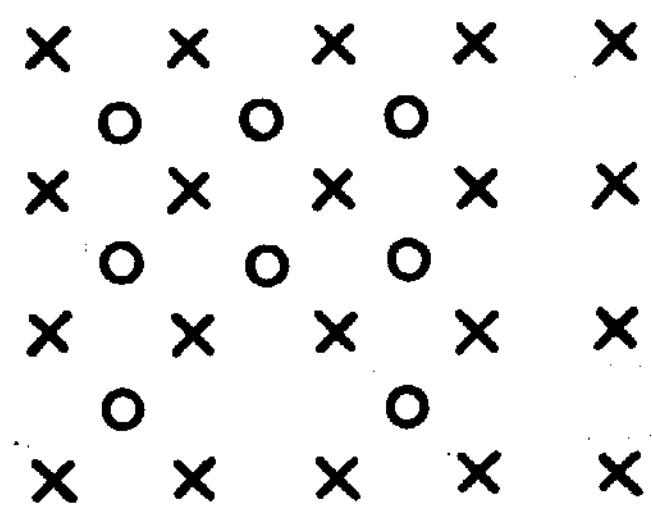

Schéma de distribution N° 2.

Carré 1 = 25 espèces.

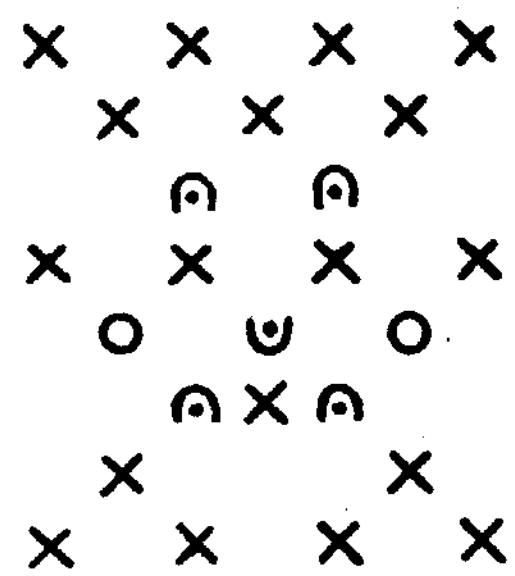

Carré 2 = 29 espèces.

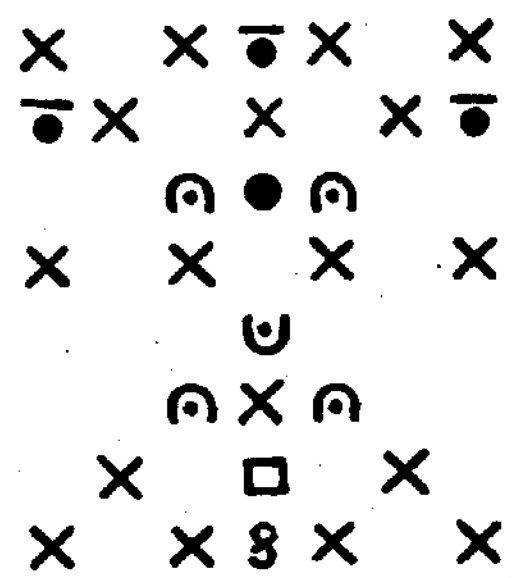

Carré 3 = 26 espèces.

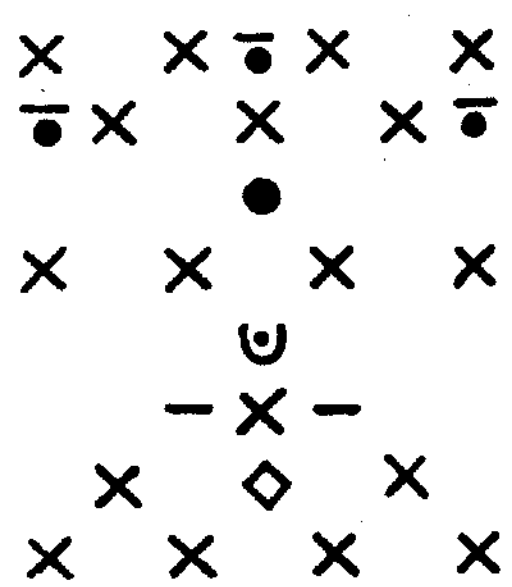

Carré 4 = 28 espèces.

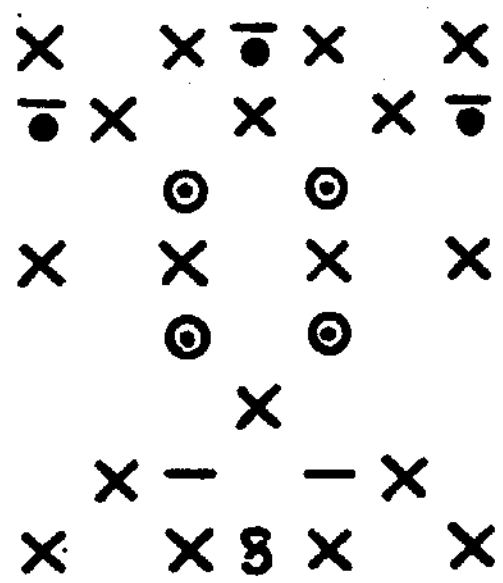

Coefficient de communauté pour les carrés 1 et 2 de la localité I (Ormonts).

× = espèces <u>communes</u> aux carrés 1 et 2 : 20 espèces.
● = „ <u>spéciales</u> au carré 1 : 5 „
○ = „ ——„—— „ „ 2 : 8 „
×+○+● = nombre total des espèces croissant sur 1 et 2 : 33 „

Sur 33 espèces <u>distinctes</u> il y a 20 esp. <u>communes</u>, soit 60 %.

Schéma N° 1.

Distribution des 38 espèces croissant sur les carrés 1 à 4.
Schéma N° 2.

Signes employés :

Espèces communes aux carrés 1 à 4 = × (13 espèces).
„ spéciales au carré 1 = ○ (2 „)
„ „ „ „ 2 = □ (1 „)
„ „ „ „ 3 = ◇ (1 „)
„ „ „ „ 4 = ⊙ (4 „)
Espèces communes aux carrés 1 et 2 (seulement) = ⌒ (4 „)
„ „ „ „ 1, 2 et 3 = ◡ (1 „)
„ „ „ „ 2 et 3 (seulement) = ● (1 „)
„ „ „ „ 2 et 4 = 8 (1 „)
„ „ „ „ 2, 3 et 4 = ‾● (3 „)
„ „ „ „ 3 et 4 (seulement) = — (2 „)

Il n'y a pas d'espèces communes à 1 et 2 ou 1 et 4 seulement, ni à 1, 2 et 4 ou 1, 3 et 4.

N° 3. Schéma de distribution pour la localité IV.

(Prairie des Ormonts).

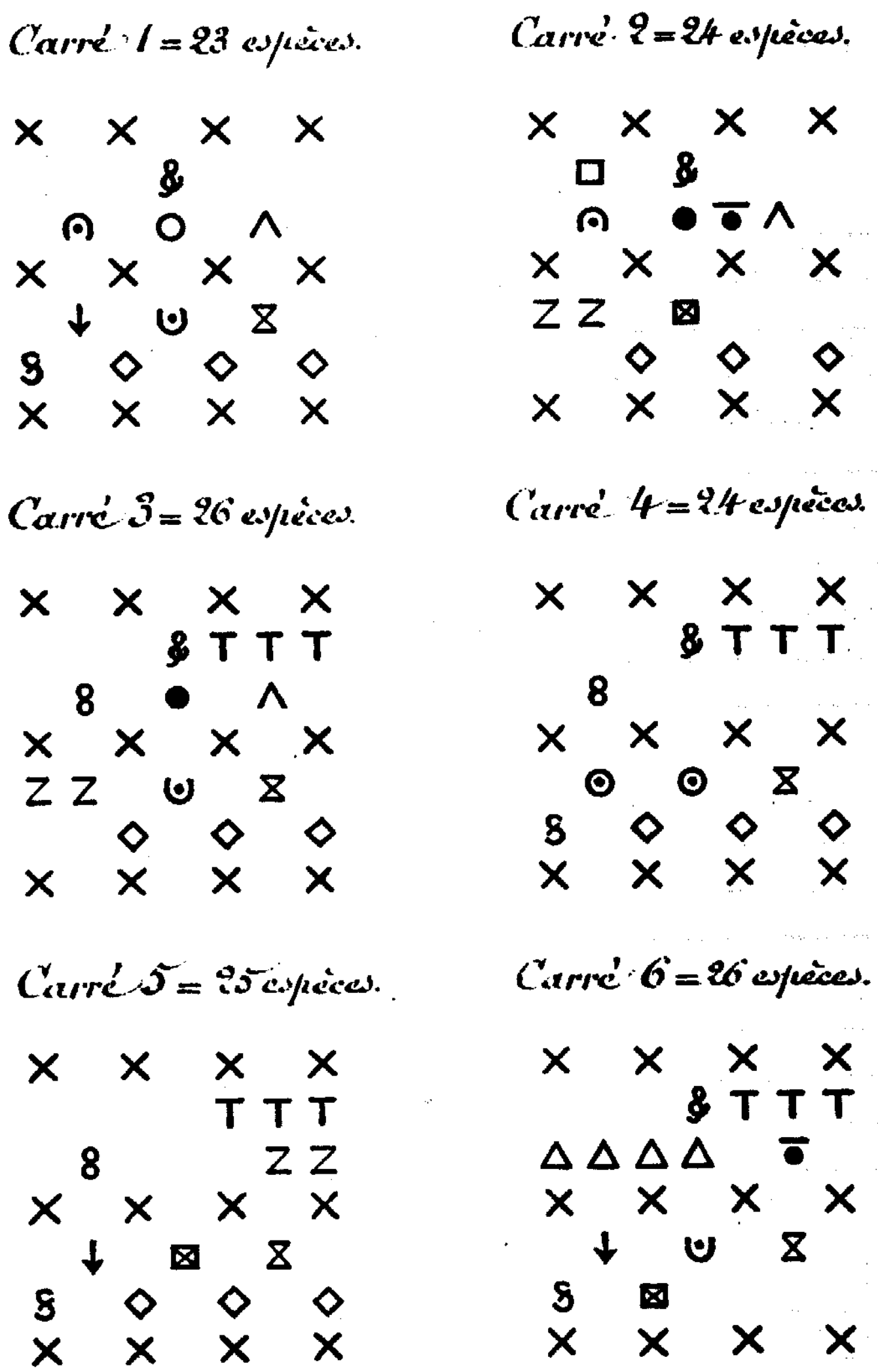

Signes conventionnels employés:

Espèces communes aux carrés	1 à 6	= ✕	(12 espèces)
„ spéciales au carré	1	= ○	(1 „)
„ „ „ „	2	= □	(1 „)
„ „ „ „	3	=	(0 „)
„ „ „ „	4	= ⊙	(2 „)
„ „ „ „	5	=	(0 „)
„ „ „ „	6	= △	(4 „)

Total des espèces distinctes: 39, représentées par 19 signes différents.

Espèces communes aux carrés	1 et 2 seulement	◠	(1 espèce).
„ „ „ „	1, 2 et 3 „	∧	(1 „)
„ „ „ „	1, 3 et 6 „	◡	(1 „)
„ „ „ „	1, 5 et 6 „	↓	(1 „)
„ „ „ „	1, 4, 5 et 6 „	§	(1 „)
„ „ „ „	1, 3, 4, 5 et 6 „	⧖	(1 „)
„ „ „ „	1, 2, 3, 4 et 5 „	◇	(3 „)
„ „ „ „	1, 2, 3, 4 et 6 „	&	(1 „)
„ „ „ „	2 et 3 „	●	(1 „)
„ „ „ „	2 et 6 „	●̄	(1 „)
„ „ „ „	2, 3 et 5 „	Z	(2 „)
„ „ „ „	2, 5 et 6 „	⊠	(1 „)
„ „ „ „	3, 4 et 5 „	8	(1 „)
„ „ „ „	3, 4, 5 et 6 „	T	(3 „)

paraison. Le schéma N° 1 traduit graphiquement ce qu'exprime en chiffres le coefficient de communauté pour les carrés 1 et 2 de la localité I (voir Pl. XI).

Pour la localité N° VI du tableau on obtient les coefficients de communauté suivants :

Esp. distinctes sur 1 et 2 = 31. Esp. communes à 1 et 2 = 23.

$$\text{Coeff. de comm.} = \frac{23 \times 100}{31} = 74{,}2\ \%.$$

Entre les carrés	1 et 2	le coeff.	de comm.	= 74 %.
»	»	1 et 3	»	»	= 59 %.
»	»	1 et 4	»	»	= 51 %.
»	»	2 et 3	»	»	= 72 %.
»	»	2 et 4	»	»	= 63 %.
»	»	3 et 4	»	»	= 74 %.

Coefficient moyen de communauté = 66 % (moyenne des 6 rapports). Nombre des espèces sur chacun des carrés 1-4 = 25, 29, 26 et 28. Nombre moyen des espèces par m² (pour les carrés 1-4) = 27. Nombre total des espèces distinctes sur les carrés 1-4 = 38.

Dans le schéma N° 2 (Pl. XI), j'ai essayé de représenter par des signes conventionnels la distribution des 38 espèces croissant sur les carrés 1-4 mentionnés ci-dessus.

Le schéma en question nous montre que ces espèces y sont distribuées de 11 manières différentes.

Les 39 espèces de la localité IV comprenant 6 mètres carrés sont distribuées de 19 manières différentes ; le nombre des espèces communes aux 6 m² est de 12. (Schéma N° 3, Pl. XII).

Pour la localité I comprenant 8 mètres carrés l'établissement d'un schéma de distribution des 53 espèces qui s'y trouvent devient déjà difficile, car il nécessite l'emploi de 40 signes représentant 40 groupements différents. Le

nombre des espèces communes aux 8 m² n'est plus dans ce cas que de 4.

Plus le nombre des termes de comparaison augmente, plus diminue le nombre des espèces communes à chacun d'eux. A partir de 15 m² déjà, répartis sur une superficie de 1 ha. par ex., on trouve rarement deux espèces ayant exactement la même distribution[1], et, comme l'indique le tableau I, aucune espèce ne se rencontre sur l'ensemble des 52 m² considérés. L'espèce la plus fréquente, *Trifolium pratense*, n'a été notée que sur 48 m² et 5 autres espèces seulement sur plus de 40 m².

La distribution des espèces dans la prairie subalpine des Ormonts, telle qu'elle ressort du tableau Nº I, ci-joint, confirme absolument ce que l'étude de la prairie alpine m'avait déjà révélé, à savoir *la grande diversité de composition florale des différentes localités d'une même formation.*

Cette diversité se manifeste non seulement dans la composition florale de territoires très étendus, mais encore dans celle de localités restreintes, comprenant quelques mètres carrés de superficie seulement. On peut donc la considérer comme un caractère essentiel et primordial du type de formation que nous envisageons, et parler d'une *diversité élémentaire de distribution florale* puisque, quelque soit l'élément de surface que l'on envisage, are, Dm² ou m², *deux portions, même contiguës d'une prairie naturelle, n'ont jamais exactement la même composition florale spécifique.*

Le plus haut degré de communauté spécifique que j'aie observé jusqu'ici s'élève à 91 % et concerne deux mètres carrés de la localité V, prairie semi-naturelle où l'influence

[1] Ceci n'est absolument vrai que pour la prairie naturelle; dès que par la culture, par l'engrais en particulier, on modifie les conditions de concurrence au profit d'un petit nombre d'espèces, la diversité de composition florale diminue.

d'une fumure printanière apparaissait soit dans la composition florale, soit dans l'exubérance de la végétation. Il n'est pas rare que dans une prairie complètement naturelle, le coefficient de communauté entre 2 mètres carrés situés à 100 mètres de distance seulement descende à 20 %.

TABLEAU Nº 2.

Prairie marécageuse à Deschampsia caespitosa et Carex paniculata.
(Ormont-dessus) 1120 m.

	m² contigus				5me m² (à 20 m. des précédents)
	1er m²	2e m²	3e m²	4e m²	
Deschampsia caespitosa	×	×	×	×	×
Carex paniculata	×	×	×	×	×
» vesicaria	×	×	×	×	
» pallescens	×				
Poa palustris	×	×	×		×
Scirpus silvaticus	×		×	×	×
Caltha palustris	×	×	×	×	×
Myosotis palustris	×				
Melandrium silvestre	×				
Plantago lanceolata	×				
Spirea ulmaria	×	×	×	×	×
Equisetum arvense		×			
Dactylis glomerata		×			
Carex panicea		×			
Allium angulosum			×	×	
Carex stricta			×		
Juncus conglomeratus			×		
Festuca arundinacea				×	×
Sanguisorba minor				×	
Galium palustre					×
Briza media					×
Lychnis (Coronaria flos cuculi)					×
Ranunculus acer					×
Trifolium pratense					×
Geum rivale					×
Lathyrus pratense					×
Valeriana officinalis					×
	11 esp.	9 esp.	10 esp.	9 esp.	15 esp.

Coefficient de communauté entre les 4 m² contigus = 26 %.

Coefficient moyen de communauté entre les carrés 1, 2, 3 et 4 (pris 2 à 2) = 46 %.

Les prairies humides ou marécageuses elles-mêmes sont loin d'avoir l'uniformité de composition qu'elles paraissent posséder lorsqu'on les observe d'une façon superficielle. Les laiches ou les graminées dominantes qui semblent à elles seules les couvrir entièrement sont accompagnées de nombreuses espèces, représentées il est vrai par un nombre relativement faible d'individus, mais qui suffisent à faire naître une diversité de composition florale qu'on ne soupçonne guère à priori dans de pareilles formations.

C'est ce qui ressort très nettement du tableau N° 2 ci-joint montrant la composition et la distribution florales sur 5 m² d'une prairie marécageuse complétement envahie par *Deschampsia caespitosa* et *Carex paniculata*.

* * *

Les 92 espèces observées sur notre prairie des Ormonts sont toutes des espèces communes, répandues dans l'ensemble de la zone subalpine des Alpes vaudoises et qui pourraient à la rigueur se rencontrer sur l'un quelconque des 52 m² explorés. Il suffit donc de causes inappréciables pour déterminer sur un point donné de la prairie l'admission ou l'exclusion de telle ou telle espèce et pour donner naissance à la variation infinie qui, d'un point à un autre, se manifeste dans le groupement des espèces d'une seule et même formation.

La diversité observée est cependant loin de correspondre au maximum mathématiquement possible. Sur 1 km² de la prairie subalpine des Ormonts, j'ai relevé approximativement 150 espèces différentes, distribuées *en moyenne* à raison de 25 par m². Les combinaisons possibles de ces 150 espèces groupées 20 à 30 par m² (ou même 18 à 38, extrêmes probables pour la prairie fourragère de la contrée), atteignent un nombre incalculable, correspondant très vraisemblablement, ainsi que la diversité constatée

nous permet de l'admettre, à celui des mètres carrés couverts par la prairie en question, soit *1 million*.

Cette diversité de composition florale qui paraît extraordinaire au premier abord, s'explique cependant lorsqu'on la rattache aux variations possibles des conditions écologiques.

Lorsqu'on envisage les divers facteurs écologiques (lumière, chaleur, humidité, aération souterraine, composition chimique et physique du sol d'où résulte la proportion relative des éléments minéraux utilisables par les plantes), facteurs qui, par leur combinaison, assurent ou permettent le développement des 150 espèces sus-mentionnées, et qu'on évalue toutes les variations d'intensité (variations quantitatives), que chacun d'eux est susceptible d'éprouver entre ses limites extrêmes, on obtient un nombre infini de combinaisons différentes que l'on peut supposer capables d'agir vis-à-vis des espèces concurrentes, soit comme causes favorisantes, soit comme entraves, en créant des inégalités propres à provoquer la diversité élémentaire de composition florale observée.

De pareilles variations qualitatives et quantitatives des conditions écologiques existent-elles réellement entre les diverses portions d'une seule et même station de peu d'étendue et d'apparence uniforme?

Cela n'est pas douteux si l'on songe que les plantes elles-mêmes réagissent les unes sur les autres (ombre portée et eau retenue par les feuilles, modifications physiques et chimiques du sol, neutralisation, (Vergiftung, Entgiftung) provoquées par les racines des plantes et par divers animaux qui les accompagnent) et que la moindre déclivité du sol modifie la quantité de chaleur et de lumière qu'il reçoit, ainsi que son degré d'humidité.

Il est d'ailleurs probable qu'à côté des variations de la composition florale qu'on observe à un moment donné entre les diverses portions d'une prairie, il s'en produise,

au cours des années, d'autres provoquées simplement par les modifications que la végétation fait naître à la longue dans le sol où elle se développe.

On sait que de pareilles transformations se manifestent tout naturellement dans la composition de certaines forêts. Il est probable que le remplacement insensible du chêne par le hêtre et par ses espèces compagnes dans nombre de massifs où ces deux essences étaient autrefois mélangées également ou mélangées en proportion inverse, résulte, en partie du moins, de l'influence défavorable qu'exerce à la longue le hêtre sur les conditions de croissance de son associé.

* * *

Enfin, rappelons encore une cause de variations que nous avons déjà signalée dans un mémoire précédent[1], à savoir l'influence considérable que l'étendue exerce, toutes autres conditions appréciables étant égales, sur la richesse florale.

A raison de 25 par m², les 150 espèces dont nous parlions tout à l'heure pourraient trouver place sur 6 m², mais, bien que la plupart d'entre elles soient des espèces tout à fait communes, il est nécessaire de parcourir au moins 1 km² de notre prairie pour les rencontrer toutes. On en peut conclure que les variations secondaires des conditions écologiques dans une station déterminée *se multiplient et se diversifient en proportion de l'étendue que l'on envisage*, alors même que les caractères généraux de la station ne changent pas d'une manière appréciable.

Afin de mieux préciser ce que nous entendons par variations secondaires, nous distinguerons dans les conditions écologiques : 1° les *agents biologiques*, c'est-à-dire ceux dont la présence est indispensable à la manifestation

[1] Voir à ce sujet : *Lois de distribution de la flore dans la zone alpine*, loc. cit. p. 89 et suiv.

de la vie végétale ; tels sont la lumière, la chaleur, l'humidité, ainsi que les éléments minéraux nécessaires à l'alimentation des plantes, et 2° les *facteurs physiques qui modifient les premiers quantitativement*. Les facteurs physiques sont d'une part *géographiques* et *topographiques* et déterminent le climat, d'autre part *édaphiques*, c'est-à-dire résultant de la nature du sol.

Sous l'influence des facteurs physiques, les agents biologiques sont soumis à des variations dont l'amplitude est fort inégale.

Les variations les plus fortes résultent des facteurs géographiques, ce sont des *variations générales* qui retentissent, directement et avant tout, sur l'intensité lumineuse et calorifique et se traduisent par la physionomie et la composition des grandes *régions de végétation* (végétation thermophyte, hygrophyte, mésophyte, etc.). Dans chaque région de végétation, des variations moins accentuées ou *variations régionales* résultant surtout de la combinaison des facteurs géographiques et topographiques, déterminent la distribution des *types de végétation* ou *formations* (forêts, prairies, garigues, etc.).

Les *variations locales*, résultant surtout de l'inégale combinaison des facteurs topographiques et édaphiques, déterminent à leur tour dans chaque formation la composition et la distribution des *associations végétales*.

Enfin, grâce aux *variations élémentaires*, c'est-à-dire aux variations secondaires qui se manifestent sur chaque point ou élément de surface d'une station déterminée, les associations végétales qui la recouvrent sont constamment différentes d'un point à un autre ; il en résulte *la diversité élémentaire de composition florale* que nous avons signalée.

* * *

Fréquence relative des espèces associées.

Si dans une station donnée le nombre et la distribution des espèces dépend en définitive, des multiples variations élémentaires des conditions écologiques, variations qui paraissent absolument désordonnées, comment expliquer la régularité relative avec laquelle les espèces y sont distribuées suivant leur degré de fréquence?

Lorsqu'on groupe les 92 espèces du tableau N° I en espèces rares, communes et très communes, suivant qu'elles apparaissent dans 1 à 16, 17 à 32 ou 33 à 48, des 52 m² explorés[1] on obtient, pour ces trois catégories, des chiffres qui, graphiquement, se traduisent par une ligne ascendante (voir Graphique n° 1, Pl. XIII). Ce graphique ressemble d'une manière frappante à ceux publiés dans mes précédents mémoires[2], concernant la proportion des espèces de la prairie alpine, suivant leur degré de fréquence. Cette concordance est d'autant plus curieuse que le nombre des espèces envisagé ici, 92, est très faible.

La loi de distribution des espèces par ordre de fréquence que nous avions établie pour la prairie alpine se trouve donc confirmée. *Ce sont les espèces rares* (c'est-à-dire distribuée sur moins de $^1/_3$ des 52 m²), *qui sont les plus nombreuses et les espèces très communes* (apparaissant sur plus des $^2/_3$ des 52 m²), *qui sont les moins nombreuses.*

Il ne s'agit cependant pas là d'une loi élémentaire aussi générale que celle qui concerne la distribution florale, car sur des surfaces restreintes telles que celles des diverses localités de notre tableau, lesquelles ne comprennent que 4 à 8 m², on constate au contraire entre les espèces rares

[1] Aucune espèce, comme nous l'avons dit plus haut, n'apparait sur plus de 48 m².

[2] *Lois et distributions, etc.* loc. cit. p. 113 et suivantes, et *Revue générale des sciences*, Paris 1907, p. 965.

et les espèces communes pour ces localités, une relation inverse.

Prenons comme exemple les localités I et III, comprenant chacune 8 m² de surface, la première avec 53, la troisième avec 45 espèces distinctes.

Les 53 espèces de la localité I, de même que les 45 espèces de la localité III peuvent apparaître chacune dans 1, 2, 3, 4.... 8 m² différents. Elles peuvent donc avoir dans chacune de ces deux localités 8 degrés différents de fréquence locale.

Le degré de fréquence des espèces variant nécessairement suivant l'étendue de la surface florale envisagée, il importe de distinguer la *fréquence locale* d'une espèce, c'est-à-dire sa fréquence relative à une localité, de sa *fréquence générale*, c'est-à-dire relative à l'ensemble des 52 mètres carrés de notre prairie. Il n'y a d'ailleurs, comme nous le verrons plus loin, aucune relation constante entre la fréquence locale et la fréquence générale d'une même espèce. En déterminant pour *chaque m²* d'une localité les nombres correspondant aux divers degrés de fréquence locale des espèces qui s'y trouvent, on obtient ce que nous appellerons la *fréquence florale élémentaire.*

TABLEAUX N°s 3 et 4

montrant la distribution de 53 et 45 espèces dans les 8 m² des localités I et III.

m² 1 à 8	I Degrés de fréquence 1	2	3	4	5	6	7	8	Nombre des esp. par m²	m² 1 à 8	III Degrés de fréquence 1	2	3	4	5	6	7	8	Nombre des esp. par m²
1	2	2	—	3	5	7	5	4	28	1	2	2	3	3	2	1	7	6	26
2	2	—	—	1	6	7	5	4	25	2	—	1	2	4	2	3	10	6	28
3	—	1	—	3	1	9	5	4	23	3	1	1	—	5	2	5	8	6	28
4	—	—	—	2	4	8	4	4	22	4	—	1	—	4	—	5	10	6	26
5	1	1	3	5	3	10	3	4	30	5	—	1	1	3	—	5	9	6	25
6	3	1	2	8	5	7	5	4	35	6	—	—	1	3	—	4	11	6	25
7	1	2	3	5	3	8	3	4	29	7	2	1	1	2	2	3	11	6	28
8	2	1	4	5	3	10	5	4	34	8	2	1	1	4	2	4	11	6	31

Les tableaux Nos 3 et 4 ainsi que les graphiques Nos 2 et 3, (Pl. XIV) expriment cette fréquence florale élémentaire pour chaque mètre carré des localités I et III. Ils nous montrent par exemple (Tabl. 3) que les 28 espèces croissant sur le premier mètre carré de la localité I comprennent deux espèces qui n'apparaissent que sur un seul des 8 m^2 de cette localité, deux qui figurent sur 2 m^2, aucune qui se rencontre sur 3 m^2, enfin 3, 5, 7, 5 et 4 qui croissent respectivement sur 4, 5, 6, 7 ou 8 m^2 de cette localité. Les 34 espèces du huitième mètre carré de la même localité se répartissent en 2 espèces à Fr. loc.[1] 1 ; 1 à Fr. loc. 2 ; 4 à Fr. loc. 3; 5 à 4; 3 à 4; 10 à 6; 5 à 7 et 4 à Fr. loc. 8.

Les tracés 1 et 8 du graphique N° 2 (Pl. XIV) permettent de saisir d'un coup d'œil les analogies et les différences qui d'un mètre carré à l'autre se manifestent entre ces divers nombres ; nous désignons ces tracés sous le nom de *courbe de fréquence florale élémentaire*, ou plus simplement *courbe de fréquence élémentaire*, tandis que par le tracé M, correspondant aux *valeurs moyennes* des diverses fréquences élémentaires, nous exprimons la *fréquence locale moyenne*, suivant laquelle les espèces se répartissent, *en moyenne*, sur toute l'étendue d'une localité. Nous appellerons ces tracés moyens *courbes de fréquence locale*.

Mieux encore que les chiffres des tableaux 3 et 4, les divers tracés des graphiques 2 et 3 font ressortir à la fois les analogies et les différences qui, dans une même localité, se manifestent d'un mètre carré à l'autre dans la distribution des espèces suivant leur fréquence locale. Ils nous montrent tout d'abord que dans les localités I et III, le plus grand nombre des espèces correspond non pas au maximum de fréquence locale, c'est-à-dire à la fréquence 8, mais bien aux fréquences 7 (Graph. 3) et 6 (Graph. 2),

[1] Abrév. pour degré de fréquence locale.
Voir à la fin du mémoire, dans le tableau 11, les chiffres concernant les autres localités.

tandis que dans les localités plus restreintes, II, IV, V et VIII, comprenant respectivement 6 et 7 m^2, le plus grand nombre des espèces correspond au maximum de fréquence locale possible. (Voir Graphiques Nos 4 à 7, Pl. XIV.)

Les graphiques Nos 2 à 7 nous montrent, à côté de quelques divergences, des analogies évidentes entre les courbes de fréquence élémentaire d'une même localité.

Dans le graphique N° 7, les ressemblances les plus fortes s'observent entre les tracés 1, 2 et 4, puis entre 3 et 5, tandis que les tracés 6 et 7 diffèrent assez sensiblement des précédents.

Les différences sont encore plus accentuées entre les diverses courbes de fréquence élémentaire des graphiques Nos 2 et 3.

Néanmoins, toutes ces courbes présentent une certaine analogie provenant surtout du fait que presque toutes possèdent deux sommets principaux d'inégale hauteur, mais tombant dans chaque localité assez régulièrement sur les mêmes degrés de fréquence : 4 et 6 pour la localité I ; 4 et 7 pour la localité III ; 3 et 6 pour les localités II, IV et V ; 2 et 7 pour la localité VIII. Ces deux sommets se maintiennent dans les courbes de fréquence locale moyenne (courbes M) et leur confèrent un cachet caractéristique qui varie d'une localité à l'autre.

En exprimant la fréquence locale moyenne non plus suivant 6 ou 8 degrés, mais suivant 3 ou 4 degrés correspondant aux désignations usuelles rares, assez communes, communes et très communes, on obtient pour les localités I à VIII des tracés analogues à celui du Graphique N° 1 relatif à la fréquence générale. Toutefois le sommet de ces courbes M 3 ou M 4 (voir les Graphiques Nos 8 et 9, Pl. XV.) correspond non pas aux espèces rares, mais bien aux espèces communes, c'est-à-dire à fréquence locale maximum[1]. Sur

[1] L'exception présentée par la courbe M de la localité *I* disparaît lorsqu'on n'envisage que 3 degrés de fréquence.

une surface très restreinte comprenant seulement quelques mètres carrés contigus ; ce sont donc, contrairement à ce qu'on observe dans des territoires plus étendus, les espèces communes, c'est-à-dire à fréquence supérieure aux $^2/_3$ du maximum possible qui dominent.

Il n'en pourrait d'ailleurs pas être autrement, puisque dans la localité I comprenant 8 m² et 52 espèces (chaque mètre carré ayant en moyenne 25 espèces) le nombre des espèces dont la fréquence locale est égale à 1 ne saurait dépasser au maximum $\frac{52}{8}$ soit 6 à 7 sur chaque mètre carré[1], tandis que le nombre des espèces à fréquence 8 peut théoriquement atteindre 25 dans le cas où toutes les espèces d'un mètre carré se répéteraient sur tous les autres (ce qui ne se réalise d'ailleurs jamais).

Par contre, dans une prairie comprenant 50 m² avec 100 espèces réparties à raison de 20 à 22 par mètre carré, le nombre des espèces « rares » à fréquence locale = 1 peut être théoriquement de 100-20 soit 80 au maximum, tandis que le nombre des espèces à fréquence maximum ne peut dépasser 20, soit le nombre minimum des espèces croissant sur un mètre carré.

* * *

Les analogies que nous avons relevées entre les courbes de fréquence locale des localités I-VIII ne proviennent pas, comme on pourrait le supposer, de la répétition des mêmes espèces possédant dans ces diverses localités le même degré de fréquence. Il est facile de s'en convaincre en dressant la liste des espèces qui dans les localités I à V et VIII ont le même degré de fréquence locale soit la fréquence 6. (Voir tableau N° 5.)

[1] Cette proportion est d'ailleurs loin d'être atteinte, elle ne pourrait l'être que si toutes les espèces étaient « rares » ce qui est par définition impossible, et si chaque mètre carré n'avait pas plus de 6 ou 7 espèces.

TABLEAU N° 5.

Espèces présentant le degré de fréquence[1] 6 dans les localités I à V et VIII

Nos [2]	I	Nos	II
8	Anthoxanthum odoratum.	6	Alectorolophus hirsutus
5	Alchemilla pratensis	19	Brunella vulgaris
14	Bellis perennis	25	Carum carvi
16	Briza media	30	Colchicum autumnale
35	Chrysanthemum leucanthem.	36	Dactylis glomerata ●
36	Dactylis glomerata	44	Geranium silvaticum
38	Equisetum arvense	50	Knautia arvensis
63	Poa pratensis	76	Sanguisorba minor
68	Primula elatior	77	Silene inflata
71	Ranunculus acer	85	Trisetum flavescens
78	Taraxacum officinalis.		
	11 espèces		10 espèces

Nos	III	Nos	IV
54	Leontodon hispidus	10	Anthriscus silvestris
70	Phyteuma spicatum	5	Alchemilla pratensis ●
74	Ranunculus montanus	35	Chrysanthem. leucanthem. ●
75	Rumex acetosa	34	Cynosurus cristatus
82	Trifolium repens.	44	Geranium silvaticum ●
		58	Melandryum silvestre
		66	Polygonum bistorta
		75	Rumex acetosa ●
		48	Taraxacum officinale ●
		83	Trifolium pratense
		85	Trisetum flavescens ●
		31	Crepis taraxacifolia
	5 espèces		12 espèces

Nos	V	Nos	VIII
10	Anthriscus silvestris ●	35	Chrysanthem. leucanthem. ● ●
5	Alchemilla pratensis ● ●	51	Lathyrus pratensis
21	Campanula rotundifolia	55	Lotus corniculatus.
31	Crepis taraxacifolia	54	Leontodon hispidus ● ●
34	Cynosurus cristatus ●	57	Medicago lupulina.
36	Dactylis glomerata ● ●	76	Sanguisorba minor.
39	Festuca pratensis ●		
44	Geranium silvaticum ● ●		
54	Leontodon hispidus ●		
66	Polygonum bistorta ●		
74	Ranunculus montanus ●		
78	Taraxacum officinale ● ●		
83	Trifolium pratense ●		
85	Trisetum flavescens ● ●		
	14 espèces		6 espèces

● = espèce figurant dans 2 localités.
● ● = » » » 3 »

[1] Obtenu en comptant dans chaque localité le nombre des espèces qui figurent sur 6m².
[2] Numéros d'ordre dans le tableau général N° 1.

TABLEAU N° 6.

Espèces présentant le degré de fréquence 7 dans les localités III et VIII

N°s	III	N°s	VIII
10	Anthriscus silvestris	6	Alectorolophus hirsutus
19	Brunella vulgaris	25	Carum carvi
35	Chrysanthem. leucanthem.	30	Colchicum autumnale
26	Cerastium caespitosum	36	Dactylis glomerata ●
36	Dactylis glomerata	39	Festuca pratensis ●
37	Deschampsia caespitosa	62	Plantago media
39	Festuca pratensis	71	Ranunculus acer
50	Knautia arvensis	78	Taraxacum officinale
66	Polygonum bistorta	83	Trifolium pratense
63	Poa pratensis	85	Trisetum flavescens
89	Vicia craca.	89	Vicia craca ●
	11 espèces.		11 espèces

● = espèces figurant sur les 2 localités.

A l'exception des localités IV et V qui doivent leur forte proportion d'espèces communes à leur grande proximité (elles ne sont distantes que de 20 m. seulement) et à leurs conditions écologiques très semblables, on constate que sur 36 espèces ayant la fréquence 6, 10 seulement sont communes à deux localités et qu'aucune ne figure à la fois sur trois localités.

Le tableau N° 6 nous montre que le degré de fréquence locale 7 est réalisé dans les localités III et VIII par 19 espèces différentes, *dont trois seulement* se rencontrent avec le même degré de fréquence sur ces deux localités. Entre les localités I et II (Tableau N° 5) sur 20 espèces présentant la fréquence 6, une seulement se rencontre avec le même degré de fréquence sur les deux localités.

Sur une étendue même restreinte de la prairie, la fréquence locale de chaque espèce varie donc d'un point à un autre.

Relation entre la fréquence locale et la fréquence générale.

Comme nous l'avons fait remarquer déjà, la fréquence locale des espèces ne dépend pas de leur fréquence générale.

Les espèces qui dans la localité I par exemple présentent la fréquence locale maximum, soit la fréquence 8, sont : *Alectorolophus hirsutus, Colchicum autumnale, Plantago lanceolata* et *Trifolium pratense* dont la fréquence générale est respectivement égale à $^{26}/_{52}$, $^{33}/_{52}$, $^{17}/_{52}$ et $^{48}/_{52}$ (c'est-à-dire que ces espèces se rencontrent dans 26, 33, 17 et 48 m² sur 52).

Dans la localité III, les espèces à fréquence locale 8 sont : *Anthoxanthum odoratum, Alchemilla pratensis, Campanula rhomboïdalis, Geranium silvaticum, Trifolium pratense, Trisetum flavescens,* dont la fréquence générale est respectivement $^{45}/_{52}$, $^{28}/_{52}$, $^{40}/_{52}$, $^{30}/_{52}$, $^{48}/_{52}$ et $^{38}/_{52}$.

Enfin dans la localité II, la fréquence maximum soit 6 est réalisée par les 10 espèces du tableau N° 5 (2me colone), lesquelles ont une fréquence générale égale à 26, 28, 26, 33, 42, 30, 14, 21, 15 et 33.

Les chiffres qui précèdent montrent nettement *que la plupart des espèces qui, dans les localités I à III ont la fréquence locale maximum, ne possèdent pas une fréquence générale correspondante,* puisque les 15 espèces à fréquence locale 8, énumérées ci-dessus, ont 12 degrés différents de fréquence générale compris entre 15 et 48.

Nous en pouvons conclure *que le degré de fréquence d'une espèce dans une prairie donnée est essentiellement variable d'un point à un autre.* Inversément, *chaque portion restreinte* (localité) *d'une prairie est caractérisée par une courbe de fréquence locale*[1] *dans laquelle le sommet*

[1] Représentant la moyenne des courbes de fréquence élémentaire établie pour chaque mètre carré.

correspond au **degré de fréquence optimum** *pour la localité considérée.*

Pour la localité I de notre tableau, ce degré de fréquence optimum est 6, c'est-à-dire que le nombre des espèces qui, dans cette localité, apparaissent sur 6 m^2 est supérieur à celui des espèces apparaissant sur 1, 2, 3, 4, 5, 7 ou 8 mètres carrés.

Nous pouvons donc dire que dans la localité I les espèces à fréquence locale 6 dominent et qu'à côté d'elles les espèces correspondant aux fréquences inférieures ou supérieures se groupent en nombre décroissant.

Cette distribution des espèces suivant leur degré de fréquence de part et d'autre de la fréquence optimum présente la plus grande analogie avec la distribution des valeurs extrêmes d'un caractère organique quelconque par rapport à sa valeur moyenne. Aussi me paraît-il légitime *d'envisager la fréquence des espèces sur une portion déterminée d'une prairie comme un caractère organique de cette formation* au même titre par exemple que la longueur des feuilles l'est pour un arbre. Chez chaque espèce d'arbre, on observe que le plus grand nombre de feuilles correspond à une longueur moyenne ou longueur optimum de cet organe. La longueur optimum, c'est-à-dire celle présentant la fréquence maximum, varie légèrement d'une branche à l'autre et davantage encore d'un arbre à l'autre.

Poursuivant notre comparaison, nous pourrions exprimer par des courbes de *fréquence locale*, la fréquence relative des diverses longueurs de feuilles pour les diverses branches d'un arbre et par une courbe de *fréquence générale* les variations de ce même caractère pour l'ensemble des feuilles de l'arbre entier.

Les courbes ainsi obtenues présenteraient à côté d'inégalités provenant de ce que les diverses branches d'un arbre ne sont pas *exactement* placées dans les mêmes conditions vis-à-vis de la lumière ou de la température, une évidente ressemblance résultant des conditions générale de croissance communes à l'ensemble de l'arbre.

La ressemblance serait moins grande si la comparaison portait sur la longueur des feuilles appartenant à des arbres distincts, de même, qu'en envisageant la fréquence des espèces dans des prairies différentes les courbes obtenues sont plus dissemblables que celles qui concernent diverses localités rapprochées d'une même prairie. (Voir graphique n° 14 concernant les prairies du Jura méridional, Pl. XIX).

Nous ne prétendons pas, cela va sans dire, qu'il existe un parallélisme complet entre les deux termes de notre comparaison. Le rapprochement que nous venons de signaler entre la fréquence relative des diverses longueurs des feuilles d'un arbre et la fréquence relative des diverses espèces d'une prairie nous paraît toutefois reposer sur autre chose qu'une simple analogie superficielle.

Rien ne fait mieux ressortir cette analogie de nos courbes de fréquence avec les courbes de variation organique que l'examen des graphiques n°s 10 et 11 (Pl. XVI et XVII), traduisant la fréquence locale des espèces recueillies sur les 16 m^2 des localités I et III considérées comme formant une localité unique et que par abréviation j'appellerai localité (I + III). Les tracés correspondant aux huit[1] premiers mètres de la localité (I + III) expriment la fréquence relative des espèces suivant 16 degrés, ils présentent très régulièrement deux sommets principaux, le premier correspondant à la fréquence 5 ou 6, le second, à la fréquence 13 ou 14. (Voir graphique n° 10).

Le tracé moyen suivant 8 degrés de fréquence (M_8^{1-8} graph. 10) fait ressortir nettement ces deux sommets tombant sur les fréquences 6 et 14 entre lesquels les fré-

[1] Les tracés correspondant aux huit autres mètres présentent sensiblement la même allure ; pour ne pas allonger je ne les ai pas fait figurer dans le graphique n° 10, mais les documents qui concernent les 16 m^2 de la localité (I + III) figurent dans le tableau ci-joint n° 7.

quences 9 à 11 présentent des valeurs moitié plus faibles. Les tracés $M_{16}^{(I+III)}$ et $M_8^{(I+III)}$ du graphique 11, montrent

TABLEAU N° 7.

Nombre des espèces correspondant aux fréquences 1 à 16 dans chaque m² *des localités réunies* I et III (72 esp.).

m² 1 à 16	Fréquences																E
	1	2	3	4	5	6	7	8	9	10	11	12	13	14	15	16	
1	2	1	1	1	3	4	2	3	2	—	1	—	4	3	—	1	28
2	2	—	—	1	4	3	1	3	2	1	—	—	4	3	—	1	25
3	—	—	—	—	—	5	1	3	2	3	1	—	4	3	—	1	23
4	—	—	—	1	2	4	1	3	2	—	1	—	4	3	—	1	22
5	1	1	2	2	2	4	1	3	2	2	3	—	4	2	—	1	30
6	2	1	—	3	5	3	2	3	2	3	4	—	2	4	—	1	35
7	—	2	2	1	3	3	1	3	1	2	3	—	3	4	—	1	29
8	1	—	3	2	3	4	2	3	1	3	2	—	5	4	—	1	34
T^{1-8} =	8	5	8	11	22	30	11	24	14	14	15	—	30	26	—	8	$\frac{T}{8}$
M_{16}^{1-8} =	1	0,6	1	1,4	2,75	3,75	1,4	3	1,75	1,75	1,9	—	3,75	3,3	—	1	
M^8 =	1,6		2,4		6,5		4,4		3,5		1,9		7		—	1	
9	1	2	3	—	3	—	3	1	2	1	3	—	3	3	—	1	26
10	—	1	2	1	4	1	1	1	1	2	4	—	5	4	—	1	28
11	1	1	—	3	3	2	1	1	-	3	4	—	4	4	—	1	28
12	—	—	—	2	1	2	2	1	2	3	4	-	5	3	—	1	26
13	—	1	1	2	1	2	2	1	1	2	2	—	5	4	—	1	25
14	—	—	—	2	—	2	3	1	2	1	4	—	5	4	—	1	25
15	1	1	—	1	3	1	3	1	2	2	4	—	4	4	—	1	28
16	1	1	1	2	3	2	2	1	3	2	4	—	4	4	—	1	31
$T^{(9-16)}$	4	7	7	13	18	12	17	8	13	16	29	—	35	30	—	8	$=\frac{T^{(9-16)}}{8}$
$M_{16}^{(9-16)}$	0,5	0,9	0,9	1,6	2,3	1,5	2,1	1	1,6	2	3,6	—	4,4	3,75	—	1	
M_8 =	1.4		2,5		3,8		3,1		3,6		3,6		8,8		1		
T^{1-16} =	12	12	15	24	40	42	28	32	27	30	44	—	65	56	—	16	
M_{16}^{1-16} =	0,75	0,75	0,9	1,5	2,5	2,6	1,75	2	1,7	1,9	2,75	—	4,1	3,5	—	1	=27,7
M_8^{1-16} =	1,5		2,4		5,1		3,75		3,6		2,75		7,6		1		
M_4^{1-16} =	3,9[r]				8,85[a.c]				6,35[c]				8,6[cc]				=27,7
Proportions.	1	::			2	::			1,5	::			2				
$M_8^I+M_8^{III}$	=		2,3	2	2,6	7,5			5,1	12,1			14,2	10			
$M_4^I+M_4^{III}$	=		4,3[r]		10,1[ac]				17,2[c]				24,2[cc]				
½ $M_4^I+M_4^{III}$	—		2,15		5,05				8,6				12,1				=27,9
Proportions.			1	::	2	::			4	::			6				

TABLEAU N° 7bis.

Enumération des 72 espèces croissant dans les localités I et III réunies avec l'indication de leur degré de fréquence dans l'une et dans l'autre de ces deux localités [1].

Degrés de fréquence de 1 à 16.	Espèces				
1	7. 9. **11**. **12**. 27. 29. **49**. 64. 72. **84**. 91. 92.	= 12 esp.	dont 8. dans I;	4. dans III;	0. dans I et dans III.
2	**33**. 41 (1+1). **46**. **56**. 58. 62. . . .	= 6 »	» 2 »	» 3 »	» 1 » »
3	2. **22**. 45. **67**. 79 (2+1)	= 5 »	» 2 »	» 2 »	» 1 » »
4	**1** **18** 21 25 31 (3+1) **59**	= 6 »	» 2 »	» 3 »	» 1 » »
5	13. 40(1+4). 52. 55(1+4). 57. 80. **81**. **90**.	= 8 »	» 4 »	» 2 »	» 2 » »
6	14. 16. 38. 68. **74**. 78. 82.	= 7 »	» 5 »	» 2 »	» — » »
7	23. **37**. **50**. 76(4+3).	= 4 »	» 1 »	» 2 »	» 1 » »
8	6. 30. 61. **85**.	= 4 »	» 3 »	» 1 »	» — » »
9	34 (7+2). 51 (5+4). 89 (2+7). . .	= 3 »	» — »	» — »	» 3 » »
10	70 (4+6). 71 (6+4). 75 (4+6). . .	= 3 »	» — »	» — »	» 3 » »
11	10(4+7). 26(4+7). 44(3+8). 66(4+7)	= 4 »	» — »	» — »	» 4 » »
12	. . . .	= — »	» — »	» — »	» — » »
13	20(5+8). 35(6+7). 36(7+6). 54(7+6) 63(6+7)	= 5 »	» — »	» — »	» 5 » »
14	5 (6+8). 8 (6+8). 19(7+7). 39(7+7).	= 4 »	» — »	» — »	» 4 » »
15	. . . .	= — »	» — »	» — »	» — » »
16	83 (8+8)	= 1 »	» — »	» — »	» 1 » »
		72 esp.	dont 27 dans I,	19 dans III	et 26 dans I et dans III.

Chaque m² des localités I + III possède en moyenne 27 à 28 espèces.

[1] Chaque espèce est désignée par son numéro d'ordre dans le Tableau général N° 1.
Les chiffres **gras** concernent les espèces qui ne que figurent que dans la localité III.
» » ordinaires » » » » I.
» » entre ( ) concernant les espèces figurant à la fois dans I (1er chiffre) et dans III (2e chiffre de la parenthèse).

le caractère de la courbe de fréquence, le premier suivant 16 degrés, le second suivant 8 degrés pour les 16 m² de la localité combinée (I+III). Ces deux tracés présentent une remarquable analogie avec ceux qu'on obtient en inscrivant l'un à la suite de l'autre les tracés moyens M_4 et M_8 des localités I et III, de façon à obtenir soit 8 soit 16 degrés de fréquence[1] (courbes pointillées $\frac{1}{2}$ (M_8^{I}. M_8^{III}) et $\frac{1}{2}$ (M_4^{I}. M_1^{III}) du graphique 11). Les courbes ainsi construites se superposent presque exactement dans les premiers degrés de fréquence 1 à 8, avec les courbes correspontes M_8^{I+III} et M_4^{I+III}, et présentent vers la droite, correspondant aux fréquences 9 à 16, où les sommets ne coïncident plus, un parallélisme frappant.

Si l'on note, en se reportant au tableau 7 bis, les espèces qui dans les localités combinées présentent les divers degrés de fréquence, on constate : 1° que sur les 52 espèces à fréquence inférieure à 9, 6 seulement doivent leur degré de fréquence à leur présence dans les deux localités à la fois; 2° que sur les 20 espèces à fréquence supérieure à 8, 4 seulement ont la même fréquence sur I et sur III. L'examen de ce même tableau montre en outre : 1° que dans les localités I et III les mêmes degrés de fréquence sont presque toujours réalisés par des espèces différentes et, 2° que les espèces communes aux deux localités y possèdent dans le plus grand nombre des cas (22 contre 4) un degré de fréquence différent. Par la combinaison de deux localités distinctes et *non contiguës* A et B par exemple, on ne constitue donc pas, au point de vue de la distribution des espèces par ordre de fréquence, une localité A+B homogène. Le graphique n° 11 complété par le tableau n° 7 bis montre que le premier sommet Fréq. 6, du tracé M_8 (I + III) est, à

[1] Les valeurs correspondant aux localités I et III considérées séparément ont été divisées par 2, pour que le total de ces valeurs représente, comme dans toutes nos courbes de fréquence locale, le nombre *moyen* des espèces figurant sur 1 m² de la localité (I + III).

deux exceptions près (13 fois sur 15), formé par des espèces ayant déjà les fréquences 5 ou 6, soit dans III soit surtout dans I où elles constituent le degré optimum, tandis que le second sommet, Fréq. 14, provient 7 fois sur 9 d'espèces ayant dans la localité III les fréquences 7 et 8 qui y sont dominantes et dans la localité I les fréquences 6 ou 7 (qui y sont également dominantes). Ceci nous explique comment il se fait que le tracé $M_8^{(I+III)}$ concernant la distribution moyenne des espèces suivant 8 degrés de fréquence dans les localités I et III réunies présente une si grande ressemblance avec le tracé $\frac{1}{2}$ (M_4^{I}. M_4^{III}) obtenu en inscrivant l'une à la suite de l'autre les courbes de fréquence de ces mêmes localités séparées[1].

TABLEAU N° 8.

Nombres des espèces correspondant aux fréquences 1 à 9 dans chaque m² des localités VI et VII réunies **(46 esp.)**

m² 1-9	Fr. 1	2	3	4	5	6	7	8	9	E
1	2	3	1	5	—	—	3	3	8	25
2	—	6	4	5	—	—	3	3	8	29
3	1	2	4	5	—	—	3	3	8	26
4	4	2	3	5	—	—	3	3	8	28
5	—	1	1	1	4	—	4	2	8	21
6	1	—	—	2	4	—	3	2	8	20
7	2	—	—	2	4	—	3	3	8	22
8	—	—	1	1	4	—	3	3	8	20
9	—	—	1	2	4	—	3	2	8	20
T	10	14	15	28	20	—	28	24	72	$= \frac{T}{9}$
$M_9 =$	1,1	1,6	1,7	3,1	2,2	—	3,1	2,7	8	
$M_3 =$		4,4ʳ			5,3ᶜ			13,6ᶜᶜ		= 23,5
Proport. :		1	::		1,2	::		3		

Le graphique n° 12 (Pl. XVIII) concernant les deux localités VI et VII combinées, confirme ce que nous venons de dire

[1] Il ne faut pas confondre la courbe $\frac{1}{2}$ (M_8^{I}. M_8^{III}) avec la courbe $\frac{1}{2}$ M_8^{I} + M_8^{III} (tracé vert) correspondant à la somme des fréquences 1 à 8 dans les deux localités I et III, et qui, naturellement, présente une toute autre physionomie.

au sujet des localités I et III combinées; la courbe de fréquence locale moyenne $M_9^{(VI+VII)}$ possède deux sommets principaux 5 et 9 correspondant aux sommets 4 et 5 des localités séparées et formées surtout par des espèces ayant les fréquences 4 et 5 dans les localités VI et VII. (Voir aussi : Tableaux n° 8 et 8bis.)

TABLEAU N° 8bis.

Enumération des 48 esp. des localités VI et VII réunies, groupées d'après leur degré de fréquence dans ces deux localités.

Fr.		VI	VII	VI + VII
1	1. 8 **29** 32 44 61 68 **69** 71. 81. = 10.	8	2	—
2	22 27 (1+1) 43. 64. 67. 79. 80. = 7.	6	—	1
3	4 9 **16** 21 3 . . . = 5.	4	1	—
4	7. 19 40. 42. **57.** **63** 82 = 7.	5	2	—
5	**2** **6** **36** **39** . . . . . = 4	—	4	—
6	. . . . = 0	—	—	—
7	13(4+3) 20(2+5) 51(4+3) 89(2+5)= 4	—	—	4
8	35 (4+4) 55 (4+4) 77 (4+4) . . = 3	—	—	3
9	5.25. 30.54. 62. 70. 78. 83 (4+5) = 8	—	—	8

Chaque point de la prairie est donc caractérisé par une courbe de fréquence locale particulière, c'est-à-dire par une relation déterminée entre les divers degrés de fréquence des espèces qui s'y trouvent. Ces courbes de fréquence qui varient naturellement avec la surface (nombre de m²) envisagée sont également variables d'un point à un autre de la prairie, alors même que l'on compare entre elles des surfaces équivalentes. Enfin, comme nous venons de le voir, le caractère local des courbes de fréquence ne disparaît pas par la combinaison de deux localités distinctes (graphiques 10 et 11).

On peut rapprocher ce fait de ce qu'on observe dans l'établissement des courbes de variation organique concernant un mélange d'individus de deux races dont les caractères respectifs se traduisent dans la courbe par la formation de deux sommets principaux distincts.

Courbes de fréquence locale dans les prairies du Jura méridional.

Comme terme de comparaison j'ai déterminé, en m'appuyant sur les documents publiés antérieurement dans mon Etude sur les prairies pâturages du Jura méridional[1], la fréquence locale de 240 espèces distribuées dans 12 localités d'environ 1 ha. de superficie moyenne et s'étendant de la Dôle au Reculet.

Le nombre des espèces correspondant dans chaque localité aux degrés de fréquence 1 à 12 est indiqué par les tableaux N°s 9 et 9 bis et par les divers tracés du Graphique N° 13, Pl. XIX.

Ces tracés sont groupés deux à deux suivant leur degré de ressemblance.

Les localités ainsi comparées deux à deux sont les suivantes :

I. Pente à exposition Est sous le sommet du Colombier, entre 1480 et 1580 m. avec 106 esp., et II. Pente à exposition S.-O. sous le sommet du Reculet entre 1480 et 1580 m. avec 93 esp. Coeff. de com.. . . 41 %

III. Pente à exposition S.-E. sous le sommet du Reculet entre 1500 et 1600 m. avec 78 esp., et IV. Pente à exposition E. sous le sommet du Reculet entre 1500 et 1600 m. avec 65 esp. Coeff. de com. . . . 41 %

V. Pente à exposition N.-E. sous le sommet du Montoisey entre 1530 et 1600 m. 56 esp. et IX. Sommet du Montoisey entre 1650 et 1670 m. 56 esp. Coeff. de com. 50 %.

VI. Sommet du Colombier entre 1650 et 1690 m. avec 110 esp., et XII. Pente à exposition E. sous le sommet de la Dôle entre 1480 et 1580 m. env., avec 88 esp. Coeff. de com. . . . . 39 %

[1] Voir : *Etude comparative de la distribution florale dans une portion des Alpes et du Jura.* Bull. Soc. vaud. sc. nat. vol. XXXVII.

VII. Sommet occidental du Colombier entre 1650 et 1680 m. env., avec 81 esp., et VIII. Sommet du Reculet entre 1640 et 1720 m. env. avec 90 esp. Coeff. de com. . . . . , . 40 %

X. Sommet du Mont-Tendre entre 1600 et 1680 m. env., avec 115 esp., et XI. Sommet de la Dôle entre 1600 et 1680 m. env., avec 148 esp. Coeff. de com. . 50 %

TABLEAU N° 9.

Nombres des espèces correspondant aux fréquences 1 à 12 dans 12 localités du Jura méridional.

Localités I à XII	Degrés de Fréquence. 1	2	3	4	5	6	7	8	9	10	11	12	Nombre des espèces par localité.
I	17	5	7	8	10	5	8	10	9	9	8	9	= 106
II	11	4	6	7	10	6	6	7	11	8	8	9	= 92
X	11	5	7	13	10	11	9	11	9	10	7	9	= 78
XI	19	12	10	18	18	11	6	14	10	12	9	9	= 64
Total (I, II, X, XI.)	58	26	30	46	48	33	29	42	39	39	32	36	= T_4
	160				152				146				
$M_s = \frac{T_4}{4} =$	40 r.				38 c.				36,5 cc.				= 114,5
III	1	2	4	6	10	6	8	10	9	9	8	9	78
IV	—	1	3	4	6	5	2	7	8	12	7	9	64
V	1	—	1	1	3	7	3	6	8	11	6	9	56
VI	1	5	10	15	8	10	9	13	11	12	8	9	111
VII	2	2	3	7	10	7	6	10	7	11	8	9	82
VIII	2	3	2	9	10	8	7	10	7	11	8	9	89
IX	—	—	2	4	5	8	3	6	4	9	6	9	56
XII	1	5	5	9	9	5	6	9	8	12	8	9	86
Total (III à IX + XII)	8	18	30	55	61	58	41	68	62	90	59	72	= T_8
	111				228				283				
$M_s = \frac{T_8}{8} =$	14 r.				28,5 c.				35,4 cc.				= 77,9
Total (I-XII) =	66	44	60	101	109	91	70	110	101	129	91	108	= T_{12}

Les analogies observées ne dépendent pas de la valeur des coefficients de communauté des localités comparées. On constate en effet que la ressemblance des courbes de fréquence figurées par le graphique N° 13 est plus grande entre les localités VI et VII avec C. c. = 40 % ou entre V et IX avec C. c. = 50 % qu'entre VI et VII avec C. c. = 62 % ; d'autre part, elle n'est pas

TABLEAU N° 9bis.

Nombres des espèces correspondant aux fréquences 1 à 12 dans 12 localités du Jura méridional. (Calculés pour 100 esp. par localités).

Localités I à XII	Degrés de Fréquence. 1	2	3	4	5	6	7	8	9	10	11	12	Nombre des espèces par localité.
I	16	4,7	6,6	7,6	9,4	5,7	7,6	9,4	8,5	8,5	7,6	8,5	100 esp.[1]
II	12,0	4,4	6,6	7,7	11.	5,5	6,6	7,7	12,0	8,8	8,8	9,9	» »
X	9,7	4,4	6,3	11,5	8,9	9,7	8.	9,7	8.	8,9	6,3	6,1	» »
XI	13.	8,1	6,8	12,2	12,2	7,5	4.	9,5	6,8	8,1	6,1	6,1	» »
Total (I, II, X, XI)	50,7	21,6	26,3	39.	41,5	28,4	26,2	36,3	35,3	34,3	28,8	32,5	= T_4
	137,6				132,4				130,9				
$M_s = \frac{T_4}{4} =$	34,4 r.				33,1 c.				32,7				= 100,2
III	1,3	2,6	5,1	7,7	12,9	10,2	6,4	9.	9.	12,8	11,5	11,5	100 esp.[1]
IV	—	1,5	4,7	6,2	9,4	7,8	3,1	10,9	12,5	18,7	10,9	14.	» »
V	1,8	—	1,8	1,8	5,4	12,5	5,4	10,7	14,3	19,7	10,7	16,1	» »
VI	0,9	4,5	9.	13,5	7,2	9.	8,1	11,7	9,9	10,8	7,2	8,1	» »
VII	2,4	2,4	3,7	8,5	12,2	8,5	7,3	12,2	8,5	13,4	9,8	11.	» »
VIII	2,2	3,4	2,2	10,1	11,2	9.	7,8	11,2	10,1	14,6	8,5	10,1	» »
IX	—	—	3,6	7,2	9.	14.	5,4	10,8	7,2	16.	10,8	16.	» »
XII	1,2	5,8	5,8	10,4	10,4	5,8	7.	10,4	9,3	14.	9,3	10,4	» »
Total (III à IX + XII)	9,8	20,2	35.9	65,4	77,7	76,8	50,5	86,9	80,8	120.	78,7	97,2	T_8
	131,3				291,9				376,7				
$M_s = \frac{T_8}{8} =$	16,4 r.				36.5 c.				47,1 cc.				= 100

[1] En chiffre rond à quelques dizaines près.

plus grande entre X et XI avec C. c. = 50 % qu'entre VI et XII dont le C. c. n'est que de 39 %.

Quant aux dissemblances, les plus fortes s'observent entre les localités II et IV, II et VIII, IV et XI ou encore entre I et VI dont les coefficients de comm. (40 %, 36 %, 33 % et 40 %) ne diffèrent pas davantage des C. c. cités plus haut que ces derniers ne diffèrent entre eux.

Les ressemblances qui se traduisent par l'analogie des courbes de fréquence *ne sont donc pas du même ordre que celles qui s'expriment par les coefficients de communauté.*

Ce qui, plus que l'analogie de composition florale spécifique, exerce une influence sur le degré de ressemblance des courbes de fréquence, c'est le *nombre* des espèces croissant sur chaque localité[1], autrement dit la richesse florale des localités comparées.

La comparaison des espèces correspondant aux divers degrés de fréquence dans les localités 1 à 12 du Jura méridional confirme en outre ce que la comparaison des diverses localités de la prairie des Ormonts nous a déjà révélé, *c'est que le même degré de fréquence n'est pas réalisé par les mêmes espèces dans les diverses localités*[2].

Fréquence générale relative.

Indépendamment de la *fréquence générale absolue* exprimant la relation qui existe entre les divers degrés de

[1] Les tracés rouges superposés aux courbes V et IX du graphique N° 13 ont été construits en calculant la proportion des divers degrés de fréquence sur 100 espèces au lieu de 56 (Voir les chiffres correspondants dans le tableau 9 bis).

Comme on pouvait s'y attendre, le caractère des tracés centésimaux (rouges) ne diffère pas sensiblement de celui des tracés noirs calculés d'après le nombre réel des espèces.

[2] Voir encore à ce sujet : *Distribution florale dans les Alpes et le Jura* (Comparaison du degré de fréquence des espèces). Bull. soc. vaud. sc. nat. vol. XXXVII, pages 567 et suivantes.

fréquence des espèces par rapport aux 52 m² explorés, j'ai cherché à déterminer quelle est, dans *chaque m²* des localités I à VIII du tableau n° 1, la proportion des espèces correspondant aux divers degrés de fréquence générale, ce que l'on peut appeler la *fréquence générale relative* des espèces. Pour y arriver, ramenons à 6 degrés seulement la fréquence générale, en attribuant la fréquence 1 aux espèces qui figurent dans 1 à 8 m², la fréquence 2 à celles qui figurent dans 9 à 16 m², la fréquence 3 de 17 à 24 m², la fréquence 4 de 25 à 32 m², la fréquence 5 de 33 à 40 m², la fréquence 6 enfin, aux espèces qui figurent dans 41 à 48 m²[1].

Le tableau N° 10 nous montre que sur chaque m² de la

TABLEAU N° 10.

Distribution des espèces dans les localités I-VIII suivant leur fréquence générale réduite à 6 degrés.

Degré 1, espèces figurant dans 1 à 8 m²; degré 2, espèces figurant dans 9 à 16 m²; degré 3, dans 17 à 24 m²; degré 4, dans 25 à 32 m²; degré 5, dans 33 à 40 m²; degré 6, dans 41 à 48 m²[1].

E nombre des espèces dans chaque mètre carré (r — rares ; c — communes ; cc — très communes ; Fr. — degrés de fréquence de 1—6).

m² 1 à 8	Localité I Fr.						E	m² 1 à 6	Localité II Fr.						E
	1	2	3	4	5	6			1	2	3	4	5	6	
1	6	2	6	6	3	5	28	1	2	3	1	7	4	4	21
2	4	3	3	5	4	6	25	2	3	6	4	6	4	4	27
3	2	3	2	8	4	4	23	3	3	4	4	8	4	6	29
4	3	2	3	6	4	4	22	4	3	3	5	8	3	6	28
5	4	4	4	9	4	5	30	5	2	4	4	10	3	3	26
6	3	7	8	8	4	5	35	6	4	4	4	5	4	4	25
7	6	3	4	8	3	5	29	—		—		—			—
8	6	6	4	8	4	6	34	—		—		—			—
T =	34	30	34	58	30	40		T =	17	24	22	44	21	27	
M_6 =	4,3	3,8	4,3	7,3	3,8	5	$= \frac{T}{8}$	M_6 =	2,8	4	3,7	7,3	3,5	4,5	$= \frac{T}{6}$
M_3 =	8,1 r.		11,6 c.		8,8 cc.		=28,5	M_3 =	6,8 r.		11 c.		8 cc.		=25,8

[1] Aucune espèce ne figurant dans 49-52 m², 41-48 représentent le maximum de fréquence observée.

[1] Aucune espèce du tableau I ne se rencontre sur plus de 48 m².

m² 1 à 8	Localité III Fr.						E
	1	2	3	4	5	6	
1	7	3	3	5	4	4	26
2	4	5	4	6	3	6	28
3	4	7	3	6	3	5	28
4	1	6	3	7	3	6	26
5	3	6	2	6	2	6	25
6	1	4	4	7	3	6	25
7	4	4	5	7	3	5	28
8	5	6	4	8	3	5	31
T =	29	41	28	52	24	43	
M_6 =	3,6	5,1	3,5	6,5	3	5,4	$=\frac{T}{8}$
M_2 =	8,7 r.		10 c.		8 cc.		=27,1

m² 1 à 6	Localité IV Fr.						E
	1	2	3	4	5	6	
1	1	3	4	5	4	6	23
2	1	5	4	6	4	4	24
3	1	5	4	6	5	5	26
4	1	5	3	6	4	5	24
5	—	5	4	6	5	5	25
6	2	5	3	7	3	6	26
—		—		—			—
—		—		—			—
T =	6	28	22	36	25	31	
M_6 =	1	4,7	3,7	6	4,2	5,2	$=\frac{T}{6}$
M_2 =	5,7 r.		9,7 c.		9,4 cc.		=24,8

m² 1 à 6	Localité V Fr.						E
	1	2	3	4	5	6	
1	1	3	3	4	5	6	22
2	1	1	4	6	5	6	23
3	1	2	2	6	5	6	22
4	—	3	2	5	5	6	21
5	1	3	2	7	4	5	22
6	—	3	3	7	4	6	23
T =	4	15	16	35	28	35	
M_6 =	0,7	2,5	2,7	5,8	4,7	5,8	$=\frac{T}{6}$
M_2 =	3,2 r.		8,5 c.		10,5 cc.		=22,2

m² 1 à 4	Localité VI Fr.						E
	1	2	3	4	5	6	
1	6	3	3	5	4	4	25
2	11	3	3	4	4	4	29
3	8	3	3	3	5	4	26
4	7	4	4	4	5	4	28
—		—		—			—
—		—		—			—
T =	32	13	13	16	18	16	
M_6 =	8	3,3	3,2	4	4,5	4	$=\frac{T}{4}$
M_2 =	11,3 r.		7,2 c.		8,5 cc.		=27

m² 1 à 5	Localité VII Fr.						E
	1	2	3	4	5	6	
1	—	3	3	4	5	6	21
2	1	2	3	5	4	5	20
3	2	2	3	4	5	6	22
4	—	2	3	5	4	6	20
5	—	1	4	4	5	6	20
—		—		—			—
T =	3	10	16	22	23	29	
M_6 =	0,6	2	3,2	4,4	4,6	5,8	$=\frac{T}{5}$
M_2 =	2,6 r		7,6 c.		10,4 cc.		=20,6

m² 1 à 7	Localité VIII Fr.						E
	1	2	3	4	5	6	
1	1	1	7	4	6	6	25
2	1	—	6	5	5	6	23
3	—	1	5	6	6	5	23
4	—	1	5	5	5	6	22
5	1	4	6	4	6	4	25
6	1	3	5	4	5	5	23
7	1	2	6	5	6	6	26
T =	5	12	40	33	39	38	
M_6 =	0.7	1,7	5,7	4,7	5,6	5,4	$=\frac{T}{7}$
M_2 =	2,4 r.		10,4 c.		11 cc.		=23,8

localité I par exemple, on trouve en moyenne 4,3 espèces ayant la fréquence générale I ; 3,8 ayant la fréquence gé-

nérale 2 ; 4,3 ayant la fréquence générale 3 ; 7,3 ayant la fréquence générale 4 ; 3,8 ayant la fréquence générale 5, et 5 ayant la fréquence générale 6. Le tracé I du graphique N° 14 (Pl. XX) traduit ces chiffres graphiquement et représente la *courbe de fréquence générale relative* pour la localité I.

Comme on pouvait s'y attendre, les courbes de fréquence ainsi obtenues diffèrent très sensiblement des courbes de fréquence locale, par contre, elles présentent entre elles, de même que ces dernières une évidente analogie. Cette ressemblance n'apparaît, il est vrai, nettement, qu'entre les localités I à IV soit entre les « pentes ». (Ce sont les seules qui figurent dans le Graphique N° 14.) Les tracés concernant les « plats » (localités V à VIII) seraient beaucoup moins réguliers, ainsi qu'on peut s'en rendre compte en comparant les chiffres du tableau N° 10.

Les localités I, II et IV sont situées sur le versant gauche de la vallée (exposition sud) et sont éloignées l'une de l'autre de 4 à 500 mètres ; la localité III par contre est située sur la rive gauche, exposée au nord et à une distance de 1100 à 1200 m. des précédentes.

Les localités VI, VII et VIII sont situées sur la rive gauche, mais sur des terrains plats ; elles sont distantes l'une de l'autre de 100 à 600 m. ; quant à la localité V elle est contiguë à la localité IV de la rive droite, mais ses 6 m² sont disposés non pas dans le sens de la pente comme dans IV, mais dans le sens *horizontal*.

Le coefficient moyen de communauté est de 35 °/₀ pour les « plats » (localité V à VIII) tandis que pour les « pentes » (localités I à IV) il s'élève à 45 °/₀.

Toutefois la ressemblance ne dépend pas directement du C. c. puisqu'entre V et VII qui ont des fréquences générales relatives très semblables (voir tableau N° 10 *bis*) le C. c. n'est que de 28 °/₀, tandis qu'entre VII et VIII qui possèdent un C. c. de 49 °/₀, la ressemblance est moindre.

La courbe moyenne de fréquence générale relative suivant

TABLEAU 10bis.

$M_6^{(I-IV)}$ Distribution moyenne suivant 6 degrés de fréquence dans les localités I à IV.

$M_3^{(I-IV)}$ Distribution moyenne suivant 3 degrés de fréquence dans les localités I à IV.

$M_3^{(V-VIII)}$ Distribution moyenne suivant 3 degrés de fréquence dans les localités V à VIII.

$M_3^{(I-VIII)}$ Distribution moyenne suivant 3 degrés de fréquence dans les localités I à VIII.

Fréquences :

	1	2	3	4	5	6
$M_6^{(I-IV)}$	$\frac{11,7}{4}$	$\frac{17,6}{4}$	$\frac{15,2}{4}$	$\frac{27,1}{4}$	$\frac{14,5}{4}$	$\frac{20,1}{4}$
$M_6 =$	2.9	4,4	3,8	6,8	3,6	5

Nombre moyen des espèces :

		rares	communes	très communes	
M_3	I =	8,1	11,6	8,8	
	II =	6,8	11.	8.	
	III =	8,7	10.	8,4	
	IV =	5,7	9,7	9,4	
	T =	29,3	42,3	34,6	
$M_3^{(I-IV)} = \frac{T}{4}$	=	7,3	10,6	8,7	= 26,6 esp.
M_3	V =	3,2	8,5	10,5	
	VI =	11,3	7,3	8,5	
	VII =	2,6	7,6	10,4	
	VIII =	2,4	10,4	11.	
	T′ =	19,5	33,8	40,4	
$M_3^{(V-VIII)} = \frac{T'}{4}$	=	4,9	8,5	10,1	= 23,5 esp.
$M_3^{(I-VIII)} = \frac{T+T'}{8}$	=	6,1 r.	9,6 c.	9,4 cc.	= 25,1 esp.

Coefficients de communauté moyens entre les localités :

(Pentes)	(Plats)
I et II = 55 %	V et VI = 25 %
I et III = 36 %	V et VII = 28 %
I et IV = 47 %	V et VIII = 39 %
II et III = 41 %	VI et VII = 33 %
II et IV = 49 %	VII et VIII = 49 %
C.c. moyen = 45 %	C. c. moyen = 35 %

C. c. entre III et VIII = 37 %.

3 degrés ($M_3^{(I \text{ à } IV)}$ graphique 14) qu'on obtient pour les localités en pentes I à IV, montre très distinctement que ce sont les espèces à fréquence générale moyenne (degrés 3 et 4) soit les espèces *communes* qui dominent dans ces localités (sur 26 à 27 par m² il s'en trouve en moyenne 10 à 11), tandis que dans les localités plates V à VIII ce sont les espèces très communes, (10 sur 23 en moyenne par m²) qui sont les plus nombreuses ($M_3^{V \text{ à } VIII}$).

Les espèces dominantes dans ces deux groupes de localités pourtant rapprochées, non seulement sont différentes, mais encore *n'ont pas, dans le territoire restreint que nous envisageons le même degré de fréquence générale.*

En résumé, non seulement la proportion des espèces correspondant aux divers degré de fréquence locale des espèces, mais encore celle correspondant à leurs divers degrés de fréquence générale varient d'un point à un autre de la prairie.

Fréquence individuelle.

Dans tout ce que nous avons dit jusqu'ici, il n'a été question que de la fréquence relative des *espèces*, soit de la *fréquence spécifique* ; il serait intéressant de connaître aussi la fréquence relative des individus, soit la *fréquence individuelle.* Jusqu'ici les documents me manquent pour l'établir d'une façon certaine; on reconnaîtra d'ailleurs qu'il n'est pas facile de faire pour une prairie d'une certaine étendue, de 100 m² par exemple, le relevé complet et exact de tous les individus qui s'y trouvent.

On peut supposer a priori, toutefois, que la courbe des divers degrés de fréquence individuelle pour une prairie déterminée présenterait une allure analogue à celle qui concerne la fréquence spécifique.

Il est en effet vraisemble d'admettre que le nombre total des individus des diverses espèces croissant sur une prai-

rie telle que celle dont nous nous occupons, doive être d'autant plus grand que l'espèce à laquelle ils appartiennent apparaît sur un plus grand nombre de mètres carrés. Le nombre des individus doit croître d'ailleurs non pas en proportion arithmétique mais en raison géométrique de la fréquence de l'espèce, c'est-à-dire que, tandis qu'une espèce à fréquence 2 serait représentée par 4 individus, par exemple, une autre espèce à fréquence 5 pourra l'être par 25 et une à fréquence 10 par 100. Ceci n'est qu'un exemple, car il est peu probable que la fréquence individuelle soit exactement proportionnelle au carré de la fréquence spécifique.

La relation qui existe entre ces deux ordres de fréquence ne saurait d'ailleurs être ni constante, ni identique pour les individus des diverses espèces, attendu que la taille et l'exubérance fort inégales de celles-ci influent considérablement sur le nombre de leurs individus. (Comparez à cet égard les Graminées fourragères avec les Anthrisques, les Géraniums, etc.)

En prenant pour base le chiffre moyen d'un millier d'espèces par m² obtenu par divers dénombrements on arriverait pour les 52 m² de notre prairie, en supposant la fréquence individuelle proportionnelle au carré de la fréquence spécifique, aux chiffres approximatifs suivants: pour les espèces rares (fréquence générale 1 à 12) 1600 individus environ, pour les espèces assez communes (fréq. gén. 13 à 24) 5000 individus environ, pour les espèces communes (fréq. gén. 26 à 36) 12 à 13 mille, enfin pour les espèces très communes (fréq. gén. 37 à 48) environ 15 mille individus, soit au total approximativement 34 à 35 mille). Or en partant du chiffre moyen de 1000 individus par m², les 52 m² de notre prairie devraient compter environ 52 000 individus.

En supposant le nombre des individus proportionel au cube des fréquences spécifiques on obtiendrait par contre un chiffre beaucoup trop élevé.

J'espère pouvoir bientôt être à même d'établir pour une petite surface de prairie la relation existant entre la fréquence spécifique et la fréquence individuelle.

Coefficient générique.

Les 92 espèces du tableau général n° 1, appartiennent à 74 genres différents. Le coefficient générique de notre prairie s'élève donc à 79 %, ce qui est conforme à la loi du cœfficient générique telle que je l'ai établie dans mes « Lois de distribution de la flore dans la zone alpine », et d'après laquelle la valeur du *coefficient générique est inversément proportionnelle à la variété des conditions écologiques*. Les conditions écologiques dont nous avons à tenir compte ici concernent les variations *locales* de notre prairie et non les variations *élémentaires* qu'il est difficile d'apprécier (voir plus haut page 231.) Le sous-sol étant comme nous l'avons vu, uniformément recouvert par des alluvions, ce sont en particulier les variations de la déclivité et celle de l'exposition qui interviennent comme causes favorisantes vis-à-vis de la diversité florale. Mais il est un autre facteur encore dont nous avons à tenir compte pour expliquer le cœfficient générique élevé mentionné plus haut: c'est *l'étendue*.

Une portion restreinte de prairie, les 52 m² que nous avons envisagés par exemple, réalise un ensemble de conditions écologiques capable de convenir à un nombre d'espèces beaucoup plus considérable que celui qui s'y trouve en réalité. Si le plus grand nombre des espèces « possibles[1] » ne s'y rencontre pas c'est qu'il s'établit entre elles une concurrence d'autant plus intense que les variations écologiques locales sont moins considérables, autrement dit que l'*uniformité écologique* est plus grande.

[1] C'est-à-dire celles qui ne sont pas exclues par les conditions écologiques propres à la station.

Cette concurrence intensive s'accentue d'autant plus que les conditions écologiques s'uniformisent davantage et se manifeste d'une façon générale chaque fois que soit un seul, soit plusieurs des facteurs écologiques atteignent une valeur extrême. Elle se traduit régulièrement par une *sélection éliminatoire* proportionnellement plus forte pour les espèces que pour les genres. Dans notre prairie des Ormonts, le facteur qui se rapproche le plus d'une de ses valeurs extrêmes c'est *l'étendue*. A mesure qu'on envisage une portion plus petite de la prairie, on constate non seulement une diminution de la richesse florale mais invariablement une augmentation du coefficient générique, c'est-à-dire du nombre des genres correspondant à un nombre déterminé d'espèces.

Sans l'avoir vérifié sur place, je suis certain que 100 m² choisis dans des conditions analogues à celles de nos 52 m., et répartis sur une étendue de 2 km² (soit sur une étendue double de celle que nous avons envisagée) non seulement nous fournirait un nombre d'espèces plus considérable, mais encore un cœfficient générique moins élevé que celui que nous avons obtenu. Inversément, en rescençant la flore de 52 m² répartis sur une superficie de 1 ha. seulement nous obtiendrions à la fois moins d'espèces et un cœfficient générique plus élevé.

La comparaison des localités de la prairie du Jura méridional dont nous nous sommes occupés plus haut présente à cet égard un réel intérêt. Le Colombier de Gex possède deux sommets d'apparence absolument semblable, distants l'un de l'autre de 1 km. seulement et différant de 10 m. seulement en altitude.

Le premier, à partir de 40 m. au-dessous du point culminant, compte 110 espèces avec un coefficient générique = 75 %; le second, sur une surface un peu moindre, n'a que 81 espèces avec un coefficient générique = 77 %. La florule des deux sommets réunis possède par contre un coefficient générique = 70 % soit de 5 à 7 % moins élevé,

diminution qui correspond à l'augmentation d'étendue de la surface florale envisagée.

Autre exemple : la localité nº 1, pente uniforme, située sur le flanc Est du Colombier de Gex et ne bénéficiant pas des variations d'exposition et des petits affleurements rocheux existant vers le sommet, *ayant par conséquent des conditions écologiques moins variées*, possède, à égalité d'étendue, 106 espèces avec un coefficient générique = 85 %. Rappelons en outre que pour les 12 localités du Jura méridional comprises entre la Dôle et le Reculet (voir page 249), le coefficient générique s'abaisse à 60 %. Sans doute, l'étendue ne constitue pas un facteur du même ordre que la chaleur ou la lumière. Au point de vue qui nous occupe, *l'étendue représente plutôt l'amplitude des variations possibles* des conditions écologiques dans une station déterminée, en même temps que *la valeur quantitative absolue d'espace et de nourriture disponibles.*

Plus l'étendue augmente plus augmente aussi la possibibilité et l'amplitude des variations élémentaires et des variations locales, et plus en définitive s'accroît *la diversité des conditions écologiques.*

Nous en pouvons conclure que d'une façon toute générale, *dans une prairie déterminée, le coefficient générique diminue à mesure que l'étendue qu'on envisage augmente.*

* * *

Proportion relative des représentants des principales subdivisions du règne végétal

Le nombre des espèces de notre prairie étant très restreint, je n'ai pas été peu surpris de voir que les relations que j'avais signalées à propos de la flore de la zone alpine[1] entre

[1] Voir : *Lois de distribution*, p. 105 et suivantes.

les coefficients génériques des grandes subdivisions florales, et le coefficient générique total se vérifient également sur les 92 espèces du tableau nº 1.

En établissant le nombre des espèces et celui des genres qui dans le tableau en question appartiennent aux Dialypétales, aux Gamopétales ainsi qu'aux Composées, on obtient le tableau suivant qui nous montre entre les coefficients génériques de ces grandes subdivisions d'une part et le coefficient générique total d'autre part, une concordance qui, pour être moins grande que celle que nous avons observée dans des territoires, plus étendus, est d'autant plus remarquable, que le nombre des espèces considéré est plus réduit.

Nombre des genres	Nombre des espèces		
23	31	Coeff. gén. des Dialypétales	76 %
26	34	» » des Gamopétales	74 %
10	13	» » des Composées	77 %
74	92	» » total	79 %

La concordance que nous avons précédemment signalée entre les cœfficients génériques des Dialypétales et des Gamopétales sur des territoires d'étendue et de richesse florale très différentes, apparaît donc également sur une surface relativement très restreinte de la prairie et acquiert par ce fait *le caractère d'une relation quasi élémentaire*. Il semble en conséquence logique d'en conclure, comme nous l'écrivions récemment[1], que les végétaux de ces deux grandes subdivisions florales (Dialypétales et Gamopétales) possèdent un pouvoir d'adaptation sensiblement équivalent, puisque dans la concurrence qui s'établit entre elles pour la conquête du terrain, leurs divers genres, présentent une diversité spécifique semblable.

Il serait donc plausible d'envisager les individus végétaux non seulement comme les représentants de telle espèce plus ou moins bien adaptée, mais encore comme les champions de groupes biologiques plus élevés, genres, ordres et classes, auxquels ils assurent une proportion déterminée dans la distribution totale.

[1] *Revue générale des sciences*, Paris. Nº 23 du 15 décembre 1907.

Conclusions.

L'analyse détaillée de la distribution de la flore dans la prairie subalpine des Ormonts, non seulement confirme d'une façon complète les lois de distribution florale telles que je les ai établies pour la zone alpine, mais encore permet de leur accorder une valeur tout à fait générale. En appliquant à des surfaces restreintes d'une prairie déterminée la méthode statistique et comparative employée pour l'étude de territoires plus étendus, nous arrivons à cette conclusion d'un incontestable intérêt, c'est que les lois de distribution des espèces végétales constituant nos *prairies naturelles* n'expriment pas des *relations moyennes* entre des valeurs inégales dont les extrêmes se compenseraient, relations dont le degré de précision dépendrait de l'étendue plus ou moins grande du territoire envisagé et du nombre des espèces qui s'y trouvent, mais que la plupart d'entre elles traduisent des *relations élémentaires*, susceptibles d'être décelées sur des surfaces de quelques m² seulement, occupées par un nombre d'espèces ne dépassant pas 100.

La diversité florale que nous avons mise tout d'abord en évidence en comparant des localités d'une superficie moyenne de 1 ha. environ, et dont nous avons donné la mesure par la détermination du *coefficient de communauté*, se manifeste dans les mêmes proportions, qu'il s'agisse de la flore de deux surfaces de 1 Ha. chacune ou de celle de deux surfaces de 1 m² chacune seulement.

En ce qui concerne la distribution par ordre de fréquence nous avons distingué, indépendamment de la *fréquence générale* des espèces, leur *fréquence locale* et constaté qu'en

chaque point de la prairie les espèces possèdent un degré de fréquence, autrement dit, une *densité* différente.

Enfin, distinguant la *fréquence générale relative* de la *fréquence générale absolue*, nous avons vu que sur chacune de nos localités ce sont tantôt les espèces *communes*[1], tantôt les espèces *très-communes*[1] qui prédominent et que ces différences sont en relation avec le caractère écologique dominant (déclivité) des localités considérées.

L'établissement des courbes de fréquence locale nous permet d'ajouter un nouveau terme à ceux que nous avons déjà proposés précédemment pour la comparaison de deux localités distinctes d'une même formation.

Pour comparer aussi complètement que possible deux localités d'une prairie déterminée, il y a donc lieu de tenir compte : 1° de leur *richesse florale*, c'est-à-dire du *nombre* de leurs espèces, 2° de leur *composition florale*, c'est-à-dire du *nom* de leurs espèces, 3° de leur *coefficient de communauté*, 4° de leur *coefficient générique*, 5° de leur *courbe de fréquence locale*. Pour être complet on devrait encore ajouter : 6° de leur *courbe de fréquence individuelle*, ainsi que de la *taille* moyenne relative des individus des diverses espèces; mais les difficultés pratiques auxquelles se heurte l'établissement de pareilles courbes ne permettront guère d'y recourir utilement.

En présence de l'impossibilité où nous sommes de déterminer *directement* la valeur des conditions écologiques dans une station donnée et surtout d'apprécier leur valeur moyenne efficiente vis-à-vis de la végétation, n'y a-t-il pas un réel intérêt à pouvoir évaluer d'une manière *indirecte*, par une méthode constante et dont les données soient comparables, les *caractères différentiels* de deux localités d'une même station ou d'une même formation?

---

[1] Ces désignations se rapportent à leur fréquence dans les 52 m² et non à leur fréquence locale ou régionale.

Aucun des cinq critères que nous venons d'énumérer : richesse florale, composition florale; coefficient de communauté, cœfficient générique et courbe de fréquence locale, ne suffit à lui seul pour déterminer le degré d'analogie que présentent deux localités d'une prairie; par contre, il me paraît certain que deux localités qui, à côté d'un cœfficient de communauté élevé, présenteraient dans leur richesse florale, dans leur cœfficient générique et dans leur courbe de fréquence locale une concordance manifeste, pourraient être considérées comme possédant des conditions écologiques semblables.

Toutefois, bien qu'il paraisse légitime d'inférer du degré de ressemblance des caractères floraux de deux localités le degré d'analogie des conditions écologiques cause de ces ressemblances, les résultats ainsi obtenus ne peuvent guère avoir qu'un intérêt théorique, car, en s'appuyant sur ce que nous savons de l'infinie diversité de composition et de distribution florales du tapis végétal de nos prairies, il semble que la recherche de localités en tous points comparables soit parfaitement illusoire.

Relevons encore, pour terminer, l'analogie que nous avons établie entre les *courbes de fréquence spécifique*, tant *locales* que *générales*, et les *courbes de variations organiques;* enfin les relations existant entre les cœfficients génériques des grandes subdivisions florales.

Vis-à-vis des variations infinies des conditions écologiques, variations entraînant une diversité correspondante dans la composition et la distribution du tapis végétal, les espèces dont l'association constitue la prairie réagissent non seulement *individuellement*, mais *collectivement*. Une pareille association se comporte comme une *unité organique ;* ses diverses espèces, de même que les divers organes d'un organisme quelconque, présentent entre elles une *corrélation* manifeste et *leurs proportions relatives, tant numé-*

rique que spécifique, révèlent une ordonnance qui n'apparaît point dans les variations du milieu où elles vivent.

Cette ordonnance créée par la concurrence qui s'établit entre les espèces associées est l'expression d'une véritable *vie sociale.*

Nous pouvons donc considérer la distribution des espèces végétales dans une formation déterminée comme résultant de l'action combinée de trois ordres de facteurs; 1° les *facteurs écologiques* (nature du sol et du climat), 2° les *facteurs biologiques*, exprimés par le degré d'adaptation des espèces à leur station et mieux encore par leur *pouvoir d'adaptation*, lequel est très inégal suivant les espèces; 3° les *facteurs sociologiques*, créés par la concurrence qui s'établit entre les espèces associées.

L'action des deux premiers facteurs a pour conséquence, dans chaque station, l'élimination d'un certain nombre d'espèces, d'où résulte une *sélection éliminatoire.*

Le troisième facteur détermine la distribution locale des espèces non éliminées, soit leur *sélection distributive.*

Cette dernière sélection étant à la fois numérique et taxinomique, il y a lieu de distinguer :

1° Une *sélection numérique*, d'où résulte le *nombre* et la *fréquence relative* des espèces ainsi que des individus associés.

2° Une *sélection taxinomique*, d'où résulte la proportion des espèces associées par rapport aux genres, ordres et classes auxquelles elles appartiennent.

Zurich, juin 1908.

TABLEAU N° 11. (Documents.)

Nombre des espèces correspondant aux fréquences 1 à 8 dans chaque m² des localités I à V et VIII.

Abréviat. : Fr. = Degrés de fréquence des espèces dans chaque localité.
E. = Nombre des espèces dans chaque m².
T = Total des nombres correspondant à chaque degré de fréquence dans les divers m².
M = moyenne = T. divisé par le nombre de m² de chaque localité.

m² I-8	Localité I Fr. 1	2	3	4	5	6	7	8	E
1	2	2	—	3	5	7	5	4	28
2	2	—	—	1	6	7	5	4	25
3	—	1	—	3	1	9	5	4	23
4	—	—	—	2	4	8	4	4	22
5	1	1	3	5	3	10	3	4	30
6	3	1	2	8	5	7	5	4	35
7	1	2	3	5	3	8	3	4	29
8	2	1	4	5	3	10	5	4	34
T=	11	8	12	32	30	66	35	32	= T/8
M=	1,4	1	1,5	4	3,8	8,3	4,4	4	

m² I-6	Localité II. Fr. 1	2	3	4	5	6	E
1	2	—	1	3	5	10	21
2	2	2	3	3	7	10	27
3	2	1	5	4	7	10	29
4	1	1	5	5	6	10	28
5	1	2	4	3	6	10	26
6	3	—	6	2	4	10	25
T=	11	6	24	20	35	60	= T/6
M=	1,8	1	4	3,3	6	10	

m² I-8	Localité III Fr. 1	2	3	4	5	6	7	8	E
1	2	2	3	3	2	1	7	6	26
2	—	1	2	4	2	3	10	6	28
3	1	1	—	5	2	5	8	6	28
4	—	1	—	4	—	5	10	6	26
5	—	1	1	3	—	5	9	6	25
6	—	—	1	3	—	4	11	6	25
7	2	1	1	2	2	3	11	6	28
8	2	1	1	4	2	4	11	6	31
T=	7	8	9	28	10	30	77	48	= T/8
M=	0,9	1	1,1	3,5	1,3	3,8	9,8	6	

m² I-6	Localité IV. Fr. 1	2	3	4	5	6	E
1	1	1	3	1	5	12	23
2	1	3	4	—	4	12	24
3	—	1	5	3	5	12	26
4	2	—	1	4	5	12	24
5	—	-	5	4	4	12	25
6	4	1	3	4	2	12	26
T=	8	6	21	16	25	72	= T/6
M=	1,3	1	3,5	2,8	4,2	12	

m² 1-6	Localité V Fr.						E
	1	2	3	4	5	6	
1	—	—	2	2	4	14	22
2	1	—	2	2	4	14	23
3	—	—	1	3	4	14	22
4	—	—	3	—	4	14	21
5	1	1	3	2	1	14	22
6	1	1	2	2	3	14	23
T=	3	2	13	11	20	84	$=\frac{T}{6}$
M=	0,5	0,3	2,1	1,9	3,3	14	

m² 1-7	Localité VIII. Fr.							E
	1	2	3	4	5	6	7	
1	1	1	—	2	4	6	11	25
2	1	1	—	1	3	6	11	23
3	—	2	1	1	4	4	11	23
4	1	1	—	1	3	5	11	22
5	—	3	2	2	3	4	11	25
6	1	1	2	2	1	5	11	23
7	—	3	1	3	2	6	11	26
T=	4	12	6	12	20	36	77	$=\frac{T}{7}$
M=	0,57	1 7	0,8	1,7	2,8	5,1	11	

M_3 et M_4 = Moyennes suivant 3 ou 4 degrés de fréquence obtenues en faisant la somme des moyennes de deux degrés de fréquence successifs, 1+2, 3+4, etc., et correspondant aux expressions rares (r), assez communes (a.c.), communes (c), ou simplement r., c. et. cc., lorsqu'on n'envisage que 3 degrés de fréquence seulement.

				T
Local I. M_4 = r = 2,4.	a.c. = 5,5.	c. = 12,1.	cc. = 8,4	= 28,4.
» III. M_4 = r = 1,9.	a.c. = 4,6.	c. = 5,1.	cc. = 15,8	= 27,4.
» II. M_3 = r = 2,8.	—	c. = 7,3.	cc. = 16,-	= 26,1.
» IV. M_3 = r = 2,3.	—	c. = 6,3.	cc. = 16,2	= 24,8.
» V. M_3 = r = 0,8.	—	c. = 4.	cc. = 17,3	= 22,1.
» VIII. M_4 = r[1] = 2,3.	a.c.[1] = 2,5.	c. = 4,5.	cc. = 16,1	= 25,4.[2]

[1] r = 1+2. ac. = 2+3.

[2] Ou, en déduisant Fr. 2 = 1,7 compté deux fois : 23.7.

TABLEAU N° 12 (Documents).

Coefficients de communauté entre les divers m² des localités I à IV.

Localité I. 53 esp.

		esp.d.[1]	esp.c.[2]	C.c.%
Entre les m²	1 et 2 :	33	20	60,6
	1 » 3 :	36	15	41,6
	1 » 4 :	32	17	50,3
	1 » 5 :	41	17	41,5
	1 » 6 :	44	19	43,2
	1 » 7 :	41	16	39.
	1 » 8 :	44	18	40,9
	2 » 3 :	33	15	45,4
	2 » 4 :	30	17	56,6
	2 » 5 :	39	16	43,6
	2 » 6 :	40	20	50.
	2 » 7 :	40	14	35.
	2 » 8 :	41	18	44.
	3 et 4 :	29	16	55.
	3 » 5 :	36	17	47,2
	3 » 6 :	40	18	45.
	3 » 7 :	36	16	44,4
	3 » 8 :	37	20	54.
	4 » 5 :	36	16	44,4
	4 » 6 :	40	17	42,5
	4 » 7 :	37	14	37,8
	4 » 8 :	39	17	43,6
	5 » 6 :	42	23	54,8
	5 » 7 :	41	18	44.
	5 » 8 :	41	23	56.
	6 » 7 :	43	21	48,8
	6 » 8 :	45	24	53,3
	7 et 8 :	41	22	53,7
				1 316,6

[1] Esp. d = espèces distinctes.

[2] Esp. c = espèces communes aux deux m² comparés.

1 316,6 : 28 = 47.

Localité II. 44 esp.

		esp.d.	esp.c.	C.c.%
Entre les m²	1 et 2 :	30	18	60.
	1 » 3 :	33	17	51,5
	1 » 4 :	32	17	53,1
	1 » 5 :	30	17	56,6
	1 » 6 :	34	12	35,3
	2 » 3 :	35	21	60.
	2 » 4 :	36	19	52.8
	2 » 5 :	34	19	55.9
	2 » 6 :	34	12	35.3
	3 » 4 :	35	22	62,8
	3 » 5 :	35	20	57,1
	3 » 6 :	34	20	58,8
	4 » 5 :	33	21	63,6
	4 » 6 :	34	19	55,9
	5 » 6 :	35	16	45,7
				804,4

804,4 : 15 = 53,6.
C. c. moyen = 53,6 = 54.

Localité IX. 25 esp. sur 2 m².
20 esp. com. : C. c. = 80 %.

Localité III. 45 esp.

		esp.d.	esp.c.	C.c.%
Entre les m²	1 et 2 :	34	20	59.
	1 » 3 :	40	14	35.
	1 » 4 :	38	14	37.
	1 » 5 :	38	13	34.
	1 » 6 :	35	16	46.
	1 » 7 :	35	19	54.
	1 » 8 :	40	17	43.
	2 » 3 :	35	21	60.
	2 » 4 :	33	21	64.
	2 » 5 :	36	17	47.
	2 » 6 :	35	18	51.
	2 » 7 :	36	20	56.
	2 » 8 :	37	22	59.
	3 » 4 :	32	22	67.
	3 » 5 :	32	21	65.
	3 » 6 :	33	20	61.
	3 » 7 :	37	19	51.
	3 » 8 :	37	22	59.
	4 et 5 :	30	21	70.
	4 » 6 :	30	21	70.
	4 » 7 :	35	19	54.
	4 » 8 :	34	22	65.
	5 » 6 :	30	20	67.
	5 » 7 :	35	18	51.
	5 » 8 :	36	20	56.
	6 » 7 :	32	21	65.
	6 » 8 :	33	23	70.
	7 » 8 :	35	24	66.
				1582

Coeff. de communauté moyen = 1582 = 28 = 56,5 soit 57 %.

Localité IV. 39 esp.

		esp.d.	esp.c.	C.c.%
Entre les m²	1 et 2 :	29	18	62.
	1 » 3 :	30	19	63.
	1 » 4 :	29	18	62.
	1 » 5 :	30	18	60.
	1 » 6 :	32	17	53.
	2 » 3 :	30	20	67.
	2 » 4 :	32	16	50.
	2 » 5 :	34	15	44.
	2 » 6 :	36	14	39.
	3 » 4 :	29	21	72.
	3 » 5 :	29	22	76.
	3 » 6 :	34	18	53.
	4 » 5 :	28	21	75.
	4 » 6 :	32	18	56.
	5 » 6 :	32	19	59.
				891

Coeffic. de communauté moyen 891 : 15 = 59,4 %.

Localité V. 29 esp.

		esp.d.	esp.c.	C.c.%
Entre les m²	1 et 2 :	25	20	80.
	1 » 3 :	23	21	91.
	1 » 4 :	24	19	79.
	1 » 5 :	27	17	63.
	1 » 6 :	27	18	67.
	2 » 3 :	24	21	88.
	2 » 4 :	26	18	69.
	2 » 5 :	27	18	67.
	2 » 6 :	27	19	70.

3 et 4 :	24	19	79.
3 » 5 :	27	17	63.
3 » 6 :	26	19	73.
4 » 5 :	26	17	65.
4 » 6 :	25	19	76.
5 » 6 :	27	18	67.
			1 097.

Coeffic. de communauté moyen = 1097 : 15 = 73 %.

Localité VI. 38 esp.

	esp.d.	esp.c.	C.c.%
Entre les m² 1 et 2 :	31	23	74.
1 » 3 :	32	19	59.
1 » 4 :	35	18	51.
2 » 3 :	32	23	72.
2 » 4 :	35	22	63.
3 » 4 :	31	23	74.
			393.

Coeffic. de communauté moyen = 393 : 6 = 65.5 % = 66 %.

Localité VII. 26 esp

	esp.d.	esp.c.	C.c.%
Entre les m² 1 et 2 :	24	17	71.
1 » 3 :	25	18	72.
1 » 4 :	24	17	71.
1 » 5 :	23	18	78.
2 » 3 :	24	18	75.
2 » 4 :	22	18	82.
2 » 5 :	23	17	74.
3 » 4 :	24	18	75.
3 » 5 :	23	19	83.
4 et 5 :	22	18	82.
			763.

Coeffic. de communauté moyen = 763 : 10 = 76 %.

Localité VIII. 36 esp.

	esp.d.	esp.c.	C.c.%
Entre les m² 1 et 2 :	27	21	78.
1 » 3 :	29	19	66.
1 » 4 :	27	20	74.
1 » 5 :	30	20	67.
1 » 6 :	30	18	60.
1 » 7 :	30	21	70.
2 » 3 :	27	19	70.
2 » 4 :	26	19	73.
2 » 5 :	31	17	55.
2 » 6 :	29	17	59.
2 » 7 :	31	18	58.
3 » 4 :	28	17	61.
3 » 5 :	31	17	55.
3 » 6 :	30	16	53.
3 » 7 :	30	19	63.
4 » 5 :	30	17	57.
4 » 6 :	30	15	50.
4 » 7 :	30	18	60.
5 » 6 :	29	19	66.
5 » 7 :	31	20	65.
6 » 7 :	29	20	69.
			1 329.

Coeffic. de communauté moyen 1329 : 21 = 63 %.

Graphique N° 1.

montrant la proportion relative des espèces rares, communes et très-communes, sur 52 m² de la prairie des Ormonts (Fréquence générale).

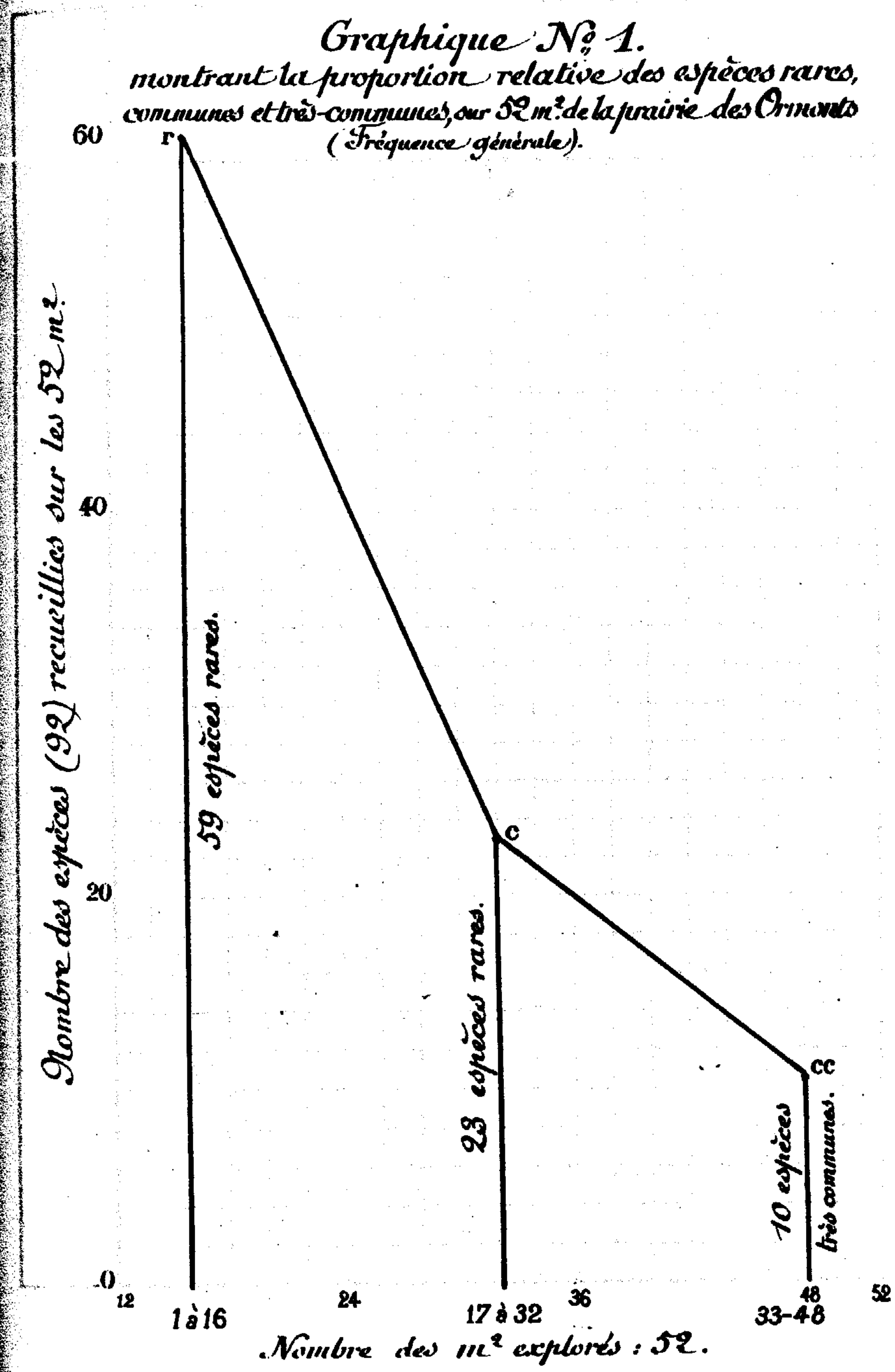

Graphique N° 2.
Fréquence locale des espèces pour les 8 m² de la localité I suivant 8 degrés.
M = Tracé moyen soit courbes de fréquence locale.

Graphique N° 3.
Fréquence locale pour les 8 m² de la localité III suivant 8 degrés.
M = Tracé moyen.

Graphiques N° 4, 5 et 6.
Fréquence locale pour les localités II, IV et V suivant 6 degrés.
M = Tracé moyen.

Graphique N° 7.
pour la localité VIII suivant 7 degrés.
M = Tracé moyen.

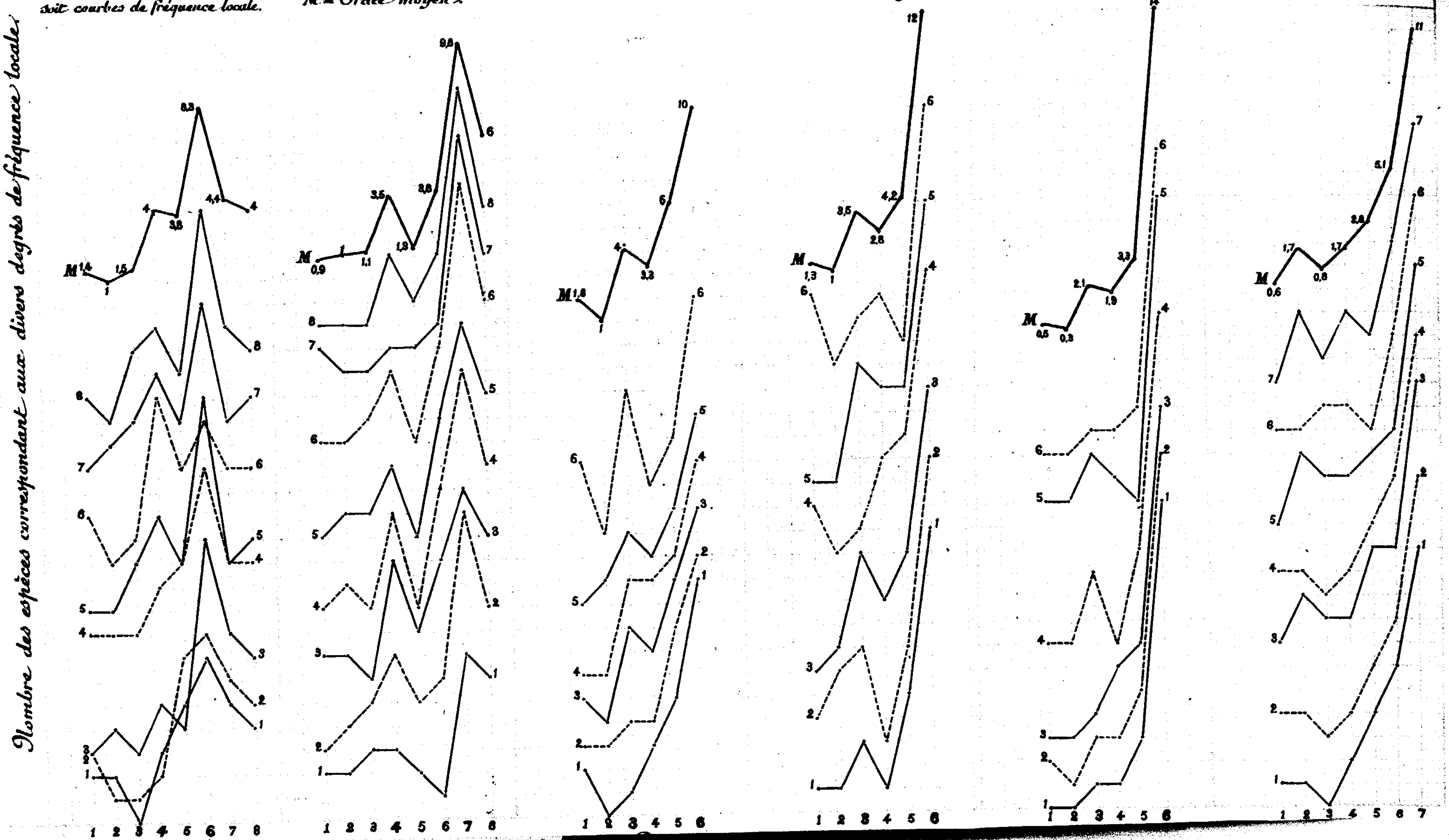

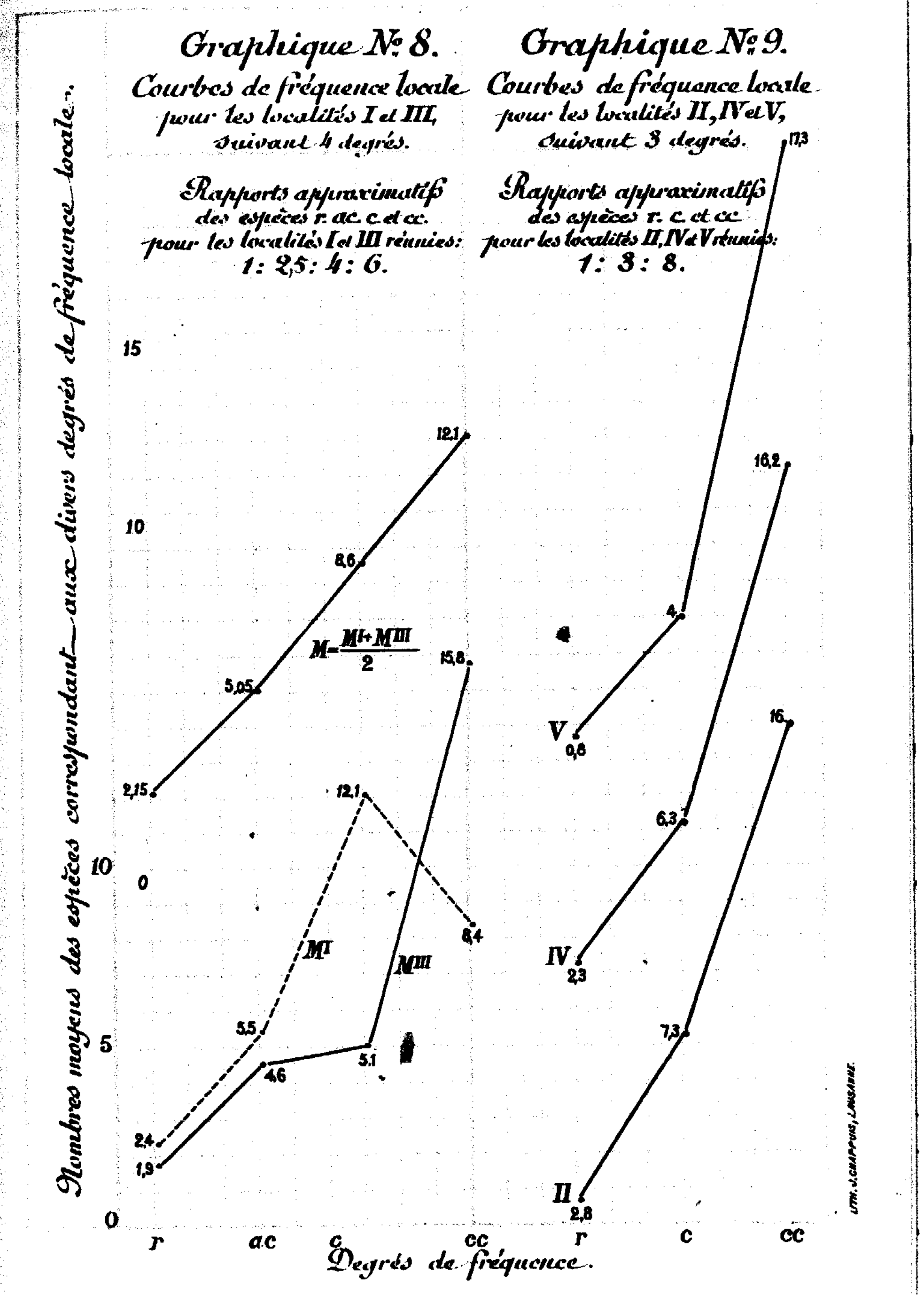
Graphique N° 8.
Courbes de fréquence locale pour les localités I et III, suivant 4 degrés.
Rapports approximatifs des espèces r. ac. c. et cc. pour les localités I et III réunies: 1: 2,5: 4: 6.
Graphique N° 9.
Courbes de fréquence locale pour les localités II, IV et V, suivant 3 degrés.
Rapports approximatifs des espèces r. c. et cc. pour les localités II, IV et V réunies: 1: 3: 8.
Nombres moyens des espèces correspondant aux divers degrés de fréquence locale.
Degrés de fréquence.
M = (MI + MIII) / 2
MI
MIII
V
IV
II
15
10
5
0
r
ac
c
cc
2,15
5,05
8,6
12,1
2,4
5,5
12,1
8,4
1,9
4,6
5,1
15,8
0,8
4
17,3
2,3
6,3
16,2
2,8
7,3
16
LITH. J. CHAPPUIS, LAUSANNE.

Graphique N° 10.

Courbes de fréquence élément^re et de fréquence locale suivant 16 et 8 degrés pour les localités I et III combinées.

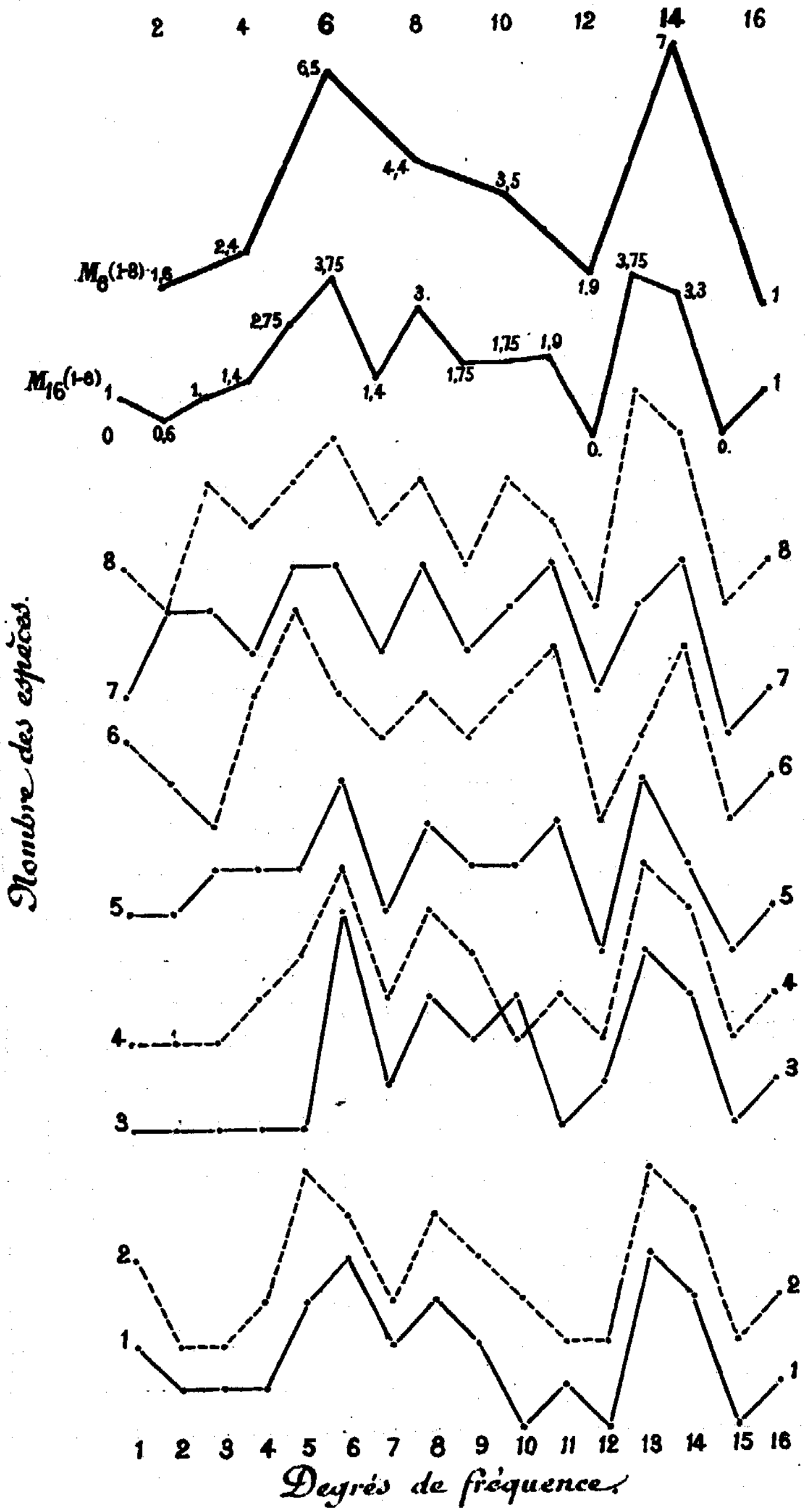

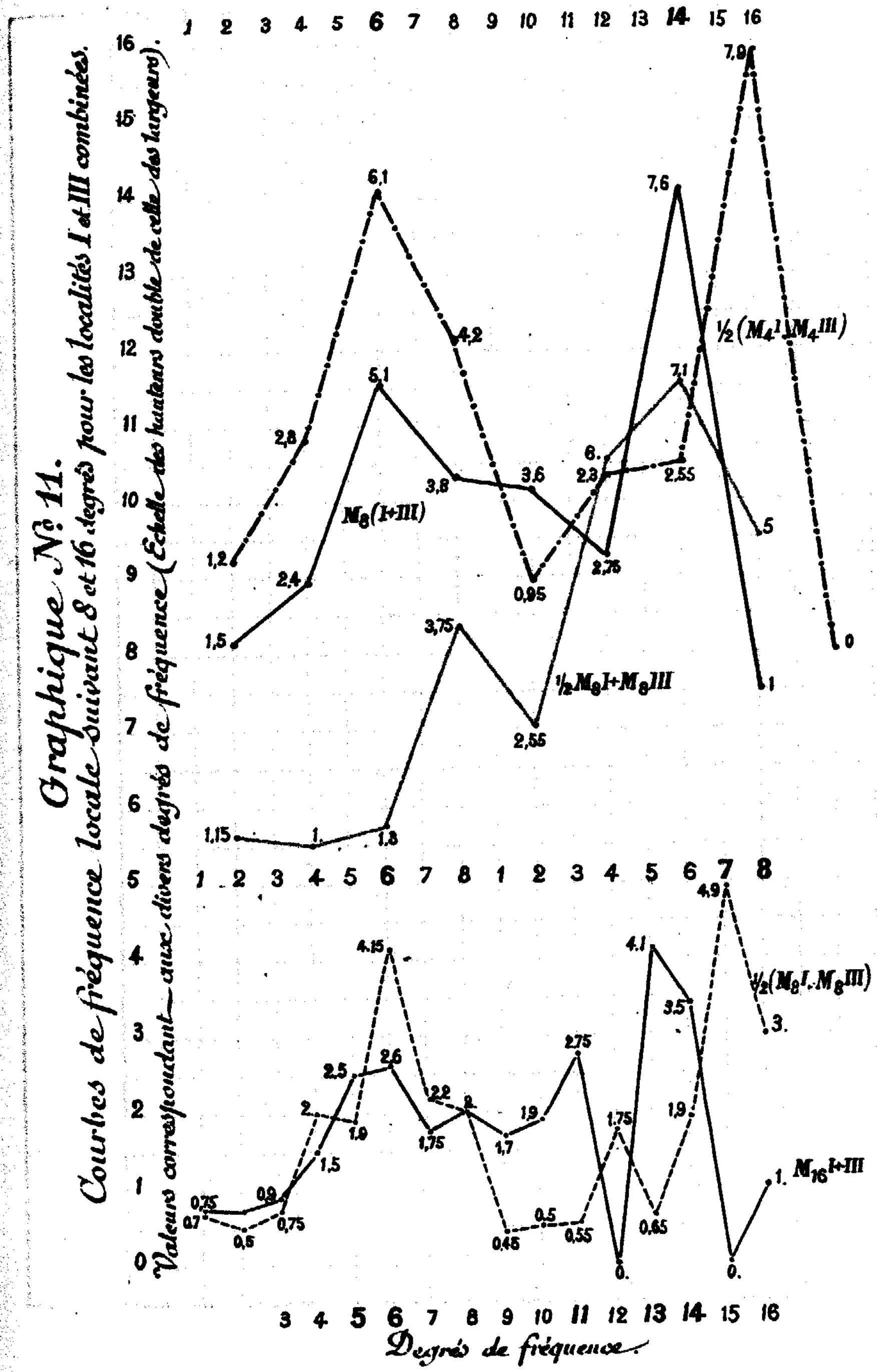
Graphique N° 11.
Courbes de fréquence locale suivant 8 et 16 degrés pour les localités I et III combinées.
Valeurs correspondant aux divers degrés de fréquence (Echelle des hauteurs double de celle des largeurs).
Degrés de fréquence.
M8(I+III)
½ M8I+M8III
½(M4I.M4III)
½(M8I.M8III)
M16 I+III

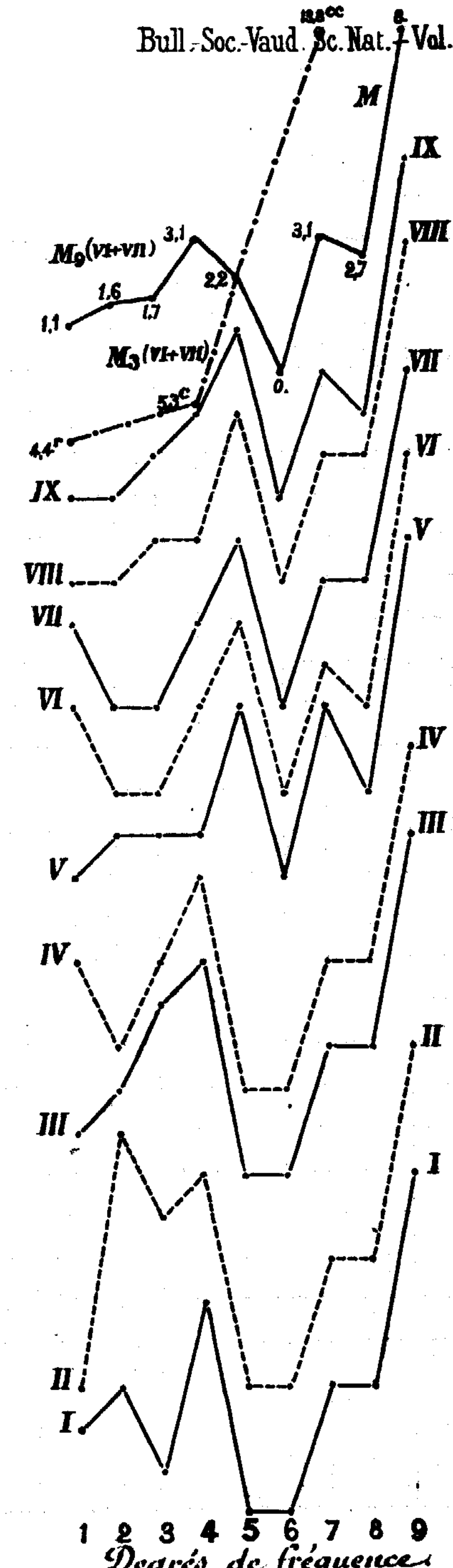

Graphique N° 12.

Courbes de fréquence élémentaire et de fréquence locale suivant 3 et 9 degrés pour les localités VI et VII combinées.

Nombre des espèces.

Graphique N° 15.

Courbes de fréquence locale concernant 12 localités du Jura méridional.
(Les tracés rouges des localités V et IX sont calculés pour 100 espèces).

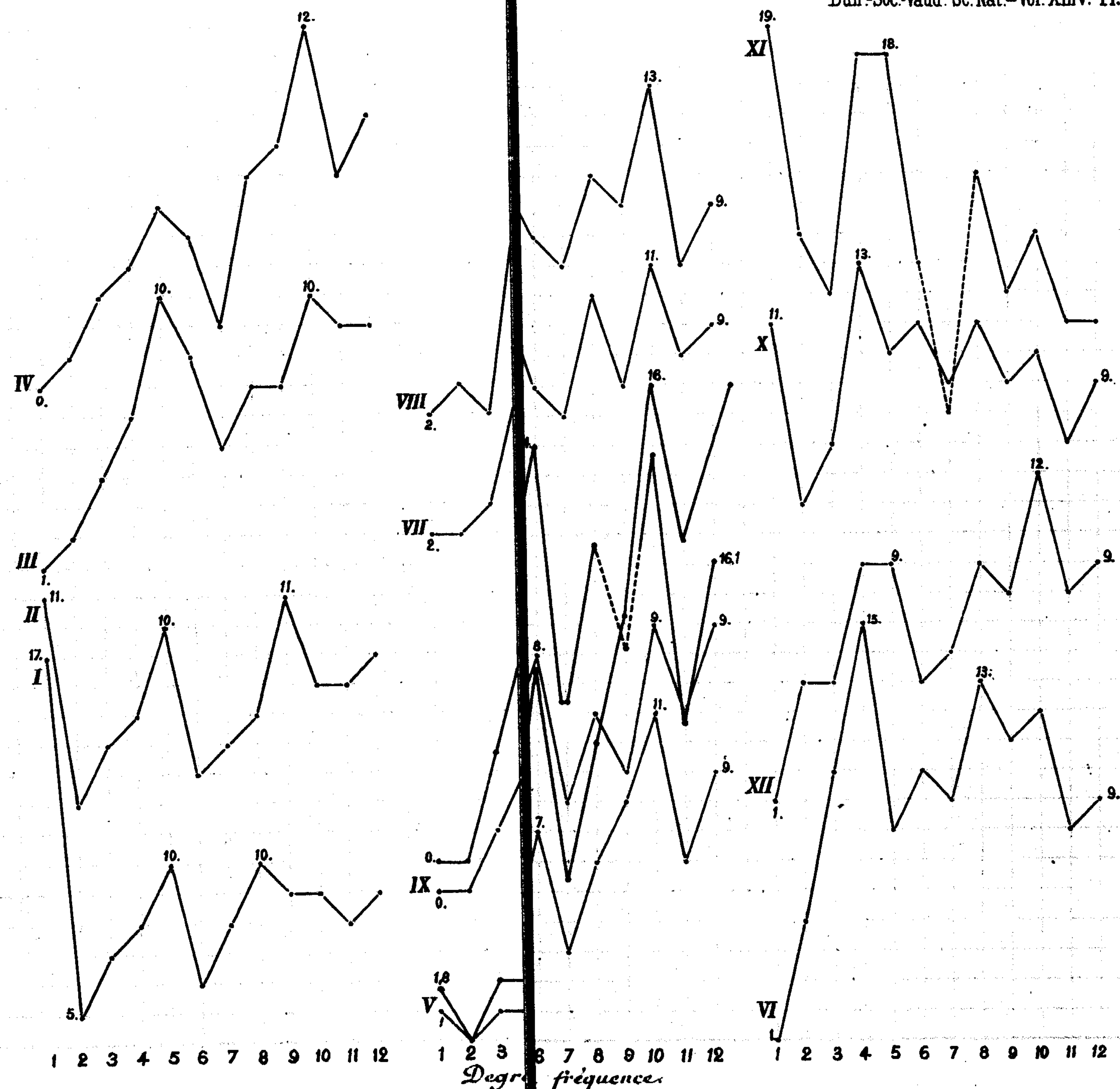

Graphique N° 14.

Fréquence générale relative suivant 6 degrés dans les localités I à IV.

M. Tracés moyens suivant 3 et 6 degrés.

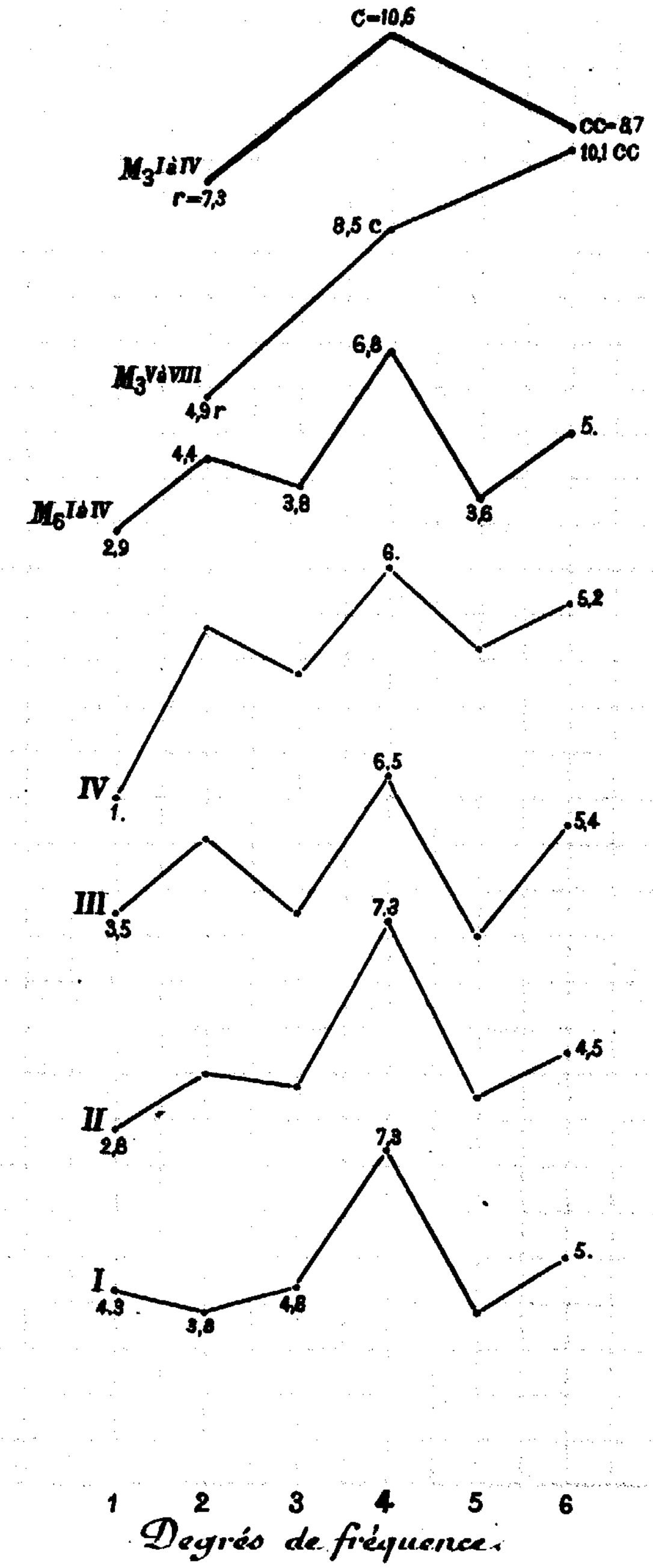

Supplement: Supplementary file 1 [file pgen.0020021.sd001.pdf]
