# Supplementary material for: Comparative Genomics of Emerging Human Ehrlichiosis Agents
Source: PLoS Genet. 2006 Feb 17;2(2):e21. doi: 10.1371/journal.pgen.0020021 (PMC1366493; doi:10.1371/journal.pgen.0020021)

Ehrlichia chaffeensis **Arkansas**

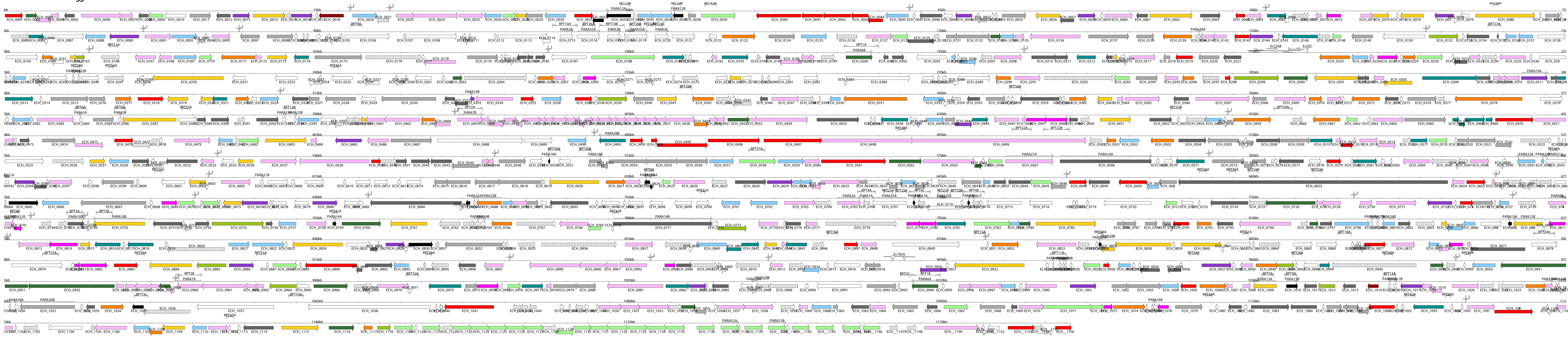

Anaplasma phagocytophilum **HZ**

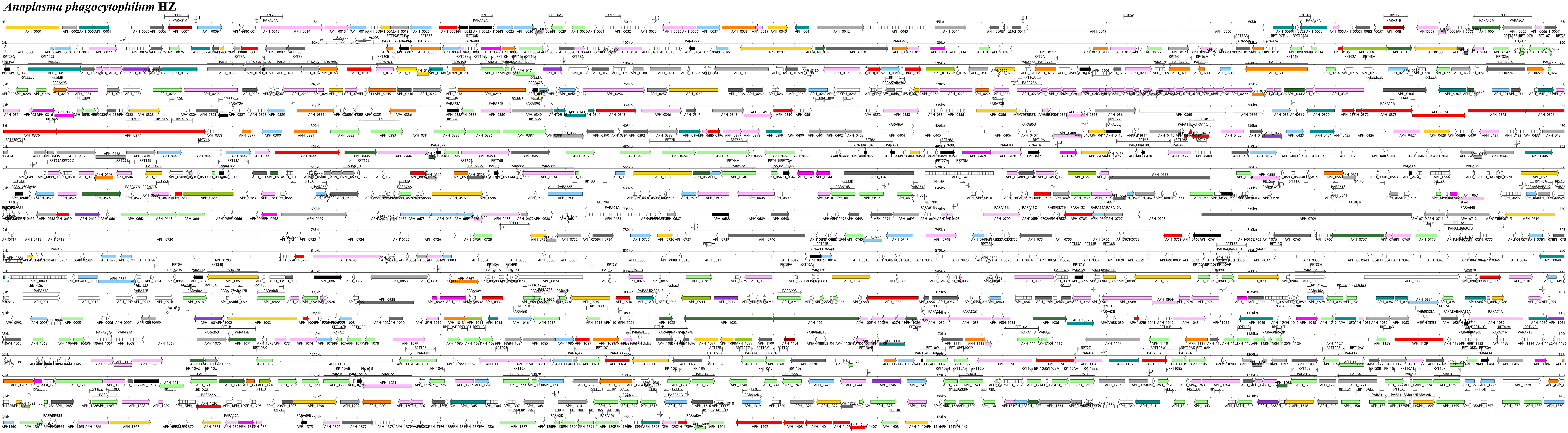

Neorickettsia sennetsu **Miyayama**

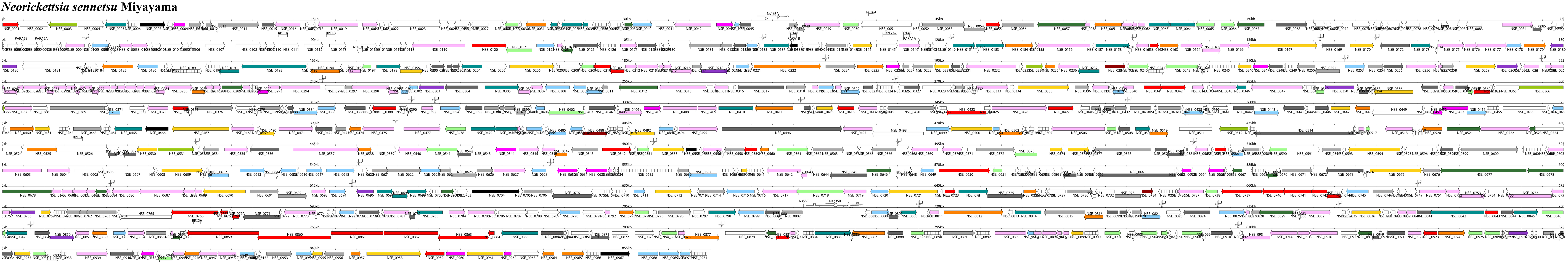

Supplement: Supplementary file 2 [file pgen.0020021.sg001.pdf]
