# Supplementary material for: Comparative Genomics of Emerging Human Ehrlichiosis Agents
Source: PLoS Genet. 2006 Feb 17;2(2):e21. doi: 10.1371/journal.pgen.0020021 (PMC1366493; doi:10.1371/journal.pgen.0020021)
Supplement: Supplementary file 5 [file pgen.0020021.sg004.pdf]

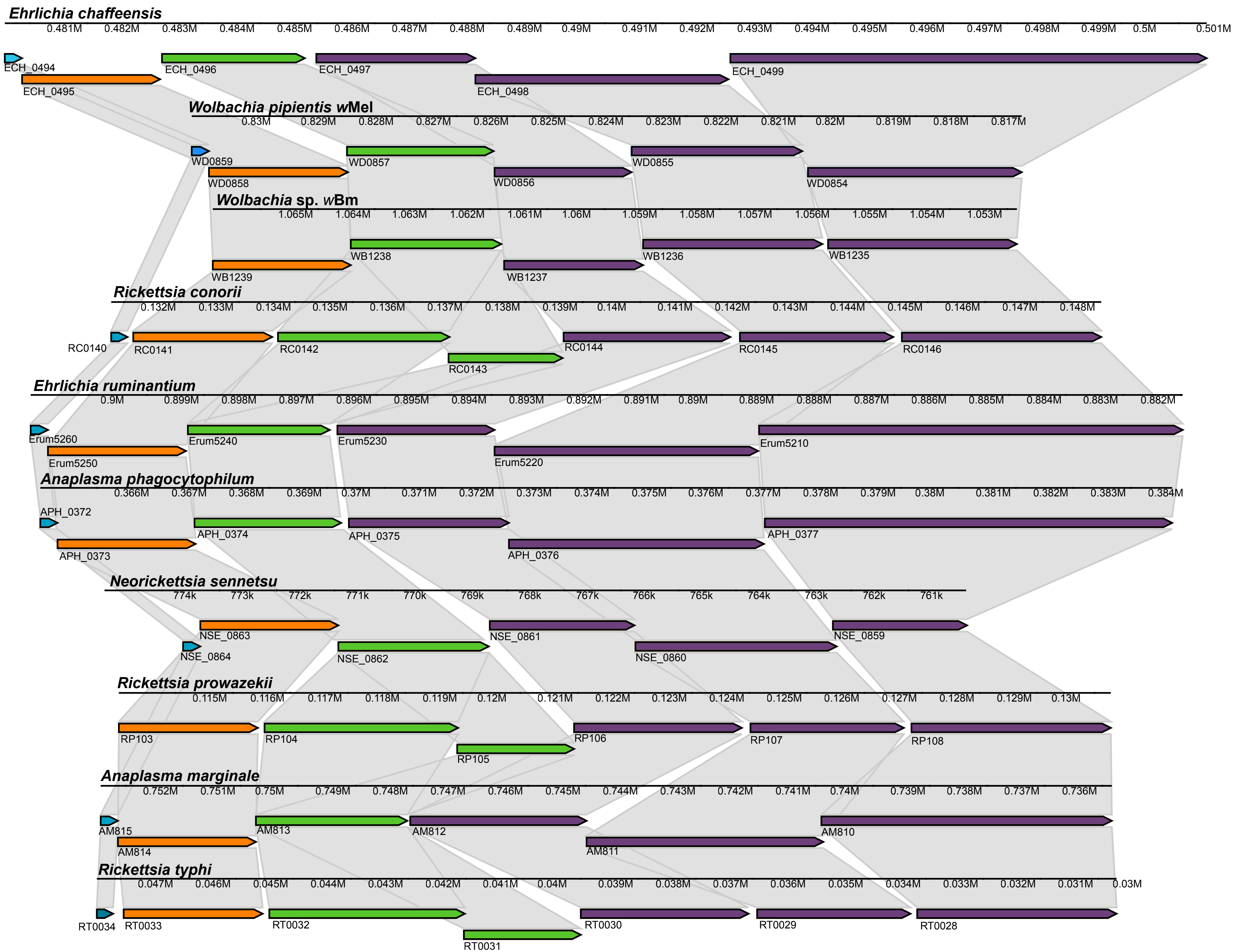

Ehrlichia chaffeensis
ECH_0494
ECH_0495
ECH_0496
ECH_0497
ECH_0498
ECH_0499
Wolbachia pipientis wMel
WD0859
WD0858
WD0857
WD0856
WD0855
WD0854
Wolbachia sp. wBm
WB1239
WB1238
WB1237
WB1236
WB1235
Rickettsia conorii
RC0140
RC0141
RC0142
RC0143
RC0144
RC0145
RC0146
Ehrlichia ruminantium
Erum5260
Erum5250
Erum5240
Erum5230
Erum5220
Erum5210
Anaplasma phagocytophilum
APH_0372
APH_0373
APH_0374
APH_0375
APH_0376
APH_0377
Neorickettsia sennetsu
NSE_0863
NSE_0864
NSE_0862
NSE_0861
NSE_0860
NSE_0859
Rickettsia prowazekii
RP103
RP104
RP105
RP106
RP107
RP108
Anaplasma marginale
AM815
AM814
AM813
AM812
AM811
AM810
Rickettsia typhi
RT0034
RT0033
RT0032
RT0031
RT0030
RT0029
RT0028
