# Supplementary material for: Comparative Genomics of Emerging Human Ehrlichiosis Agents
Source: PLoS Genet. 2006 Feb 17;2(2):e21. doi: 10.1371/journal.pgen.0020021 (PMC1366493; doi:10.1371/journal.pgen.0020021)
Supplement: Supplementary file 6 [file pgen.0020021.st001.doc]

**Supplementary Table 1.** Ortholog clusters (see methods) conserved across all representative obligate and facultative intracellular pathogens and endosymbionts presented in a tab-delimited format.

Cluster 1

Number of proteins: 151

Average blastp percent identity: 33%

Average blastp sequence coverage: 78%

AM023	ATP transporter ATP-binding protein (224 aa)

AM392	hypothetical protein (358 aa)

AM397	zinc uptake system ATP-binding protein (245 aa)

AM872	phosphate ABC transporter (274 aa)

AM1210	ribonucleotide transport ATP-binding protein (239 aa)

APH_0004	ABC transporter, ATP-binding protein (241 aa)

APH_0041	ABC transporter, ATP-binding protein (225 aa)

APH_0313	phosphate ABC transporter, ATP-binding protein (252 aa)

APH_0983	cation ABC transporter, ATP-binding protein, putative (220 aa)

APH_0986	ABC transporter, ATP-binding protein (342 aa)

BH00170	ABC transporter/ATP-binding protein (264 aa)

BH00870	Iron transport protein yfeB (276 aa)

BH01840	Sugar ABC transporter, ATP-binding protein (348 aa)

BH01910	ABC transporter, ATP-binding protein (250 aa)

BH02400	ABC transporter, ATP-binding protein (536 aa)

BH02460	Phosphate transport system ATP-binding protein (252 aa)

BH05520	ABC transporter, ATP-binding protein (625 aa)

BH06340	Amino-acid ABC transporter (257 aa)

BH08250	High-affinity branched-chain amino acid transport, ATP-binding protein (236 aa)

BH08260	High-affinity branched-chain amino acid transport, ATP-binding protein (260 aa)

BH08770	Lipoprotein releasing system ATP-binding protein lolD (226 aa)

BH10610	Glutamate /aspartate transport ATP-binding protein (248 aa)

BH11380	ABC transporter, ATP-binding protein (549 aa)

BH12140	ABC transporter, ATP-binding protein (260 aa)

BH12920	ABC transporter, ATP-binding protein (344 aa)

BH15170	ABC transporter, ATP-binding protein (620 aa)

BH15420	Cell division protein ftsE (219 aa)

BH15460	ABC transporter, ATP-binding protein (660 aa)

Bfl040	putative manganese transport system ATP-binding protein (218 aa)

Bfl042	probable ABC transport protein, ATP-binding component (241 aa)

Bfl395	ABC transporter ATP-binding subunit (235 aa)

Bfl511	sulphate transport ATP-binding protein CysA (350 aa)

BR0223	ABC transporter, ATP-binding protein (312 aa)

BR0157	ABC transporter, ATP-binding protein (277 aa)

BR0110	sulfate ABC transporter, ATP-binding protein, putative (359 aa)

BR0086	ABC transporter, ATP-binding protein (242 aa)

BR0006	ABC transporter, ATP-binding protein (541 aa)

BR2141	phosphate ABC transporter, ATP-binding protein (257 aa)

BR1997	cell division ABC transporter, ATP-binding protein FtsE (219 aa)

BR1956	amino acid ABC transporter, ATP-binding protein (271 aa)

BR1789	branched-chain amino acid ABC transporter, ATP-binding protein (440 aa)

BR1753	ABC transporter, ATP-binding protein (605 aa)

BR1456	ABC transporter, ATP-binding protein (549 aa)

BR1350	ABC transporter, ATP binding/permease protein (922 aa)

BR1020	ABC transporter, ATP-binding protein (285 aa)

BR0824	lipoprotein releasing system ATP-binding protein LolD (227 aa)

BR0745	amino acid ABC transporter, ATP-binding protein (249 aa)

BR0692	ABC transporter, ATP-binding protein (627 aa)

BRA1123	zinc ABC transporter, ATP-binding protein (298 aa)

BRA0395	branched-chain amino acid ABC transporter, ATP-binding protein (234 aa)

BRA0404	peptide ABC transporter, ATP-binding protein (317 aa)

BRA0405	peptide ABC transporter, ATP-binding protein (338 aa)

BRA0534	ABC transporter, ATP-binding protein (536 aa)

BRA0658	glycerol-3-phosphate ABC transporter, ATP-binding protein (351 aa)

BRA0678	iron compound ABC transporter, ATP-binding protein (252 aa)

BRA0701	iron compound ABC transporter, ATP-binding protein, putative (367 aa)

BRA0961	ABC transporter, ATP-binding protein (352 aa)

BU296	hypothetical ABC transporter ATP-binding protein ycfv (228 aa)

BU318	high-affinity zinc uptake system ATP-binding protein ZnuC (238 aa)

BU364	ABC transporter ATP-binding protein uup (596 aa)

CP0023	ABC transporter, ATP-binding protein (240 aa)

CP0064	peptide ABC transporter, ATP-binding protein (324 aa)

CP0065	peptide ABC transporter, ATP-binding protein (324 aa)

CP0412	ABC transporter, ATP-binding protein (259 aa)

CP0478	ABC transporter, ATP-binding protein (341 aa)

CP0513	ABC transporter, ATP-binding protein (226 aa)

CP0565	peptide ABC transporter, ATP-binding protein (262 aa)

CP0566	peptide ABC transporter, ATP-binding protein (284 aa)

CP0576	amino acid ABC transporter, ATP-binding protein (226 aa)

CP0753	ABC transporter, ATP-binding protein (530 aa)

CBU1000	lipoprotein ABC transporter, ATP-binding protein LolD (233 aa)

CBU0934	ABC transporter, ATP-binding protein (304 aa)

CBU0746	ABC transporter, ATP-binding protein (249 aa)

CBU0728	ABC transporter, ATP-binding protein (268 aa)

CBU0481	amino acid ABC transporter, ATP-binding protein (250 aa)

CBU0178	ABC transporter, ATP-binding protein (252 aa)

CBU0107	ABC transporter, ATP-binding protein (351 aa)

CBU1952	ABC transporter, ATP-binding protein (532 aa)

CBU1904	cell division ABC transporter, ATP-binding protein FtsE (217 aa)

CBU1857	peptide ABC transporter, ATP-binding protein (530 aa)

CBU1809	ABC transporter, ATP-binding protein (221 aa)

ECH_0085	ABC transporter, ATP-binding protein (238 aa)

ECH_0174	ABC transporter, ATP-binding protein (235 aa)

ECH_0444	phosphate ABC transporter, ATP-binding protein (253 aa)

ECH_0841	cation ABC transporter, ATP-binding protein, putative (242 aa)

ECH_0845	ABC transporter, ATP-binding protein (340 aa)

ERGA_CDS_00480	Putative ribonucleotide transport ATP-binding protein MKL (239 aa)

ERGA_CDS_01120	Lipoprotein releasing system (ABC transporter) ATP-binding protein lolD (249 aa)

ERGA_CDS_02550	Spermidine/putrescine transport ATP-binding protein potA (340 aa)

ERGA_CDS_02590	High-affinity zinc uptake system ATP-binding protein znuC (242 aa)

ERGA_CDS_05960	Phosphate import ATP-binding protein pstB (ABC phosphate transporter) (253 aa)

Erum0580	putative ABC transporter, ATP binding protein (239 aa)

Erum1190	lipoprotein releasing system ATP-binding protein LolD (228 aa)

Erum2550	putative ABC transporter, ATP-binding protein (340 aa)

Erum2590	putative ABC transporter, ATP-binding protein (242 aa)

Erum5760	putative phosphate ABC transporter, ATP-binding protein (253 aa)

NSE_0808	phosphate ABC transporter, ATP-binding protein, putative (255 aa)

NSE_0698	ABC transporter, ATP-binding protein (242 aa)

NSE_0480	ABC transporter, ATP-binding protein (337 aa)

NSE_0315	lipoprotein releasing system ATP-binding protein LolD, putative (224 aa)

RC0088	ABC transporter ATP-binding protein (555 aa)

RC0130	ribonucleotide ABC transporter ATP-binding protein (251 aa)

RC0658	ABC transporter ATP-binding protein (240 aa)

RC1077	ABC transporter ATP-binding protein (221 aa)

RC1187	ABC transporter ATP-binding protein Uup (593 aa)

RC1289	zinc ABC transporter ATP-binding protein (233 aa)

RC1341	glutamine ABC transporter ATP-binding protein (240 aa)

RP060	ABC TRANSPORTER ATP-BINDING PROTEIN (abcT1) (555 aa)

RP097	POSSIBLE RIBONUCLEOTIDE TRANSPORT ATP-BINDING PROTEIN MKL (mkl) (252 aa)

RP508	ABC TRANSPORTER ATP-BINDING PROTEIN (abcT2) (254 aa)

RP700	GLUTAMINE TRANSPORT ATP-BINDING PROTEIN GLNQ (glnQ1) (221 aa)

RP834	FERRIC TRANSPORT ATP-BINDING PROTEIN (afuC) (233 aa)

RP868	GLUTAMINE TRANSPORT ATP-BINDING PROTEIN GLNQ (glnQ2) (240 aa)

RT0040	ribonucleotide ABC transporter ATP-binding protein (252 aa)

RT0072	ABC transporter ATP-binding protein (555 aa)

RT0493	ABC transporter ATP-binding protein (240 aa)

RT0695	ABC transporter ATP-binding protein (221 aa)

RT0822	metal transport ATP-binding protein (233 aa)

RT0859	glutamine transport ATP-binding protein GlnQ (240 aa)

TW142	dipeptide transport system ATP-binding protein (542 aa)

TW175	ABC transporter ATP-binding protein (220 aa)

TW177	ABC transporter ATP-binding protein (346 aa)

TW199	cell division ATP-binding protein (918 aa)

TW352	phosphate ABC transporter ATP-binding protein (269 aa)

TW401	ABC transporter ATP-binding protein (556 aa)

TW557	branched-chain amino acid ABC transporter ATP-binding protein (233 aa)

TW558	branched-chain amino acid ABC transporter ATP-binding protein (270 aa)

TW584	ABC transporter ATP-binding protein (275 aa)

TW663	iron ABC transporter ATP-binding protein (276 aa)

ycfV	Transport and binding proteins (229 aa)

yebM	Transport and binding proteins (232 aa)

gltL	Transport and binding proteins (257 aa)

yadG	Cellular processes (302 aa)

yhbG	Transport and binding proteins (239 aa)

WD0990	ABC transporter, ATP-binding protein (233 aa)

WD0153	ABC transporter, ATP-binding protein (257 aa)

WD0371	phosphate ABC transporter, ATP-binding protein (252 aa)

WD0455	amino acid ABC transporter, ATP-binding protein, putative (213 aa)

WD0707	ABC transporter, ATP-binding protein (224 aa)

WD0938	cation ABC transporter, ATP-binding protein, putative (231 aa)

Wbm0023	ABC transporter, ATP-binding protein (229 aa)

Wbm0178	phosphate ABC transporter, ATP-binding protein (252 aa)

Wbm0618	ABC transporter, ATP-binding protein (236 aa)

Wbm0677	sugar ABC transporter, ATP-binding protein (258 aa)

Wbm0715	ZnuC protein (246 aa)

Cluster 2

Number of proteins: 114

Average blastp percent identity: 29%

Average blastp sequence coverage: 80%

AM112	hypothetical protein (422 aa)

AM171	bicyclomycin resistance protein (393 aa)

AM400	hypothetical protein (401 aa)

AM1235	1-acyl-sn-glycerol-3-phosphate acyltransferase (241 aa)

APH_1373	1-acyl-sn-glycerol-3-phosphate acyltransferase family protein (241 aa)

APH_0094	membrane protein, putative (391 aa)

APH_0164	bicyclomycin resistance protein (389 aa)

APH_0878	membrane protein, putative (419 aa)

BH00090	hypothetical protein (613 aa)

BH02120	Transport transmembrane protein (542 aa)

BH09770	Bicyclomycin resistance protein (415 aa)

BH10060	Transport transmembrane protein (475 aa)

BH10070	Transport transmembrane protein (396 aa)

BH12560	Transport protein (398 aa)

BH12710	Multidrug resistance protein vceB (523 aa)

BH16290	1-acyl-sn-glycerol-3-phosphate acyltransferase (268 aa)

Bfl066	1-acyl-glycerol-3-phosphate acyltransferase (244 aa)

Bfl240	putative MFS family transporter (394 aa)

BR0289	long-chain-fatty-acid--CoA ligase (554 aa)

BR2152	acyltransferase, putative (267 aa)

BR1994	1-acyl-sn-glycerol-3-phosphate acyltransferase, putative (260 aa)

BR1963	major facilitator family transporter (435 aa)

BR1731	propionate--CoA ligase (636 aa)

BR1401	drug resistance transporter, Bcr/CflA family (403 aa)

BR1059	drug resistance transporter, EmrB/QacA family (513 aa)

BR0833	drug resistance transporter, Bcr/CflA family (421 aa)

BRA0015	2,3-dihydroxybenzoate-AMP ligase (539 aa)

BRA0116	drug resistance transporter, Bcr/CflA family (529 aa)

BRA0143	phthalate transporter, putative (433 aa)

BRA0300	nitrite extrusion protein (895 aa)

BRA0311	transporter, putative (542 aa)

BRA0444	major facilitator family transporter (433 aa)

BU466	hypothetical protein (390 aa)

BU535	hypothetical protein (388 aa)

BU588	hypothetical protein (410 aa)

CP0944	Aas bifunctional protein, putative (538 aa)

CP0945	conserved hypothetical protein (253 aa)

CP0009	efflux protein, putative (569 aa)

CP0082	GlpT/PgpT/UhpT family protein (455 aa)

CP0180	1-acyl-sn-glycerol-3-phosphate acyltransferase, putative (212 aa)

CBU1345	major facilitator family transporter (429 aa)

CBU1179	drug resistance transporter, Bcr/CflA family (398 aa)

CBU0922	major facilitator family transporter (437 aa)

CBU0797	drug resistance transporter, EmrB/QacA family (526 aa)

CBU0566	major facilitator family transporter (421 aa)

CBU0515	transporter, putative (428 aa)

CBU0324	AMP-binding enzyme family protein (559 aa)

CBU0272	major facilitator family transporter (458 aa)

CBU0029	acyltransferase family protein (242 aa)

CBU2068	major facilitator family transporter (446 aa)

CBU1967	drug resistance transporter, Bcr/CflA family (409 aa)

CBU1928	acyltransferase family protein (853 aa)

CBU1896	membrane protein, putative (401 aa)

CBU1756	drug resistance transporter, Bcr/CflA family (393 aa)

ECH_0072	1-acyl-sn-glycerol-3-phosphate acyltransferase family protein (236 aa)

ECH_0230	membrane protein, putative (411 aa)

ECH_0318	drug resistance transporter, Bcr/CflA family (402 aa)

ECH_0838	conserved hypothetical protein (405 aa)

ERGA_CDS_00450	Possible 1-acyl-sn-glycerol-3-phosphate acyltransferase (241 aa)

ERGA_CDS_02610	Hypothetical protein (402 aa)

ERGA_CDS_07050	Bicyclomycin resistance protein (398 aa)

ERGA_CDS_07760	Bicyclomycin resistance protein (409 aa)

Erum0550	putative 1-acyl-sn-glycerol-3-phosphate acyltransferase (241 aa)

Erum2610	putative integral membrane protein (401 aa)

Erum6780	putative bicyclomycin resistance protein (398 aa)

Erum7450	putative integral membrane protein (409 aa)

NSE_0064	drug resistance transporter, Bcr/CflA family (397 aa)

NSE_0846	membrane protein, putative (399 aa)

NSE_0407	1-acyl-sn-glycerol-3-phosphate acyltransferase family protein (264 aa)

RC0082	glycerol-3-phosphate transporter (431 aa)

RC0200	multidrug resistance protein B (519 aa)

RC0512	proline/betaine transporter (418 aa)

RC0711	possible 1-acyl-sn-glycerol-3-phosphate acyltransferase [EC:2.3.1.51] (241 aa)

RC0923	similarity to bicyclomycin resistance protein (Bcr1) (122 aa)

RC0962	2-acylglycerophosphoethanolamine acyltransferase [EC:6.2.1.20] (1140 aa)

RC1075	bicyclomycin resistance protein (391 aa)

RP054	GLYCEROL-3-PHOSPHATE TRANSPORTER (glpT) (429 aa)

RP157	MULTIDRUG RESISTANCE PROTEIN B (emrB) (519 aa)

RP375	PROLINE/BETAINE TRANSPORTER (proP3) (406 aa)

RP469	POSSIBLE 1-ACYL-SN-GLYCEROL-3-PHOSPHATE ACYLTRANSFERASE (plsC) (243 aa)

RP603	BICYCLOMYCIN RESISTANCE PROTEIN (bcr1) (407 aa)

RP620	2-ACYLGLYCEROPHOSPHOETHANOLAMINE ACYLTRANSFERASE (aas) (1152 aa)

RP698	BICYCLOMYCIN RESISTANCE PROTEIN (bcr2) (391 aa)

RT0078	glycerol-3-phosphate transporter (429 aa)

RT0146	multidrug resistance protein B (519 aa)

RT0363	proline/betaine transporter ProP3 (406 aa)

RT0456	probable 1-acyl-sn-glycerol-3-phosphate O-acyltransferase (242 aa)

RT0591	bicyclomycin resistance protein (409 aa)

RT0611	bifunctional protein Aas (2-acylglycerophosphoethanolamine acyltransferase and Acyl-acyl carrier protein synthetase) (1152 aa)

RT0693	bicyclomycin resistance protein (391 aa)

TW086	putative drug efflux protein (377 aa)

TW111	O-succinylbenzoic acid-CoA ligase (428 aa)

TW197	multidrug efflux protein (390 aa)

TW214	1-acyl-sn-glycerol-3-phosphate acyltransferase (221 aa)

TW507	acyltransferase family protein (241 aa)

TW645	MFS family transporter (D-galactonate) (439 aa)

yajR	Transport and binding proteins (388 aa)

emrD	Cellular processes (388 aa)

plsC	Fatty acid and phospholipid metabolism (243 aa)

WD1033	permease, putative (409 aa)

WD1034	membrane protein, putative (406 aa)

WD1149	1-acyl-sn-glycerol-3-phosphate acyltransferase family protein (241 aa)

WD0056	major facilitator family transporter (395 aa)

WD0248	drug resistance transporter (387 aa)

WD0384	bicyclomycin resistance protein (400 aa)

WD0619	GlpT/PgpT/UhpT transporter family protein (396 aa)

Wbm0004	major facilitator family transporter (403 aa)

Wbm0201	bicyclomycin resistance protein (388 aa)

Wbm0359	drug resistance transporter (388 aa)

gbw.ORF00996	truncated major facilitator family transporter predicted from 860750-861016 between Wbm0661 and Wbm5080 (88 aa)

Wbm0733	1-acyl-sn-glycerol-3-phosphate acyltransferase family protein (237 aa)

Cluster 3

Number of proteins: 87

Average blastp percent identity: 30%

Average blastp sequence coverage: 70%

AM127	hypothetical protein (999 aa)

AM712	hypothetical protein (3492 aa)

AM742	hypothetical protein (458 aa)

AM1063	major surface protein 3 (943 aa)

AM1108	hypothetical protein (597 aa)

AM091	msp3 pseudogene 7 (733 aa)

AM078	msp3 pseudogene 3 (582 aa)

AM718	msp3 pseudogene 6 (612 aa)

AM1148	msp3 pseudogene truncated 5 end (235 aa)

APH_0110	acetamidase/formamidase family protein (999 aa)

APH_0441	hypothetical protein (597 aa)

APH_1170	pentapeptide repeat family protein (564 aa)

BH00460	hypothetical protein (1548 aa)

BH01620	Filament-A percursor (426 aa)

BH05430	expressed protein (789 aa)

BH07480	hypothetical protein (1520 aa)

BH13620	hypothetical protein (274 aa)

Bfl090	putative exported protein (1286 aa)

Bfl129	organic solvent tolerance protein precursor (818 aa)

Bfl337	tolA protein (434 aa)

BR0497	SMC family protein (1152 aa)

BR0049	conserved hypothetical protein (1515 aa)

BR1838	hypothetical protein (449 aa)

BR1024	conserved hypothetical protein (1557 aa)

BR0925	conserved hypothetical protein (1146 aa)

BR0685	organic solvent tolerance, putative (792 aa)

BU079	flagellar hook-length control protein (235 aa)

BU087	hypothetical protein (970 aa)

CP0163	hypothetical protein (651 aa)

CP0581	hypothetical protein (390 aa)

CBU0918	conserved domain protein (589 aa)

CBU0540	SMC family protein (1169 aa)

CBU0197	conserved hypothetical protein (929 aa)

CBU1978	organic solvent tolerance protein (870 aa)

CBU1627	IcmE protein (1034 aa)

120 kDa immunodominant surface protein (388 aa)

ECH_0264	conserved hypothetical protein (995 aa)

ECH_0558	lipoprotein, putative (583 aa)

ECH_1083	pentapeptide repeat protein (607 aa)

ERGA_CDS_04860	Conserved hypothetical protein (572 aa)

ERGA_CDS_07550	Conserved hypothetical protein (1010 aa)

ERGA_CDS_08730	Conserved hypothetical protein (606 aa)

Erum4740	putative exported protein (639 aa)

Erum7250	putative membrane protein (999 aa)

Erum8330	conserved hypothetical protein (609 aa)

NSE_0181	hypothetical protein (943 aa)

NSE_0725	pentapeptide repeat domain protein (568 aa)

NSE_0591	hypothetical protein (490 aa)

RC0019	cell surface antigen (1902 aa)

RC0667	cell surface antigen (1026 aa)

RC0808	unknown (957 aa)

RC0850	unknown (589 aa)

RC1024	organic solvent tolerance protein homolog (713 aa)

RC1188	unknown (365 aa)

RC1213	unknown (836 aa)

RP018	190 KD ANTIGEN PRECURSOR (sca1) (347 aa)

RP278	unknown (371 aa)

RP498	CELL SURFACE ANTIGEN (sca4) (785 aa)

RP563	unknown (588 aa)

RP674	unknown (711 aa)

RP780	unknown (835 aa)

RT0015	190 kDa antigen precursor (1130 aa)

RT0269	rickettsial conserved hypothetical protein (380 aa)

RT0485	cell surface antigen (1019 aa)

RT0551	rickettsial conserved hypothetical protein (589 aa)

RT0668	probable organic solvent tolerance protein OstA (711 aa)

RT0767	rickettsial conserved hypothetical protein (837 aa)

TW418	unknown (574 aa)

imp	Cellular processes (723 aa)

fliK	Cellular processes (411 aa)

tolA	Cellular processes (251 aa)

ytfN	Hypothetical proteins (749 aa)

WD1039	hypothetical protein (474 aa)

WD0073	ankyrin repeat domain protein (800 aa)

WD0117	hypothetical protein (142 aa)

WD0305	hypothetical protein (1040 aa)

WD0335	hypothetical protein (825 aa)

WD0424	hypothetical protein (792 aa)

WD0440	pentapeptide repeat domain protein (601 aa)

WD0462	hypothetical protein (440 aa)

Wbm0006	conserved hypothetical protein (381 aa)

Wbm0304	conserved hypothetical protein (983 aa)

Wbm0362	conserved hypothetical protein (885 aa)

Wbm0581	pentapeptide repeat domain protein (606 aa)

Wbm0781	conserved hypothetical protein (204 aa)

gbw.ORF01208	ATPase, AAA family predicted from 1040555-1040704 between Wbm0786 and Wbm0787 (49 aa)

Cluster 4

Number of proteins: 56

Average blastp percent identity: 38%

Average blastp sequence coverage: 87%

AM487	hypothetical protein (343 aa)

AM524	tRNA pseudouridine 55 synthase (306 aa)

AM1289	2-octaprenyl-3-methyl-6-methoxy-1,4-benzoquinol hydroxylase (388 aa)

APH_0009	ubiquinone biosynthesis hydroxylase, UbiH/UbiF/VisC/COQ6 family (392 aa)

APH_0566	conserved hypothetical protein (326 aa)

APH_0769	tRNA pseudouridine synthase B (300 aa)

BH02130	tRNA pseudouridine synthase B (319 aa)

BH08540	2-octaprenyl-6-methoxyphenol hydroxylase (430 aa)

BH11810	hypothetical protein (359 aa)

BH15520	Oxidoreductase (426 aa)

Bfl106	tRNA pseudouridine 5S synthase (255 aa)

Bfl259	2-octaprenyl-6-methoxyphenol hydroxylase (394 aa)

Bfl318	putative monooxygenase (397 aa)

BR2167	tRNA pseudouridine synthase B (324 aa)

BR1899	UbiH/COQ6 monooxygenase family (414 aa)

BR1529	conserved hypothetical protein (365 aa)

BRA0573	2-octaprenyl-6-methoxyphenol hydroxylase (452 aa)

BU375	tRNA pseudouridine 55 synthase (312 aa)

CP0438	tRNA pseudouridine synthase B (235 aa)

CBU1430	tRNA pseudouridine synthase B (309 aa)

CBU0076	visC protein (411 aa)

CBU0075	2-octaprenyl-6-methoxyphenol hydroxylase (410 aa)

CBU1828	conserved hypothetical protein (388 aa)

ECH_0020	ubiquinone biosynthesis hydroxylase, UbiH/UbiF/VisC/COQ6 family (390 aa)

ECH_0728	tRNA pseudouridine synthase B (296 aa)

ECH_0762	conserved hypothetical protein (335 aa)

ERGA_CDS_03330	Conserved hypothetical protein (367 aa)

ERGA_CDS_03550	tRNA pseudouridine synthase B (296 aa)

ERGA_CDS_09450	Conserved hypothetical protein. Belongs to ubiH/COQ6 family (390 aa)

Erum0080	putative 2-octaprenyl-3-methyl-6-methoxy-1,4-benzoquinol hydroxylase (390 aa)

Erum3300	conserved hypothetical protein (339 aa)

Erum3520	putative tRNA pseudouridine synthase B (296 aa)

NSE_0101	tRNA pseudouridine synthase B (235 aa)

NSE_0601	conserved hypothetical protein (323 aa)

NSE_0384	ubiquinone biosynthesis hydroxylase, UbiH/UbiF/VisC/COQ6 family (377 aa)

RC0071	unknown (406 aa)

RC0665	tRNA pseudouridine 55 synthase (345 aa)

RC0848	UbiH protein [EC:1.14.13.-] (431 aa)

RP045	unknown (358 aa)

RP501	TRNA PSEUDOURIDINE 55 SYNTHASE (truB) (172 aa)

RP561	unknown (302 aa)

RT0085	conserved hypothetical protein (358 aa)

RT0487	tRNA pseudouridylate synthase B (292 aa)

RT0548	monooxygenase (384 aa)

TW087	tRNA pseudouridine synthase B (377 aa)

truB	Protein synthesis (327 aa)

ubiH	Biosynthesis of cofactors, prosthetic groups, and carrier (395 aa)

yleB	Unknown function (393 aa)

WD0177	monoxygenase family protein (382 aa)

WD0717	conserved hypothetical protein (349 aa)

WD0904	hypothetical protein (86 aa)

gbw.ORF00032	conserved hypothetical protein predicted from 28479-28634 between Wbm0020 and Wbm0021 (51 aa)

gbw.ORF00035	tRNA pseudouridine synthase B predicted from 29140-29358 between Wbm0020 and Wbm0021 (72 aa)

Wbm0064	2-polyprenyl-6-methoxyphenol 4-hydroxylase (385 aa)

Wbm0452	ATP synthase beta subunit/transription termination factor rho (409 aa)

Cluster 5

Number of proteins: 50

Average blastp percent identity: 33%

Average blastp sequence coverage: 74%

AM320	hypothetical protein (437 aa)

AM733	membrane protein (237 aa)

AM1303	hypothetical protein (293 aa)

APH_0021	hemolysin, putative (289 aa)

APH_0449	integral membrane protein, TerC family (236 aa)

APH_0957	CBS/transporter associated domain protein (422 aa)

BH02230	Hemolysin (339 aa)

BH10860	hypothetical protein (521 aa)

Bfl315	haemolysin-related protein (291 aa)

Bfl444	putative membrane protein (519 aa)

BR2157	CBS domain protein (342 aa)

BR2027	CBS domain protein (435 aa)

BR1721	conserved hypothetical protein (247 aa)

BR1332	CBS domain protein (518 aa)

BU323	hypothetical protein (521 aa)

BU443	hypothetical protein (291 aa)

CP0244	hemolysin, putative (387 aa)

CP0361	conserved hypothetical protein (411 aa)

CBU0565	CBS domain protein (279 aa)

CBU0452	conserved hypothetical protein (417 aa)

ECH_0031	hemolysin (295 aa)

ECH_0566	membrane protein, TerC family (228 aa)

ECH_0892	CBS domain protein (433 aa)

ERGA_CDS_00060	Conserved hypothetical protein (290 aa)

ERGA_CDS_02040	Conserved hypothetical protein (431 aa)

ERGA_CDS_04790	Conserved hypothetical protein (235 aa)

Erum0190	putative magnesium and cobalt efflux protein (288 aa)

Erum2070	putative integral membrane protein (431 aa)

Erum4670	conserved hypothetical integral membrane protein (235 aa)

NSE_0794	membrane protein, TerC family (224 aa)

NSE_0404	CBS domain protein (430 aa)

NSE_0327	CBS/transporter associated domain protein (252 aa)

RC1079	hemolysin homolog (424 aa)

RC1141	hemolysin C (299 aa)

RC1219	tellurium resistance protein TerC (301 aa)

RP702	unknown (424 aa)

RP740	HEMOLYSIN C (tlyC) (303 aa)

RP786	TELLURIUM RESISTANCE PROTEIN TERC (terC) (297 aa)

RT0697	conserved hypothetical protein (424 aa)

RT0725	hemolysin C (305 aa)

RT0774	tellurium resistance protein (302 aa)

TW509	tellurium resistence protein TerC-like protein (317 aa)

yoaE	Unknown function (424 aa)

WD1027	CBS domain protein (272 aa)

WD0194	membrane protein, TerC family (235 aa)

WD0558	CBS domain protein (427 aa)

Wbm0070	CBS domain protein (428 aa)

Wbm0442	membrane protein, TerC family (238 aa)

Wbm0809	CBS domain protein, putative (237 aa)

Cluster 6

Number of proteins: 43

Average blastp percent identity: 39%

Average blastp sequence coverage: 74%

AM1013	glucosamine--fructose-6-phosphate aminotransferase (608 aa)

AM1276	hypothetical protein (233 aa)

AM1338	amidophosphoribosyltransferase (469 aa)

APH_1300	amidophosphoribosyltransferase (468 aa)

APH_1345	membrane protein, putative (233 aa)

BH00080	hypothetical protein (257 aa)

BH05240	Amidophosphoribosyltransferase precursor (508 aa)

BH09920	Glucosamine-fructose-6-phosphate aminotransferase (607 aa)

Bfl343	putative permease (236 aa)

BR0446	amidophosphoribosyltransferase (485 aa)

BR0088	membrane protein, putative (245 aa)

BRA0582	glucosamine--fructose-6-phosphate aminotransferase (isomerizing) (607 aa)

BU026	D-fructose-6-phosphate amidotransferase (621 aa)

CP0888	conserved hypothetical protein (238 aa)

CP0890	hypothetical protein (106 aa)

CP0892	glucosamine--fructose-6-phosphate aminotransferase (isomerizing) (609 aa)

CBU0897	amidophosphoribosyltransferase (506 aa)

CBU0214	membrane protein, putative (239 aa)

CBU1787	glucosamine--fructose-6-phosphate aminotransferase, isomerizing (611 aa)

ECH_0139	amidophosphoribosyltransferase (462 aa)

ECH_0009	membrane protein, putative (233 aa)

ERGA_CDS_00830	Amidophosphoribosyltransferase precursor (466 aa)

ERGA_CDS_09350	Conserved hypothetical protein (234 aa)

Erum0900	glutamine phosphoribosylpyrophosphate amidotransferase (466 aa)

Erum8920	putative integral membrane protein (234 aa)

NSE_0194	amidophosphoribosyltransferase (463 aa)

NSE_0050	membrane proetin, putative (251 aa)

RC0189	unknown (236 aa)

RP147	unknown (236 aa)

RT0136	conserved hypothetical protein (integral membrane protein) (236 aa)

TW606	glucosamine--fructose-6-phosphate aminotransferase (616 aa)

TW723	amidophosphoribosyltransferase (504 aa)

glmS	Central intermediary metabolism (612 aa)

purF	Purines, pyrimidines, nucleosides, and nucleotides (470 aa)

yccA	Cell envelope (219 aa)

WD0950	membrane protein, putative (240 aa)

WD1109	amidophosphoribosyltransferase (461 aa)

WD0535	glucosamine--fructose-6-phosphate aminotransferase (isomerizing) (606 aa)

Wbm0223	glucosamine--fructose-6-phosphate aminotransferase, isomerizing (606 aa)

Wbm0255	amidophosphoribosyltransferase (461 aa)

Wbm0490	membrane protein (240 aa)

Cluster 7

Number of proteins: 43

Average blastp percent identity: 31%

Average blastp sequence coverage: 78%

AM684	isoleucine-tRNA ligase (1101 aa)

AM1170	valyl-tRNA synthetase (823 aa)

APH_1224	valyl-tRNA synthetase (806 aa)

APH_0796	isoleucyl-tRNA synthetase (1114 aa)

APH_0249.1	valyl-tRNA synthetase, truncation (92 aa)

BH02580	Isoleucyl-tRNA synthetase (971 aa)

BH10630	Valyl-tRNA synthetase (907 aa)

Bfl033	valyl-tRNA synthetase (966 aa)

Bfl118	isoleucyl-tRNA synthetase (953 aa)

BR0948	valyl-tRNA synthetase (910 aa)

BRA0202	isoleucyl-tRNA synthetase (972 aa)

BU149	isoleucyl-tRNA synthetase (940 aa)

BU366	valyl-tRNA synthetase (955 aa)

CP0665	isoleucyl-tRNA synthetase (1043 aa)

CP0680	valyl-tRNA synthetase (940 aa)

CBU0808	valyl-tRNA synthetase (920 aa)

CBU0396	isoleucyl-tRNA synthetase (936 aa)

ECH_0136	valyl-tRNA synthetase (808 aa)

ECH_0538	isoleucyl-tRNA synthetase (1122 aa)

ERGA_CDS_00680	Valyl-tRNA synthetase (812 aa)

ERGA_CDS_00690	Hypothetical protein (95 aa)

ERGA_CDS_00700	Hypothetical protein (95 aa)

ERGA_CDS_05000	Isoleucyl-tRNA synthetase (1125 aa)

Erum0780	valyl-tRNA synthetase (810 aa)

Erum4870	isoleucyl-tRNA synthetase (1104 aa)

NSE_0603	valyl-tRNA synthetase, putative (877 aa)

NSE_0537	isoleucyl-tRNA synthetase (1045 aa)

RC0953	isoleucyl-tRNA synthetase [EC:6.1.1.5] (1106 aa)

RC1053	valyl-tRNA synthetase [EC:6.1.1.9] (812 aa)

RP617	PROBABLE ISOLEUCYL-TRNA SYNTHETASE (ileS) (1086 aa)

RP687	VALYL-TRNA SYNTHETASE (valS) (814 aa)

RT0606	isoleucine--tRNA ligase (1086 aa)

RT0682	valine--tRNA ligase (814 aa)

TW477	isoleucyl-tRNA synthetase (1066 aa)

TW478	valyl-tRNA synthetase (856 aa)

ileS	Protein synthesis (940 aa)

valS	Protein synthesis (881 aa)

WD0423	isoleucyl-tRNA synthetase (1111 aa)

WD0464	valyl-tRNA synthetase (864 aa)

Wbm0363	isoleucyl-tRNA synthetase (1134 aa)

Wbm0786	valyl-tRNA synthetase (857 aa)

Cluster 8

Number of proteins: 40

Average blastp percent identity: 41%

Average blastp sequence coverage: 70%

AM824	propionyl-CoA carboxylase alpha chain precursor (662 aa)

AM933	carbamyl-phosphate synthase, large subunit (1102 aa)

APH_0249	carbamoyl-phosphate synthase, large subunit (1086 aa)

APH_0364	propionyl-CoA carboxylase, alpha subunit (659 aa)

BH08050	Biotin carboxylase (452 aa)

BH11620	Carbamoyl-phosphate synthase large chain (1161 aa)

Bfl123	carbamoyl-phosphate synthase large chain (1088 aa)

Bfl291	acetyl CoA carboxylase, biotin carboxylase subunit (450 aa)

BR0018	biotin carboxylase (673 aa)

BR1781	pyruvate carboxylase (1158 aa)

BR1488	carbamoyl-phosphate synthase, large subunit (1162 aa)

BR1189	propionyl-CoA carboxylase, alpha subunit (667 aa)

BR0906	acetyl-CoA carboxylase, biotin carboxylase (452 aa)

BU144	carbamoyl-phosphate synthase large chain (1079 aa)

CP0586	acetyl-coenzyme A carboxylase, biotin carboxylase (454 aa)

CBU1281	carbamoyl-phosphate synthase, large subunit (1073 aa)

CBU0977	biotin carboxylase/biotin carboxyl carrier protein (661 aa)

CBU1726	acetyl-CoA carboxylase, biotin carboxylase (448 aa)

ECH_0378	carbamoyl-phosphate synthase, large subunit (1076 aa)

ECH_0487	propionyl-CoA carboxylase, alpha subunit (655 aa)

ERGA_CDS_05470	Propionyl-CoA carboxylase alpha chain (660 aa)

ERGA_CDS_06530	Carbamoyl-phosphate synthase large chain (1075 aa)

Erum5320	putative acetyl-/propionyl-coenzyme A carboxylase alpha chain (660 aa)

Erum6310	carbamoyl-phosphate synthase, large subunit (1075 aa)

NSE_0222	carbamoyl-phosphate synthase, large subunit (1073 aa)

NSE_0815	propionyl-CoA carboxylase, alpha subunit (652 aa)

RC0959	propionyl-CoA carboxylase alpha chain precursor [EC:6.4.1.3] (665 aa)

RP618	PROPIONYL-COA CARBOXYLASE ALPHA CHAIN PRECURSOR (pccA) (665 aa)

RT0608	propionyl-CoA carboxylase alpha subunit precursor (665 aa)

TW020	biotin carboxylase (591 aa)

TW365	carbamoyl-phosphate synthase large chain (1103 aa)

TW630	pyruvate carboxylase (1131 aa)

carB	Purines, pyrimidines, nucleosides, and nucleotides (1084 aa)

accC	Fatty acid and phospholipid metabolism (445 aa)

WD1295	carbamoyl-phosphate synthase, large subunit (1151 aa)

WD0433	propionyl-CoA carboxylase, alpha subunit (691 aa)

Wbm0455	propionyl-CoA carboxylase, alpha subunit (632 aa)

Wbm0512	carbamoyl-phosphate synthase, large subunit (1084 aa)

Cluster 9

Number of proteins: 40

Average blastp percent identity: 40%

Average blastp sequence coverage: 91%

AM255	hypothetical protein (70 aa)

AM1125	heme exporter protein B (220 aa)

APH_1397	heme exporter protein CcmB, putative (214 aa)

APH_1030	preprotein translocase, SecE subunit (66 aa)

BH01140	Heme exporter protein B (221 aa)

BH06040	Preprotein translocase secE subunit (70 aa)

Bfl563	preprotein translocase SecE subunit (124 aa)

Bfl575	putative inner membrane protein (197 aa)

BR0095	heme exporter protein CcmB (221 aa)

BR1250	preprotein translocase, SecE subunit (79 aa)

BR1098	membrane protein, putative (209 aa)

BU040	preprotein translocase secE subunit (127 aa)

BU267	hypothetical protein (215 aa)

BU449	hypothetical protein (182 aa)

CP0843	conserved hypothetical protein (202 aa)

CBU0278	membrane protein, MarC family (197 aa)

CBU0224	preprotein translocase, SecE subunit (127 aa)

ECH_0065	heme exporter protein CcmB, putative (220 aa)

ECH_0958	preprotein translocase, SecE subunit, putative (65 aa)

ERGA_CDS_00340	Heme exporter protein B (220 aa)

ERGA_CDS_01590	Hypothetical protein (69 aa)

Erum0450	putative heme exporter protein B (220 aa)

NSE_0161	CcmB-related protein (203 aa)

NSE_0683.1	preprotein translocase, SecE subunit (60 aa)

RC0175	preprotein translocase SecE subunit (84 aa)

RC0356	heme exporter protein B (215 aa)

RP134	unknown (secE) (66 aa)

RP268	HAEM EXPORTER PROTEIN B (ccmB) (215 aa)

RT0123	preprotein translocase SecE subunit (66 aa)

RT0259	heme exporter protein B (219 aa)

TW716	unknown (80 aa)

ydeB	Hypothetical proteins (224 aa)

yhgN	Hypothetical proteins (197 aa)

secE	Protein fate (127 aa)

WD1093	heme exporter protein CcmB (215 aa)

WD0018	protein translocase subunit SecE (69 aa)

Wbm0578	heme exporter protein CcmB (215 aa)

gbw.ORF00985	preprotein translocase, SecE subunit predicted from 853500-853709 between Wbm0652 and Wbm0653 (69 aa)

Cluster 10

Number of proteins: 39

Average blastp percent identity: 34%

Average blastp sequence coverage: 66%

AM369	DNA helicase II (671 aa)

AM927	hypothetical protein (867 aa)

APH_0258	ATP-dependent DNA helicase, UvrD/Rep family (788 aa)

APH_0903	helicase, UvrD/Rep family (659 aa)

BH00270	ATP-dependent nuclease subunit A (1160 aa)

BH11070	DNA helicase II (778 aa)

Bfl268	exonuclease V, beta chain (1208 aa)

BR2103	helicase, UvrD/Rep family (1180 aa)

BR1413	DNA helicase II (858 aa)

BU454	exodeoxyribonuclease V 135 kD polypeptide (1174 aa)

BU598	ATP-dependent DNA helicase Rep (645 aa)

CP1100	ATP-dependent helicase PcrA (639 aa)

CP0007	exodeoxyribonuclease V, beta chain, putative (1050 aa)

CBU1230	UvrD/REP helicase family protein (1110 aa)

CBU2054	DNA helicase II (720 aa)

ECH_0387	ATP-dependent DNA helicase, UvrD/Rep family (860 aa)

ECH_0860	helicase, UvrD/Rep family (639 aa)

ERGA_CDS_02380	DNA helicase II (639 aa)

ERGA_CDS_06470	Putative Exodeoxyribonuclease V beta chain (857 aa)

Erum2390	DNA helicase II (639 aa)

Erum6250	putative exodeoxyribonuclease V beta chain (857 aa)

NSE_0467	ATP-dependent DNA helicase, UvrD/REP family (907 aa)

NSE_0356	helicase, UvrD/Rep family (630 aa)

RC0624	DNA helicase II [EC:3.6.1.-] (653 aa)

RC1114	ATP-dependent helicase (822 aa)

RP447	DNA HELICASE II (uvrD) (658 aa)

RP734	ATP-DEPENDENT NUCLEASE SUBUNIT A (addA) (822 aa)

RT0434	DNA helicase II (658 aa)

RT0719	ATP-dependent helicase/nuclease (823 aa)

TW080	ATP-dependent DNA helicase (743 aa)

TW519	putative DNA helicase II (973 aa)

recB	DNA metabolism (1162 aa)

uvrD	DNA metabolism (719 aa)

WD0963	helicase II - UvrD/PcrA (638 aa)

WD0359	helicase, UvrD/Rep/AddA family (1089 aa)

Wbm0172	helicase, UvrD/Rep/AddA family (1119 aa)

Wbm0628	helicase II - UvrD/PcrA (615 aa)

Cluster 11

Number of proteins: 38

Average blastp percent identity: 35%

Average blastp sequence coverage: 84%

AM474	ribosomal large subunit pseudouridine synthase (296 aa)

AM825	sfhb-like protein (327 aa)

APH_0362	ribosomal large subunit pseudouridine synthase, RluA family (324 aa)

APH_0549	ribosomal large subunit pseudouridine synthase C (297 aa)

BH03820	Ribosomal large subunit pseudouridine synthase D (334 aa)

BH10200	Ribosomal large subunit pseudouridine synthase c (327 aa)

Bfl181	pseudouridine synthase (pseudouridines 1911, 1915, 1917 in 23S RNA) (321 aa)

Bfl409	ribosomal large subunit pseudouridine synthase C (322 aa)

BR1651	ribosomal large subunit pseudouridine synthase, RluD subfamily (347 aa)

BR1000	ribosomal large subunit pseudouridine synthase C (326 aa)

BU348	ribosomal large subunit pseudouridine synthase C (314 aa)

BU401	ribosomal large subunit pseudouridine synthase D (312 aa)

CP0027	ribosomal large subunit pseudouridine synthase D (325 aa)

CBU0757	ribosomal large subunit pseudouridine synthase D (323 aa)

CBU0487	ribosomal large subunit pseudouridine synthase C (308 aa)

ECH_0486	ribosomal large subunit pseudouridine synthase, RluA family (337 aa)

ECH_0774	ribosomal large subunit pseudouridine synthase C (307 aa)

ERGA_CDS_03220	Ribosomal large subunit pseudouridine synthase C (305 aa)

ERGA_CDS_05480	SfhB protein homolog (peudouridylate synthase) (324 aa)

Erum3210	putative ribosomal large subunit pseudouridine synthase C (305 aa)

Erum5330	ribosomal large subunit pseudouridine synthase D (324 aa)

NSE_0146	ribosomal large subunit pseudouridine synthases, RluA family (330 aa)

NSE_0138	RNA pseudouridylate synthase, putative, truncation (119 aa)

NSE_0556	ribosomal large subunit pseudouridine synthase C (305 aa)

RC0344	ribosomal large subunit pseudouridine synthase C [EC:4.2.1.70] (303 aa)

RC1310	sfhB protein homolog (313 aa)

RP258	unknown (303 aa)

RP846	SFHB PROTEIN HOMOLOG (sfhB) (313 aa)

RT0249	ribosomal large subunit pseudouridylate synthase C (303 aa)

RT0834	ribosomal large subunit pseudouridylate synthase D (313 aa)

TW511	pseudouridylate synthase-like protein (303 aa)

rluD	Protein synthesis (314 aa)

WD1162	ribosomal large subunit pseudouridine synthase, RluD subfamily (276 aa)

WD0415	ribosomal large subunit pseudouridine synthase C, putative (307 aa)

Wbm0206	ribosomal large subunit pseudouridine synthase C, putative (389 aa)

Wbm5075	ribosomal large subunit pseudouridine synthase D (78 aa)

Cluster 12

Number of proteins: 37

Average blastp percent identity: 55%

Average blastp sequence coverage: 91%

AM262	RNA polymerase beta subunit (1382 aa)

AM263	DNA-directed RNA polymerase beta chain (1415 aa)

APH_1023	DNA-directed RNA polymerase, beta' subunit (1409 aa)

APH_1024	DNA-directed RNA polymerase, beta subunit (1383 aa)

BH06100	DNA-directed RNA polymerase beta chain (1383 aa)

BH06110	DNA-directed RNA polymerase beta prime chain (1402 aa)

Bfl556	DNA-directed RNA polymerase, beta-prime-subunit (1420 aa)

Bfl557	DNA-directed RNA polymerase, beta-subunit (1342 aa)

BR1243	DNA-directed RNA polymerase, beta subunit (1377 aa)

BR1242	DNA-directed RNA polymerase, beta' subunit (1400 aa)

BU033	DNA-directed RNA polymerase beta chain (1407 aa)

BU034	DNA-directed RNA polymerase beta chain (1342 aa)

CP0693	DNA-directed RNA polymerase, beta` subunit (1397 aa)

CP0694	DNA-directed RNA polymerase, beta subunit (1262 aa)

CBU0232	DNA-directed RNA polymerase beta' subunit (1414 aa)

CBU0231	DNA-directed RNA polymerase, beta subunit (1375 aa)

ECH_0951	DNA-directed RNA polymerase, beta' subunit (1410 aa)

ECH_0952	DNA-directed RNA polymerase, beta subunit (1380 aa)

ERGA_CDS_01650	DNA-directed RNA polymerase beta chain (1380 aa)

ERGA_CDS_01660	DNA-directed RNA polymerase beta chain (1411 aa)

Erum1720	DNA-directed RNA polymerase beta chain (1380 aa)

Erum1730	DNA-directed RNA polymerase beta chain (1411 aa)

NSE_0678	DNA-directed RNA polymerase, beta subunit (1358 aa)

NSE_0677	DNA-directed RNA polymerase, beta' subunit (1375 aa)

RC0181	DNA-directed RNA polymerase beta chain [EC:2.7.7.6] (1373 aa)

RC0182	DNA-directed RNA polymerase beta prime chain [EC:2.7.7.6] (1372 aa)

RP140	DNA-DIRECTED RNA POLYMERASE BETA CHAIN (rpoB) (1374 aa)

RP141	DNA-DIRECTED RNA POLYMERASE BETA PRIME CHAIN (rpoC) (1372 aa)

RT0129	DNA-directed RNA polymerase beta subunit (1374 aa)

RT0130	DNA-directed RNA polymerase beta prime subunit (1372 aa)

TW071	DNA-directed RNA polymerase beta chain (1188 aa)

TW072	DNA-directed RNA polymerase beta chain (1286 aa)

rpoB	Transcription (1342 aa)

rpoC	Transcription (1405 aa)

WD0024	DNA-directed RNA polymerase, beta/beta' subunits (2837 aa)

Wbm0647	DNA-directed RNA polymerase, beta/beta' subunits (2839 aa)

Cluster 13

Number of proteins: 37

Average blastp percent identity: 41%

Average blastp sequence coverage: 78%

AM058	UDP-N-acetylglucosamine pyrophosphorylase (428 aa)

AM1088	hypothetical protein (129 aa)

APH_1197	hexapeptide transferase family protein (170 aa)

BH06290	UDP-3-o-[3-hydroxymyristoyl] glucosamine N-acyltransferase (348 aa)

BH09930	UDP-N-acetylglucosamine pyrophosphorylase (448 aa)

Bfl010	UDP-N-acetylglucosamine pyrophosphorylase (465 aa)

Bfl281	UDP-3-O-[3-hydroxymyristoyl] glucosamine N-acyltransferase (369 aa)

BR1260	ferripyochelin-binding protein, putative (175 aa)

BR1153	UDP-3-O-(3-hydroxymyristoyl) glucosamine N-acyltransferase (351 aa)

BRA0583	UDP-N-acetylglucosamine pyrophosphorylase (454 aa)

BU027	UDP-N-acetylglucosamine pyrophosphorylase (459 aa)

CP1124	UDP-N-acetlyglucosamine pyrophosphorylase GlmU-related enzyme (176 aa)

CP0456	UDP-3-O-(R-3-hydroxymyristoyl)-glucosamine N-acyltransferase (360 aa)

CBU0832	acetyltransferase, putative (183 aa)

CBU0613	UDP-3-O-[3-hydroxymyristoyl] glucosamine N-acyltransferase (342 aa)

CBU1947	UDP-N-acetylglucosamine pyrophosphorylase (455 aa)

ECH_1066	hexapeptide transferase family protein (170 aa)

ERGA_CDS_08600	Conserved hypothetical protein (172 aa)

Erum8210	putative transferase (172 aa)

NSE_0045	hexapeptide transferase family protein (185 aa)

RC0010	UDP-3-O-[3-hydroxymyristoyl] glucosamine N-acyltransferase [EC:2.3.1.-] (346 aa)

RC0635	unknown (130 aa)

RC0680	UDP-N-acetylglucosamine pyrophosphorylase [EC:2.7.7.23] (248 aa)

RP009	UDP-3-O-[3-HYDROXYMYRISTOYL] GLUCOSAMINE N-ACYLTRANSFERASE (lpxD) (346 aa)

RP454	UDP-N-ACETYLGLUCOSAMINE PYROPHOSPHORYLASE (glmU) (249 aa)

RP516	unknown (185 aa)

RT0008	UDP-3-O-[3-hydroxymyristoyl] glucosamine N-acyltransferase (346 aa)

RT0441	UDP-N-acetylglucosamine pyrophosphorylase (248 aa)

RT0502	conserved hypothetical protein (185 aa)

TW188	putative UDP-N-acetylglucosamine pyrophosphorylase (605 aa)

glmU	Cell envelope (461 aa)

lpxD	Cell envelope (340 aa)

WD0133	UDP-N-acetylglucosamine pyrophosphorylase (430 aa)

WD0466	hexapeptide transferase family protein (171 aa)

Wbm0088	UDP-N-acetylglucosamine pyrophosphorylase (394 aa)

Wbm0289	hexapeptide transferase family protein (176 aa)

Cluster 14

Number of proteins: 36

Average blastp percent identity: 42%

Average blastp sequence coverage: 86%

AM1027	hypothetical protein (343 aa)

AM1266	chromosome partitioning protein (271 aa)

APH_1111	ATP-binding protein, Mrp/Nbp35 family (342 aa)

BH12380	mrp protein (364 aa)

BH16660	Chromosome partitioning protein parA (265 aa)

Bfl440	septum site determining protein MinD (274 aa)

BR0057	mrp-related protein (387 aa)

BR2059	chromosome partitioning protein ParA (265 aa)

BRA0322	septum site-determining protein MinD (271 aa)

BU326	septum site-determining protein minD (270 aa)

CP1066	ParA family protein (255 aa)

CBU1926	sporulation initiation inhibitor protein soj (256 aa)

CBU1689	mrp protein, putative (306 aa)

ECH_1023	conserved hypothetical protein (352 aa)

ECH_1156	chromosome partitioning ATPase, ParA family (256 aa)

ERGA_CDS_08240	Mrp protein (349 aa)

ERGA_CDS_09250	Sporulation initiation inhibitor protein soj (260 aa)

Erum7890	conserved hypothetical protein (349 aa)

Erum8830	chromosome partitioning protein ParA (255 aa)

NSE_0888	ATP-binding protein, Mrp/Nbp35 family (246 aa)

RC0086	soj protein (255 aa)

RC0163	Mrp protein (319 aa)

RP058	SOJ PROTEIN (soj) (255 aa)

RP121	unknown (318 aa)

RT0074	chromosome partitioning protein ParA (Soj protein) (255 aa)

RT0110	ATP-binding protein Mrp (318 aa)

TW049	putative sporulation protein Soj (260 aa)

TW525	ATP-binding Mrp protein (389 aa)

TW802	chromosome partitioning protein ParA (300 aa)

minD	Cellular processes (277 aa)

WD1217	ParA family protein (280 aa)

WD0179	GTP/ATP binding protein, putative (340 aa)

Wbm0063	ATP-binding protein, Mrp/Nbp35 family (335 aa)

Wbm5089	ParA family protein pseudogene portion from 926186-926311 (41 aa)

Wbm5089	ParA family protein pseudogene portion from 926516-926671 (51 aa)

Cluster 15

Number of proteins: 35

Average blastp percent identity: 41%

Average blastp sequence coverage: 97%

AM215	glutathione reductase (470 aa)

AM797	dihydrolipoamide dehydrogenase (471 aa)

APH_0065	dihydrolipoamide dehydrogenase (471 aa)

APH_0393	dihydrolipoamide dehydrogenase (468 aa)

APH_1070	dihydrolipoamide dehydrogenase (471 aa)

BH05780	Dihydrolipoamide dehydrogenase (486 aa)

BH16520	Dihydrolipoamide dehydrogenase (468 aa)

Bfl151	dihydrolipoamide dehydrogenase (470 aa)

BR1918	2-oxoglutarate dehydrogenase, E3 component, lipoamide dehydrogenase (467 aa)

BR1126	pyruvate dehydrogenase complex, E3 component, lipoamide dehydrogenase (487 aa)

BRA0527	2-oxoisovalerate dehydrogenase, E3 component, lipoamide dehydrogenase (464 aa)

BU207	dihydrolipoamide dehydrogenase (473 aa)

CP1037	2-oxo acid dehydrogenase, E3 component, lipoamide dehydrogenase, putative (461 aa)

CBU0463	dihydrolipoamide dehydrogenase (474 aa)

ECH_0509	dihydrolipoamide dehydrogenase (463 aa)

ECH_0992	dihydrolipoamide dehydrogenase (468 aa)

ERGA_CDS_01340	Dihydrolipoamide dehydrogenase (491 aa)

ERGA_CDS_05280	Dihydrolipoamide dehydrogenase (474 aa)

Erum1420	putative dihydrolipoamide dehydrogenase, E3 component of pyruvate or 2-oxoglutarate dehydrogenase complex (469 aa)

Erum5130	putative dihydrolipoamide dehydrogenase, E3 component of pyruvate or 2-oxoglutarate dehydrogenase complex (465 aa)

NSE_0671	dihydrolipoamide dehydrogenase (468 aa)

NSE_0463	dihydrolipoamide dehydrogenase (457 aa)

RC0693	dihydrolipoamide dehydrogenase precursor [EC:1.8.1.4] (459 aa)

RC1239	dihydrolipoamide dehydrogenase [EC:1.8.1.4] (459 aa)

RP460	DIHYDROLIPOAMIDE DEHYDROGENASE PRECURSOR (pdhD) (453 aa)

RP805	DIHYDROLIPOAMIDE DEHYDROGENASE (pdhD) (459 aa)

RT0447	dihydrolipoamide dehydrogenase (459 aa)

RT0792	dihydrolipoamide dehydrogenase (459 aa)

TW502	dihydrolipoamide dehydrogenase (450 aa)

lpdA	Energy metabolism (476 aa)

WD0325	alpha keto acid dehydrogenase complex, E3 component, lipoamide dehydrogenase (457 aa)

WD0751	alpha keto acid dehydrogenase complex, E3 component, lipoamide dehydrogenase (459 aa)

Wbm0559	dihydrolipoamide dehydrogenase (461 aa)

Wbm0561	dihydrolipoamide dehydrogenase (462 aa)

Cluster 16

Number of proteins: 35

Average blastp percent identity: 34%

Average blastp sequence coverage: 87%

AM876	4-hydroxybenzoate octaprenyltransferase (299 aa)

AM1009	cytochrome c oxidase assembly factor (301 aa)

APH_0310	4-hydroxybenzoate octaprenyltransferase (292 aa)

APH_1086	protoheme IX farnesyltransferase (296 aa)

BH04280	4-hydroxybenzoate-octaprenyl transferase (334 aa)

BH04400	Protoheme IX farnesyltransferase (312 aa)

Bfl025	4-hydroxybenzoate octaprenyl transferase (284 aa)

Bfl241	protohaeme IX farnesyltransferase (haeme O biosynthesis) (293 aa)

BR0469	protoheme IX farnesyltransferase (315 aa)

BR0415	4-hydroxybenzoate octaprenyltransferase (333 aa)

BU468	protohaeme IX farnesyltransferase (285 aa)

CP0496	4-hydroxybenzoate octaprenyltransferase (297 aa)

CBU0996	protoheme IX farnesyltransferase (305 aa)

CBU0055	4-hydroxybenzoate octaprenyltransferase (287 aa)

ECH_0441	4-hydroxybenzoate octaprenyltransferase (291 aa)

ECH_1004	protoheme IX farnesyltransferase (295 aa)

ERGA_CDS_05990	4-hydroxybenzoate octaprenyltransferase (295 aa)

ERGA_CDS_08090	Protoheme IX farnesyltransferase (295 aa)

Erum5790	4-hydroxybenzoate octaprenyltransferase (295 aa)

Erum7750	putative protoheme IX farnesyltransferase (295 aa)

NSE_0806	4-hydroxybenzoate octaprenyltransferase (280 aa)

NSE_0623	protoheme IX farnesyltransferase (302 aa)

RC0469	cytochrome c oxidase assembly factor [EC:1.9.3.1] (305 aa)

RC1049	4-hydroxybenzoate octaprenyltransferase [EC:2.5.1.-] (282 aa)

RP346	CYTOCHROME C OXIDASE ASSEMBLY FACTOR (ctaB) (310 aa)

RP686	4-HYDROXYBENZOATE OCTAPRENYLTRANSFERASE (ubiA) (282 aa)

RT0681	4-hydroxybenzoate octaprenyltransferase (282 aa)

TW339	protoheme IX farnesyltransferase (300 aa)

ubiA	Biosynthesis of cofactors, prosthetic groups, and carrier (273 aa)

cyoE	Energy metabolism (288 aa)

WD1316	4-hydroxybenzoate octaprenyltransferase (285 aa)

WD0302	protoheme IX farnesyltransferase (295 aa)

Wbm0306	protoheme IX farnesyltransferase (295 aa)

Wbm0535	4-hydroxybenzoate polyprenyl transferase (268 aa)

Cluster 17

Number of proteins: 34

Average blastp percent identity: 43%

Average blastp sequence coverage: 84%

AM096	thioredoxin (115 aa)

AM156	hypothetical protein (183 aa)

AM485	hypothetical protein (199 aa)

APH_0080	thioredoxin (110 aa)

APH_0141	thiol:disulfide oxidoreductase, putative (166 aa)

APH_0561	thiol-disulfide oxidoreductase, putative (210 aa)

BH00260	Thioredoxin (107 aa)

BH01110	Thiol 3A-disulfide interchange protein (215 aa)

BH16180	Thiol 3-disulfide interchange protein (232 aa)

Bfl587	thioredoxin 1, redox factor (113 aa)

BR0098	thiol:disulfide interchange protein, thioredoxin family (203 aa)

BR2105	thioredoxin (107 aa)

BR1980	thiol:disulfide interchange protein, putative (238 aa)

BU597	thioredoxin (108 aa)

CP0088	thioredoxin (102 aa)

CBU2087	thioredoxin (112 aa)

ECH_0218	thioredoxin (107 aa)

ECH_0304	thiol:disulfide oxidoreductase, putative (165 aa)

ERGA_CDS_07180	Thiol:disulfide interchange protein dsb (166 aa)

ERGA_CDS_07850	Thioredoxin (107 aa)

Erum6910	putative thiol:disulfide interchange protein (166 aa)

Erum7540	thioredoxin 1 (107 aa)

NSE_0894	thioredoxin 1 (102 aa)

RC0002	thioredoxin (105 aa)

RC0544	thiol:disulfide interchange protein tlpA (214 aa)

RP002	THIOREDOXIN (trxA) (130 aa)

RP397	THIOL:DISULFIDE INTERCHANGE PROTEIN TLPA (tlpA) (221 aa)

RT0002	thioredoxin (105 aa)

RT0385	thiol-disulfide interchange protein (220 aa)

TW756	thiol:disulfide interchange protein (228 aa)

trxA	Energy metabolism (112 aa)

WD0879	thioredoxin (107 aa)

Wbm0608	thioredoxin (107 aa)

Cluster 18

Number of proteins: 34

Average blastp percent identity: 42%

Average blastp sequence coverage: 90%

AM655	cysteine desulfurase (408 aa)

AM656	hypothetical protein (520 aa)

APH_0673	rrf2 family protein /cysteine desulfurase (486 aa)

APH_0674	cysteine desulfurase (412 aa)

BH08650	Nitrogenase cofactor synthesis protein nifS (384 aa)

Bfl534	putative L-cysteine desulfurase (395 aa)

BR0930	cysteine desulfurase, putative (392 aa)

BU602	hypothetical protein (404 aa)

CP0359	nifS protein, putative (371 aa)

CBU1182	cysteine desulfurase (380 aa)

CBU1129	cysteine desulfurase (396 aa)

ECH_0628	rrf2/aminotransferase, class V family protein (492 aa)

ECH_0629	cysteine desulfurase (410 aa)

ERGA_CDS_04220	Conserved hypothetical protein (145 aa)

ERGA_CDS_04240	Cysteine desulfurase (NifS protein homolog) duplication (526 aa)

ERGA_CDS_04250	Cysteine desulfurase (NifS protein homolog) (413 aa)

Erum4120	conserved hypothetical protein (156 aa)

Erum4140	hypothetical protein (522 aa)

Erum4150	cysteine desulfurase (413 aa)

NSE_0304	rrf2/aminotransferase, class V family protein (530 aa)

NSE_0303	cysteine desulfurase (402 aa)

RC0730	nifs protein homolog (410 aa)

RC0731	nifs protein homolog (372 aa)

RP486	NIFS PROTEIN HOMOLOG (spl1) (410 aa)

RP487	NIFS PROTEIN HOMOLOG (spl1) (376 aa)

RT0473	cysteine desulfurase protein IscS/NifS (410 aa)

RT0474	cysteine desulfurase protein IscS/NifS (378 aa)

TW416	cysteine desulfurase NifS-like protein (383 aa)

yfhO	Biosynthesis of cofactors, prosthetic groups, and carrier (386 aa)

WD0997	cysteine desulfurase (415 aa)

WD0705	aminotransferase, class V (378 aa)

Wbm0021	aminotransferase, class V (386 aa)

Wbm0028	cysteine desulfurase (431 aa)

Cluster 19

Number of proteins: 34

Average blastp percent identity: 37%

Average blastp sequence coverage: 90%

AM039	glutamate tRNA ligase (441 aa)

AM775	glutamyl-tRNA synthetase (516 aa)

APH_0072	glutamyl-tRNA synthetase (450 aa)

APH_0408	glutamyl-tRNA synthetase (468 aa)

BH06390	Glutamyl-tRNA synthetase (483 aa)

BH06460	Glutamyl-tRNA synthetase (459 aa)

Bfl504	glutamyl-tRNA synthetase (483 aa)

BR1147	glutamyl-tRNA synthetase (473 aa)

BR1016	glutamyl-tRNA synthetase (457 aa)

BU070	glutamyl-tRNA synthetase (467 aa)

CP0190	glutamyl-tRNA synthetase (505 aa)

CBU1488	glutamyl-tRNA synthetase (464 aa)

CBU0205	glutamyl-tRNA synthetase (469 aa)

ECH_0208	glutamyl-tRNA synthetase (443 aa)

ECH_0605	glutamyl-tRNA synthetase (469 aa)

ERGA_CDS_04420	Glutamyl-tRNA synthetase 2 (470 aa)

ERGA_CDS_07930	Glutamyl-tRNA synthetase 1 (445 aa)

Erum4310	glutamyl-tRNA synthetase 2 (470 aa)

Erum7610	glutamyl-tRNA synthetase 1 (443 aa)

NSE_0737	glutamyl-tRNA synthetase (409 aa)

NSE_0518	glutamyl-tRNA synthetase (433 aa)

RC0448	glutamyl-tRNA synthetase [EC:6.1.1.17] (447 aa)

RC0966	glutamyl-tRNA synthetase [EC:6.1.1.17] (513 aa)

RP325	GLUTAMYL-TRNA SYNTHETASE (gltX1) (447 aa)

RP623	GLUTAMYL-TRNA SYNTHETASE (gltX2) (470 aa)

RT0316	glutamate--tRNA ligase (448 aa)

RT0614	glutamate--tRNA ligase (469 aa)

TW209	glutamyl-tRNA synthetase (480 aa)

gltX	Protein synthesis (473 aa)

WD0435	glutamyl-tRNA synthetase (473 aa)

WD0778	glutamyl-tRNA synthetase (440 aa)

Wbm0445	glutamyl-tRNA synthetase (440 aa)

Wbm0453	glutamyl-tRNA synthetase (453 aa)

Cluster 20

Number of proteins: 33

Average blastp percent identity: 42%

Average blastp sequence coverage: 86%

AM511	thioredoxin peroxidase 1 (207 aa)

AM685	hypothetical protein (160 aa)

APH_0659	antioxidant, AhpC/Tsa family (203 aa)

APH_0795	antioxidant, AhpC/Tsa family (154 aa)

BH08680	Bacterioferritin comigratory protein (162 aa)

Bfl228	alkyl hydroperoxide reductase C22 protein (199 aa)

Bfl519	bacterioferritin comigratory protein (159 aa)

BR0924	bacterioferritin comigratory protein (154 aa)

BRA0708	alkyl hydroperoxide reductase C (184 aa)

BU182	alkyl hydroperoxide reductase (197 aa)

CP1094	antioxidant, AhpC/Tsa family (208 aa)

CBU1477	antioxidant, AhpC/TSA family (179 aa)

CBU0963	bacterioferritin comigratory protein (151 aa)

CBU1706	antioxidant, AhpC/Tsa family (200 aa)

ECH_0539	antioxidant, AhpC/TSA family (147 aa)

ECH_0734	antioxidant, AhpC/Tsa family (205 aa)

ERGA_CDS_03510	Probable peroxiredoxin (26 kDa antigen) (205 aa)

ERGA_CDS_05010	Bacterioferritin comigratory protein (147 aa)

Erum3480	putative peroxiredoxin (205 aa)

Erum4880	putative bacterioferritin comigratory protein (147 aa)

NSE_0723	bacterioferritin comigratory protein (162 aa)

NSE_0559	antioxidant, AhpC/Tsa family (200 aa)

RC0451	thioredoxin peroxidase 1 [EC:1.6.4.-] (202 aa)

RC1090	bacterioferritin comigratory protein (156 aa)

RP327	THIOREDOXIN PEROXIDASE 1 (tdpX1) (200 aa)

RT0318	thioredoxin peroxidase I (200 aa)

TW256	thiol-specific antioxidant protein (156 aa)

ahpC	Cellular processes (199 aa)

WD1285	bacterioferritin comigratory protein (155 aa)

WD0756	antioxidant, AhpC/Tsa family (199 aa)

Wbm0127	bacterioferritin comigratory protein (155 aa)

Wbm0567	antioxidant, AhpC/Tsa family, authentic frameshift (204 aa)

Cluster 21

Number of proteins: 32

Average blastp percent identity: 36%

Average blastp sequence coverage: 78%

AM861	geranyltranstransferase (farnesyl-diphosphate synthase) (291 aa)

AM1208	octaprenyl-diphosphate synthase (327 aa)

APH_1247	octaprenyl-diphosphate synthase (316 aa)

APH_0329	geranyltranstransferase (280 aa)

BH04180	Octaprenyl-diphosphate synthase (356 aa)

BH15260	Geranyltranstransferase (farnesyl-diphosphate synthase) (300 aa)

Bfl092	octaprenyl-diphosphate synthase (324 aa)

BR0401	polyprenyl synthetase family protein (340 aa)

BR1777	geranyltranstransferase (304 aa)

BU465	geranyltranstransferase (282 aa)

CP1125	geranylgeranyl pyrophosphate synthase, putative (289 aa)

CBU1255	geranyltranstransferase, putative (267 aa)

CBU0382	octylprenyl diphosphate synthase-like protein (332 aa)

ECH_0088	octaprenyl-diphosphate synthase (325 aa)

ECH_0455	geranyltranstransferase, putative (267 aa)

ERGA_CDS_00500	Octaprenyl-diphosphate synthase (325 aa)

ERGA_CDS_05840	Possible geranyltranstransferase (276 aa)

Erum0600	octaprenyl-diphosphate synthase (325 aa)

Erum5660	putative geranyltranstransferase (276 aa)

NSE_0238	polyprenyl synthetase family protein (321 aa)

NSE_0694	geranyltranstransferase, putative (256 aa)

RC0724	octaprenyl-diphosphate synthase [EC:2.5.1.-] (371 aa)

RP479	OCTAPRENYL-DIPHOSPHATE SYNTHASE (ispB) (327 aa)

RT0466	octaprenyl-diphosphate synthase (327 aa)

TW054	probable heptaprenyl diphosphate synthase component II (338 aa)

ispA	Biosynthesis of cofactors, prosthetic groups, and carrier (300 aa)

ispB	Biosynthesis of cofactors, prosthetic groups, and carrier (323 aa)

WD1192	geranyltranstransferase (335 aa)

WD0799	octaprenyl-diphosphate synthase (328 aa)

Wbm0532	octaprenyl-diphosphate synthase (328 aa)

Wbm0737	geranyltranstransferase (305 aa)

Cluster 22

Number of proteins: 30

Average blastp percent identity: 72%

Average blastp sequence coverage: 97%

AM254	translation elongation factor Tu (393 aa)

AM914	translation elongation factor Tu (393 aa)

APH_0278	translation elongation factor Tu (393 aa)

APH_1032	translation elongation factor Tu (393 aa)

BH06020	Elongation factor Tu (EF-Tu) (391 aa)

BH10530	Elongation factor tu (EF-tu) (391 aa)

Bfl564	elongation factor Tu (EF-Tu) (394 aa)

BR1251	translation elongation factor Tu (391 aa)

BR1235	translation elongation factor Tu (391 aa)

BU526	elongation factor EF-Tu (423 aa)

CP0701	translation elongation factor Tu (394 aa)

CBU0236	translation elongation factor Tu (397 aa)

CBU0223	translation elongation factor Tu (299 aa)

ECH_0960	translation elongation factor Tu (395 aa)

ECH_0407	translation elongation factor Tu (395 aa)

ERGA_CDS_01580	Elongation factor Tu (EF-Tu) (395 aa)

ERGA_CDS_06310	Elongation factor Tu (EF-Tu) (407 aa)

Erum1660	elongation factor Tu-A (395 aa)

Erum6090	elongation factor Tu-B (395 aa)

NSE_0686	translation elongation factor Tu (430 aa)

RC1008	elongation factor EF-Tu (394 aa)

RP661	ELONGATION FACTOR TU (tuf) (394 aa)

RT0653	elongation factor Tu (394 aa)

TW120	elongation factor EF-Tu (397 aa)

tufA	Protein synthesis (394 aa)

WD0017	translation elongation factor Tu (390 aa)

WD0683	translation elongation factor Tu (390 aa)

Wbm0343	translation elongation factor Tu (399 aa)

Wbm0653	translation elongation factor Tu (390 aa)

Cluster 23

Number of proteins: 29

Average blastp percent identity: 37%

Average blastp sequence coverage: 91%

AM145	UDP-N-acetylglucosamine 1-carboxyvinyltransferase (428 aa)

AM289	3-phosphoshikimate 1-carboxyvinyltransferase (462 aa)

APH_1001	3-phosphoshikimate 1-carboxyvinyltransferase, putative (440 aa)

BH00950	3-phosphoshikimate 1-carboxyvinyltransferase (442 aa)

BH14420	UDP-N-acetylglucosamine 1-carboxyvinyltransferase (431 aa)

Bfl046	UDP-N-acetylglucosamine 1-carboxyvinyltransferase (420 aa)

Bfl382	3-phosphoshikimate 1-carboxyvinyltransferase (433 aa)

BR0254	UDP-N-acetylglucosamine 1-carboxyvinyltransferase (429 aa)

BR0025	3-phosphoshikimate 1-carboxyvinyltransferase (450 aa)

BU311	3-phosphoshikimate 1-carboxyvinyltransferase (427 aa)

BU386	UDP-N-acetylglucosamine 1-carboxyvinyltransferase (416 aa)

CP0813	3-phosphoshikimate 1-carboxyvinyltransferase (445 aa)

CP0178	UDP-N-acetylglucosamine 1-carboxyvinyltransferase (458 aa)

CBU0751	UDP-N-acetylglucosamine 1-carboxyvinyltransferase (434 aa)

CBU0526	3-phosphoshikimate 1-carboxyvinyltransferase (438 aa)

ECH_0920	3-phosphoshikimate 1-carboxyvinyltransferase, putative (428 aa)

ERGA_CDS_01830	3-phosphoshikimate 1-carboxyvinyltransferase (427 aa)

Erum1880	3-phosphoshikimate 1-carboxyvinyltransferase (427 aa)

NSE_0180	3-phosphoshikimate 1-carboxyvinyltransferase, putative (435 aa)

RC0883	UDP-N-acetylglucosamine 1-carboxyvinyltransferase [EC:2.5.1.7] (419 aa)

RP579	UDP-N-ACETYLGLUCOSAMINE 1-CARBOXYVINYLTRANSFERASE (murA) (419 aa)

RT0568	UDP-N-acetylglucosamine 1-carboxyvinyltransferase (419 aa)

TW508	UDP-N-acetylglucosamine 1-carboxyvinyltransferase (441 aa)

TW567	3-phosphoshikimate 1-carboxyvinyltransferase (486 aa)

murA	Cell envelope (418 aa)

WD1197	UDP-N-acetylglucosamine 1-carboxyvinyltransferase (425 aa)

Wbm0740	UDP-N-acetylglucosamine 1-carboxyvinyltransferase (413 aa)

Cluster 24

Number of proteins: 29

Average blastp percent identity: 34%

Average blastp sequence coverage: 80%

AM1084	protein-(glutamine-N5) methyltransferase (285 aa)

APH_1200	modification methylase, HemK family (282 aa)

BH01960	Protoporphyrinogen oxidase protein (288 aa)

BH02190	hypothetical protein (233 aa)

Bfl349	putative protoporphyrinogen oxidase (281 aa)

Bfl499	conserved hypothetical protein (303 aa)

BR2161	methyltransferase, putative (233 aa)

BR1868	hemK protein (283 aa)

BU172	hemK protein (277 aa)

BU551	hypothetical protein (239 aa)

CP0869	conserved hypothetical protein (224 aa)

CP0659	modification methylase, HemK family (288 aa)

CBU1964	protein methyltransferase HemK (277 aa)

ECH_1063	modification methylase, HemK family (280 aa)

ERGA_CDS_08540	Bifunctional methyltransferase-hemK proteinhomolog and tRNA (guanine-N(7)-)-methyltransferase protoporphyrinogen oxidase (hemK) (280 aa)

Erum8150	putative methyltransferase (280 aa)

NSE_0128	modification methylase, HemK family (289 aa)

NSE_0922	tRNA (guanine-N(7)-)-methyltransferase, putative (217 aa)

RC1314	possible protoporphyrinogen oxidase [EC:1.3.3.4] (524 aa)

RP847	POSSIBLE PROTOPORPHYRINOGEN OXIDASE (hemK) (518 aa)

RT0836	bifunctional methyltransferase (518 aa)

TW434	protoporphyrinogen oxidase (285 aa)

hemK	Biosynthesis of cofactors, prosthetic groups, and carrier (281 aa)

yfcB	DNA metabolism (307 aa)

WD1091	methyltransferase, putative (221 aa)

WD0010	modification methylase, HemK family (279 aa)

Wbm0285	modification methylase, HemK family (279 aa)

Cluster 25

Number of proteins: 26

Average blastp percent identity: 42%

Average blastp sequence coverage: 76%

AM1351	pyruvate dehydrogenase E1 beta subunit precursor (341 aa)

APH_1308	pyruvate dehydrogenase complex, E1 component, beta subunit, putative (332 aa)

BH04350	1-deoxyxylulose-5-phosphate synthase (640 aa)

BH05760	Pyruvate dehydrogenase E1 component beta subunit (457 aa)

Bfl238	1-deoxyxylulose-5-phosphate synthase (617 aa)

BR0436	deoxyxylulose-5-phosphate synthase (643 aa)

BR1128	pyruvate dehydrogenase complex, E1 component, beta subunit (461 aa)

BRA0525	2-oxoisovalerate dehydrogenase, E1 component, beta subunit (337 aa)

BU464	dxs protein (608 aa)

CP0453	pyruvate dehydrogenase, E1 component, beta subunit (328 aa)

CP0743	2-oxoisovalerate dehydrogenase, E1 component, alpha and beta subunit (678 aa)

CP0790	1-deoxyxylulose-5-phosphate synthase (644 aa)

CBU0639	dehydrogenase, E1 component, beta subunit, putative (326 aa)

ECH_0149	pyruvate dehydrogenase complex, E1 component, beta subunit, putative (332 aa)

ERGA_CDS_00910	Pyruvate dehydrogenase E1 component, beta subunit precursor (332 aa)

Erum0980	putative pyruvate dehydrogenase E1 component, beta subunit (332 aa)

NSE_0746	pyruvate dehydrogenase complex, E1 component, beta subunit, putative (332 aa)

RC0348	pyruvate dehydrogenase e1 component, beta subunit precursor (326 aa)

RP262	PYRUVATE DEHYDROGENASE E1 COMPONENT, BETA SUBUNIT PRECURSOR (326 aa)

RT0253	pyruvate dehydrogenase (lipoamide) E1 component, beta subunit precursor (326 aa)

TW484	1-deoxyxylulose-5-phosphate synthase (629 aa)

TW789	pyruvate dehydrogenase E1 component beta subunit (334 aa)

dxs	Biosynthesis of cofactors, prosthetic groups, and carrier (626 aa)

WD0473	pyruvate dehydrogenase complex, E1 component, pyruvate dehydrogenase beta subunit, putative (332 aa)

Wbm0666	pyruvate dehydrogenase E1 beta subunit (315 aa)

Cluster 26

Number of proteins: 26

Average blastp percent identity: 40%

Average blastp sequence coverage: 89%

AM1087	dihydrolipoamide acetyltransferase component (437 aa)

APH_1198	2-oxoglutarate dehydrogenase, E2 component, dihydrolipoamide succinyltransferase (406 aa)

BH16530	Dihydrolipoamide succinyltransferase (406 aa)

Bfl152	dihydrolipoamide acetyltransferase component (E2) of pyruvate dehydrogenase (425 aa)

Bfl332	dihydrolipoamide succinyltransferase component (E2) (438 aa)

BR1922	2-oxoglutarate dehydrogenase, E2 component, dihydrolipoamide succinyltransferase (408 aa)

BRA0526	2-oxoisovalerate dehydrogenase E2 component, dihydrolipoamide acetyltransferase (431 aa)

BU206	dihydrolipoamide acetyltransferase (396 aa)

BU303	dihydrolipoamide succinyltransferase component (E2) of 2-oxoglutarate dehydrogenase complex (420 aa)

CP0225	2-oxo acid dehydrogenase, E2 component, lipoamide acyltransferase (393 aa)

CP0379	2-oxoglutarate dehydrogenase, E2 component, dihydrolipoamide succinyltransferase (364 aa)

CBU1398	2-oxoglutarate dehydrogenase, E2 component, dihydrolipoamide succinyltransferase (405 aa)

CBU0462	pyruvate dehydrogenase, E2 component, dihydrolipoamide acetyltransferase (436 aa)

ECH_1065	2-oxoglutarate dehydrogenase, E2 component, dihydrolipoamide succinyltransferase (404 aa)

ERGA_CDS_08590	Dihydrolipoamide succinyltransferase component of 2-oxoglutarate dehydrogenase complex (402 aa)

Erum8200	dihydrolipoamide succinyltransferase, E2 component of 2-oxoglutarate dehydrogenase complex (402 aa)

NSE_0548	2-oxoglutarate dehydrogenase, E2 component, dihydrolipoamide succinyltransferase (427 aa)

RC0226	dihydrolipoamide acetyltransferase component [EC:2.3.1.61] (395 aa)

RP179	DIHYDROLIPOAMIDE ACETYLTRANSFERASE COMPONENT (sucB) (401 aa)

RT0170	dihydrolipoamide S-succinyltransferase (398 aa)

TW503	dihydrolipoamide succinyltransferase component E2 (461 aa)

aceF	Energy metabolism (496 aa)

sucB	Energy metabolism (413 aa)

WD0544	2-oxoglutarate dehydrogenase, E2 component, dihydrolipoamide succinyltransferase (390 aa)

Wbm0108	2-oxoglutarate dehydrogenase, E2 component, dihydrolipoamide succinyltransferase (386 aa)

Cluster 27

Number of proteins: 25

Average blastp percent identity: 66%

Average blastp sequence coverage: 95%

AM051	transcription termination factor (454 aa)

AM992	transcription termination factor RHO (460 aa)

APH_0064	transcription termination factor Rho (452 aa)

APH_1071	transcription termination factor Rho (443 aa)

BH16700	Transcription termination factor rho (421 aa)

Bfl586	transcription termination factor Rho (419 aa)

BR2064	transcription termination factor Rho (421 aa)

BU596	transcription termination factor rho (419 aa)

CP0137	transcription termination factor Rho (464 aa)

CBU2086	transcription termination factor Rho (418 aa)

ECH_0994	transcription termination factor Rho (422 aa)

ECH_0200	transcription termination factor Rho (422 aa)

ERGA_CDS_01320	Transcription termination factor RHO (478 aa)

ERGA_CDS_08000	Transcription termination factor rho (458 aa)

Erum1400	transcription termination factor 1 (478 aa)

Erum7670	transcription termination factor 2 (458 aa)

NSE_0067	transcription termination factor Rho (439 aa)

RC0759	transcription termination factor (458 aa)

RP526	TRANSCRIPTION TERMINATION FACTOR RHO (rho) (457 aa)

RT0513	transcription termination factor Rho (450 aa)

TW436	transcription termination factor Rho (454 aa)

rho	Transcription (419 aa)

WD0795	transcription termination factor Rho (435 aa)

Wbm0528	transcription termination factor Rho (471 aa)

Cluster 28

Number of proteins: 25

Average blastp percent identity: 39%

Average blastp sequence coverage: 83%

AM199	asparaginyl-tRNA synthetase (325 aa)

AM200	asparaginyl-tRNA synthetase (296 aa)

APH_0190	aspartyl-tRNA synthetase (598 aa)

BH09790	Aspartyl-tRNA synthetase (596 aa)

Bfl421	asparaginyl-tRNA synthetase (470 aa)

Bfl452	aspartyl-tRNA synthetase (576 aa)

BR0751	aspartyl-tRNA synthetase (595 aa)

BU316	aspartyl-tRNA synthetase (586 aa)

BU360	asparaginyl-tRNA synthetase (466 aa)

CP0085	aspartyl-tRNA synthetase (584 aa)

CBU1565	aspartyl-tRNA synthetase (590 aa)

ECH_0334	aspartyl-tRNA synthetase (590 aa)

ERGA_CDS_06890	Aspartyl-tRNA synthetase (590 aa)

Erum6660	aspartyl-tRNA synthetase (590 aa)

NSE_0477	aspartyl-tRNA synthetase (588 aa)

RC0187	asparaginyl-tRNA synthetase [EC:6.1.1.12] (615 aa)

RP145	ASPARAGINYL-TRNA SYNTHETASE (aspS) (605 aa)

RT0134	aspartate--tRNA ligase (605 aa)

TW754	aspartyl-tRNA synthetase (446 aa)

aspS	Protein synthesis (560 aa)

asnS	Protein synthesis (466 aa)

WD0413	aspartyl-tRNA synthetase (600 aa)

Wbm0012	aspartyl-tRNA synthetase (600 aa)

Cluster 29

Number of proteins: 24

Average blastp percent identity: 39%

Average blastp sequence coverage: 86%

AM1066	hypothetical protein (487 aa)

APH_1148	protease DO family protein (490 aa)

BH04770	Serine protease (503 aa)

BH10250	Serine protease (463 aa)

BH10940	Serine protease (505 aa)

Bfl047	serine protease (461 aa)

BR1394	serine protease Do, putative (524 aa)

BR1207	serine protease (474 aa)

BR0611	serine protease (513 aa)

BU228	protease do precursor (478 aa)

CP0877	serine protease, HtrA/DegQ/DegS family (488 aa)

CBU0755	protease DO (451 aa)

ECH_1052	serine protease, DO/DeqQ family (471 aa)

ERGA_CDS_08430	Probable serine protease do-like precursor (476 aa)

Erum8050	putative exported serine protease (476 aa)

NSE_0166	periplasmic serine protease, DO/DeqQ family (473 aa)

RC0166	periplasmic serine protease [EC:3.4.21.-] (511 aa)

RP124	PROBABLE PERIPLASMIC SERINE PROTEASE DO-LIKE PRECURSOR (htrA) (513 aa)

RT0113	serine protease, HtrA/DegQ/DegS family (518 aa)

TW625	putative serine protease (420 aa)

degQ	Protein fate (457 aa)

WD0833	protease DO (497 aa)

Wbm0770	protease DO (496 aa)

Cluster 30

Number of proteins: 24

Average blastp percent identity: 34%

Average blastp sequence coverage: 72%

AM517	hypothetical protein (197 aa)

APH_0610	glycoprotease family protein (195 aa)

BH02270	Peptide N-acetyltransferase (163 aa)

BH02280	hypothetical protein (227 aa)

Bfl442	conserved hypothetical protein (220 aa)

BR2151	acetyltransferase, GNAT family (166 aa)

BR2150	protease, putative (261 aa)

BU324	hypothetical protein (221 aa)

CP0174	acetyltransferase, GNAT family (171 aa)

CP0746	conserved hypothetical protein (213 aa)

CBU1371	protease, putative (226 aa)

CBU0801	ribosomal-protein-alanine acetyltransferase (145 aa)

ECH_0730	glycoprotease family protein (195 aa)

ERGA_CDS_03540	Conserved hypothetical protein (193 aa)

Erum3510	putative glycoprotease (193 aa)

NSE_0844	glycoprotease family protein (196 aa)

RC0814	unknown (232 aa)

RP551	unknown (186 aa)

RT0537	conserved hypothetical protein (225 aa)

TW592	ribosomal protein acetyltransferase (501 aa)

yeaZ	Hypothetical proteins (230 aa)

WD0210	endopeptidase-related protein (191 aa)

Wbm0110	endopeptidase-related protein (205 aa)

Cluster 31

Number of proteins: 23

Average blastp percent identity: 56%

Average blastp sequence coverage: 90%

AM666	ATP synthase beta chain (486 aa)

APH_0494	ATP synthase F1, beta subunit (479 aa)

BH15320	ATP synthase beta chain (531 aa)

Bfl008	ATP synthase beta subunit (464 aa)

BR1799	ATP synthase F1, beta subunit (521 aa)

BRA0129	flagellum-specific ATP synthase FliI (422 aa)

BU008	ATP synthase beta chain (465 aa)

BU076	flagellum-specific ATP synthase (467 aa)

CP0039	type III secretion cytoplasmic ATPase SctN (442 aa)

CBU1945	ATP synthase F1, beta subunit (461 aa)

ECH_0573	ATP synthase F1, beta subunit (507 aa)

ERGA_CDS_04710	ATP synthase beta chain (515 aa)

Erum4590	ATP synthase beta chain (504 aa)

NSE_0763	ATP synthase F1, beta subunit (474 aa)

RC1235	ATP synthase beta chain [EC:3.6.1.34] (499 aa)

RP801	ATP SYNTHASE BETA CHAIN (atpD) (474 aa)

RT0788	H(+)-transporting two-sector ATPase F(1) beta subunit (474 aa)

TW424	ATP synthase beta chain (477 aa)

atpD	Energy metabolism (462 aa)

fliI	Cellular processes (458 aa)

WD0203	ATP synthase F1, beta subunit (475 aa)

Wbm0689	ATP synthase F1, beta subunit (480 aa)

Cluster 32

Number of proteins: 23

Average blastp percent identity: 48%

Average blastp sequence coverage: 97%

AM584	3-oxoacyl-reductase (302 aa)

APH_0668	3-oxoacyl-(acyl-carrier-protein) reductase (245 aa)

BH05350	3-oxoacyl-(acyl carrierprotein) reductase (245 aa)

Bfl404	3-oxoacyl-[acyl-carrier protein] reductase (244 aa)

BR0458	3-oxoacyl-(acyl-carrier-protein) reductase (245 aa)

BU351	3-oxoacyl-[acyl-carrier protein] reductase (244 aa)

CP0462	3-oxoacyl-(acyl-carrier protein) reductase (248 aa)

CBU0495	3-oxoacyl-(acyl-carrier-protein) reductase (249 aa)

ECH_0669	3-oxoacyl-(acyl-carrier-protein) reductase (247 aa)

ERGA_CDS_03920	3-oxoacyl-[acyl-carrier protein] reductase (245 aa)

Erum3840	3-oxoacyl-[acyl carrier protein] reductase (245 aa)

NSE_0665	3-oxoacyl-[acyl-carrier protein] reductase (246 aa)

RC0054	acetoacetyl-CoA reductase [EC:1.1.1.100] (241 aa)

RC1183	3-oxoacyl reductase [EC:1.1.1.100] (248 aa)

RP035	ACETOACETYL-COA REDUCTASE (phbB) (241 aa)

RP762	3-OXOACYL REDUCTASE (fabG) (241 aa)

RT0095	acetoacetyl-CoA reductase (241 aa)

RT0748	3-oxoacyl-[acyl-carrier protein] reductase (241 aa)

TW313	3-oxoacyl-[acyl-carrier protein] reductase (238 aa)

fabG	Fatty acid and phospholipid metabolism (244 aa)

WD0650	3-oxoacyl-(acyl-carrier-protein) reductase (244 aa)

Wbm0310	3-oxoacyl-(acyl-carrier-protein) reductase (244 aa)

Cluster 33

Number of proteins: 23

Average blastp percent identity: 38%

Average blastp sequence coverage: 68%

AM1136	poly (A) polymerase (419 aa)

APH_1216	polyA polymerase/tRNA nucleotidyltransferase family protein (409 aa)

BH11930	Poly(a) polymerase (417 aa)

Bfl062	tRNA nucleotidyltransferase (411 aa)

BR1553	polyA polymerase family protein (423 aa)

BU061	tRNA nucleotidyltransferase (414 aa)

CP0894	poly(A) polymerase family protein (430 aa)

CP1024	poly(A) polymerase family protein (410 aa)

CBU0286	polyA polymerase (439 aa)

CBU1827	tRNA nucleotidyl transferase, putative (376 aa)

ECH_1116	polyA polymerase/tRNA nucleotidyltransferase family protein (401 aa)

ERGA_CDS_08970	Poly(A) polymerase (397 aa)

Erum8560	putative nucleic acid independent RNA polymerase (397 aa)

NSE_0646	polyA polymerase family protein (392 aa)

RC0015	poly(A) polymerase [EC:2.7.7.19] (436 aa)

RP015	POLY(A) POLYMERASE (pcnB) (387 aa)

RT0014	polynucleotide adenylyltransferase (387 aa)

TW800	poly(A) polymerase (484 aa)

cca	Transcription (403 aa)

WD0444	poly A polymerase family protein (391 aa)

Wbm0160	poly A polymerase family protein (391 aa)

Cluster 34

Number of proteins: 22

Average blastp percent identity: 48%

Average blastp sequence coverage: 99%

AM338	3-oxoacyl-(acyl-carrier-protein) synthase II (427 aa)

APH_0930	3-oxoacyl-(acyl-carrier-protein) synthase II (423 aa)

BH01280	3-oxoacyl-[acyl-carrier-protein ] synthase I (406 aa)

BH05370	3-oxoacyl-[acyl-carrier-protein ] synthase II (420 aa)

Bfl498	3-oxoacyl-[acyl-carrier-protein] synthase I (405 aa)

BR0461	3-oxoacyl-(acyl-carrier-protein) synthase II (420 aa)

BR2172	3-oxoacyl-(acyl-carrier-protein) synthase I (407 aa)

BU092	3-oxoacyl-[acyl-carrier-protein] synthase I (406 aa)

CP0950	3-oxoacyl-(acyl-carrier-protein) synthase II (416 aa)

CBU0497	3-oxoacyl-acyl carrier protein synthase II (414 aa)

ECH_0882	3-oxoacyl-(acyl-carrier-protein) synthase II (422 aa)

ERGA_CDS_02130	3-oxoacyl-[acyl-carrier-protein] synthase II (423 aa)

Erum2150	3-oxoacyl-[acyl-carrier-protein] synthase II (423 aa)

NSE_0453	3-oxoacyl-(acyl-carrier-protein) synthase II (415 aa)

RC1186	3-oxoacyl-[acyl carrier protein] synthase II [EC:2.3.1.41] (428 aa)

RP764	3-OXOACYL-[ACYL-CARRIER-PROTEIN] SYNTHASE II (fabF) (426 aa)

RT0750	3-oxoacyl-[acyl carrier protein] synthase II (426 aa)

TW251	3-oxoacyl-[acyl-carrier-protein] synthase (411 aa)

fabB	Fatty acid and phospholipid metabolism (407 aa)

WD1194	3-oxoacyl-(acyl-carrier-protein) synthase II (423 aa)

Wbm0739	3-oxoacyl-(acyl-carrier-protein) synthase II (422 aa)

Cluster 35

Number of proteins: 22

Average blastp percent identity: 37%

Average blastp sequence coverage: 86%

AM678	arginyl-tRNA synthetase (579 aa)

APH_0564	arginyl-tRNA synthetase FRAMESHIFT (57 aa)

APH_0700	arginyl-tRNA synthetase (580 aa)

APH_0701	hypothetical protein (40 aa)

BH09980	Arginyl-tRNA synthetase (585 aa)

Bfl453	arginyl-tRNA synthetase (583 aa)

BR0877	arginyl-tRNA synthetase (585 aa)

BU242	arginyl-tRNA synthetase (574 aa)

CP0179	arginyl-tRNA synthetase (561 aa)

CBU2008	arginyl-tRNA synthetase (592 aa)

ECH_0537	arginyl-tRNA synthetase (576 aa)

ERGA_CDS_05040	Arginyl-tRNA synthetase (601 aa)

Erum4910	arginyl-tRNA synthetase (576 aa)

NSE_0049	arginyl-tRNA synthetase (570 aa)

RC0095	arginyl-tRNA synthetase [EC:6.1.1.19] (576 aa)

RP065	ARGINYL-TRNA SYNTHETASE (argS) (576 aa)

RT0067	arginine--tRNA ligase (576 aa)

TW351	arginyl-tRNA synthetase (552 aa)

argS	Protein synthesis (576 aa)

WD0402	arginyl-tRNA synthetase (568 aa)

Wbm0205	arginyl-tRNA synthetase (565 aa)

Cluster 36

Number of proteins: 21

Average blastp percent identity: 63%

Average blastp sequence coverage: 94%

AM253	elongation factor G (690 aa)

APH_0448	translation elongation factor G, truncation (75 aa)

APH_1033	translation elongation factor G (690 aa)

BH10540	Elongation factor g (EF-g) (694 aa)

Bfl565	elongation factor G (EF-G) (709 aa)

BR1236	translation elongation factor G (694 aa)

BU527	elongation factor G (702 aa)

CP0202	translation elongation factor G (694 aa)

CBU0235	translation elongation factor G (699 aa)

ECH_0961	translation elongation factor G (690 aa)

ERGA_CDS_01570	Elongation factor G (EF-G) (689 aa)

Erum1650	elongation factor G (689 aa)

NSE_0687	translation elongation factor G (692 aa)

RC0174	elongation factor EF-G (699 aa)

RP132	ELONGATION FACTOR G (fusA) (699 aa)

RT0121	elongation factor G (699 aa)

TW675	elongation factor EF-G (701 aa)

fusA	Protein synthesis (705 aa)

WD0016	translation elongation factor G (691 aa)

Wbm0344	translation elongation factor G (688 aa)

Cluster 37

Number of proteins: 21

Average blastp percent identity: 62%

Average blastp sequence coverage: 95%

AM309	ATP-dependent clp protease proteolytic subunit (215 aa)

APH_0970	ATP-dependent Clp protease, proteolytic subunit ClpP (197 aa)

BH05880	ATP-dependent clp protease proteolytic subunit (210 aa)

Bfl246	ATP-dependent Clp protease proteolytic subunit (209 aa)

BR1109	ATP-dependent Clp protease, proteolytic subunit ClpP (209 aa)

BU475	ATP-dependent clp protease proteolytic subunit (208 aa)

CP1022	ATP-dependent Clp protease, proteolytic subunit (203 aa)

CBU0738	ATP-dependent Clp protease, proteolytic subunit ClpP (195 aa)

ECH_0901	ATP-dependent Clp protease, proteolytic subunit ClpP (199 aa)

ERGA_CDS_01970	ATP-dependent CLP protease proteolytic subunit (198 aa)

Erum2000	ATP-dependent Clp protease proteolytic subunit (198 aa)

NSE_0752	ATP-dependent Clp protease, proteolytic subunit ClpP (211 aa)

RC0746	ATP-dependent clp protease proteolytic subunit [EC:3.4.21.92] (216 aa)

RP520	ATP-DEPENDENT CLP PROTEASE PROTEOLYTIC SUBUNIT (clpP) (201 aa)

RT0507	ATP-dependent Clp protease proteolytic subunit ClpP (201 aa)

TW480	ATP-dependent Clp protease proteolytic subunit (207 aa)

TW481	ATP-dependent Clp protease proteolytic subunit (180 aa)

clpP	Protein fate (194 aa)

WD0319	ATP-dependent Clp protease, proteolytic subunit ClpP (208 aa)

Wbm0553	ATP-dependent Clp protease, proteolytic subunit ClpP (208 aa)

Cluster 38

Number of proteins: 21

Average blastp percent identity: 58%

Average blastp sequence coverage: 90%

AM1086	methionine aminopeptidase (265 aa)

APH_1199	methionine aminopeptidase, type I (264 aa)

BH08350	Methionine aminopeptidase (276 aa)

Bfl270	methionine aminopeptidase (264 aa)

BR1282	methionine aminopeptidase, type I (275 aa)

BU230	methionine aminopeptidase (264 aa)

CP0844	methionine aminopeptidase (266 aa)

CBU1388	methionine aminopeptidase, type I (258 aa)

ECH_1064	methionine aminopeptidase, type I (266 aa)

ERGA_CDS_08550	Methionine aminopeptidase (266 aa)

ERGA_CDS_08560	Hypothetical protein (61 aa)

Erum8160	methionine aminopeptidase (266 aa)

NSE_0337	methionine aminopeptidase, type I (262 aa)

RC1276	methionine aminopeptidase [EC:3.4.11.18] (259 aa)

RP824	METHIONINE AMINOPEPTIDASE (map) (259 aa)

RT0812	methionyl aminopeptidase (259 aa)

TW259	methionine aminopeptidase (308 aa)

map	Protein fate (263 aa)

WD0167	methionine aminopeptidase, type I (260 aa)

Wbm0112	methionine aminopeptidase, type I (247 aa)

Cluster 39

Number of proteins: 21

Average blastp percent identity: 56%

Average blastp sequence coverage: 90%

AM164	glycine/serine hydroxymethyltransferase (430 aa)

APH_0154	serine hydroxymethyltransferase (425 aa)

BH07540	Serine hydroxymethyltransferase (437 aa)

Bfl536	serine hydroxymethyltransferase (414 aa)

BR0765	serine hydroxymethyltransferase (438 aa)

BU289	serine hydroxymethyltransferase (417 aa)

CP0232	serine hydroxymethyltransferase (497 aa)

CBU1419	serine hydroxymethyltransferase (419 aa)

ECH_0311	serine hydroxymethyltransferase (420 aa)

ECH_0333	serine hydroxymethyltransferase domain protein (68 aa)

ERGA_CDS_07110	Serine hydroxymethyltransferase (421 aa)

Erum6840	serine hydroxymethyltransferase (421 aa)

NSE_0218	serine hydroxymethyltransferase (419 aa)

RC1146	serine hydroxymethyltransferase [EC:2.1.2.1] (420 aa)

RP743	SERINE HYDROXYMETHYLTRANSFERASE (glyA) (420 aa)

RT0728	glycine hydroxymethyltransferase (serine hydroxymethyltransferase) (420 aa)

TW635	serine hydroxymethyltransferase (428 aa)

glyA	Amino acid biosynthesis (420 aa)

WD1035	serine hydroxymethyltransferase (425 aa)

Wbm0005	serine hydroxymethyltransferase (425 aa)

Cluster 40

Number of proteins: 21

Average blastp percent identity: 55%

Average blastp sequence coverage: 89%

AM1294	glycyl-tRNA synthetase alpha subunit (283 aa)

APH_0013	glycyl-tRNA synthetase, alpha subunit (283 aa)

APH_0165	glycyl-tRNA synthetase, alpha subunit (283 aa)

BH04160	Glycyl-tRNA synthetase alpha chain (317 aa)

Bfl020	glycine-tRNA synthetase, alpha subunit (301 aa)

BR0403	glycyl-tRNA synthetase, alpha subunit (308 aa)

BU136	glycyl-tRNA synthetase alpha chain (322 aa)

CP0913	glycyl-tRNA synthetase, tetrameric type, alpha/beta subunits (1010 aa)

CBU1913	glycyl-tRNA synthetase, alpha subunit (319 aa)

ECH_0023	glycyl-tRNA synthetase, alpha subunit (280 aa)

ERGA_CDS_09480	Glycyl-tRNA synthetase alpha chain (280 aa)

Erum0110	glycyl-tRNA synthetase alpha chain (280 aa)

NSE_0757	glycyl-tRNA synthetase, alpha subunit (305 aa)

RC1317	glycyl-tRNA synthetase alpha chain [EC:6.1.1.14] (288 aa)

RP850	GLYCYL-TRNA SYNTHETASE ALPHA SUBUNIT (glyQ) (289 aa)

RT0839	glycine--tRNA ligase alpha subunit (289 aa)

TW500	glycyl-tRNA synthetase alpha chain and beta chain (1024 aa)

glyQ	Protein synthesis (302 aa)

WD0156	glycyl-tRNA synthetase, alpha subunit (279 aa)

Wbm0370	glycyl-tRNA synthetase, alpha subunit (279 aa)

Cluster 41

Number of proteins: 21

Average blastp percent identity: 52%

Average blastp sequence coverage: 96%

AM510	thioredoxin reductase (325 aa)

APH_0658	thioredoxin-disulfide reductase (321 aa)

BH11660	Thioredoxin reductase (317 aa)

Bfl387	thioredoxin reductase (323 aa)

BR1499	thioredoxin reductase (324 aa)

BU314	thioredoxin reductase (319 aa)

CP0444	thioredoxin reductase (311 aa)

CBU1193	thioredoxin reductase (320 aa)

CBU0716	pyridine nucleotide-disulfide oxidoreductase (306 aa)

ECH_0735	thioredoxin-disulfide reductase (318 aa)

ERGA_CDS_03500	Thioredoxin reductase (319 aa)

Erum3470	thioredoxin reductase (318 aa)

NSE_0558	thioredoxin-disulfide reductase (314 aa)

RC0618	thioredoxin reductase [EC:1.6.4.5] (310 aa)

RP445	THIOREDOXIN REDUCTASE (trxB1) (310 aa)

RT0432	thioredoxin-disulfide reductase (310 aa)

TW210	putative thioredoxin reductase (373 aa)

trxB	Purines, pyrimidines, nucleosides, and nucleotides (314 aa)

WD0755	thioredoxin-disulfide reductase (315 aa)

Wbm0566	thioredoxin-disulfide reductase (318 aa)

Cluster 42

Number of proteins: 21

Average blastp percent identity: 50%

Average blastp sequence coverage: 91%

AM1020	inorganic phosphatase protein (175 aa)

APH_1101	inorganic pyrophosphatase (178 aa)

BH01690	Inorganic pyrophosphatase (176 aa)

Bfl091	inorganic pyrophosphatase (179 aa)

BR1993	inorganic pyrophosphatase (176 aa)

BU088	inorganic pyrophosphatase (182 aa)

CP0948	inorganic pyrophosphatase (215 aa)

CBU0628	inorganic pyrophosphatase (175 aa)

ECH_1014	inorganic pyrophosphatase (172 aa)

ERGA_CDS_08190	Inorganic pyrophosphatase (188 aa)

Erum7840	inorganic pyrophosphatase (173 aa)

NSE_0734	inorganic pyrophosphatase (172 aa)

RC0897	inorganic pyrophosphatase [EC:3.6.1.1] (173 aa)

RP589	INORGANIC PYROPHOSPHATASE (ppa) (172 aa)

RT0578	inorganic pyrophosphatase (178 aa)

TW154	inorganic pyrophosphatase (164 aa)

TW307	inorganic pyrophosphatase (164 aa)

ppa	Central intermediary metabolism (179 aa)

WD1263	inorganic pyrophosphatase (168 aa)

Wbm0583	inorganic pyrophosphatase (171 aa)

Cluster 43

Number of proteins: 21

Average blastp percent identity: 50%

Average blastp sequence coverage: 91%

AM601	GTP-binding protein (348 aa)

AM858	hypothetical protein (328 aa)

APH_0694	GTP-binding protein, GTP1/Obg family (352 aa)

BH01560	GTP-binding protein (340 aa)

Bfl095	probable GTP-binding protein (305 aa)

BR1845	GTP-binding protein, GTP1/OBG family (341 aa)

BU389	hypothetical 43.3 kD GTP-binding protein in dacB-rpmA intergenic region (F390) (334 aa)

CP0208	GTP1/OBG family protein (343 aa)

CBU0387	GTP-binding protein, GTP1/Obg family (339 aa)

ECH_0543	GTP-binding protein, GTP1/Obg family (340 aa)

ERGA_CDS_04970	Conserved hypothetical protein (340 aa)

Erum4850	conserved hypothetical GTP-binding protein (340 aa)

NSE_0444	GTP-binding protein, GTP1/Obg family (341 aa)

RC1307	GTP-binding protein (362 aa)

RP843	unknown (331 aa)

RT0831	GTP-binding protein (330 aa)

TW471	GTP-binding protein (445 aa)

yhbZ	Unknown function (337 aa)

WD0493	GTP-binding protein (340 aa)

Wbm0120	GTP-binding protein (340 aa)

Cluster 44

Number of proteins: 21

Average blastp percent identity: 50%

Average blastp sequence coverage: 93%

AM644	DNA gyrase subunit B (793 aa)

APH_0716	DNA gyrase, B subunit (793 aa)

BH00410	DNA gyrase subunit B (810 aa)

Bfl017	DNA gyrase subunit B (806 aa)

BR0125	DNA gyrase, B subunit (810 aa)

BU010	DNA gyrase subunit B (803 aa)

CP0031	DNA gyrase, subunit B (602 aa)

CP0484	DNA gyrase, subunit B (805 aa)

CBU0004	DNA gyrase, B subunit (808 aa)

ECH_0620	DNA gyrase, B subunit (798 aa)

ERGA_CDS_04380	DNA gyrase subunit B (798 aa)

Erum4260	DNA gyrase subunit B (798 aa)

NSE_0721	DNA gyrase, B subunit (783 aa)

RC0884	DNA gyrase subunit B [EC:5.99.1.3] (807 aa)

RP580	DNA GYRASE SUBUNIT B (gyrB2) (807 aa)

RT0569	DNA topoisomerase II subunit B (DNA gyrase subunit B) (807 aa)

TW005	DNA gyrase subunit B (649 aa)

gyrB	DNA metabolism (803 aa)

WD0112	DNA gyrase, B subunit (795 aa)

Wbm0764	DNA gyrase, B subunit (768 aa)

Cluster 45

Number of proteins: 21

Average blastp percent identity: 48%

Average blastp sequence coverage: 87%

AM1274	translation initiation factor IF-3 (177 aa)

AM1311	translation initiation factor IF-3 (85 aa)

APH_1263	translation initiation factor IF-3 (172 aa)

BH00490	Translation initiation factor IF-3 (172 aa)

Bfl352	translation initiation factor IF-3 (175 aa)

BR2117	translation initiation factor IF-3 (134 aa)

BU126	translation initiation factor IF-3 (179 aa)

CP0865	translation initiation factor 3 (186 aa)

CBU1325	translation initiation factor IF-3 (169 aa)

ECH_0007	translation initiation factor IF-3 (173 aa)

ERGA_CDS_09330	Translation initiation factor IF-3 (173 aa)

Erum8900	translation initiation factor IF-3 (173 aa)

NSE_0293	translation initiation factor IF-3 (189 aa)

RC0765	translation initiation factor IF-3 (157 aa)

RP531	TRANSLATION INITIATION FACTOR IF-3 (infC) (185 aa)

RT0518	translation initiation factor IF-3 (185 aa)

TW164	translation initiation factor IF-3 (182 aa)

infC	Protein synthesis (196 aa)

WD0977	translation initiation factor IF-3 (172 aa)

Wbm0473	translation initiation factor IF-3 (144 aa)

Cluster 46

Number of proteins: 21

Average blastp percent identity: 47%

Average blastp sequence coverage: 87%

AM507	prolyl-tRNA synthetase (425 aa)

APH_0647	prolyl-tRNA synthetase (426 aa)

BH08790	Prolyl-tRNA synthetase (441 aa)

Bfl289	prolyl-tRNA synthetase (581 aa)

BR0822	prolyl-tRNA synthetase (442 aa)

BU239	prolyl-tRNA synthetase (572 aa)

CP0254	prolyl-tRNA synthetase (568 aa)

CBU0081	prolyl-tRNA synthetase (566 aa)

ECH_0624	conserved domain protein (41 aa)

ECH_0740	prolyl-tRNA synthetase (424 aa)

ERGA_CDS_03470	Prolyl-tRNA synthetase (426 aa)

Erum3440	prolyl-tRNA synthetase (426 aa)

NSE_0367	prolyl-tRNA synthetase (436 aa)

RC0528	prolyl-tRNA synthetase [EC:6.1.1.15] (426 aa)

RP384	PROLYL-TRNA SYNTHETASE (proS) (432 aa)

RT0372	proline--tRNA ligase (428 aa)

TW183	prolyl-tRNA synthetase (613 aa)

proS	Protein synthesis (561 aa)

WD0813	prolyl-tRNA synthetase (422 aa)

Wbm0077	prolyl-tRNA synthetase (425 aa)

Cluster 47

Number of proteins: 21

Average blastp percent identity: 47%

Average blastp sequence coverage: 92%

AM561	hypothetical protein (341 aa)

AM572	O-sialoglycoprotein endopeptidase (168 aa)

APH_0626	metalloendopeptidase, putative, glycoprotease family (342 aa)

BH16110	O-sialoglycoprotein endopeptidase (364 aa)

Bfl059	possible glycoprotease (353 aa)

BR1888	O-sialoglycoprotein endopeptidase (359 aa)

BU058	O-sialoglycoprotein endopeptidase (336 aa)

CP0573	O-sialoglycoprotein endopeptidase (344 aa)

CBU1240	O-sialoglycoprotein endopeptidase (339 aa)

ECH_0644	metalloendopeptidase, putative, glycoprotease family (349 aa)

ERGA_CDS_04140	Probable O-sialoglycoprotein endopeptidase (Glycoprotease) (348 aa)

Erum4060	o-sialoglycoprotein endopeptidase (348 aa)

NSE_0236	metalloendopeptidase, putative, glycoprotease family (329 aa)

RC0061	sialoglycoprotease [EC:3.4.24.57] (344 aa)

RP037	PROBABLE O-SIALOGLYCOPROTEIN ENDOPEPTIDASE (gcp) (387 aa)

RT0093	O-sialoglycoprotein endopeptidase (387 aa)

TW591	O-syaloglycoprotein endopeptidase (401 aa)

ygjD	Protein fate (340 aa)

WD0699	peptidase, M22 family protein (335 aa)

Wbm0217	peptidase, M22 family protein (335 aa)

Cluster 48

Number of proteins: 21

Average blastp percent identity: 46%

Average blastp sequence coverage: 90%

AM868	cytosine deaminase (154 aa)

APH_0321	cytidine and deoxycytidylate deaminase family protein (147 aa)

BH02600	Nitrogen fixation protein (148 aa)

Bfl537	putative cytosine/adenosine deaminase (159 aa)

BRA0205	cytidine and deoxycytidylate deaminase family protein (157 aa)

BU255	hypothetical protein yfhC (161 aa)

CP0852	cytidine/deoxycytidylate deaminase family protein (155 aa)

CBU0622	zinc-binding domain protein (142 aa)

ECH_0449	cytidine and deoxycytidylate deaminase family protein (140 aa)

ERGA_CDS_05870	Hypothetical protein (62 aa)

ERGA_CDS_05880	Conserved hypothetical protein (putative cytidine deaminase) (145 aa)

Erum5690	putative deaminase (145 aa)

NSE_0085	cytidine/deoxycytidylate deaminase family protein (144 aa)

RC1285	cytosine deaminase [EC:3.5.4.1] (168 aa)

RP831	unknown (148 aa)

RT0819	cytosine deaminase (148 aa)

TW751	unknown (170 aa)

yfhC	Unknown function (151 aa)

WD0469	cytidine and deoxycytidylate deaminase family protein (135 aa)

Wbm0168	cytidine and deoxycytidylate deaminase family protein (143 aa)

Cluster 49

Number of proteins: 21

Average blastp percent identity: 46%

Average blastp sequence coverage: 89%

AM447	hypothetical protein (262 aa)

APH_0528	hydrolase, TatD family (270 aa)

APH_0530	hydrolase, TatD family, truncation (79 aa)

BH08490	hypothetical protein (257 aa)

Bfl399	putative deoxyribonuclease (259 aa)

BR0996	hydrolase, TatD family (263 aa)

BU355	hypothetical protein (264 aa)

CP1085	mttC protein, putative (261 aa)

CBU0502	hydrolase, putative (225 aa)

ECH_0793	hydrolase, TatD family (260 aa)

ERGA_CDS_03030	Conserved hypothetical protein (putative deoxyribonuclease) (261 aa)

Erum3030	putative deoxyribonuclease (261 aa)

NSE_0357	hydrolase, TatD family (268 aa)

RC1045	unknown (306 aa)

RP682	unknown (293 aa)

RT0677	conserved hypothetical protein (293 aa)

TW325	deoxyribonuclease (328 aa)

ycfH	Unknown function (264 aa)

WD1120	deoxyribonuclease, TatD family (255 aa)

Wbm0245	deoxyribonuclease, TatD family (254 aa)

Cluster 50

Number of proteins: 21

Average blastp percent identity: 45%

Average blastp sequence coverage: 91%

AM446	leucyl-tRNA synthetase (829 aa)

APH_0532	hypothetical protein (44 aa)

APH_0534	leucyl-tRNA synthetase (837 aa)

BH15390	Leucyl-tRNA synthetase (880 aa)

Bfl313	leucyl-tRNA synthetase (867 aa)

BR1807	leucyl-tRNA synthetase (877 aa)

BU444	leucyl-tRNA synthetase (859 aa)

CP0618	leucyl-tRNA synthetase (820 aa)

CBU0559	leucyl-tRNA synthetase (820 aa)

ECH_0794	leucyl-tRNA synthetase (829 aa)

ERGA_CDS_03010	Leucyl-tRNA synthetase (830 aa)

Erum3010	leucyl-tRNA synthetase (830 aa)

NSE_0506	leucyl-tRNA synthetase (851 aa)

RC0585	leucyl-tRNA synthetase [EC:6.1.1.4] (835 aa)

RP421	LEUCYL-TRNA SYNTHETASE (leuS) (828 aa)

RT0407	leucine--tRNA ligase (828 aa)

TW385	leucyl-tRNA synthetase (806 aa)

leuS	Protein synthesis (864 aa)

WD0060	leucyl-tRNA synthetase (838 aa)

Wbm0605	leucyl-tRNA synthetase (865 aa)

Cluster 51

Number of proteins: 21

Average blastp percent identity: 44%

Average blastp sequence coverage: 92%

AM224	alanyl-tRNA synthetase (882 aa)

APH_1059	alanyl-tRNA synthetase (876 aa)

APH_1096	alanyl-tRNA synthetase domain protein (94 aa)

BH10220	Alanyl-tRNA synthetase (887 aa)

Bfl168	alanyl-tRNA synthetase (891 aa)

BR1201	alanyl-tRNA synthetase (885 aa)

BU403	alanyl-tRNA synthetase (878 aa)

CP0974	alanyl-tRNA synthetase (872 aa)

CBU1052	alanyl-tRNA synthetase (865 aa)

ECH_0981	alanyl-tRNA synthetase (887 aa)

ERGA_CDS_01420	Alanyl-tRNA synthetase (887 aa)

Erum1500	alanyl-tRNA synthetase (887 aa)

NSE_0156	alanyl-tRNA synthetase (848 aa)

RC1327	alanyl-tRNA synthetase [EC:6.1.1.7] (878 aa)

RP856	ALANYL-TRNA SYNTHETASE (alaS) (877 aa)

RT0845	alanine--tRNA ligase (877 aa)

TW376	alanyl-tRNA synthetase (880 aa)

alaS	Protein synthesis (873 aa)

WD0862	alanyl-tRNA synthetase (877 aa)

Wbm0361	alanyl-tRNA synthetase (840 aa)

Cluster 52

Number of proteins: 21

Average blastp percent identity: 44%

Average blastp sequence coverage: 86%

AM177	guanylate kinase (244 aa)

APH_0147	guanylate kinase, truncation (95 aa)

APH_0170	guanylate kinase (210 aa)

BH05390	Guanylate kinase (222 aa)

Bfl616	guanylate kinase (210 aa)

BR0464	guanylate kinase (220 aa)

BU434	guanylate kinase (207 aa)

CP0653	guanylate kinase (205 aa)

CBU0301	guanylate kinase (206 aa)

ECH_0322	guanylate kinase (209 aa)

ERGA_CDS_06990	Guanylate kinase (209 aa)

Erum6740	guanylate kinase (209 aa)

NSE_0726	guanylate kinase (206 aa)

RC1194	guanylate kinase [EC:2.7.4.8] (229 aa)

RP765	GUANYLATE KINASE (gmk) (197 aa)

RT0752	guanylate kinase (197 aa)

TW363	guanylate kinase (304 aa)

gmk	Purines, pyrimidines, nucleosides, and nucleotides (207 aa)

WD0439	guanylate kinase (205 aa)

Wbm0580	guanylate kinase (195 aa)

Cluster 53

Number of proteins: 21

Average blastp percent identity: 43%

Average blastp sequence coverage: 76%

AM708	signal peptidase (239 aa)

APH_0802	signal peptidase I (243 aa)

BH05080	Signal peptidase I (270 aa)

Bfl541	signal peptidase I (332 aa)

BR0660	signal peptidase I (260 aa)

BU259	signal peptidase I (314 aa)

CP0664	signal peptidase I (636 aa)

CBU1504	signal peptidase I (256 aa)

CBU1099	signal peptidase I (259 aa)

ECH_0690	signal peptidase I (235 aa)

ERGA_CDS_03800	Signal peptidase I (238 aa)

Erum3720	prokaryotic type I signal peptidase (238 aa)

NSE_0784	signal peptidase I (252 aa)

RC0156	signal peptidase I [EC:3.4.21.89] (266 aa)

RP116	SIGNAL PEPTIDASE I (lepB) (264 aa)

RT0020	signal peptidase I (264 aa)

TW456	signal peptidase I (230 aa)

lepB	Protein fate (313 aa)

WD0893	signal peptidase I (248 aa)

Wbm0760	signal peptidase I (248 aa)

Cluster 54

Number of proteins: 21

Average blastp percent identity: 43%

Average blastp sequence coverage: 90%

AM1236	N-formylmethionylaminoacyl-tRNA deformylase (185 aa)

APH_1372	peptide deformylase (187 aa)

APH_1375	peptide deformylase, truncation (91 aa)

BH00760	Polypeptide deformylase (176 aa)

Bfl219	polypeptide deformylase (175 aa)

BRA1035	polypeptide deformylase (187 aa)

BU496	polypeptide deformylase (173 aa)

CP0783	peptide deformylase (186 aa)

CBU0993	peptide deformylase (170 aa)

ECH_0073	peptide deformylase (188 aa)

ERGA_CDS_00440	Peptide deformylase (181 aa)

Erum0540	putative peptide deformylase 1 (181 aa)

NSE_0392	peptide deformylase (186 aa)

RC0278	polypeptide deformylase [EC:3.5.1.31] (175 aa)

RP208	METHIONYL-TRNA DEFORMYLASE (def) (175 aa)

RT0197	peptide deformylase (175 aa)

TW310	formylmethionine deformylase (228 aa)

def	Protein fate (152 aa)

WD0165	peptide deformylase (179 aa)

Wbm0114	peptide deformylase (179 aa)

Cluster 55

Number of proteins: 21

Average blastp percent identity: 43%

Average blastp sequence coverage: 86%

AM956	cytosol aminopeptidase (534 aa)

APH_0231	cytosol aminopeptidase (510 aa)

BH02040	Leucyl aminopeptidase (455 aa)

Bfl035	cytosol aminopeptidase (504 aa)

BR2181	cytosol aminopeptidase family protein (460 aa)

BR0689	cytosol aminopeptidase family protein (497 aa)

BU367	aminopeptidase A/I (499 aa)

CP0370	leucine aminopeptidase (499 aa)

CBU0572	cytosol aminopeptidase family protein (458 aa)

ECH_0369	cytosol aminopeptidase (500 aa)

ERGA_CDS_06600	Probable cytosol aminopeptidase (Aminopeptidase A) (500 aa)

Erum6380	cytosol aminopeptidase (500 aa)

NSE_0475	cytosol aminopeptidase (506 aa)

RC0184	aminopeptidase A [EC:3.4.11.1] (500 aa)

RP142	AMINOPEPTIDASE A (pepA) (500 aa)

RT0131	leucyl aminopeptidase (500 aa)

TW501	aminopeptidase A/I (522 aa)

pepA	Protein fate (501 aa)

WD0054	cytosol aminopeptidase (500 aa)

Wbm0630	cytosol aminopeptidase (488 aa)

Cluster 56

Number of proteins: 21

Average blastp percent identity: 41%

Average blastp sequence coverage: 85%

AM568	translation initiation factor IF-2 (189 aa)

AM735	translation initiation factor IF-2 (832 aa)

APH_0446	translation initiation factor IF-2 (823 aa)

BH02150	Translation initiation factor (845 aa)

Bfl104	protein chain initiation factor 2 (897 aa)

BR2165	translation initiation factor IF-2 (959 aa)

BU377	translation initiation factor IF-2 (864 aa)

CP0440	translation initiation factor 2 (890 aa)

CBU1432	translation initiation factor IF-2 (803 aa)

ECH_0563	translation initiation factor IF-2 (830 aa)

ERGA_CDS_04810	Translation initiation factor IF-2 (856 aa)

Erum4690	translation initiation factor IF-2 (856 aa)

NSE_0522	translation initiation factor IF-2 (779 aa)

RC0816	translation initiation factor IF-2 (831 aa)

RP552	TRANSLATION INITIATION FACTOR IF-2 (infB) (831 aa)

RT0539	translation initiation factor IF-2 (831 aa)

TW157	translation initiation factor IF-2 (803 aa)

infB	Protein synthesis (841 aa)

WD1318	translation initiation factor IF-2 (774 aa)

Wbm0537	translation initiation factor IF-2 (777 aa)

Cluster 57

Number of proteins: 21

Average blastp percent identity: 40%

Average blastp sequence coverage: 79%

AM460	60 kD inner-membrane protein (647 aa)

APH_0540	inner membrane protein, 60 kDa (568 aa)

APH_0541	membrane protein, putative (122 aa)

BH12410	hypothetical protein (612 aa)

Bfl012	putative Preprotein translocase subunit YidC (558 aa)

BRA1023	inner-membrane protein, 60 kDa (610 aa)

BU015	60 kD inner-membrane protein (532 aa)

CP0448	inner membrane protein, putative (795 aa)

CBU1920	inner-membrane protein, 60kDa (566 aa)

ECH_0782	inner-membrane protein, 60 kDa (579 aa)

ERGA_CDS_03140	60 kD inner-membrane protein (575 aa)

Erum3140	putative integral membrane protein (575 aa)

NSE_0402	inner membrane protein, 60 kDa (566 aa)

RC0074	60 kD inner-membrane protein (560 aa)

RP048	60 KD INNER-MEMBRANE PROTEIN (yidC) (560 aa)

RT0082	60 kDa inner membrane protein (558 aa)

TW805	stage III sporulation protein J-like protein (333 aa)

yidC	Unknown function (540 aa)

WD0237	60 kd inner-membrane protein (569 aa)

Wbm0188	60 kd inner-membrane protein (572 aa)

Cluster 58

Number of proteins: 21

Average blastp percent identity: 35%

Average blastp sequence coverage: 95%

AM1014	DNA polymerase III beta chain (379 aa)

APH_1097	DNA polymerase III, beta subunit (375 aa)

BH01190	DNA polymerase III, beta chain (373 aa)

Bfl016	DNA polymerase III beta-subunit (367 aa)

BR0002	DNA polymerase III, beta subunit (397 aa)

BU011	DNA polymerase III beta chain (366 aa)

CP0419	DNA polymerase III, beta subunit (366 aa)

CBU0002	DNA polymerase III, beta subunit (369 aa)

ECH_1009	DNA polymerase III, beta subunit (369 aa)

ECH_1010	DNA polymerase III, beta subunit domain protein (182 aa)

ERGA_CDS_08230	DNA polymerase III, beta chain (375 aa)

Erum7880	DNA polymerase III, beta subunit (375 aa)

NSE_0170	DNA polymerase III, beta subunit (372 aa)

RC0583	DNA polymerase III beta chain [EC:2.7.7.7] (379 aa)

RP419	DNA POLYMERASE III, BETA CHAIN (dnaN) (381 aa)

RT0405	DNA-directed DNA polymerase III beta subunit (381 aa)

TW002	DNA polymerase III beta chain (377 aa)

dnaN	DNA metabolism (369 aa)

WD1067	DNA polymerase III, beta subunit (365 aa)

Wbm0429	DNA polymerase III, beta subunit (393 aa)

Cluster 59

Number of proteins: 21

Average blastp percent identity: 34%

Average blastp sequence coverage: 87%

AM013	GRPE protein (164 aa)

APH_0036	co-chaperone GrpE (211 aa)

BH00560	Heat shock protein (hsp-70 cofactor) grpE (220 aa)

Bfl544	heat shock protein GrpE (195 aa)

BR0171	heat shock protein GrpE (230 aa)

BU184	heat shock protein grpE 2 (188 aa)

BU252	heat shock protein grpE 1 (194 aa)

CP0252	heat shock protein GrpE, putative (184 aa)

CBU1293	heat shock protein GrpE (204 aa)

ECH_0168	co-chaperone GrpE (203 aa)

ERGA_CDS_01060	Similar to yeast GrpE protein (HSP-70 cofactor) (202 aa)

Erum1130	GrpE protein (199 aa)

NSE_0212	co-chaperone GrpE (187 aa)

RC0977	grpE protein (178 aa)

RP629	GRPE PROTEIN (grpE) (178 aa)

RT0620	HSP-70 cofactor (178 aa)

TW749	HSP-70 cofactor GrpE (189 aa)

grpE	Protein fate (223 aa)

WD0800	heat shock protein GrpE (189 aa)

Wbm0533	co-chaperone GrpE (168 aa)

Cluster 60

Number of proteins: 21

Average blastp percent identity: 34%

Average blastp sequence coverage: 81%

AM436	hypothetical protein (197 aa)

APH_0782	Sua5/YciO/YrdC/YwlC family protein (200 aa)

BH04240	hypothetical protein (320 aa)

Bfl220	conserved hypothetical protein (189 aa)

BR0405	Sua5/YciO/YrdC family protein (323 aa)

BU494	hypothetical protein (175 aa)

CP0489	Sua5/YciO/YrdC family protein (286 aa)

CBU0909	Sua5/YciO/YrdC/YwlC family protein (204 aa)

CBU2003	Sua5/YciO/YrdC/YwlC family protein (186 aa)

ECH_0802	Sua5/YciO/YrdC/YwlC family protein (194 aa)

ERGA_CDS_02950	Conserved hypothetical protein (putative translation factor SUA5 family) (199 aa)

Erum2950	conserved hypothetical protein (199 aa)

NSE_0579	Sua5/YciO/YrdC/YwlC family protein (195 aa)

RC1315	unknown (312 aa)

RP848	unknown (313 aa)

RT0837	conserved hypothetical protein (313 aa)

TW433	unknown (225 aa)

yrdC	Unknown function (194 aa)

WD0820	Sua5/YciO/YrdC/YwlC family protein (195 aa)

Wbm0638	Sua5/YciO/YrdC/YwlC family protein (208 aa)

Cluster 61

Number of proteins: 20

Average blastp percent identity: 71%

Average blastp sequence coverage: 98%

AM249	30S ribosomal protein S12 (124 aa)

APH_1035	ribosomal protein S12 (124 aa)

BH10560	30S ribosomal protein s12 (123 aa)

Bfl567	30S ribosomal subunit protein S12 (124 aa)

BR1238	ribosomal protein S12 (123 aa)

BU529	30S ribosomal protein S12 (124 aa)

CP0200	ribosomal protein S12 (123 aa)

CBU0233	ribosomal protein S12 (124 aa)

ECH_0963	ribosomal protein S12 (123 aa)

ERGA_CDS_01550	30S ribosomal protein S12 (123 aa)

Erum1630	30S ribosomal protein S12 (123 aa)

NSE_0689	ribosomal protein S12 (123 aa)

RC0172	30S ribosomal protein S12 (129 aa)

RP130	30S RIBOSOMAL PROTEIN S12 (rpsL) (129 aa)

RT0119	30S ribosomal protein S12 (129 aa)

TW677	30S ribosomal protein S12 (124 aa)

rpsL	Protein synthesis (125 aa)

WD0014	ribosomal protein S12 (124 aa)

Wbm0346	ribosomal protein S12 (124 aa)

Cluster 62

Number of proteins: 20

Average blastp percent identity: 65%

Average blastp sequence coverage: 98%

AM842	DNAK protein (645 aa)

APH_0346	chaperone protein DnaK (645 aa)

BH00650	Heat shock protein 70 DnaK (630 aa)

Bfl114	DnaK protein (heat shock protein 70) (643 aa)

BR2125	chaperone protein DnaK (637 aa)

BU153	dnaK protein (637 aa)

CP0251	dnaK protein (660 aa)

CBU1290	chaperone protein dnak (656 aa)

ECH_0471	chaperone protein DnaK (635 aa)

ERGA_CDS_05670	Chaperone protein dnaK (Heat shock protein) (645 aa)

Erum5500	chaperone protein DnaK (645 aa)

NSE_0019	chaperone protein DnaK (636 aa)

RC0233	dnaK protein (627 aa)

RP185	DNAK PROTEIN (dnaK) (627 aa)

RT0176	chaperone protein DnaK (627 aa)

TW750	chaperone protein (603 aa)

dnaK	Protein fate (645 aa)

WD0928	dnaK protein (640 aa)

Wbm0495	chaperone protein DnaK (635 aa)

Cluster 63

Number of proteins: 20

Average blastp percent identity: 64%

Average blastp sequence coverage: 94%

AM596	50S ribosomal protein L27 (90 aa)

APH_0698	ribosomal protein L27 (91 aa)

BH01400	50S ribosomal protein l27 (89 aa)

Bfl094	50S ribosomal subunit protein L27 (85 aa)

BR1849	ribosomal protein L27 (89 aa)

BU388	50S ribosomal protein L27 (84 aa)

CP0207	ribosomal protein L27 (84 aa)

CBU0386	ribosomal protein L27 (90 aa)

ECH_0546	ribosomal protein L27 (88 aa)

ERGA_CDS_04940	50S ribosomal protein L27 (88 aa)

Erum4820	50S ribosomal protein L27 (88 aa)

NSE_0897	ribosomal protein L27 (82 aa)

RC1163	50S ribosomal protein L27 (86 aa)

RP752	50S RIBOSOMAL PROTEIN L27 (rpmA) (86 aa)

RT0737	50S ribosomal protein L27 (86 aa)

TW472	50S ribosomal protein L27 (82 aa)

rpmA	Protein synthesis (87 aa)

WD0135	ribosomal protein L27 (89 aa)

Wbm0700	ribosomal protein L27 (85 aa)

Cluster 64

Number of proteins: 20

Average blastp percent identity: 62%

Average blastp sequence coverage: 99%

AM901	50S ribosomal protein L14 (119 aa)

APH_0290	ribosomal protein L14 (119 aa)

BH10410	50S ribosomal protein l14 (122 aa)

Bfl201	50S ribosomal subunit protein L14 (123 aa)

BR1223	ribosomal protein L14 (122 aa)

BU514	50S ribosomal protein L14 (122 aa)

CP0110	ribosomal protein L14 (122 aa)

CBU0248	ribosomal protein L14 (122 aa)

ECH_0419	ribosomal protein L14 (119 aa)

ERGA_CDS_06190	50S ribosomal protein L14 (119 aa)

Erum5980	50S ribosomal protein L14 (119 aa)

NSE_0276	ribosomal protein L14 (119 aa)

RC0996	50S ribosomal protein L14 (122 aa)

RP649	50S RIBOSOMAL PROTEIN L14 (rplN) (122 aa)

RT0641	50S ribosomal protein L14 (122 aa)

TW544	50S ribosomal protein L14 (123 aa)

rplN	Protein synthesis (123 aa)

WD0671	ribosomal protein L14 (119 aa)

Wbm0331	ribosomal protein L14 (119 aa)

Cluster 65

Number of proteins: 20

Average blastp percent identity: 61%

Average blastp sequence coverage: 95%

AM310	ATP-dependent clp protease ATP-binding subunit (405 aa)

APH_0969	ATP-dependent Clp protease, ATP-binding subunit ClpX (415 aa)

BH05890	ATP-dependent clp protease ATP-binding subunit clpX (424 aa)

Bfl247	ATP-dependent Clp protease ATP-binding subunit ClpX (426 aa)

BR1108	ATP-dependent Clp protease, ATP-binding subunit ClpX (424 aa)

BU476	ATP-dependent clp protease ATP-binding subunit clpX (429 aa)

CP1023	ATP-dependent Clp protease, ATP-binding regulatory subunit ClpX (421 aa)

CBU0739	ATP-dependent Clp protease, ATP-binding subunit ClpX (422 aa)

ECH_0900	ATP-dependent Clp protease, ATP-binding subunit ClpX (406 aa)

ERGA_CDS_01980	ATP-dependent clp protease ATP-binding subunit ClpX (406 aa)

Erum2010	ATP-dependent Clp protease ATP-binding subunit ClpX (406 aa)

NSE_0753	ATP-dependent Clp protease, ATP-binding subunit ClpX (400 aa)

RC1068	ATP-dependent clp protease ATP-binding subunit clpX (425 aa)

RP692	ATP-DEPENDENT CLP PROTEASE ATP-BINDING SUBUNIT CLPX (clpX) (425 aa)

RT0687	ATP-dependent Clp protease ATP and substrate binding subunit ClpX (425 aa)

TW479	ATP-dependent Clp protease ATP-binding subunit (423 aa)

clpX	Protein fate (415 aa)

WD0318	ATP-dependent Clp protease, ATP-binding subunit ClpX (425 aa)

Wbm0552	ATP-dependent Clp protease, ATP-binding subunit ClpX (426 aa)

Cluster 66

Number of proteins: 20

Average blastp percent identity: 61%

Average blastp sequence coverage: 98%

AM909	50S ribosomal protein L2 (277 aa)

APH_0283	ribosomal protein L2 (276 aa)

BH10480	50S ribosomal protein l2 (277 aa)

Bfl194	50S ribosomal subunit protein L2 (276 aa)

BR1230	ribosomal protein L2 (277 aa)

BU521	50S ribosomal protein L2 (273 aa)

CP0103	ribosomal protein L2 (284 aa)

CBU0241	ribosomal protein L2 (275 aa)

ECH_0412	ribosomal protein L2 (276 aa)

ERGA_CDS_06260	50S ribosomal protein L2 (276 aa)

Erum6040	50S ribosomal protein L2 (276 aa)

NSE_0269	ribosomal protein L2 (279 aa)

RC1003	50S ribosomal protein L2 (273 aa)

RP656	50S RIBOSOMAL PROTEIN L2 (rplB) (273 aa)

RT0648	50S ribosomal protein L2 (273 aa)

TW551	50S ribosomal protein L2 (277 aa)

rplB	Protein synthesis (275 aa)

WD0678	ribosomal protein L2 (274 aa)

Wbm0338	ribosomal protein L2 (274 aa)

Cluster 67

Number of proteins: 20

Average blastp percent identity: 60%

Average blastp sequence coverage: 99%

AM528	GTP-binding protein (599 aa)

APH_0765	GTP-binding protein LepA (598 aa)

BH00710	GTP-binding protein lepA (601 aa)

Bfl542	GTP-binding protein LepA (599 aa)

BRA1039	GTP-binding protein LepA (602 aa)

BU260	GTP-binding protein lepA (610 aa)

CP0399	GTP-binding protein Lepa (602 aa)

CBU1505	GTP-binding protein LepA (614 aa)

ECH_0724	GTP-binding protein LepA (598 aa)

ERGA_CDS_03590	GTP-binding protein LepA (598 aa)

Erum3560	GTP-binding protein LepA (598 aa)

NSE_0771	GTP-binding protein LepA (596 aa)

RC0369	GTP-binding protein lepA (600 aa)

RP275	GTP-BINDING PROTEIN LEPA (lepA) (600 aa)

RT0266	GTP-binding protein LepA (600 aa)

TW278	GTP-binding protein LepA (601 aa)

lepA	Unknown function (599 aa)

WD0477	GTP-binding protein LepA (598 aa)

Wbm0667	GTP-binding protein LepA (563 aa)

Cluster 68

Number of proteins: 20

Average blastp percent identity: 60%

Average blastp sequence coverage: 99%

AM944	60 kD chaperonin (549 aa)

APH_0240	60 kDa chaperonin (550 aa)

BH13530	Chaperonin protein groEL (547 aa)

Bfl071	GroEL protein (547 aa)

BRA0195	chaperonin, 60 kDa (546 aa)

BU019	60 kD chaperonin (548 aa)

CP0638	60 kDa chaperonin (544 aa)

CBU1718	chaperonin, 60 kDa (552 aa)

ECH_0365	chaperonin, 60 kd (550 aa)

ERGA_CDS_06640	60 kDa chaperonin (Protein Cpn60) (groEL protein) (551 aa)

Erum6420	60 kDa chaperonin GroEL (551 aa)

NSE_0642	60 kDa chaperonin (548 aa)

RC0968	60 kD chaperonin (548 aa)

RP626	60 KD CHAPERONIN (groEL) (550 aa)

RT0617	60 kDa chaperonin (550 aa)

TW441	60 kDa chaperonin 2 (540 aa)

mopA	Protein fate (546 aa)

WD0307	chaperonin, 60 kDa (549 aa)

Wbm0350	chaperonin GroEL (530 aa)

Cluster 69

Number of proteins: 20

Average blastp percent identity: 59%

Average blastp sequence coverage: 93%

AM1119	cell division protein (610 aa)

APH_1179	ATP-dependent metalloprotease FtsH (611 aa)

BH14750	Cell division protein ftsH (715 aa)

Bfl098	cell division protein FtsH (644 aa)

BR1691	cell division protein FtsH (644 aa)

BU382	cell division protein ftsh (596 aa)

CP0857	cell division protein FtsH, putative (910 aa)

CBU1352	ATP-dependent metalloprotease FtsH (647 aa)

ECH_1098	ATP-dependent metalloprotease FtsH (610 aa)

ERGA_CDS_08850	Cell division protein ftsh homolog (611 aa)

Erum8430	cell division protein FtsH (611 aa)

NSE_0423	ATP-dependent metalloprotease FtsH (636 aa)

RC0068	cell division protein ftsH [EC:3.4.24.-] (637 aa)

RP043	CELL DIVISION PROTEIN FTSH (ftsH) (637 aa)

RT0087	cell division protein FtsH (637 aa)

TW588	cell division protein FtsH (666 aa)

hflB	Cellular processes (638 aa)

WD1253	cell division protein FtsH (613 aa)

Wbm0150	cell division protein FtsH (609 aa)

Cluster 70

Number of proteins: 20

Average blastp percent identity: 57%

Average blastp sequence coverage: 97%

AM890	30S ribosomal protein S13 (123 aa)

APH_0301	ribosomal protein S13 (123 aa)

BH10290	30S ribosomal protein s13 (122 aa)

Bfl213	30S ribosomal subunit protein S13 (118 aa)

BR1211	ribosomal protein S13 (122 aa)

BU502	30S ribosomal protein S13 (118 aa)

CP0119	ribosomal protein S13 (122 aa)

CBU0260	ribosomal protein S13/S18 (119 aa)

ECH_0430	ribosomal protein S13 (123 aa)

ERGA_CDS_06080	30S ribosomal protein S13 (123 aa)

Erum5870	30S ribosomal protein S13 (123 aa)

NSE_0287	ribosomal protein S13 (123 aa)

RC0984	30S ribosomal protein S13 (125 aa)

RP637	30S RIBOSOMAL PROTEIN S13 (rpsM) (125 aa)

RT0629	30S ribosomal protein S13 (125 aa)

TW530	30S ribosomal protein S13 (124 aa)

rpsM	Protein synthesis (118 aa)

WD0660	ribosomal protein S13 (122 aa)

Wbm0320	ribosomal protein S13p/S18e (122 aa)

Cluster 71

Number of proteins: 20

Average blastp percent identity: 57%

Average blastp sequence coverage: 94%

AM913	30S ribosomal protein S10 (105 aa)

APH_0279	ribosomal protein S10 (105 aa)

BH10520	30S ribosomal protein s10 (102 aa)

Bfl190	30S ribosomal subunit protein S10 (103 aa)

BR1234	ribosomal protein S10 (102 aa)

BU525	30S ribosomal protein S10 (103 aa)

CP0203	ribosomal protein S10 (105 aa)

CBU0237	ribosomal protein S10 (110 aa)

ECH_0408	ribosomal protein S10 (108 aa)

ERGA_CDS_06300	30S ribosomal protein S10 (110 aa)

Erum6080	30S ribosomal protein S10 (111 aa)

NSE_0265	ribosomal protein S10 (103 aa)

RC1007	30S ribosomal protein S10 (105 aa)

RP660	30S RIBOSOMAL PROTEIN S10 (rpsJ) (105 aa)

RT0652	30S ribosomal protein S10 (105 aa)

TW555	30S ribosomal protein S10 (102 aa)

rpsJ	Protein synthesis (103 aa)

WD0682	ribosomal protein S10 (109 aa)

Wbm0342	ribosomal protein S10 (124 aa)

Cluster 72

Number of proteins: 20

Average blastp percent identity: 57%

Average blastp sequence coverage: 94%

AM054	cyanelle 50S ribosomal protein L20 (128 aa)

APH_0060	ribosomal protein L20 (128 aa)

BH00830	50S ribosomal protein l20 (133 aa)

Bfl354	50S ribosomal subunit protein L20 (118 aa)

BR2120	ribosomal protein L20 (134 aa)

BU128	50S ribosomal protein L20 (118 aa)

CP0863	ribosomal protein L20 (121 aa)

CBU1323	ribosomal protein L20 (119 aa)

ECH_0197	ribosomal protein L20 (124 aa)

ERGA_CDS_01290	50S ribosomal protein L20 (123 aa)

Erum1370	50S ribosomal protein L20 (123 aa)

NSE_0215	ribosomal protein L20 (117 aa)

RC0935	50S ribosomal protein L20 (117 aa)

RP609	CYANELLE 50S RIBOSOMAL PROTEIN L20 (rplT) (117 aa)

RT0597	50S ribosomal protein L20 (117 aa)

TW166	50S ribosomal protein L20 (136 aa)

rplT	Protein synthesis (117 aa)

WD0865	ribosomal protein L20 (121 aa)

Wbm0657	ribosomal protein L20 (121 aa)

Cluster 73

Number of proteins: 20

Average blastp percent identity: 57%

Average blastp sequence coverage: 95%

AM906	30S ribosomal protein S3 (211 aa)

APH_0286	ribosomal protein S3 (211 aa)

BH10450	30S ribosomal protein s3 (236 aa)

Bfl197	30S ribosomal subunit protein S3 (235 aa)

BR1227	ribosomal protein S3 (236 aa)

BU518	30S ribosomal protein S3 (233 aa)

CP0106	ribosomal protein S3 (223 aa)

CBU0244	ribosomal protein S3 (227 aa)

ECH_0415	ribosomal protein S3 (211 aa)

ERGA_CDS_06230	30S ribosomal protein S3 (217 aa)

Erum6010	30S ribosomal protein S3 (211 aa)

NSE_0272	ribosomal protein S3 (211 aa)

RC1000	30S ribosomal protein S3 (217 aa)

RP653	30S RIBOSOMAL PROTEIN S3 (rpsC) (217 aa)

RT0645	30S ribosomal protein S3 (217 aa)

TW548	30S ribosomal protein S3 (220 aa)

rpsC	Protein synthesis (236 aa)

WD0675	ribosomal protein S3 (204 aa)

Wbm0335	ribosomal protein S3 (206 aa)

Cluster 74

Number of proteins: 20

Average blastp percent identity: 56%

Average blastp sequence coverage: 97%

AM905	50S ribosomal protein L16 (142 aa)

APH_0287	ribosomal protein L16 (137 aa)

BH10440	50S ribosomal protein l16 (137 aa)

Bfl198	50S ribosomal subunit protein L16 (133 aa)

BR1226	ribosomal protein L16 (137 aa)

BU517	50S ribosomal protein L16 (136 aa)

CP0107	ribosomal protein L16 (138 aa)

CBU0245	ribosomal protein L16 (137 aa)

ECH_0416	ribosomal protein L16 (136 aa)

ERGA_CDS_06220	50S ribosomal protein L16 (136 aa)

Erum6000	50S ribosomal protein L16 (136 aa)

NSE_0273	ribosomal protein L16 (139 aa)

RC0999	50S ribosomal protein L16 (136 aa)

RP652	50S RIBOSOMAL PROTEIN L16 (rplP) (136 aa)

RT0644	50S ribosomal protein L16 (136 aa)

TW547	50S ribosomal protein L16 (143 aa)

rplP	Protein synthesis (136 aa)

WD0674	ribosomal protein L16 (137 aa)

Wbm0334	ribosomal protein L16 (137 aa)

Cluster 75

Number of proteins: 20

Average blastp percent identity: 56%

Average blastp sequence coverage: 96%

AM908	30S ribosomal protein S19 (93 aa)

APH_0284	ribosomal protein S19 (94 aa)

BH10470	30S ribosomal protein s19 (92 aa)

Bfl195	30S ribosomal subunit protein S19 (93 aa)

BR1229	ribosomal protein S19 (92 aa)

BU520	30S ribosomal protein S19 (92 aa)

CP0104	ribosomal protein S19 (88 aa)

CBU0242	ribosomal protein S19 (95 aa)

ECH_0413	ribosomal protein S19 (93 aa)

ERGA_CDS_06250	30S ribosomal protein S19 (93 aa)

Erum6030	30S ribosomal protein S19 (93 aa)

NSE_0270	ribosomal protein S19 (91 aa)

RC1002	30S ribosomal protein S19 (92 aa)

RP655	30S RIBOSOMAL PROTEIN S19 (rpsS) (92 aa)

RT0647	30S ribosomal protein S19 (92 aa)

TW550	30S ribosomal protein S19 (144 aa)

rpsS	Protein synthesis (142 aa)

WD0677	ribosomal protein S19 (94 aa)

Wbm0337	ribosomal protein S19 (94 aa)

Cluster 76

Number of proteins: 20

Average blastp percent identity: 56%

Average blastp sequence coverage: 97%

AM899	50S ribosomal protein L5 (179 aa)

APH_0292	ribosomal protein L5 (177 aa)

BH10390	50S ribosomal protein l5 (185 aa)

Bfl203	50S ribosomal subunit protein L5 (190 aa)

BR1221	ribosomal protein L5 (185 aa)

BU512	50S ribosomal protein L5 (179 aa)

CP0112	ribosomal protein L5 (180 aa)

CBU0250	ribosomal protein L5 (182 aa)

ECH_0421	ribosomal protein L5 (177 aa)

ERGA_CDS_06170	50S ribosomal protein L5 (177 aa)

Erum5960	50S ribosomal protein L5 (177 aa)

NSE_0278	ribosomal protein L5 (177 aa)

RC0994	50S ribosomal protein L5 (179 aa)

RP647	50S RIBOSOMAL PROTEIN L5 (rplE) (179 aa)

RT0639	50S ribosomal protein L5 (179 aa)

TW542	50S ribosomal protein L5 (183 aa)

rplE	Protein synthesis (178 aa)

WD0669	ribosomal protein L5 (177 aa)

Wbm0329	ribosomal protein L5 (177 aa)

Cluster 77

Number of proteins: 20

Average blastp percent identity: 55%

Average blastp sequence coverage: 91%

AM795	translation initiation factor IF-1 (111 aa)

APH_0395	translation initiation factor IF-1 (81 aa)

BH14140	Translation initiation factor if-1 (72 aa)

Bfl388	initiation factor IF-1 (72 aa)

BR0249	translation initiation factor IF-1 (72 aa)

BU315	translation initiation factor IF-1 (72 aa)

CP0702	translation initiation factor 1 (73 aa)

CBU1195	translation initiation factor IF-1 (83 aa)

ECH_0511	translation initiation factor IF-1 (82 aa)

ERGA_CDS_05260	Translation initiation factor IF-1 (82 aa)

Erum5110	putative translation initiation factor IF-1 (82 aa)

NSE_0882	translation initiation factor IF-1 (93 aa)

RC1265	translation initiation factor IF-1 (71 aa)

RP814	TRANSLATION INITIATION FACTOR IF-1 (infA) (71 aa)

RT0802	translation initiation factor IF-1 (71 aa)

TW532	translation initiation factor IF-1 (73 aa)

infA	Protein synthesis (72 aa)

WD0334	translation initiation factor IF-1 (87 aa)

Wbm0191	translation initiation factor IF-1 (87 aa)

Cluster 78

Number of proteins: 20

Average blastp percent identity: 55%

Average blastp sequence coverage: 97%

AM767	peptide chain release factor 1 (359 aa)

APH_0420	peptide chain release factor 1 (359 aa)

BH01970	Peptide chain release factor 1 (359 aa)

Bfl348	peptide chain release factor 1 (RF-1) (358 aa)

BR1869	peptide chain release factor 1 (359 aa)

BU171	peptide chain release factor 1 (361 aa)

CP0660	peptide chain release factor 1 (357 aa)

CBU1965	peptide chain release factor 1 (361 aa)

ECH_0597	peptide chain release factor 1 (359 aa)

ERGA_CDS_04620	Peptide chain release factor 1 (RF-1) (359 aa)

Erum4500	peptide release factor 1 (359 aa)

NSE_0406	peptide chain release factor 1 (367 aa)

RC0762	peptide chain release factor RF-1 (355 aa)

RP529	PEPTIDE CHAIN RELEASE FACTOR 1 (prfA) (355 aa)

RT0516	peptide chain release factor 1 (RF-1) (355 aa)

TW435	peptide chain release factor RF-1 (347 aa)

prfA	Protein synthesis (364 aa)

WD0247	peptide chain release factor 1 (359 aa)

Wbm0358	peptide chain release factor 1 (359 aa)

Cluster 79

Number of proteins: 20

Average blastp percent identity: 54%

Average blastp sequence coverage: 94%

AM1120	preprotein translocase subunit (896 aa)

APH_1178	preprotein translocase, SecA subunit (873 aa)

BH01990	Preprotein translocase secA subunit (905 aa)

Bfl148	preprotein translocase SecA subunit (840 aa)

BR1945	preprotein translocase, SecA subunit (906 aa)

BU201	preprotein translocase secA subunit (875 aa)

CP1028	preprotein translocase SecA subunit (970 aa)

CBU0147	preprotein translocase, SecA subunit (913 aa)

ECH_1149	preprotein translocase, SecA subunit (862 aa)

ERGA_CDS_09200	Preprotein translocase secA subunit (870 aa)

Erum8780	preprotein translocase SecA subunit (870 aa)

NSE_0232	preprotein translocase, SecA subunit (804 aa)

RC0879	preprotein translocase secA subunit (906 aa)

RP575	PREPROTEIN TRANSLOCASE SECA SUBUNIT (secA) (906 aa)

RT0564	preprotein translocase SecA subunit (905 aa)

TW130	preprotein translocase SecA subunit (847 aa)

secA	Protein fate (832 aa)

WD0549	preprotein translocase, SecA subunit (886 aa)

Wbm0266	preprotein translocase, SecA subunit (815 aa)

Cluster 80

Number of proteins: 20

Average blastp percent identity: 54%

Average blastp sequence coverage: 96%

AM888	30S ribosomal protein S11 (113 aa)

APH_0302	ribosomal protein S11 (124 aa)

BH10280	30S ribosomal protein s11 (129 aa)

Bfl214	30S ribosomal subunit protein S11 (130 aa)

BR1210	ribosomal protein S11 (129 aa)

BU501	30S ribosomal protein S11 (131 aa)

CP0120	ribosomal protein S11 (133 aa)

CBU0261	ribosomal protein S11 (123 aa)

ECH_0431	ribosomal protein S11 (126 aa)

ERGA_CDS_06070	30S ribosomal protein S11 (127 aa)

Erum5860	30S ribosomal protein S11 (127 aa)

NSE_0288	ribosomal protein S11 (120 aa)

RC0983	30S ribosomal protein S11 (127 aa)

RP636	30S RIBOSOMAL PROTEIN S11 (rpsK) (127 aa)

RT0628	30S ribosomal protein S11 (127 aa)

TW529	30S ribosomal protein S11 (130 aa)

rpsK	Protein synthesis (131 aa)

WD0659	ribosomal protein S11 (128 aa)

Wbm0319	ribosomal protein S11 (128 aa)

Cluster 81

Number of proteins: 20

Average blastp percent identity: 54%

Average blastp sequence coverage: 89%

AM1022	30S ribosomal protein S9 (153 aa)

APH_1107	ribosomal protein S9 (149 aa)

BH07810	30S ribosomal protein s9 (161 aa)

Bfl050	30S ribosomal subunit protein S9 (130 aa)

BR0790	ribosomal protein S9 (158 aa)

BU390	30S ribosomal protein S9 (130 aa)

CP0516	ribosomal protein S9 (134 aa)

CBU1748	ribosomal protein S9 (139 aa)

ECH_1018	ribosomal protein S9 (152 aa)

ERGA_CDS_08170	30S ribosomal protein S9 (153 aa)

Erum7820	30S ribosomal protein S9 (153 aa)

NSE_0839	ribosomal protein S9 (145 aa)

RC0316	30S ribosomal protein S9 (159 aa)

RP234	30S RIBOSOMAL PROTEIN S9 (rpsI) (161 aa)

RT0226	30S ribosomal protein S9 (161 aa)

TW145	30S ribosomal protein S9 (154 aa)

rpsI	Protein synthesis (129 aa)

WD0066	ribosomal protein S9 (151 aa)

Wbm0573	ribosomal protein S9 (148 aa)

Cluster 82

Number of proteins: 20

Average blastp percent identity: 54%

Average blastp sequence coverage: 91%

AM894	30S ribosomal protein S5 (174 aa)

APH_0297	ribosomal protein S5 (173 aa)

BH10340	30S ribosomal protein s5 (182 aa)

Bfl208	30S ribosomal subunit protein S5 (177 aa)

BR1216	ribosomal protein S5 (186 aa)

BU507	30S ribosomal protein S5 (167 aa)

CP0116	ribosomal protein S5 (165 aa)

CBU0255	ribosomal protein S5 (168 aa)

ECH_0426	ribosomal protein S5 (174 aa)

ERGA_CDS_06120	30S ribosomal protein S5 (177 aa)

Erum5910	30S ribosomal protein S5 (174 aa)

NSE_0283	ribosomal protein S5 (172 aa)

RC0989	30S ribosomal protein S5 (176 aa)

RP642	30S RIBOSOMAL PROTEIN S5 (rpsE) (175 aa)

RT0634	30S ribosomal protein S5 (175 aa)

TW538	30S ribosomal protein S5 (286 aa)

rpsE	Protein synthesis (166 aa)

WD0664	ribosomal protein S5 (170 aa)

Wbm0324	ribosomal protein S5 (134 aa)

Cluster 83

Number of proteins: 20

Average blastp percent identity: 54%

Average blastp sequence coverage: 96%

AM340	50S ribosomal protein L33 (56 aa)

APH_0925	ribosomal protein L33 (56 aa)

BH08140	50S ribosomal protein l33 (55 aa)

Bfl611	50S ribosomal subunit protein L33 (53 aa)

BRA0609	ribosomal protein L33 (55 aa)

BU085	50S ribosomal protein L33 (55 aa)

CP0511	ribosomal protein L33 (52 aa)

CBU0290	ribosomal protein L33 (70 aa)

ECH_0876	ribosomal protein L33 (56 aa)

ERGA_CDS_02170	50S ribosomal protein L33 (59 aa)

Erum2190	50S ribosomal protein L33 (56 aa)

NSE_0257	ribosomal protein L33 (59 aa)

RC1360	50S ribosomal protein L33 (56 aa)

RP879	50S RIBOSOMAL PROTEIN L33 (rpmG) (56 aa)

RT0870	50S ribosomal protein L33 (56 aa)

TW114	50S ribosomal protein L33 (56 aa)

rpmG	Protein synthesis (55 aa)

WD0930	ribosomal protein L33 (66 aa)

Wbm0497	ribosomal protein L33 (66 aa)

Cluster 84

Number of proteins: 20

Average blastp percent identity: 54%

Average blastp sequence coverage: 97%

AM250	30S ribosomal protein S7 (160 aa)

APH_1034	ribosomal protein S7 (160 aa)

BH10550	30S ribosomal protein s7 (156 aa)

Bfl566	30S ribosomal subunit protein S7 (157 aa)

BR1237	ribosomal protein S7 (156 aa)

BU528	30S ribosomal protein S7 (156 aa)

CP0201	ribosomal protein S7 (157 aa)

CBU0234	ribosomal protein S7 (191 aa)

ECH_0962	ribosomal protein S7 (160 aa)

ERGA_CDS_01560	30S ribosomal protein S7 (160 aa)

Erum1640	30S ribosomal protein S7 (160 aa)

NSE_0688	ribosomal protein S7 (175 aa)

RC0173	30S ribosomal protein S7 (160 aa)

RP131	30S RIBOSOMAL PROTEIN S7 (rpsG) (160 aa)

RT0120	30S ribosomal protein S7 (160 aa)

TW676	30S ribosomal protein S7 (156 aa)

rpsG	Protein synthesis (156 aa)

WD0015	ribosomal protein S7 (159 aa)

Wbm0345	ribosomal protein S7 (159 aa)

Cluster 85

Number of proteins: 20

Average blastp percent identity: 53%

Average blastp sequence coverage: 81%

AM161.5	small subunit ribosomal protein S18 (75 aa)

APH_0152	ribosomal protein S18 (96 aa)

BH05310	30S ribosomal protein s18 (82 aa)

Bfl086	30S ribosomal subunit protein S18 (78 aa)

BR0454	ribosomal protein S18 (82 aa)

BU563	30S ribosomal protein S18 (75 aa)

CP0907	ribosomal protein S18 (82 aa)

CBU0865	ribosomal protein S18 (73 aa)

ECH_0309	ribosomal protein S18, putative (95 aa)

ERGA_CDS_07130	30S ribosomal protein S18 (95 aa)

Erum6860	30S ribosomal protein S18 (95 aa)

NSE_0061	ribosomal protein S18 (73 aa)

RC0065	30S ribosomal protein S18 (95 aa)

RP040	30S RIBOSOMAL PROTEIN S18 (rpsR) (95 aa)

RT0090	30S ribosomal protein S18 (95 aa)

TW106	30S ribosomal protein S18 (83 aa)

rpsR	Protein synthesis (75 aa)

WD0782	ribosomal protein S18 (91 aa)

Wbm0501	ribosomal protein S18 (91 aa)

Cluster 86

Number of proteins: 20

Average blastp percent identity: 53%

Average blastp sequence coverage: 97%

AM018	CTP synthase (560 aa)

APH_0038	CTP synthase (541 aa)

BH05700	CTP synthase (542 aa)

Bfl156	CTP synthetase (550 aa)

BR1134	CTP synthase (542 aa)

BU416	CTP synthase (545 aa)

CP0526	CTP synthase (537 aa)

CBU1682	CTP synthase (555 aa)

ECH_0171	CTP synthase (532 aa)

ERGA_CDS_01090	CTP synthase (543 aa)

Erum1160	CTP synthase (540 aa)

NSE_0877	CTP synthase (548 aa)

RC0523	ctp synthase [EC:6.3.4.2] (537 aa)

RP378	CTP SYNTHASE (pyrG) (586 aa)

RT0367	CTP synthase (583 aa)

TW100	CTP synthase (545 aa)

pyrG	Purines, pyrimidines, nucleosides, and nucleotides (552 aa)

WD0468	CTP synthase (529 aa)

Wbm0169	CTP synthase (540 aa)

Cluster 87

Number of proteins: 20

Average blastp percent identity: 53%

Average blastp sequence coverage: 86%

AM792	30S ribosomal protein S2 (365 aa)

APH_0397	ribosomal protein S2 (296 aa)

BH06220	30S ribosomal protein s2 (257 aa)

Bfl271	30S ribosomal protein S2 (231 aa)

BR1162	ribosomal protein S2 (256 aa)

BU231	30S ribosomal protein S2 (244 aa)

CP0050	ribosomal protein S2 (277 aa)

CBU1386	ribosomal protein S2 (313 aa)

ECH_0514	ribosomal protein S2 (288 aa)

ERGA_CDS_05240	30S ribosomal protein S2 (286 aa)

Erum5090	30S ribosomal protein S2 (286 aa)

NSE_0948	ribosomal protein S2 (298 aa)

RC0112	30S ribosomal protein S2 (295 aa)

RP086	30S RIBOSOMAL PROTEIN S2 (rpsB) (296 aa)

RT0050	30S ribosomal protein S2 (295 aa)

TW449	30S ribosomal protein S2 (308 aa)

rpsB	Protein synthesis (240 aa)

WD0532	ribosomal protein S2 (282 aa)

Wbm0808	ribosomal protein S2 (284 aa)

Cluster 88

Number of proteins: 20

Average blastp percent identity: 53%

Average blastp sequence coverage: 95%

AM1023	50S ribosomal protein L13 (170 aa)

APH_1108	ribosomal protein L13 (158 aa)

BH07820	50S ribosomal protein l13 (154 aa)

Bfl049	50S ribosomal subunit protein L13 (139 aa)

BR0791	ribosomal protein L13 (154 aa)

BU391	50S ribosomal protein L13 (142 aa)

CP0515	ribosomal protein L13 (149 aa)

CBU1749	ribosomal protein L13 (142 aa)

ECH_1019	ribosomal protein L13 (156 aa)

ERGA_CDS_08160	50S ribosomal protein L13 (156 aa)

Erum7810	50S ribosomal protein L13 (156 aa)

NSE_0840	ribosomal protein L13 (152 aa)

RC0315	50S ribosomal protein L13 (155 aa)

RP233	50S RIBOSOMAL PROTEIN L13 (rplM) (155 aa)

RT0225	50S ribosomal protein L13 (155 aa)

TW144	50S ribosomal protein L13 (142 aa)

rplM	Protein synthesis (142 aa)

WD0067	ribosomal protein L13 (152 aa)

Wbm0574	ribosomal protein L13 (153 aa)

Cluster 89

Number of proteins: 20

Average blastp percent identity: 52%

Average blastp sequence coverage: 83%

AM059	30S ribosomal protein S16 (89 aa)

APH_0054	ribosomal protein S16 (96 aa)

BH16020	30S ribosomal protein s16 (127 aa)

Bfl173	30S ribosomal subunit protein S16 (82 aa)

BR1824	ribosomal protein S16 (134 aa)

BU394	30S ribosomal protein S16 (79 aa)

CP0657	ribosomal protein S16 (119 aa)

CBU0445	ribosomal protein S16 (137 aa)

ECH_0192	ribosomal protein S16 (87 aa)

ERGA_CDS_01240	30S ribosomal protein S16 (87 aa)

Erum1320	30S ribosomal protein S16 (87 aa)

NSE_0123	ribosomal protein S16 (85 aa)

RC1359	30S ribosomal protein S16 (111 aa)

RP878	30S RIBOSOMAL PROTEIN S16 (rpsP) (111 aa)

RT0869	30S ribosomal protein S16 (111 aa)

TW461	30S ribosomal protein S16 (148 aa)

rpsP	Protein synthesis (80 aa)

WD0798	ribosomal protein S16 (106 aa)

Wbm0531	ribosomal protein S16 (106 aa)

Cluster 90

Number of proteins: 20

Average blastp percent identity: 52%

Average blastp sequence coverage: 93%

AM525	30S ribosomal protein S15 (93 aa)

APH_0768	ribosomal protein S15 (93 aa)

BH02110	30S ribosomal protein s15 (89 aa)

Bfl107	30S ribosomal subunit protein S15 (90 aa)

BR2168	ribosomal protein S15 (89 aa)

BU374	30S ribosomal protein S15 (89 aa)

CP0854	ribosomal protein S15 (89 aa)

CBU0851	ribosomal protein S15 (89 aa)

ECH_0727	ribosomal protein S15 (93 aa)

ERGA_CDS_03560	30S ribosomal protein S15 (93 aa)

Erum3530	30S ribosomal protein S15 (93 aa)

NSE_0058	ribosomal protein S15 (86 aa)

RC0664	30S ribosomal protein S15 (91 aa)

RP503	30S RIBOSOMAL PROTEIN S15 (rpsO) (91 aa)

RT0488	30S ribosomal protein S15 (91 aa)

TW422	30S ribosomal protein S15 (142 aa)

rpsO	Protein synthesis (89 aa)

WD0905	ribosomal protein S15 (90 aa)

Wbm0020	ribosomal protein S15 (90 aa)

Cluster 91

Number of proteins: 20

Average blastp percent identity: 52%

Average blastp sequence coverage: 90%

AM886	50S ribosomal protein L17 (134 aa)

APH_0304	ribosomal protein L17 (132 aa)

BH10260	50S ribosomal protein l17 (142 aa)

Bfl217	50S ribosomal subunit protein L17 (125 aa)

BR1208	ribosomal protein L17 (142 aa)

BU498	50S ribosomal protein L17 (130 aa)

CP0122	ribosomal protein L17 (142 aa)

CBU0264	ribosomal protein L17 (126 aa)

ECH_0433	ribosomal protein L17 (133 aa)

ERGA_CDS_06050	50S ribosomal protein L17 (128 aa)

Erum5840	50S ribosomal protein L17 (128 aa)

NSE_0290	ribosomal protein L17 (117 aa)

RC0981	50S ribosomal protein L17 (136 aa)

RP634	50S RIBOSOMAL PROTEIN L17 (rplQ) (136 aa)

RT0626	50S ribosomal protein L17 (137 aa)

TW527	50S ribosomal protein L17 (179 aa)

rplQ	Protein synthesis (122 aa)

WD0657	ribosomal protein L17 (142 aa)

Wbm0317	ribosomal protein L17 (142 aa)

Cluster 92

Number of proteins: 20

Average blastp percent identity: 51%

Average blastp sequence coverage: 99%

AM898	small ribosomal protein S14 (82 aa)

APH_0293	ribosomal protein S14p/S29e (101 aa)

BH10380	30S ribosomal protein s14 (101 aa)

Bfl204	30S ribosomal subunit protein S14 (101 aa)

BR1220	ribosomal protein S14 (101 aa)

BU511	30S ribosomal protein S14 (101 aa)

CP0924	ribosomal protein S14 (101 aa)

CBU0251	ribosomal protein S14 (99 aa)

ECH_0422	ribosomal protein S14p (101 aa)

ERGA_CDS_06160	30S ribosomal protein S14 (102 aa)

Erum5950	30S ribosomal protein S14 (101 aa)

NSE_0279	ribosomal protein S14 (101 aa)

RC0993	30S ribosomal protein S14 (101 aa)

RP646	CYANELLE 30S RIBOSOMAL PROTEIN S14 (rpsN) (101 aa)

RT0638	30S ribosomal protein S14 (101 aa)

TW115	30S ribosomal protein S14 (101 aa)

rpsN	Protein synthesis (101 aa)

WD0668	ribosomal protein S14 (102 aa)

Wbm0328	ribosomal protein S14 (102 aa)

Cluster 93

Number of proteins: 20

Average blastp percent identity: 51%

Average blastp sequence coverage: 92%

AM053	ribosomal protein L35 (66 aa)

APH_0061	ribosomal protein L35 (66 aa)

BH00820	50S ribosomal protein l35 (67 aa)

Bfl353	50S ribosomal subunit protein L35 (65 aa)

BR2119	ribosomal protein L35 (66 aa)

BU127	50S ribosomal protein L35 (65 aa)

CP0864	ribosomal protein L35 (64 aa)

CBU1324	ribosomal protein L35 (64 aa)

ECH_0198	ribosomal protein L35 (66 aa)

ERGA_CDS_01300	50S ribosomal protein L35 (66 aa)

Erum1380	50S ribosomal protein L35 (66 aa)

NSE_0214	ribosomal protein L35 (68 aa)

RC0934	50S ribosomal protein L35 (89 aa)

RP608	50S RIBOSOMAL PROTEIN L35 (rpmI) (67 aa)

RT0596	50S ribosomal protein L35 (68 aa)

TW165	50S ribosomal protein L35 (65 aa)

rpmI	Protein synthesis (66 aa)

WD0864	ribosomal protein L35 (68 aa)

Wbm0658	ribosomal protein L35 (68 aa)

Cluster 94

Number of proteins: 20

Average blastp percent identity: 51%

Average blastp sequence coverage: 91%

AM903	30S ribosomal protein S17 (77 aa)

APH_0289	ribosomal protein S17 (76 aa)

BH10420	30S ribosomal protein s17 (79 aa)

Bfl200	30S ribosomal subunit protein S17 (85 aa)

BR1224	ribosomal protein S17 (80 aa)

BU515	30S ribosomal protein S17 (83 aa)

CP0109	ribosomal protein S17 (86 aa)

CBU0247	ribosomal protein S17 (89 aa)

ECH_0418	ribosomal protein S17 (75 aa)

ERGA_CDS_06200	30S ribosomal protein S17 (82 aa)

Erum5990	30S ribosomal protein S17 (74 aa)

NSE_0275	ribosomal protein S17 (74 aa)

RC0997	30S ribosomal protein S17 (82 aa)

RP650	30S RIBOSOMAL PROTEIN S17 (rpsQ) (77 aa)

RT0642	30S ribosomal protein S17 (82 aa)

TW545	30S ribosomal protein S17 (86 aa)

rpsQ	Protein synthesis (92 aa)

WD0672	ribosomal protein S17 (78 aa)

Wbm0332	ribosomal protein S17 (77 aa)

Cluster 95

Number of proteins: 20

Average blastp percent identity: 51%

Average blastp sequence coverage: 96%

AM1355	hypothetical protein (364 aa)

APH_1285	GTP-binding protein YchF (363 aa)

BH11880	GTP-binding protein (367 aa)

Bfl344	putative ATP/GTP-binding protein (348 aa)

BR1537	GTP-binding protein, putative (367 aa)

BU191	GTP-binding protein (362 aa)

CP0436	GTP-binding protein, YcfH family (364 aa)

CBU1842	GTP-binding protein YchF (363 aa)

ECH_0154	GTP-binding protein YchF (362 aa)

ERGA_CDS_00940	Conserved hypothetical protein (363 aa)

Erum1010	conserved hypothetical GTP-binding protein (363 aa)

NSE_0910	GTP-binding protein YchF (350 aa)

RC0928	probable GTP-binding protein (379 aa)

RP604	unknown (365 aa)

RT0592	probable GTP-binding protein (365 aa)

TW639	GTP-binding protein (344 aa)

ychF	Hypothetical proteins (357 aa)

WD0442	GTP-binding protein YchF (364 aa)

Wbm0159	GTP-binding protein YchF (371 aa)

Cluster 96

Number of proteins: 20

Average blastp percent identity: 51%

Average blastp sequence coverage: 92%

AM424	single-strand binding protein (179 aa)

APH_0465	single-strand binding protein (159 aa)

BH10130	Single-strand binding protein (ssb) (172 aa)

Bfl028	single-strand DNA-binding protein (158 aa)

BR1102	single-stranded DNA-binding protein family (168 aa)

BU545	single-strand binding protein (171 aa)

CP0369	single-strand binding protein (160 aa)

CBU0271	single-strand binding protein (158 aa)

ECH_0815	single-strand binding protein (157 aa)

ERGA_CDS_02840	Single-strand binding protein (177 aa)

Erum2830	single-strand DNA binding protein (156 aa)

NSE_0759	single-strand binding protein (151 aa)

RC1295	single-strand binding protein (152 aa)

RP836	SINGLE-STRAND BINDING PROTEIN (ssb) (152 aa)

RT0824	single-strand binding protein (152 aa)

TW107	single-strand binding protein (186 aa)

ssb	DNA metabolism (167 aa)

WD0774	single-strand binding protein (160 aa)

Wbm0211	single-strand binding protein (137 aa)

Cluster 97

Number of proteins: 20

Average blastp percent identity: 51%

Average blastp sequence coverage: 91%

AM1271	50S ribosomal subunit L19 (134 aa)

APH_1266	ribosomal protein L19 (128 aa)

BH15810	50S ribosomal protein l19 (146 aa)

Bfl176	50S ribosomal subunit protein L19 (117 aa)

BR1907	ribosomal protein L19 (145 aa)

BU397	50S ribosomal protein L19 (115 aa)

CP0655	ribosomal protein L19 (121 aa)

CBU0442	ribosomal protein L19 (115 aa)

ECH_0004	ribosomal protein L19 (125 aa)

ERGA_CDS_09290	50S ribosomal protein L19 (129 aa)

Erum8870	50S ribosomal protein L19 (125 aa)

NSE_0895	ribosomal protein L19 (120 aa)

RC0152	50S ribosomal protein L19 (138 aa)

RP112	50S RIBOSOMAL PROTEIN L19 (rplS) (138 aa)

RT0024	50S ribosomal protein L19 (138 aa)

TW457	50S ribosomal protein L19 (115 aa)

rplS	Protein synthesis (115 aa)

WD1021	ribosomal protein L19 (125 aa)

Wbm0229	ribosomal protein L19 (113 aa)

Cluster 98

Number of proteins: 20

Average blastp percent identity: 50%

Average blastp sequence coverage: 92%

AM805	deoxyuridine 5triphosphate nucleotidohydrolase (147 aa)

APH_0379	deoxyuridine 5'-triphosphate nucleotidohydrolase (146 aa)

BH14690	Deoxyuridine 5 27-triphosphate nucleotidohydrolase (184 aa)

Bfl613	deoxyuridine 5-triphosphate nucleotidohydrolase (151 aa)

BR1675	deoxyuridine 5'-triphosphate nucleotidohydrolase (157 aa)

BU560	deoxyuridine 5-triphosphate nucleotidohydrolase (154 aa)

CP0716	deoxyuridine 5`-triphosphate nucleotidohydrolase (145 aa)

CBU0293	deoxyuridine 5'-triphosphate nucleotidohydrolase (152 aa)

ECH_0501	deoxyuridine 5'triphosphate nucleotidohydrolase (155 aa)

ERGA_CDS_05340	Putative Deoxyuridine 5-triphosphate nucleotidohydrolase (124 aa)

Erum5190	putative deoxyuridine 5-triphosphate nucleotidohydrolase (124 aa)

NSE_0957	deoxyuridine 5'triphosphate nucleotidohydrolase (149 aa)

RC0546	deoxyuridine 5-triphosphate nucleotidohydrolase [EC:3.6.1.23] (148 aa)

RP399	DEOXYURIDINE 5-TRIPHOSPHATE NUCLEOTIDOHYDROLASE (dut) (148 aa)

RT0387	dUTP pyrophosphatase (148 aa)

TW487	deoxyuridine 5-triphosphate nucleotidohydrolase (146 aa)

dut	Purines, pyrimidines, nucleosides, and nucleotides (153 aa)

WD0243	deoxyuridine 5'-triphosphate nucleotidohydrolase (153 aa)

Wbm0355	deoxyuridine 5triphosphate nucleotidohydrolase (141 aa)

Cluster 99

Number of proteins: 20

Average blastp percent identity: 50%

Average blastp sequence coverage: 91%

AM1173	transcription elongation factor greA (174 aa)

APH_1333	transcription elongation factor GreA (166 aa)

BH11700	Transcription elongation factor greA (157 aa)

Bfl096	transcription elongation factor, cleaves 3 nucleotide of paused mRNA (162 aa)

BR1504	transcription elongation factor GreA (157 aa)

BU384	transcription elongation factor greA (159 aa)

CP0004	transcription elongation protein, GreA/GreB family (722 aa)

CBU1280	transcription elongation factor GreA (159 aa)

ECH_0133	transcription elongation factor GreA (162 aa)

ERGA_CDS_00730	Transcription elongation factor greA (166 aa)

Erum0810	transcription elongation factor GreA (162 aa)

NSE_0669	transcription elongation factor GreA (159 aa)

RC1332	transcription elongation factor EF (206 aa)

RP861	TRANSCRIPTION ELONGATION FACTOR GREA (greA) (162 aa)

RT0850	transcription elongation factor GreA (162 aa)

TW680	transcription elongation factor (172 aa)

greA	Transcription (158 aa)

WD0654	transcription elongation factor GreA (164 aa)

Wbm0313	transcription elongation factor GreA (164 aa)

Cluster 100

Number of proteins: 20

Average blastp percent identity: 50%

Average blastp sequence coverage: 95%

AM1273	threonyl-tRNA synthetase (636 aa)

APH_1264	threonyl-tRNA synthetase (615 aa)

BH07660	Threonyl-tRNA synthetase (658 aa)

Bfl351	threonyl-tRNA synthetase (410 aa)

BR1071	threonyl-tRNA synthetase (658 aa)

BU125	threonyl-tRNA synthetase (642 aa)

CP1065	threonyl-tRNA synthetase (635 aa)

CBU1326	threonyl-tRNA synthetase (651 aa)

ECH_0006	threonyl-tRNA synthetase (633 aa)

ERGA_CDS_09320	Threonyl-tRNA synthetase (633 aa)

Erum8890	threonyl-tRNA synthetase (633 aa)

NSE_0294	threonyl-tRNA synthetase (637 aa)

RC0296	threonyl-tRNA synthetase [EC:6.1.1.3] (635 aa)

RP221	THREONYL-TRNA SYNTHETASE (thrS) (635 aa)

RT0212	threonine--tRNA ligase (635 aa)

TW262	threonyl-tRNA synthetase (640 aa)

thrS	Protein synthesis (636 aa)

WD0978	threonyl-tRNA synthetase (633 aa)

Wbm0472	threonyl-tRNA synthetase (632 aa)

Cluster 101

Number of proteins: 20

Average blastp percent identity: 50%

Average blastp sequence coverage: 93%

AM887	DNA-directed RNA polymerase alpha chain (366 aa)

APH_0303	DNA-directed RNA polymerase, alpha subunit (344 aa)

BH10270	DNA-directed RNA polymerase alpha chain (337 aa)

Bfl216	DNA-directed RNA polymerase alpha chain (331 aa)

BR1209	DNA-directed RNA polymerase, alpha subunit (337 aa)

BU499	DNA-directed RNA polymerase alpha chain (329 aa)

CP0121	DNA-directed RNA polymerase, alpha subunit (391 aa)

CBU0263	DNA-directed RNA polymerase, alpha subunit (327 aa)

ECH_0432	DNA-directed RNA polymerase, alpha subunit (341 aa)

ERGA_CDS_06060	DNA-directed RNA polymerase alpha chain (374 aa)

Erum5850	DNA-directed RNA polymerase alpha chain (358 aa)

NSE_0289	DNA-directed RNA polymerase, alpha subunit (333 aa)

RC0982	DNA-directed RNA polymerase alpha chain [EC:2.7.7.6] (340 aa)

RP635	DNA-DIRECTED RNA POLYMERASE ALPHA CHAIN (rpoA) (340 aa)

RT0627	DNA-directed RNA polymerase alpha subunit (340 aa)

TW528	DNA-directed RNA polymerase alpha chain (327 aa)

rpoA	Transcription (328 aa)

WD0658	DNA-directed RNA polymerase, alpha subunit (355 aa)

Wbm0318	DNA-directed RNA polymerase, alpha subunit (355 aa)

Cluster 102

Number of proteins: 20

Average blastp percent identity: 50%

Average blastp sequence coverage: 97%

AM1296	DNAJ protein (379 aa)

APH_0015	chaperone protein DnaJ (382 aa)

BH00660	Heat shock protein DnaJ (381 aa)

Bfl115	DnaJ protein (377 aa)

BR2126	chaperone protein DnaJ (377 aa)

BU152	dnaJ protein (377 aa)

CP0744	dnaJ protein (392 aa)

CBU1289	chaperone protein dnaJ (374 aa)

ECH_0025	chaperone protein DnaJ (380 aa)

ERGA_CDS_09500	Chaperone protein dnaJ (382 aa)

Erum0130	chaperone protein DnaJ (382 aa)

NSE_0535	chaperone protein DnaJ (379 aa)

RC0232	dnaJ protein (373 aa)

RP184	DNAJ PROTEIN (dnaJ) (370 aa)

RT0175	chaperone protein DnaJ (370 aa)

TW280	chaperone protein (348 aa)

dnaJ	Protein fate (374 aa)

WD0040	dnaJ protein (372 aa)

Wbm0785	chaperone protein DnaJ (374 aa)

Cluster 103

Number of proteins: 20

Average blastp percent identity: 50%

Average blastp sequence coverage: 96%

AM179	methylenetetrahydrofolate dehydrogenase (299 aa)

APH_0175	methylenetetrahydrofolate dehydrogenase/methenyltetrahydrofolate cyclohydrolase (304 aa)

BH03940	Methylenetetrahydrofolate dehydrogenase (299 aa)

methylenetetrahydrofolate dehydrogenase methenyltetrahydrofolate cyclohydrolase (296 aa)

BRA0781	FolD bifunctional protein (299 aa)

BU486	methylenetetrahydrofolate dehydrogenase / methenyltetrahydrofolate cyclohydrolase (285 aa)

CP0423	methylenetetrahydrofolate dehydrogenase/methenyltetrahydrofolate cyclohydrolase (286 aa)

CBU0312	folD bifunctional protein (283 aa)

ECH_0324	FolD bifunctional protein (300 aa)

ERGA_CDS_06980	FolD bifunctional protein [Methylenetetrahydrofolate dehydrogenase Methenyltetrahydrofolate cyclohydrolase] (300 aa)

Erum6730	methylenetetrahydrofolate dehydrogenase/methenyltetrahydrofolate cyclohydrolase (300 aa)

NSE_0821	FolD bifunctional protein (298 aa)

RC0636	methylenetetrahydrofolate dehydrogenase [EC:1.5.1.5 3.5.4.9] (288 aa)

RP515	METHYLENETETRAHYDROFOLATE DEHYDROGENASE (folD) (310 aa)

RT0501	methylenetetrahydrofolate dehydrogenase (NADP+) (285 aa)

TW634	methylenetetrahydrofolate dehydrogenase NADP+ (295 aa)

folD	Biosynthesis of cofactors, prosthetic groups, and carrier (283 aa)

WD0555	methylenetetrahydrofolate dehydrogenase/methenyltetrahydrofolate cyclohydrolase (292 aa)

Wbm0073	methylenetetrahydrofolate dehydrogenase/methenyltetrahydrofolate cyclohydrolase (295 aa)

Cluster 104

Number of proteins: 20

Average blastp percent identity: 49%

Average blastp sequence coverage: 94%

AM372	DNA gyrase subunit A (908 aa)

APH_0899	DNA gyrase, A subunit (906 aa)

BH10120	DNA gyrase subunit a (931 aa)

Bfl476	DNA gyrase subunit A (862 aa)

BR1097	DNA gyrase, A subunit (922 aa)

BU180	DNA gyrase subunit A (830 aa)

CP0485	DNA gyrase, subunit A (834 aa)

CBU0524	DNA gyrase, A subunit (848 aa)

ECH_0858	DNA gyrase, A subunit (898 aa)

ERGA_CDS_02420	DNA gyrase subunit A (898 aa)

Erum2420	DNA gyrase subunit A (898 aa)

NSE_0958	DNA gyrase, A subunit (869 aa)

RC0273	DNA gyrase subunit A [EC:5.99.1.3] (905 aa)

RP206	DNA GYRASE SUBUNIT A (gyrA) (905 aa)

RT0196	DNA topoisomerase II subunit A (DNA gyrase subunit A) (905 aa)

TW006	DNA gyrase subunit A (819 aa)

gyrA	DNA metabolism (847 aa)

WD1202	DNA gyrase, A subunit (900 aa)

Wbm0405	DNA gyrase, A subunit (900 aa)

Cluster 105

Number of proteins: 20

Average blastp percent identity: 49%

Average blastp sequence coverage: 94%

AM526	polyribonucleotide nucleotidyltransferase (805 aa)

APH_0767	polyribonucleotide nucleotidyltransferase (838 aa)

BH02100	Polyribonucleotide nucleotidyltransferase (733 aa)

Bfl108	polynucleotide phosphorylase, member of mRNA degradosome (697 aa)

BR2169	polyribonucleotide nucleotidyltransferase (714 aa)

BU373	polyribonucleotide nucleotidyltransferase (707 aa)

CP0855	polyribonucleotide nucleotidyltransferase (694 aa)

CBU0852	polyribonucleotide nucleotidyltransferase (696 aa)

ECH_0726	polyribonucleotide nucleotidyltransferase (796 aa)

ERGA_CDS_03570	Polyribonucleotide nucleotidyltransferase (789 aa)

Erum3540	polyribonucleotide nucleotidyltransferase (789 aa)

NSE_0057	polyribonucleotide nucleotidyltransferase (757 aa)

RC0663	polyribonucleotide nucleotidyltransferase [EC:2.7.7.8] (749 aa)

RP504	POLYRIBONUCLEOTIDE NUCLEOTIDYLTRANSFERASE (pnp) (745 aa)

RT0489	polyribonucleotide nucleotidyltransferase (745 aa)

TW421	polyribonucleotide nucleotidyltransferase (738 aa)

pnp	Transcription (705 aa)

WD0906	polyribonucleotide nucleotidyltransferase (757 aa)

Wbm0019	polyribonucleotide nucleotidyltransferase (755 aa)

Cluster 106

Number of proteins: 20

Average blastp percent identity: 49%

Average blastp sequence coverage: 90%

AM108	50S ribosomal protein L31E (99 aa)

APH_0090	50S ribosomal protein L31, putative (73 aa)

BH15050	50S ribosomal protein l31 (73 aa)

Bfl599	50S ribosomal protein L31 (73 aa)

BR1716	ribosomal protein L31 (73 aa)

BU577	50S ribosomal protein L31 (72 aa)

CP0661	ribosomal protein L31 (109 aa)

CBU0905	ribosomal protein L31 (79 aa)

ECH_0226	ribosomal protein L31 (75 aa)

ERGA_CDS_07790	Conserved hypothetical protein (75 aa)

Erum7480	50S ribosomal protein L31 (75 aa)

NSE_0503	conserved hypothetical protein (76 aa)

RC0137	50S ribosomal protein L31 (78 aa)

RP100	50S RIBOSOMAL PROTEIN L31 (rpmE) (78 aa)

RT0037	50S ribosomal protein L31 (78 aa)

TW314	50S ribosomal protein L31 (83 aa)

rpmE	Protein synthesis (71 aa)

WD0870	ribosomal protein L31, putative (67 aa)

Wbm0401	ribosomal protein L31, putative (70 aa)

Cluster 107

Number of proteins: 20

Average blastp percent identity: 49%

Average blastp sequence coverage: 95%

AM943	10 KD chaperonin (107 aa)

APH_0241	10 kDa chaperonin (94 aa)

BH13540	Chaperonin protein groES (98 aa)

Bfl070	GroES protein (97 aa)

BRA0196	chaperonin, 10 kDa (98 aa)

BU018	10 kD chaperonin (96 aa)

CP0637	10 kDa chaperonin (102 aa)

CBU1719	chaperonin, 10 kDa (96 aa)

ECH_0364	chaperonin, 10 kDa (94 aa)

ERGA_CDS_06650	10 kDa chaperonin (Protein Cpn10) (groES protein) (94 aa)

Erum6430	10 kDa chaperonin GroES (94 aa)

NSE_0643	10 kDa chaperonin (98 aa)

RC0969	10 kD chaperonin (99 aa)

RP627	10 KD CHAPERONIN (groES) (95 aa)

RT0618	10 kDa chaperonin (95 aa)

TW074	10 kDa chaperone (120 aa)

mopB	Protein fate (97 aa)

WD0308	chaperonin, 10 kDa (96 aa)

Wbm0349	chaperonin, 10 kDa (96 aa)

Cluster 108

Number of proteins: 20

Average blastp percent identity: 48%

Average blastp sequence coverage: 98%

AM759	seryl-tRNA synthetase (437 aa)

APH_0427	seryl-tRNA synthetase (428 aa)

BH05610	Seryl-tRNA synthetase (425 aa)

Bfl384	seryl-tRNA synthetase (436 aa)

BR0885	seryl-tRNA synthetase (427 aa)

BU313	seryl-tRNA synthetase (427 aa)

CP0999	seryl-tRNA synthetase (427 aa)

CBU1188	seryl-tRNA synthetase (423 aa)

ECH_0584	seryl-tRNA synthetase (426 aa)

ERGA_CDS_04660	Seryl-tRNA synthetase (425 aa)

Erum4540	seryl-tRNA synthetase (427 aa)

NSE_0569	seryl-tRNA synthetase (415 aa)

RC1216	seryl-tRNA synthetase [EC:6.1.1.11] (425 aa)

RP783	SERYL-TRNA SYNTHETASE (serS) (425 aa)

RT0770	serine--tRNA ligase (425 aa)

TW726	seryl-tRNA synthetase (417 aa)

serS	Protein synthesis (429 aa)

WD0028	seryl-tRNA synthetase (425 aa)

Wbm0464	seryl-tRNA synthetase (425 aa)

Cluster 109

Number of proteins: 20

Average blastp percent identity: 48%

Average blastp sequence coverage: 98%

AM897	30S ribosomal protein S8 (132 aa)

APH_0294	ribosomal protein S8 (133 aa)

BH10370	30S ribosomal protein s8 (132 aa)

Bfl205	30S ribosomal subunit protein S8 (131 aa)

BR1219	ribosomal protein S8 (132 aa)

BU510	30S ribosomal protein S8 (130 aa)

CP0113	ribosomal protein S8 (133 aa)

CBU0252	ribosomal protein S8 (130 aa)

ECH_0423	ribosomal protein S8 (132 aa)

ERGA_CDS_06150	30S ribosomal protein S8 (141 aa)

Erum5940	30S ribosomal protein S8 (132 aa)

NSE_0280	ribosomal protein S8 (131 aa)

RC0992	30S ribosomal protein S8 (132 aa)

RP645	30S RIBOSOMAL PROTEIN S8 (rpsH) (132 aa)

RT0637	30S ribosomal protein S8 (132 aa)

TW541	30S ribosomal protein S8 (132 aa)

rpsH	Protein synthesis (130 aa)

WD0667	ribosomal protein S8 (131 aa)

Wbm0327	ribosomal protein S8 (131 aa)

Cluster 110

Number of proteins: 20

Average blastp percent identity: 48%

Average blastp sequence coverage: 97%

AM258	50S ribosomal protein L11 (147 aa)

APH_1028	ribosomal protein L11 (148 aa)

BH06060	50S ribosomal protein l11 (142 aa)

Bfl561	50S ribosomal subunit protein L11 (146 aa)

BR1248	ribosomal protein L11 (142 aa)

BU038	50S ribosomal protein L11 (142 aa)

CP0698	ribosomal protein L11 (141 aa)

CBU0226	ribosomal protein L11 (145 aa)

ECH_0956	ribosomal protein L11 (147 aa)

ERGA_CDS_01610	50S ribosomal protein L11 (147 aa)

Erum1680	50S ribosomal protein L11 (147 aa)

NSE_0682	ribosomal protein L11 (140 aa)

RC0177	50S ribosomal protein L11 (145 aa)

RP136	50S RIBOSOMAL PROTEIN L11 (rplK) (145 aa)

RT0125	50S ribosomal protein L11 (145 aa)

TW714	50S ribosomal protein L11 (141 aa)

rplK	Protein synthesis (142 aa)

WD0020	ribosomal protein L11 (146 aa)

Wbm0651	50S ribosomal protein (146 aa)

Cluster 111

Number of proteins: 20

Average blastp percent identity: 48%

Average blastp sequence coverage: 95%

AM892	preprotein translocase secY subunit (433 aa)

APH_0299	preprotein translocase, SecY subunit (434 aa)

BH10310	Preprotein translocase secY subunit (448 aa)

Bfl211	preprotein translocase subunit secY (451 aa)

BR1213	preprotein translocase, SecY subunit (446 aa)

BU504	preprotein translocase secY subunit (437 aa)

CP0118	preprotein translocase SecY subunit (462 aa)

CBU0258	preprotein translocase, SecY subunit (442 aa)

ECH_0428	preprotein translocase, SecY subunit (432 aa)

ERGA_CDS_06100	Preprotein translocase secY subunit (432 aa)

Erum5890	preprotein translocase secY subunit (432 aa)

NSE_0285	preprotein translocase, SecY subunit (436 aa)

RC0986	preprotein translocase secY subunit (433 aa)

RP639	PREPROTEIN TRANSLOCASE SECY SUBUNIT (secY) (433 aa)

RT0631	preprotein translocase SecY subunit (433 aa)

TW535	preprotein translocase SecY subunit (441 aa)

prlA	Protein fate (449 aa)

WD0662	preprotein translocase, SecY subunit (442 aa)

Wbm0322	preprotein translocase, SecY subunit (442 aa)

Cluster 112

Number of proteins: 20

Average blastp percent identity: 48%

Average blastp sequence coverage: 91%

AM907	50S ribosomal subunit L22 (112 aa)

APH_0285	ribosomal protein L22 (112 aa)

BH10460	50S ribosomal protein l22 (129 aa)

Bfl196	50S ribosomal subunit protein L22 (109 aa)

BR1228	ribosomal protein L22 (129 aa)

BU519	50S ribosomal protein L22 (110 aa)

CP0105	ribosomal protein L22 (111 aa)

CBU0243	ribosomal protein L22 (115 aa)

ECH_0414	ribosomal protein L22 (110 aa)

ERGA_CDS_06240	50S ribosomal protein L22 (145 aa)

Erum6020	50S ribosomal protein L22 (114 aa)

NSE_0271	ribosomal protein L22 (113 aa)

RC1001	50S ribosomal protein L22 (119 aa)

RP654	50S RIBOSOMAL PROTEIN L22 (rplV) (119 aa)

RT0646	50S ribosomal protein L22 (119 aa)

TW549	50S ribosomal protein L22 (119 aa)

rplV	Protein synthesis (111 aa)

WD0676	ribosomal protein L22 (115 aa)

Wbm0336	ribosomal protein L22 (116 aa)

Cluster 113

Number of proteins: 20

Average blastp percent identity: 48%

Average blastp sequence coverage: 94%

AM912	50S ribosomal protein L3 (232 aa)

APH_0280	ribosomal protein L3 (220 aa)

BH10510	50S ribosomal protein l3 (246 aa)

Bfl191	50S ribosomal subunit protein L3 (219 aa)

BR1233	ribosomal protein L3 (237 aa)

BU524	50S ribosomal protein L3 (209 aa)

CP0100	ribosomal protein L3 (219 aa)

CBU0238	ribosomal protein L3 (217 aa)

ECH_0409	ribosomal protein L3 (231 aa)

ERGA_CDS_06290	50S ribosomal protein L3 (235 aa)

Erum6070	50S ribosomal protein L3 (231 aa)

NSE_0266	ribosomal protein L3 (210 aa)

RC1006	50S ribosomal protein L3 (215 aa)

RP659	50S RIBOSOMAL PROTEIN L3 (rplC) (216 aa)

RT0651	50S ribosomal protein L3 (215 aa)

TW554	50S ribosomal protein L3 (210 aa)

rplC	Protein synthesis (215 aa)

WD0681	ribosomal protein L3 (240 aa)

Wbm0341	ribosomal protein L3 (228 aa)

Cluster 114

Number of proteins: 20

Average blastp percent identity: 48%

Average blastp sequence coverage: 93%

AM1171	small protein B homolog (149 aa)

APH_1331	SsrA-binding protein (149 aa)

BH05010	SSRA-binding protein (158 aa)

Bfl548	small protein B putative tmRNA-binding protein (148 aa)

BR0647	SsrA-binding protein (158 aa)

BU254	small protein B (162 aa)

CP0421	smpB protein (165 aa)

CBU1305	SsrA-binding protein (159 aa)

ECH_0135	SsrA-binding protein (149 aa)

ERGA_CDS_00710	SsrA-binding protein (148 aa)

Erum0790	SsrA-binding protein (148 aa)

NSE_0811	SsrA-binding protein (147 aa)

RC0596	small protein B homolog (152 aa)

RP430	SMALL PROTEIN B HOMOLOG (smpB) (152 aa)

RT0416	tmRNA-binding protein SmpB (152 aa)

TW201	tmRNA (SsrA)-binding protein (211 aa)

smpB	Protein synthesis (160 aa)

WD0767	SsrA-binding protein (149 aa)

Wbm0080	SsrA-binding protein (149 aa)

Cluster 115

Number of proteins: 20

Average blastp percent identity: 48%

Average blastp sequence coverage: 94%

AM301	ribosomal protein S4 (178 aa)

APH_0989	ribosomal protein S4 (204 aa)

BH08390	30S ribosomal protein s4 (205 aa)

Bfl215	30S ribosomal subunit protein S4 (207 aa)

BR0830	ribosomal protein S4 (205 aa)

BU500	30S ribosomal protein S4 (206 aa)

CP0013	ribosomal protein S4 (209 aa)

CBU0262	ribosomal protein S4 (206 aa)

ECH_0912	ribosomal protein S4 (202 aa)

ERGA_CDS_01900	30S ribosomal protein S4 (202 aa)

Erum1940	30S ribosomal protein S4 (202 aa)

NSE_0583	ribosomal protein S4 (205 aa)

RC0468	30S ribosomal protein S4 (205 aa)

RP345	30S RIBOSOMAL PROTEIN S4 (rpsD) (205 aa)

RT0335	30S ribosomal protein S4 (205 aa)

TW378	30S ribosomal protein S4 (261 aa)

rpsD	Protein synthesis (225 aa)

WD0388	ribosomal protein S4 (204 aa)

Wbm0663	ribosomal protein S4 (223 aa)

Cluster 116

Number of proteins: 20

Average blastp percent identity: 47%

Average blastp sequence coverage: 95%

AM900	50S ribosomal protein L24 (105 aa)

APH_0291	ribosomal protein L24 (104 aa)

BH10400	50S ribosomal protein l24 (104 aa)

Bfl202	50S ribosomal subunit protein L24 (106 aa)

BR1222	ribosomal protein L24 (103 aa)

BU513	50S ribosomal protein L24 (104 aa)

CP0111	ribosomal protein L24 (111 aa)

CBU0249	ribosomal protein L24 (107 aa)

ECH_0420	ribosomal protein L24 (109 aa)

ERGA_CDS_06180	50S ribosomal protein L24 (114 aa)

Erum5970	50S ribosomal protein L24 (109 aa)

NSE_0277	ribosomal protein L24 (104 aa)

RC0995	50S ribosomal protein L24 (109 aa)

RP648	50S RIBOSOMAL PROTEIN L24 (rplX) (113 aa)

RT0640	50S ribosomal protein L24 (113 aa)

TW543	50S ribosomal protein L24 (113 aa)

rplX	Protein synthesis (104 aa)

WD0670	ribosomal protein L24 (105 aa)

Wbm0330	ribosomal protein L24 (105 aa)

Cluster 117

Number of proteins: 20

Average blastp percent identity: 47%

Average blastp sequence coverage: 87%

AM490	RNA polymerase sigma factor (632 aa)

APH_0576	RNA polymerase sigma factor RpoD (627 aa)

BH11530	RNA polymerase sigma factor rpod (667 aa)

Bfl056	RNA polymerase sigma-70 factor (617 aa)

BR1479	RNA polymerase sigma-70 factor (672 aa)

BU055	RNA polymerase sigma factor rpoD (612 aa)

CP1116	RNA polymerase sigma factor, sigma-70 family (591 aa)

CBU1596	RNA polymerase sigma factor RpoD (698 aa)

ECH_0760	RNA polymerase sigma factor RpoD (622 aa)

ERGA_CDS_03350	RNA polymerase sigma factor rpoD (622 aa)

Erum3320	RNA polymerase sigma-70 factor (622 aa)

NSE_0312	RNA polymerase sigma factor RpoD (620 aa)

RC1329	RNA polymerase sigma factor (634 aa)

RP858	RNA POLYMERASE SIGMA FACTOR RPOD (rpoD) (635 aa)

RT0847	RNA polymerase sigma factor RpoD (635 aa)

TW499	RNA polymerase sigma factor (387 aa)

rpoD	Transcription (601 aa)

WD1298	RNA polymerase sigma factor RpoD (642 aa)

Wbm0393	RNA polymerase sigma factor RpoD (649 aa)

Cluster 118

Number of proteins: 20

Average blastp percent identity: 47%

Average blastp sequence coverage: 87%

AM339	acyl carrier protein (115 aa)

APH_0929	acyl carrier protein, putative (84 aa)

BH05360	Acyl carrier protein (78 aa)

Bfl403	acyl carrier protein (78 aa)

BR0459	acyl carrier protein (78 aa)

BU352	acyl carrier protein (80 aa)

CP0463	acyl carrier protein (79 aa)

CBU0496	acyl carrier protein (84 aa)

ECH_0881	acyl carrier protein (91 aa)

ERGA_CDS_02140	Putative Acyl carrier protein (92 aa)

Erum2160	acyl carrier protein (92 aa)

NSE_0452	acyl carrier protein (144 aa)

RC1185	acyl carrier protein (86 aa)

RP763	ACYL CARRIER PROTEIN (acpP) (86 aa)

RT0749	acyl carrier protein (81 aa)

TW252	acyl carrier protein (82 aa)

acpP	Fatty acid and phospholipid metabolism (78 aa)

WD1193	acyl carrier protein (86 aa)

Wbm0738	acyl carrier protein (88 aa)

Cluster 119

Number of proteins: 20

Average blastp percent identity: 47%

Average blastp sequence coverage: 93%

AM259	ribosomal protein L1 (217 aa)

APH_1027	ribosomal protein L1 (216 aa)

BH06070	50S ribosomal protein l1 (232 aa)

Bfl560	50S ribosomal subunit protein L1 (235 aa)

BR1247	ribosomal protein L1 (233 aa)

BU037	50S ribosomal protein L1 (231 aa)

CP0697	ribosomal protein L1 (232 aa)

CBU0227	ribosomal protein L1 (232 aa)

ECH_0955	ribosomal protein L1 (220 aa)

ERGA_CDS_01620	50S ribosomal protein L1 (220 aa)

Erum1690	50S ribosomal protein L1 (220 aa)

NSE_0681	ribosomal protein L1 (209 aa)

RC0178	50S ribosomal protein L1 (239 aa)

RP137	50S RIBOSOMAL PROTEIN L1 (rplA) (238 aa)

RT0126	50S ribosomal protein L1 (237 aa)

TW713	50S ribosomal protein L1 (227 aa)

rplA	Protein synthesis (242 aa)

WD0021	ribosomal protein L1 (217 aa)

Wbm0650	ribosomal protein L1 (217 aa)

Cluster 120

Number of proteins: 20

Average blastp percent identity: 46%

Average blastp sequence coverage: 91%

AM1129	cell division protein (305 aa)

APH_1227	signal recognition particle-docking protein FtsY (305 aa)

BH16620	Cell division protein ftsY (416 aa)

Bfl627	cell division protein FtsY (308 aa)

BR1934	signal recognition particle-docking protein FtsY (463 aa)

BU024	cell division protein ftsY (378 aa)

CP0887	signal recognition particle protein FtsY (290 aa)

CBU1903	signal recognition particle-docking protein FtsY (324 aa)

ECH_1111	signal recognition particle-docking protein FtsY (310 aa)

ERGA_CDS_08930	Cell division protein ftsY homolog (309 aa)

Erum8520	putative cell division protein FtsY (309 aa)

NSE_0545	signal recognition particle-docking protein FtsY (309 aa)

RC1205	cell division protein ftsY (303 aa)

RP775	CELL DIVISION PROTEIN FTSY (ftsY) (303 aa)

RT0762	cell division protein FtsY (303 aa)

TW556	cell division protein FtsY (222 aa)

ftsY	Cellular processes (326 aa)

WD0888	cell division ABC transporter, periplasmic substrate-binding protein FtsY (303 aa)

Wbm0755	signal recognition particle-docking protein FtsY (303 aa)

Cluster 121

Number of proteins: 20

Average blastp percent identity: 46%

Average blastp sequence coverage: 95%

AM055	phenylalanyl tRNA synthetase alpha subunit (351 aa)

APH_0059	phenylalanyl-tRNA synthetase, alpha subunit (342 aa)

BH00840	Phenylalanyl-tRNA synthetase alpha chain (361 aa)

Bfl355	phenylalanyl-tRNA synthetase alpha chain (335 aa)

BR2122	phenylalanyl-tRNA synthetase, alpha subunit (369 aa)

BU129	phenylalanyl-tRNA synthetase alpha chain (329 aa)

CP0862	phenylalanyl-tRNA synthetase alpha chain (339 aa)

CBU1322	phenylalanyl-tRNA synthetase, alpha subunit (328 aa)

ECH_0196	phenylalanyl-tRNA synthetase, alpha subunit (346 aa)

ERGA_CDS_01280	Phenylalanyl-tRNA synthetase alpha chain (344 aa)

Erum1360	phenylalanyl-tRNA synthetase alpha chain (344 aa)

NSE_0216	phenylalanyl-tRNA synthetase, alpha subunit (325 aa)

RC0581	phenylalanyl-tRNA synthetase alpha chain [EC:6.1.1.20] (349 aa)

RP417	PHENYLALANYL-TRNA SYNTHETASE ALPHA CHAIN (pheS) (350 aa)

RT0403	phenylalanine--tRNA ligase alpha subunit (350 aa)

TW167	phenylalanyl-tRNA synthetase alpha chain (336 aa)

pheS	Protein synthesis (333 aa)

WD0132	phenylalanyl-tRNA synthetase, alpha subunit (347 aa)

Wbm0087	phenylalanyl-tRNA synthetase, alpha subunit (347 aa)

Cluster 122

Number of proteins: 20

Average blastp percent identity: 46%

Average blastp sequence coverage: 85%

AM826	50S ribosomal protein L28 (134 aa)

APH_0359	ribosomal protein L28 (103 aa)

BH15920	50S ribosomal protein l28 (97 aa)

Bfl612	50S ribosomal subunit protein L28 (79 aa)

BR2015	ribosomal protein L28 (97 aa)

BU086	50S ribosomal protein L28 (75 aa)

CP0430	ribosomal protein L28 (89 aa)

CBU0291	ribosomal protein L28 (79 aa)

ECH_0484	50S ribosomal protein L28 (100 aa)

ERGA_CDS_05500	50S ribosomal protein L28 (100 aa)

Erum5350	50S ribosomal protein L28 (100 aa)

NSE_0321	ribosomal protein L28 (102 aa)

RC0136	50S ribosomal protein L28 (97 aa)

RP099	50S RIBOSOMAL PROTEIN L28 (rpmB) (97 aa)

RT0038	50S ribosomal protein L28 (97 aa)

TW113	50S ribosomal protein L28 (78 aa)

rpmB	Protein synthesis (78 aa)

WD0391	ribosomal protein L28 (104 aa)

Wbm0302	ribosomal protein L28 (104 aa)

Cluster 123

Number of proteins: 20

Average blastp percent identity: 46%

Average blastp sequence coverage: 94%

AM839	signal recognition particle protein (451 aa)

APH_0349	signal recognition particle protein (449 aa)

BH16040	Signal recognition particle protein (523 aa)

Bfl172	4.5S-RNP protein, GTP binding export factor, part of signal recognition particle with 4.5 RNA (457 aa)

BR1826	signal recognition particle protein (523 aa)

BU393	signal recognition particle protein (451 aa)

CP0658	signal recognition particle protein FFH (448 aa)

CBU0450	signal recognition particle protein (461 aa)

ECH_0475	signal recognition particle protein (448 aa)

ERGA_CDS_05580	Signal recognition particle protein (450 aa)

Erum5430	signal recognition particle protein (450 aa)

NSE_0540	signal recognition particle protein (449 aa)

RC0214	signal recognition particle protein (449 aa)

RP173	SIGNAL RECOGNITION PARTICLE PROTEIN (ffh) (449 aa)

RT0164	signal recognition particle protein (449 aa)

TW462	signal recognition particle protein (448 aa)

ffh	Protein fate (446 aa)

WD1080	signal recognition particle protein (447 aa)

Wbm0049	signal recognition particle protein (375 aa)

Cluster 124

Number of proteins: 20

Average blastp percent identity: 46%

Average blastp sequence coverage: 97%

AM261	ribosomal protein L7/L12 (134 aa)

APH_1025	ribosomal protein L7/L12 (134 aa)

BH06090	50S ribosomal protein l7 /l12 (123 aa)

Bfl558	50S ribosomal subunit protein L7/L12 (126 aa)

BR1245	ribosomal protein L7/L12 (124 aa)

BU035	50S ribosomal protein L7/L12 (122 aa)

CP0695	ribosomal protein L7/L12 (129 aa)

CBU0229	ribosomal protein L7/L12 (126 aa)

ECH_0953	ribosomal protein L7/L12 (133 aa)

ERGA_CDS_01640	50S ribosomal protein L7/L12 (136 aa)

Erum1710	50S ribosomal protein L7/L12 (131 aa)

NSE_0679	ribosomal protein L7/L12 (137 aa)

RC0180	50S ribosomal protein L7/L12 (140 aa)

RP139	50S RIBOSOMAL PROTEIN L7/L12 (rplL) (125 aa)

RT0128	50S ribosomal protein L7/L12 (125 aa)

TW711	50S ribosomal protein L7/L12 (131 aa)

rplL	Protein synthesis (123 aa)

WD0023	ribosomal protein L7/L12 (133 aa)

Wbm0648	ribosomal protein L7/L12 (133 aa)

Cluster 125

Number of proteins: 20

Average blastp percent identity: 45%

Average blastp sequence coverage: 92%

AM865	replicative DNA helicase (491 aa)

APH_0323	replicative DNA helicase (485 aa)

BH05270	Replicative DNA helicase (495 aa)

Bfl027	replicative DNA helicase (466 aa)

BR0450	replicative DNA helicase (501 aa)

BU546	replicative DNA helicase (459 aa)

CP0131	replicative DNA helicase (468 aa)

CBU0868	replicative DNA helicase, intein-containing (610 aa)

ECH_0451	replicative DNA helicase (488 aa)

ERGA_CDS_05910	Replicative DNA helicase (486 aa)

Erum5710	replicative DNA helicase (486 aa)

NSE_0551.1	replicative DNA helicase (494 aa)

RC0803	replicative DNA helicase [EC:3.6.1.-] (494 aa)

RP542	PROBABLE REPLICATIVE DNA HELICASE (dnaB) (486 aa)

RT0531	replicative DNA helicase (497 aa)

TW738	replicative DNA helicase (441 aa)

dnaB	DNA metabolism (465 aa)

WD0354	replicative DNA helicase (483 aa)

Wbm0299	replicative DNA helicase (455 aa)

Cluster 126

Number of proteins: 20

Average blastp percent identity: 45%

Average blastp sequence coverage: 90%

AM917	30S ribosomal protein S1 (559 aa)

APH_0273	ribosomal protein S1 (542 aa)

BH00930	30S ribosomal protein S1 (566 aa)

Bfl380	30S ribosomal protein S1 (575 aa)

BR0027	ribosomal protein S1 (566 aa)

BU309	30S ribosomal protein S1 (558 aa)

CP0442	ribosomal protein S1 (580 aa)

CBU0528	ribosomal protein S1 (551 aa)

ECH_0402	ribosomal protein S1, putative (567 aa)

ERGA_CDS_06340	30S ribosomal protein S1 (567 aa)

Erum6120	30S ribosomal protein S1 (565 aa)

NSE_0408	ribosomal protein S1 (553 aa)

RC0747	30S ribosomal protein S1 (568 aa)

RP521	30S RIBOSOMAL PROTEIN S1 (rpsA) (568 aa)

RT0508	30S ribosomal protein S1 (568 aa)

TW293	30S ribosomal protein S1 (473 aa)

rpsA	Protein synthesis (554 aa)

WD1090	ribosomal protein S1, putative (550 aa)

Wbm0059	ribosomal protein S1 (491 aa)

Cluster 127

Number of proteins: 20

Average blastp percent identity: 45%

Average blastp sequence coverage: 93%

AM893	50S ribosomal protein L15 (146 aa)

APH_0298	ribosomal protein L15 (145 aa)

BH10320	50S ribosomal protein l15 (154 aa)

Bfl210	50S ribosomal subunit protein L15 (147 aa)

BR1214	ribosomal protein L15 (156 aa)

BU505	50S ribosomal protein L15 (144 aa)

CP0117	ribosomal protein L15 (144 aa)

CBU0257	ribosomal protein L15 (143 aa)

ECH_0427	ribosomal protein L15 (156 aa)

ERGA_CDS_06110	50S ribosomal protein L15 (157 aa)

Erum5900	50S ribosomal protein L15 (157 aa)

NSE_0284	ribosomal protein L15 (149 aa)

RC0987	50S ribosomal protein L15 (153 aa)

RP640	50S RIBOSOMAL PROTEIN L15 (rplO) (150 aa)

RT0632	50S ribosomal protein L15 (150 aa)

TW536	50S ribosomal protein L15 (160 aa)

rplO	Protein synthesis (144 aa)

WD0663	ribosomal protein L15 (156 aa)

Wbm0323	ribosomal protein L15 (156 aa)

Cluster 128

Number of proteins: 20

Average blastp percent identity: 45%

Average blastp sequence coverage: 96%

AM335	tRNA (5-methylaminomethyl-2-thiouridylate)-methyltransferase (387 aa)

APH_0910	tRNA (5-methylaminomethyl-2-thiouridylate)-methyltransferase (378 aa)

BH12020	tRNA (5-methylaminomethyl-2-thiouridylate)-methyltransferase (409 aa)

Bfl392	tRNA (5-methylaminomethyl-2-thiouridylate)-methyltransferase (369 aa)

BR1591	tRNA (5-methylaminomethyl-2-thiouridylate)-methyltransferase (398 aa)

BU261	tRNA (5-methylaminomethyl-2-thiouridylate)-methy ltransferase (392 aa)

CP0315	tRNA (5-methylaminomethyl-2-thiouridylate)-methyltransferase (361 aa)

CBU1147	tRNA (5-methylaminomethyl-2-thiouridylate)-methyltransferase (362 aa)

ECH_0872	tRNA (5-methylaminomethyl-2-thiouridylate)-methyltransferase (367 aa)

ERGA_CDS_02210	Probable tRNA (5-methylaminomethyl-2-thiouridylate)-methyltransferase (370 aa)

Erum2230	tRNA (5-methylaminomethyl-2-thiouridylate)-methyltransferase (370 aa)

NSE_0468	tRNA (5-methylaminomethyl-2-thiouridylate)-methyltransferase (371 aa)

RC0410	tRNA (5-methylaminomethyl-2-thiouridylate)-methyltransferase [EC:2.1.1.61] (370 aa)

RP306	unknown (358 aa)

RT0296	tRNA (5-methylaminomethyl-2-thiouridylate)-methyltransferase (358 aa)

TW415	tRNA (5-methylaminomethyl-2-thiouridylate)-methyltransferase (359 aa)

ycfB	Protein synthesis (368 aa)

WD1259	tRNA (5-methylaminomethyl-2-thiouridylate)-methyltransferase (370 aa)

Wbm0155	tRNA (5-methylaminomethyl-2-thiouridylate)-methyltransferase (371 aa)

Cluster 129

Number of proteins: 20

Average blastp percent identity: 45%

Average blastp sequence coverage: 95%

AM012	tryptophanyl-tRNA synthetase (329 aa)

APH_0035	tryptophanyl-tRNA synthetase (331 aa)

BH13470	Tryptophanyl-tRNA synthetase (356 aa)

Bfl569	tryptophanyl-tRNA synthetase (334 aa)

BR0142	tryptophanyl-tRNA synthetase (385 aa)

BU536	tryptophanyl-tRNA synthetase (335 aa)

CP1069	tryptophanyl-tRNA synthetase (344 aa)

CBU2049	tryptophanyl-tRNA synthetase (356 aa)

ECH_0167	tryptophanyl-tRNA synthetase (333 aa)

ERGA_CDS_01050	Tryptophanyl-tRNA synthetase (340 aa)

Erum1120	tryptophanyl-tRNA synthetase (332 aa)

NSE_0213	tryptophanyl-tRNA synthetase (330 aa)

RC0710	tryptophanyl-tRNA synthetase [EC:6.1.1.2] (330 aa)

RP468	TRYPTOPHANYL-TRNA SYNTHETASE (trpS) (330 aa)

RT0455	tryptophan--tRNA ligase (330 aa)

TW686	tryptophanyl-tRNA synthetase (335 aa)

trpS	Protein synthesis (334 aa)

WD0801	tryptophanyl-tRNA synthetase (332 aa)

Wbm0534	tryptophanyl-tRNA synthetase (332 aa)

Cluster 130

Number of proteins: 20

Average blastp percent identity: 45%

Average blastp sequence coverage: 92%

AM605	S-adenosyl-methyltransferase (291 aa)

APH_0690	S-adenosyl-methyltransferase MraW (295 aa)

BH11320	hypothetical protein (346 aa)

Bfl134	putative S-adenosyl methionine adenyltransferase (321 aa)

BR1439	conserved hypothetical protein TIGR00006 (346 aa)

BU224	hypothetical protein (312 aa)

CP0333	conserved hypothetical protein (297 aa)

CBU0116	S-adenosyl-methyltransferase MraW (307 aa)

ECH_0542	S-adenosyl-methyltransferase MraW (297 aa)

ERGA_CDS_04980	S-adenosyl-methyltransferase mraW (301 aa)

Erum4860	putative S-adenosyl-methyltransferase MraW (301 aa)

NSE_0921	S-adenosyl-methyltransferase MraW (296 aa)

RC0857	unknown (307 aa)

RP569	unknown (306 aa)

RT0557	S-adenosyl-methyltransferase MraW (306 aa)

TW220	unknown (317 aa)

yabC	Hypothetical proteins (309 aa)

WD0170	S-adenosyl-methyltransferase MraW (314 aa)

Wbm0107	S-adenosyl-methyltransferase MraW (323 aa)

Cluster 131

Number of proteins: 20

Average blastp percent identity: 44%

Average blastp sequence coverage: 90%

AM409	protein kinase C inhibitor 1 (123 aa)

APH_0860	HIT family protein (124 aa)

BH06190	Hit-like protein involved in cell-cycle regulation (140 aa)

Bfl398	putative protein kinase C inhibitor (118 aa)

BR1168	HIT family protein (140 aa)

BU357	hypothetical protein (114 aa)

CP0266	HIT family protein (125 aa)

CBU0796	HIT family protein (113 aa)

ECH_0826	HIT family protein (116 aa)

ERGA_CDS_02680	Protein kinase C inhibitor 1 (113 aa)

Erum2680	putative HIT-like protein (113 aa)

NSE_0413	HIT domain protein (117 aa)

RC0431	protein kinase C inhibitor 1 (120 aa)

RP317	PROTEIN KINASE C INHIBITOR 1 (pkcI) (120 aa)

RT0307	protein kinase C inhibitor 1 (120 aa)

TW282	HIT-like protein (107 aa)

ycfF	Unknown function (113 aa)

WD1125	HIT family protein (122 aa)

Wbm0240	HIT family protein (122 aa)

Cluster 132

Number of proteins: 20

Average blastp percent identity: 44%

Average blastp sequence coverage: 93%

AM1233	30S ribosomal protein S20 (106 aa)

APH_1374	ribosomal protein S20 (96 aa)

BH01170	30S ribosomal protein s20 (88 aa)

Bfl116	30S ribosomal protein S20 (88 aa)

BR2185	ribosomal protein S20 (88 aa)

BU151	30S ribosomal protein S20 (89 aa)

CP1118	ribosomal protein S20 (99 aa)

CBU0389	ribosomal protein S20 (90 aa)

ECH_0071	ribosomal protein S20 (95 aa)

ERGA_CDS_00370	30S ribosomal protein S20 (95 aa)

Erum0480	30S ribosomal protein S20 (95 aa)

NSE_0792	ribosomal protein S20 (87 aa)

RC0980	30S ribosomal protein S20 (93 aa)

RP633	30S RIBOSOMAL PROTEIN S20 (rpsT) (90 aa)

RT0625	30S ribosomal protein S20 (90 aa)

TW276	30S ribosomal protein S20 (95 aa)

rpsT	Protein synthesis (88 aa)

WD1150	ribosomal protein S20 (89 aa)

Wbm0734	ribosomal protein S20 (89 aa)

Cluster 133

Number of proteins: 20

Average blastp percent identity: 44%

Average blastp sequence coverage: 96%

AM816	Fe superoxide dismutase (223 aa)

APH_0371	Fe superoxide dismutase (206 aa)

BH04630	Superoxide dismutase (200 aa)

Bfl022	manganese superoxide dismutase (207 aa)

BR0566	superoxide dismutase, Fe-Mn family (199 aa)

BU189	superoxide dismutase (203 aa)

CP0718	superoxide dismutase (207 aa)

CBU1708	superoxide dismutase (fe) (193 aa)

ECH_0493	superoxide dismutase, Fe (205 aa)

ERGA_CDS_05420	Superoxide dismutase (210 aa)

Erum5270	superoxide dismutase [Fe] (210 aa)

NSE_0843	superoxide dismutase, Fe (205 aa)

RC0778	superoxide dismutase [EC:1.15.1.1] (209 aa)

RP535	SUPEROXIDE DISMUTASE (sodB) (209 aa)

RT0523	superoxide dismutase (209 aa)

TW299	superoxide dismutase (202 aa)

sodA	Cellular processes (208 aa)

WD0738	superoxide dismutase, Fe (206 aa)

Wbm0220	superoxide dismutase, Fe (202 aa)

Cluster 134

Number of proteins: 20

Average blastp percent identity: 44%

Average blastp sequence coverage: 92%

AM481	cysteinyl-tRNA synthetase (466 aa)

APH_0555	cysteinyl-tRNA synthetase (462 aa)

BH05180	Cysteinyl-tRNA synthetase (503 aa)

Bfl304	cysteinyl-tRNA synthetase (474 aa)

BR0677	cysteinyl-tRNA synthetase (506 aa)

BU487	cysteinyl-tRNA synthetase (464 aa)

CP0931	cysteinyl-tRNA synthetase (474 aa)

CBU1487	cysteinyl-tRNA synthetase (458 aa)

ECH_0768	cysteinyl-tRNA synthetase (459 aa)

ERGA_CDS_03270	Cysteinyl-tRNA synthetase (457 aa)

Erum3250	cysteinyl-tRNA synthetase (457 aa)

NSE_0333	cysteinyl-tRNA synthetase (507 aa)

RC0111	cysteinyl-tRNA synthetase [EC:6.1.1.16] (459 aa)

RP085	CYSTEINYL-TRNA SYNTHETASE (cysS) (457 aa)

RT0051	cysteine--tRNA ligase (457 aa)

TW347	cysteinyl-tRNA synthetase (466 aa)

cysS	Protein synthesis (463 aa)

WD0149	cysteinyl-tRNA synthetase (457 aa)

Wbm0680	cysteinyl-tRNA synthetase (433 aa)

Cluster 135

Number of proteins: 20

Average blastp percent identity: 44%

Average blastp sequence coverage: 96%

AM661	pseudouridylate synthase (247 aa)

APH_0502	tRNA pseudouridine synthase A (246 aa)

BH00740	tRNA pseudouridine synthase A (247 aa)

Bfl496	tRNA pseudouridine synthase A (252 aa)

BRA1033	tRNA pseudouridine synthase A (251 aa)

BU199	pseudouridylate synthase I (266 aa)

CP0168	tRNA pseudouridine synthase A (267 aa)

CBU0892	tRNA pseudouridine synthase A (261 aa)

ECH_0622	tRNA pseudouridine synthase A (245 aa)

ERGA_CDS_04360	tRNA pseudouridine synthase A (246 aa)

Erum4240	tRNA pseudouridine synthase A (246 aa)

NSE_0020	tRNA pseudouridine synthase A (243 aa)

RC1328	pseudouridylate synthase I [EC:4.2.1.70] (245 aa)

RP857	PSEUDOURIDYLATE SYNTHASE I (truA) (253 aa)

RT0846	tRNA pseudouridylate synthase A (245 aa)

TW526	pseudouridylate synthase I (315 aa)

truA	Protein synthesis (264 aa)

WD1068	tRNA pseudouridine synthase A (245 aa)

Wbm0428	tRNA pseudouridine synthase A (249 aa)

Cluster 136

Number of proteins: 20

Average blastp percent identity: 44%

Average blastp sequence coverage: 93%

AM895	50S ribosomal protein L18 (125 aa)

APH_0296	50S ribosomal protein L18 (121 aa)

BH10350	50S ribosomal protein l18 (120 aa)

Bfl207	50S ribosomal subunit protein L18 (117 aa)

BR1217	ribosomal protein L18 (120 aa)

BU508	50S ribosomal protein L18 (122 aa)

CP0115	ribosomal protein L18 (123 aa)

CBU0254	ribosomal protein L18 (117 aa)

ECH_0425	ribosomal protein L18 (121 aa)

ERGA_CDS_06130	50S ribosomal protein L18 (120 aa)

Erum5920	50S ribosomal protein L18 (120 aa)

NSE_0282	ribosomal protein L18 (117 aa)

RC0990	50S ribosomal protein L18 (118 aa)

RP643	50S RIBOSOMAL PROTEIN L18 (rplR) (118 aa)

RT0635	50S ribosomal protein L18 (118 aa)

TW539	50S ribosomal protein L18 (119 aa)

rplR	Protein synthesis (117 aa)

WD0665	ribosomal protein L18 (123 aa)

Wbm0325	Ribosomal L18p/L5e family (123 aa)

Cluster 137

Number of proteins: 20

Average blastp percent identity: 44%

Average blastp sequence coverage: 94%

AM1205	tyrosyl-tRNA synthetase (414 aa)

APH_1244	tyrosyl-tRNA synthetase (412 aa)

BH08670	Tyrosyl-tRNA synthetase (417 aa)

Bfl371	tyrosyl-tRNA synthetase (422 aa)

BR0926	tyrosyl-tRNA synthetase (417 aa)

BU121	tyrosyl-tRNA synthetase (422 aa)

CP0397	tyrosyl-tRNA synthetase (412 aa)

CBU0181	tyrosyl-tRNA synthetase (399 aa)

ECH_0091	tyrosyl-tRNA synthetase (418 aa)

ERGA_CDS_00520	Tyrosyl-tRNA synthetase (418 aa)

Erum0620	tyrosyl-tRNA synthetase (418 aa)

NSE_0345	tyrosyl-tRNA synthetase (391 aa)

RC0823	tyrosyl-tRNA synthetase [EC:6.1.1.1] (411 aa)

RP556	TYROSYL-TRNA SYNTHETASE (tyrS) (411 aa)

RT0544	tyrosine--tRNA ligase (411 aa)

TW169	tyrosyl-tRNA synthetase (471 aa)

tyrS	Protein synthesis (496 aa)

WD1115	tyrosyl-tRNA synthetase (416 aa)

Wbm0248	tyrosyl-tRNA synthetase (386 aa)

Cluster 138

Number of proteins: 20

Average blastp percent identity: 43%

Average blastp sequence coverage: 92%

AM1231	prolipoprotein diacylglyceryl transferase (285 aa)

APH_1376	prolipoprotein diacylglyceryl transferase (277 aa)

BH11800	Prolipoprotein diacylglyceryl transferase (292 aa)

Bfl265	prolipoprotein diacylglyceryl transferase (284 aa)

BR1528	prolipoprotein diacylglyceryl transferase (281 aa)

BU439	prolipoprotein diacylglyceryl transferase (281 aa)

CP0447	prolipoprotein diacylglyceryl transferase, putative (292 aa)

CBU1549	prolipoprotein diacylglyceryl transferase (261 aa)

ECH_1101	prolipoprotein diacylglyceryl transferase (280 aa)

ERGA_CDS_08860	Prolipoprotein diacylglyceryl transferase (259 aa)

Erum8440	prolipoprotein diacylglyceryl transferase (259 aa)

NSE_0854	prolipoprotein diacylglyceryl transferase (266 aa)

RC0072	prolipoprotein diacylglyceryl transferase [EC:2.4.99.-] (259 aa)

RP046	PROLIPOPROTEIN DIACYLGLYCERYL TRANSFERASE (lgt) (268 aa)

RT0084	prolipoprotein diacylglyceryl transferase (259 aa)

TW309	putative prolipoprotein diacylglyceryl transferase (272 aa)

lgt	Protein fate (279 aa)

WD0768	prolipoprotein diacylglyceryl transferase (263 aa)

Wbm0079	prolipoprotein diacylglyceryl transferase (263 aa)

Cluster 139

Number of proteins: 20

Average blastp percent identity: 43%

Average blastp sequence coverage: 92%

AM597	50S ribosomal protein L21 (102 aa)

APH_0697	ribosomal protein L21 (99 aa)

BH01390	50S ribosomal protein l21 (158 aa)

Bfl093	50S ribosomal subunit protein L21 (127 aa)

BR1850	ribosomal protein L21 (142 aa)

BU387	50S ribosomal protein L21 (108 aa)

CP0206	ribosomal protein L21 (106 aa)

CBU0385	ribosomal protein L21 (116 aa)

ECH_0545	ribosomal protein L21 (102 aa)

ERGA_CDS_04950	50S ribosomal protein L21 (102 aa)

Erum4830	50S Ribosomal protein L21 (102 aa)

NSE_0898	ribosomal protein L21 (99 aa)

RC1162	50S ribosomal protein L21 (105 aa)

RP751	50S RIBOSOMAL PROTEIN L21 (rplU) (105 aa)

RT0736	50S ribosomal protein L21 (105 aa)

TW473	50S ribosomal protein L21 (115 aa)

rplU	Protein synthesis (103 aa)

WD0134	ribosomal protein L21 (100 aa)

Wbm0701	ribosomal protein L21 (100 aa)

Cluster 140

Number of proteins: 20

Average blastp percent identity: 42%

Average blastp sequence coverage: 92%

AM736	transcription termination factor A (515 aa)

APH_0445	N utilization substance protein A (514 aa)

BH02170	N utilization substance protein A (528 aa)

Bfl103	transcription pausing L factor (501 aa)

BR2163	N utilization substance protein A (537 aa)

BU378	N utilization substance protein A (496 aa)

CP0441	N utilization substance protein A (434 aa)

CBU1433	N utilization substance protein A (503 aa)

ECH_0562	N utilization substance protein A (517 aa)

ERGA_CDS_04820	Transcription elongation protein nusA (517 aa)

Erum4700	putative N utilization substance protein A (517 aa)

NSE_0521	N utilization substance protein A (537 aa)

RC0817	N utilization substance protein A (503 aa)

RP553	N UTILIZATION SUBSTANCE PROTEIN A (nusA) (503 aa)

RT0540	transcription elongation protein NusA (N utilization substance protein A) (503 aa)

TW156	N utilization substance protein A (319 aa)

nusA	Transcription (494 aa)

WD1319	N utilization substance protein A (520 aa)

Wbm0538	N utilization substance protein A (520 aa)

Cluster 141

Number of proteins: 20

Average blastp percent identity: 42%

Average blastp sequence coverage: 78%

AM844	ribonuclease E (619 aa)

APH_0345	ribonuclease, Rne/Rng family (636 aa)

BH08720	Ribonuclease E (880 aa)

Bfl410	ribonuclease E (710 aa)

BR0912	ribonuclease, Rne/Rng domain protein (922 aa)

BU347	ribonuclease E (902 aa)

CP0901	ribonuclease G (515 aa)

CBU0486	ribonuclease, Rne/Rng family (720 aa)

ECH_0470	ribonuclease, Rne/Rng family (606 aa)

ERGA_CDS_05680	Ribonuclease E (610 aa)

Erum5510	putative ribonuclease (610 aa)

NSE_0120	ribonuclease, Rne/Rng family (549 aa)

RC0342	ribonuclease E [EC:3.1.4.-] (690 aa)

RP256	RIBONUCLEASE E (rne) (683 aa)

RT0248	ribonuclease E (683 aa)

TW474	ribonuclease G (549 aa)

rne	Transcription (783 aa)

WD0929	ribonuclease, Rne/Rng family protein (587 aa)

Wbm0496	ribonuclease, Rne/Rng family protein (560 aa)

Cluster 142

Number of proteins: 20

Average blastp percent identity: 42%

Average blastp sequence coverage: 96%

AM896	50S ribosomal protein L6 (181 aa)

APH_0295	ribosomal protein L6 (180 aa)

BH10360	50S ribosomal protein l6 (177 aa)

Bfl206	50S ribosomal subunit protein L6 (181 aa)

BR1218	ribosomal protein L6 (177 aa)

BU509	50S ribosomal protein L6 (190 aa)

CP0114	ribosomal protein L6 (183 aa)

CBU0253	ribosomal protein L6 (178 aa)

ECH_0424	ribosomal protein L6 (144 aa)

ERGA_CDS_06140	50S ribosomal protein L6 (178 aa)

Erum5930	50S ribosomal protein L6 (178 aa)

NSE_0281	ribosomal protein L6 (176 aa)

RC0991	50S ribosomal protein L6 (177 aa)

RP644	50S RIBOSOMAL PROTEIN L6 (rplF) (177 aa)

RT0636	50S ribosomal protein L6 (177 aa)

TW540	50S ribosomal protein L6 (198 aa)

rplF	Protein synthesis (178 aa)

WD0666	ribosomal protein L6 (181 aa)

Wbm0326	ribosomal protein L6 (181 aa)

Cluster 143

Number of proteins: 20

Average blastp percent identity: 41%

Average blastp sequence coverage: 95%

AM911	50S ribosomal protein L4 (208 aa)

APH_0281	ribosomal protein L4 (208 aa)

BH10500	50S ribosomal protein l4 (206 aa)

Bfl192	50S ribosomal subunit protein L4 (212 aa)

BR1232	ribosomal protein L4 (206 aa)

BU523	50S ribosomal protein L4 (201 aa)

CP0101	ribosomal protein L4 (224 aa)

CBU0239	ribosomal protein L4 (205 aa)

ECH_0410	ribosomal protein L4 (205 aa)

ERGA_CDS_06280	50S ribosomal protein L4 (205 aa)

Erum6060	50S ribosomal protein L4 (205 aa)

NSE_0267	ribosomal protein L4 (214 aa)

RC1005	50S ribosomal protein L4 (207 aa)

RP658	50S RIBOSOMAL PROTEIN L4 (rplD) (207 aa)

RT0650	50S ribosomal protein L4 (207 aa)

TW553	50S ribosomal protein L4 (248 aa)

rplD	Protein synthesis (201 aa)

WD0680	ribosomal protein L4 (204 aa)

Wbm0340	ribosomal protein L4/L1 family (204 aa)

Cluster 144

Number of proteins: 20

Average blastp percent identity: 41%

Average blastp sequence coverage: 91%

AM910	50S ribosomal protein L23 (102 aa)

APH_0282	ribosomal protein L23 (96 aa)

BH10490	50S ribosomal protein l23 (97 aa)

Bfl193	50S ribosomal subunit protein L23 (99 aa)

BR1231	ribosomal protein L23 (97 aa)

BU522	50S ribosomal protein L23 (100 aa)

CP0102	ribosomal protein L23 (111 aa)

CBU0240	ribosomal protein L23 (95 aa)

ECH_0411	ribosomal protein L23 (96 aa)

ERGA_CDS_06270	50S ribosomal protein L23 (96 aa)

Erum6050	50S ribosomal protein L23 (96 aa)

NSE_0268	ribosomal protein L23 (94 aa)

RC1004	50S ribosomal protein L23 (98 aa)

RP657	50S RIBOSOMAL PROTEIN L23 (rplW) (98 aa)

RT0649	50S ribosomal protein L23 (98 aa)

TW552	50S ribosomal protein L23 (103 aa)

rplW	Protein synthesis (101 aa)

WD0679	ribosomal protein L23 (95 aa)

Wbm0339	ribosomal protein L23 (95 aa)

Cluster 145

Number of proteins: 20

Average blastp percent identity: 41%

Average blastp sequence coverage: 97%

AM130	ribosome recycling factor (183 aa)

APH_0112	ribosome recycling factor (184 aa)

BH06250	Ribosome recycling factor (186 aa)

Bfl274	ribosome recycling factor (188 aa)

BR1159	ribosome recycling factor (186 aa)

BU234	ribosome recycling factor (185 aa)

CP0047	ribosome recycling factor (180 aa)

CBU1383	ribosome recycling factor (185 aa)

ECH_0267	ribosome recycling factor (185 aa)

ERGA_CDS_07530	Ribosome recycling factor (185 aa)

Erum7230	ribosome recycling factor (185 aa)

NSE_0945	ribosome recycling factor (185 aa)

RC0197	ribosome recycling factor (186 aa)

RP154	RIBOSOME RECYCLING FACTOR (rrf) (186 aa)

RT0143	ribosome recycling factor (186 aa)

TW446	ribosome recycling factor (181 aa)

frr	Protein synthesis (185 aa)

WD0529	ribosome recycling factor (185 aa)

Wbm0805	ribosome recycling factor (185 aa)

Cluster 146

Number of proteins: 20

Average blastp percent identity: 41%

Average blastp sequence coverage: 94%

AM1069	ribonuclease III (232 aa)

APH_1149	ribonuclease III (236 aa)

BH05090	Ribonuclease III (235 aa)

Bfl540	ribonuclease III (232 aa)

BR0661	ribonuclease III (245 aa)

BU258	ribonuclease III (226 aa)

CP0721	ribonuclease III (237 aa)

CBU1503	ribonuclease III (233 aa)

ECH_1054	ribonuclease III (226 aa)

ERGA_CDS_08450	Ribonuclease III (227 aa)

Erum8070	ribonuclease III (227 aa)

NSE_0918	ribonuclease III (222 aa)

RC0157	ribonuclease III [EC:3.1.26.3] (227 aa)

RP117	RIBONUCLEASE III (rnc) (225 aa)

RT0019	ribonuclease III (227 aa)

TW562	ribonuclease III (223 aa)

rnc	Transcription (226 aa)

WD1240	ribonuclease III, putative (232 aa)

Wbm0099	ribonuclease III (243 aa)

Cluster 147

Number of proteins: 20

Average blastp percent identity: 41%

Average blastp sequence coverage: 79%

AM1214	DNA polymerase (864 aa)

APH_0001	DNA polymerase I (853 aa)

BH00060	DNA polymerase I (968 aa)

Bfl619	DNA polymerase I (943 aa)

BR0123	DNA polymerase I (979 aa)

BU431	DNA polymerase I (286 aa)

CP0135	DNA polymerase I (870 aa)

CBU1795	DNA polymerase I (895 aa)

ECH_0080	DNA polymerase I (944 aa)

ERGA_CDS_00380	DNA polymerase I (POL I) (860 aa)

Erum0490	DNA polymerase I (865 aa)

NSE_0675	DNA polymerase I (829 aa)

RC1206	DNA polymerase I (POL I) [EC:2.7.7.7] (875 aa)

RP776	DNA POLYMERASE I (polA) (867 aa)

RT0763	DNA-directed DNA polymerase I (872 aa)

TW292	DNA polymerase I (356 aa)

polA	DNA metabolism (289 aa)

WD1003	DNA polymerase I (858 aa)

Wbm0725	DNA polymerase I (849 aa)

Cluster 148

Number of proteins: 20

Average blastp percent identity: 41%

Average blastp sequence coverage: 94%

AM256	transcription antitermination protein (188 aa)

APH_1029	transcription termination/antitermination factor NusG (186 aa)

BH06050	Transcription antitermination protein (176 aa)

Bfl562	transcription antitermination protein NusG (181 aa)

BR1249	transcription antitermination protein NusG (175 aa)

BU039	transcription antitermination protein nusG (181 aa)

CP0699	transcription antitermination protein NusG (182 aa)

CBU0225	transcription antitermination protein NusG (173 aa)

ECH_0957	transcription antitermination protein NusG (179 aa)

ERGA_CDS_01600	Transcription antitermination protein NUSG (179 aa)

Erum1670	transcription antitermination protein NusG (179 aa)

NSE_0683	transcription termination/antitermination factor NusG, putative (176 aa)

RC0176	transcription antitermination protein NusG (192 aa)

RP135	TRANSCRIPTION ANTITERMINATION PROTEIN NUSG (nusG) (192 aa)

RT0124	transcription antitermination protein NusG (N utilization substance protein G) (192 aa)

TW715	transcription antitermination protein (217 aa)

nusG	Transcription (181 aa)

WD0019	transcription antitermination protein NusG, putative (196 aa)

Wbm0652	transcription antitermination protein NusG (282 aa)

Cluster 149

Number of proteins: 20

Average blastp percent identity: 41%

Average blastp sequence coverage: 97%

AM472	elongation factor PEF-P (188 aa)

APH_0547	translation elongation factor P (188 aa)

BH15020	Elongation factor p (191 aa)

Bfl072	elongation factor P (EF-P) (187 aa)

BR1710	translation elongation factor P (186 aa)

BU020	elongation factor P (203 aa)

CP0971	translation elongation factor P (190 aa)

CBU1816	translation elongation factor P (188 aa)

ECH_0777	translation elongation factor P (189 aa)

ERGA_CDS_03190	Elongation factor P (EF-P) (189 aa)

Erum3190	putative elongation factor P (189 aa)

NSE_0196	translation elongation factor P (191 aa)

RC0322	elongation factor EF-P (188 aa)

RP238	ELONGATION FACTOR P (efp) (188 aa)

RT0230	elongation factor P (188 aa)

TW368	elongation factor EF-P (185 aa)

efp	Protein synthesis (189 aa)

WD1281	translation elongation factor P (188 aa)

Wbm0131	translation elongation factor P (188 aa)

Cluster 150

Number of proteins: 20

Average blastp percent identity: 41%

Average blastp sequence coverage: 86%

AM287	DNA polymerase III alpha subunit (1127 aa)

APH_1003	DNA polymerase III, alpha subunit (1127 aa)

BH09850	DNA polymerase III alpha subunit (1162 aa)

Bfl286	DNA polymerase III, alpha chain (1166 aa)

BR0825	DNA polymerase III, alpha subunit (1163 aa)

BU238	DNA polymerase III alpha chain (1161 aa)

CP0081	DNA polymerase III, alpha subunit (1240 aa)

CBU1337	DNA polymerase III, alpha subunit (1143 aa)

ECH_0922	DNA polymerase III, alpha subunit (1120 aa)

ERGA_CDS_01820	DNA polymerase III alpha subunit (1122 aa)

Erum1870	DNA polymerase III, alpha subunit (1119 aa)

NSE_0167	DNA polymerase III, alpha subunit (1077 aa)

RC1211	DNA polymerase III alpha chain [EC:2.7.7.7] (1181 aa)

RP778	DNA POLYMERASE III, ALPHA CHAIN (dnaE) (1182 aa)

RT0765	DNA-directed DNA polymerase III alpha subunit (1180 aa)

TW510	DNA polymerase III alpha chain (1164 aa)

dnaE	DNA metabolism (1159 aa)

WD0780	DNA polymerase III, alpha subunit (1109 aa)

Wbm0499	DNA polymerase III, alpha subunit (1081 aa)

Cluster 151

Number of proteins: 20

Average blastp percent identity: 41%

Average blastp sequence coverage: 92%

AM152	DNA ligase (673 aa)

APH_0138	DNA ligase, NAD-dependent (677 aa)

BH11140	DNA ligase (716 aa)

Bfl507	DNA ligase (594 aa)

BR1420	DNA ligase, NAD-dependent (719 aa)

BU067	DNA ligase (NAD+) (675 aa)

CP0624	DNA ligase (662 aa)

CBU0542	DNA ligase, NAD-dependent (670 aa)

ECH_0301	DNA ligase, NAD-dependent (676 aa)

ERGA_CDS_07210	DNA ligase (674 aa)

Erum6940	NAD-dependent DNA ligase (674 aa)

NSE_0500	DNA ligase, NAD-dependent (670 aa)

RC1096	DNA ligase [EC:6.5.1.2] (689 aa)

RP720	DNA LIGASE (lig) (689 aa)

RT0706	DNA ligase (NAD+) (693 aa)

TW414	DNA ligase (634 aa)

lig	DNA metabolism (591 aa)

WD0776	DNA ligase, NAD-dependent (662 aa)

Wbm0548	DNA ligase, NAD-dependent (683 aa)

Cluster 152

Number of proteins: 20

Average blastp percent identity: 41%

Average blastp sequence coverage: 87%

AM483	ribonuceoside-diphosphate reductase beta chain (324 aa)

APH_0560	ribonucleoside-diphosphate reductase, beta subunit (323 aa)

BH01950	Ribonucleotide reductase beta-chain (324 aa)

Bfl479	ribonucleoside-diphosphate reductase 1 beta chain (376 aa)

BRA0317	ribonucleoside-diphosphate reductase, beta subunit (329 aa)

BU178	ribonucleoside-diphosphate reductase 1 beta chain (376 aa)

CP0871	ribonucleoside-diphosphate reductase, beta subunit (346 aa)

CBU1554	ribonucleoside-diphosphate reductase, beta subunit (401 aa)

ECH_0766	ribonucleoside-diphosphate reductase, beta subunit (324 aa)

ERGA_CDS_03290	Ribonucleoside-diphosphate reductase beta chain (329 aa)

Erum3270	putative ribonucleoside-diphosphate reductase beta chain (324 aa)

NSE_0520	ribonucleoside-diphosphate reductase, beta subunit (322 aa)

RC0651	ribonucleoside-diphosphate reductase beta chain [EC:1.17.4.1] (328 aa)

RP512	RIBONUCLEOSIDE-DIPHOSPHATE REDUCTASE BETA CHAIN (nrdB) (324 aa)

RT0497	ribonucleoside-diphosphate reductase beta subunit (324 aa)

TW682	ribonucleotide reductase beta chain (327 aa)

nrdF	Purines, pyrimidines, nucleosides, and nucleotides (324 aa)

WD0212	ribonucleoside-diphosphate reductase, beta subunit (329 aa)

Wbm0267	ribonucleoside-diphosphate reductase, beta subunit (331 aa)

Cluster 153

Number of proteins: 20

Average blastp percent identity: 40%

Average blastp sequence coverage: 90%

AM1339	peptidyl-tRNA hydrolase (186 aa)

APH_1301	peptidyl-tRNA hydrolase (183 aa)

BH11860	Peptidyl-tRNA hydrolase (193 aa)

Bfl345	peptidyl-tRNA hydrolase (197 aa)

BR1536	peptidyl-tRNA hydrolase (250 aa)

BU190	peptidyl-tRNA hydrolase (177 aa)

CP0909	peptidyl-tRNA hydrolase (180 aa)

CBU1841	peptidyl-tRNA hydrolase (187 aa)

ECH_0141	peptidyl-tRNA hydrolase (198 aa)

ERGA_CDS_00840	Peptidyl-tRNA hydrolase (215 aa)

Erum0910	peptidyl-tRNA hydrolase (193 aa)

NSE_0307	peptidyl-tRNA hydrolase (194 aa)

RC0931	peptidyl-tRNA hydrolase [EC:3.1.1.29] (185 aa)

RP605	PEPTIDYL-TRNA HYDROLASE (pth) (185 aa)

RT0593	aminoacyl-tRNA hydrolase (185 aa)

TW651	peptidyl-tRNA hydrolase (186 aa)

pth	Protein synthesis (191 aa)

WD0175	peptidyl-tRNA hydrolase (182 aa)

Wbm0093	peptidyl-tRNA hydrolase (182 aa)

Cluster 154

Number of proteins: 20

Average blastp percent identity: 40%

Average blastp sequence coverage: 79%

AM1280	DNA polymerase III gamma subunit (474 aa)

APH_1348	DNA polymerase III, subunits gamma and tau (456 aa)

BH02320	DNA polymerase III subunit tau (646 aa)

Bfl301	DNA polymerase III subunit gamma (541 aa)

BR0034	DNA polymerase III, gamma and tau subunits (602 aa)

BU481	DNA polymerase III subunits gamma and tau (361 aa)

CP0735	DNA polymerase III, gamma subunit (442 aa)

CBU0659	DNA polymerase III, gamma and tau subunits (509 aa)

ECH_0014	DNA polymerase III, subunits gamma and tau (505 aa)

ERGA_CDS_09410	DNA polymerase III gamma and tau chains (500 aa)

Erum0040	putative DNA polymerase III, gamma subunit (487 aa)

NSE_0492	DNA polymerase III, gamma/tau subunit, putative (396 aa)

RC1336	DNA polymerase III gamma and tau chains (509 aa)

RP865	DNA POLYMERASE III SUBUNITS GAMMA AND TAU (541 aa)

RT0855	DNA-directed DNA polymerase III gamma subunit (400 aa)

TW710	DNA polymerase III gamma and tau subunit (522 aa)

dnaX	DNA metabolism (365 aa)

WD1060	DNA polymerase III, gamma and tau subunits (533 aa)

Wbm0434	dnaX (473 aa)

Cluster 155

Number of proteins: 20

Average blastp percent identity: 40%

Average blastp sequence coverage: 91%

AM558	dimethyladenosine transferase (270 aa)

APH_0622	dimethyladenosine transferase (269 aa)

BH05400	Dimethyladenosine transferase (276 aa)

Bfl126	dimethyladenosine transferase (271 aa)

BR0682	dimethyladenosine transferase (276 aa)

BU141	dimethyladenosine transferase (273 aa)

CP0791	dimethyladenosine transferase (277 aa)

CBU1982	dimethyladenosine transferase (255 aa)

ECH_0648	dimethyladenosine transferase (277 aa)

ERGA_CDS_04110	Dimethyladenosine transferase (262 aa)

Erum4030	dimethyladenosine transferase (262 aa)

NSE_0362	dimethyladenosine transferase (262 aa)

RC1022	dimethyladenosine transferase [EC:2.1.1-] (315 aa)

RP672	DIMETHYLADENOSINE TRANSFERASE (ksgA) (268 aa)

RT0666	dimethyladenosine transferase (268 aa)

TW203	dimethyladenosine transferase (448 aa)

ksgA	Protein synthesis (261 aa)

WD0090	dimethyladenosine transferase (286 aa)

Wbm0407	dimethyladenosine transferase (261 aa)

Cluster 156

Number of proteins: 20

Average blastp percent identity: 40%

Average blastp sequence coverage: 82%

AM1264	hypothetical protein (154 aa)

APH_1290	conserved hypothetical protein TIGR00043 (154 aa)

BH02240	hypothetical protein (158 aa)

Bfl316	conserved hypothetical protein (153 aa)

BR2156	conserved hypothetical protein TIGR00043 (158 aa)

BU442	hypothetical protein (111 aa)

CP0245	conserved hypothetical protein (158 aa)

CBU0567	conserved hypothetical protein TIGR00043 (152 aa)

ECH_1155	conserved hypothetical protein TIGR00043 (154 aa)

ERGA_CDS_09240	Conserved hypothetical protein (157 aa)

Erum8820	conserved hypothetical protein (157 aa)

NSE_0249	conserved hypothetical protein TIGR00043 (143 aa)

RC1142	unknown (167 aa)

RP741	unknown (180 aa)

RT0726	conserved hypothetical protein (167 aa)

TW283	unknown (171 aa)

ybeY	Hypothetical proteins (152 aa)

WD0726	conserved hypothetical protein TIGR00043 (150 aa)

Wbm0601	predicted metal-dependent hydrolase (171 aa)

Cluster 157

Number of proteins: 20

Average blastp percent identity: 40%

Average blastp sequence coverage: 95%

AM313	methionyl-tRNA formyltransferase (301 aa)

APH_0965	methionyl-tRNA formyltransferase (301 aa)

BH00750	Methionyl-tRNA formyltransferase (311 aa)

Bfl218	methionyl-tRNA formyltransferase (323 aa)

BRA1034	methionyl-tRNA formyltransferase (306 aa)

BU497	methionyl-tRNA formyltransferase (314 aa)

CP0098	methionyl-tRNA formyltransferase (321 aa)

CBU1997	methionyl-tRNA formyltransferase (314 aa)

ECH_0897	methionyl-tRNA formyltransferase (303 aa)

ERGA_CDS_02000	Methionyl-tRNA formyltransferase (303 aa)

Erum2030	methionyl-tRNA formyltransferase (303 aa)

NSE_0094	methionyl-tRNA formyltransferase (307 aa)

RC0279	methionyl-tRNA formyltransferase [EC:2.1.2.9] (303 aa)

RP209	METHIONYL-TRNA FORMYLTRANSFERASE (fmt) (303 aa)

RT0198	methionyl-tRNA formyltransferase (298 aa)

TW358	methionyl-tRNA formyltransferase (334 aa)

fmt	Protein synthesis (319 aa)

WD0866	methionyl-tRNA formyltransferase (299 aa)

Wbm0656	methionyl-tRNA formyltransferase (297 aa)

Cluster 158

Number of proteins: 20

Average blastp percent identity: 40%

Average blastp sequence coverage: 88%

AM111	thymidylate kinase (211 aa)

APH_0093	thymidylate kinase (219 aa)

BH08460	Thymidylate kinase (215 aa)

Bfl401	thymidylate kinase (211 aa)

BR0992	thymidylate kinase (211 aa)

BU353	thymidylate kinase (212 aa)

CP0486	thymidylate kinase (206 aa)

CBU0499	thymidylate kinase (198 aa)

ECH_0229	thymidylate kinase (202 aa)

ERGA_CDS_07770	Thymidylate kinase (202 aa)

Erum7460	putative thymidylate kinase (202 aa)

NSE_0547	thymidylate kinase (199 aa)

RC1047	thymidylate kinase [EC:2.7.4.9] (203 aa)

RP684	THYMIDYLATE KINASE (tmk) (203 aa)

RT0679	thymidylate kinase (203 aa)

TW695	thymidylate kinase (199 aa)

tmk	Purines, pyrimidines, nucleosides, and nucleotides (234 aa)

WD1251	thymidylate kinase (212 aa)

Wbm0148	thymidylate kinase (187 aa)

Cluster 159

Number of proteins: 20

Average blastp percent identity: 39%

Average blastp sequence coverage: 90%

AM1001	methionyl-tRNA synthetase (455 aa)

APH_1079	methionyl-tRNA synthetase (513 aa)

BH08480	Methionyl-tRNA synthetase (519 aa)

Bfl471	methionyl-tRNA synthetase (545 aa)

BR0995	methionyl-tRNA synthetase (515 aa)

BU109	methionyl-tRNA synthetase (547 aa)

CP0651	methionyl-tRNA synthetase (551 aa)

CBU1695	methionyl-tRNA synthetase (546 aa)

ECH_1000	methionyl-tRNA synthetase (506 aa)

ERGA_CDS_08050	Methionyl-tRNA synthetase (506 aa)

Erum7710	methionyl-tRNA synthetase (506 aa)

NSE_0893	methionyl-tRNA synthetase (503 aa)

RC1046	methionyl-tRNA synthetase [EC:6.1.1.10] (508 aa)

RP683	METHIONYL-TRNA SYNTHETASE (metS) (508 aa)

RT0678	methionine--tRNA ligase (507 aa)

TW763	methionyl-tRNA synthetase (583 aa)

metG	Protein synthesis (550 aa)

WD0352	methionyl-tRNA synthetase (545 aa)

Wbm0196	methionyl-tRNA synthetase (540 aa)

Cluster 160

Number of proteins: 20

Average blastp percent identity: 39%

Average blastp sequence coverage: 90%

AM801	GTP-binding protein (438 aa)

APH_0390	GTP-binding protein EngA, putative (440 aa)

BH04080	GTP-binding protein (475 aa)

Bfl530	putative GTP-binding protein (485 aa)

BR0375	GTP-binding protein (483 aa)

BU607	hypothetical GTP-binding protein (453 aa)

CP1025	GTP-binding protein (487 aa)

CBU1245	GTP-binding protein (443 aa)

ECH_0504	GTP-binding protein EngA (442 aa)

ERGA_CDS_05310	Probable GTP-binding protein engA (439 aa)

Erum5160	putative GTP binding protein EngA (439 aa)

NSE_0536	GTP-binding protein EngA, putative (473 aa)

RC1030	conserved GTP-binding protein (447 aa)

RP677	unknown (447 aa)

RT0673	probable GTP-binding protein EngA (447 aa)

TW051	cytidylate kinase/GTP-binding protein fusion (686 aa)

b2511	Unknown function (456 aa)

WD1098	GTP-binding protein (441 aa)

Wbm0586	GTP-binding protein (470 aa)

Cluster 161

Number of proteins: 20

Average blastp percent identity: 38%

Average blastp sequence coverage: 81%

AM161	30S ribosomal protein S6 (127 aa)

APH_0151	ribosomal protein S6 (109 aa)

BH05320	30S ribosomal protein s6 (135 aa)

Bfl085	30S ribosomal subunit protein S6 (120 aa)

BR0455	ribosomal protein S6 (148 aa)

BU564	30S ribosomal protein S6 (113 aa)

CP0908	ribosomal protein S6 (112 aa)

CBU0864	ribosomal protein S6 (127 aa)

ECH_0308	ribosomal protein S6 (109 aa)

ERGA_CDS_07140	30S ribosomal protein S6 (109 aa)

Erum6870	30S ribosomal protein S6 (109 aa)

NSE_0062	ribosomal protein S6 (114 aa)

RC0064	30S ribosomal protein S6 (145 aa)

RP039	30S RIBOSOMAL PROTEIN S6 (rpsF) (121 aa)

RT0091	30S ribosomal protein S6 (121 aa)

TW108	30S ribosomal protein S6 (153 aa)

rpsF	Protein synthesis (113 aa)

WD0781	ribosomal protein S6 family protein (120 aa)

Wbm0500	ribosomal protein S6 family protein (223 aa)

Cluster 162

Number of proteins: 20

Average blastp percent identity: 38%

Average blastp sequence coverage: 89%

AM138	histidyl tRNA synthetase (430 aa)

APH_0120	histidyl-tRNA synthetase (420 aa)

BH13610	Histidyl-tRNA synthetase (495 aa)

Bfl531	histidyl-tRNA synthetase (433 aa)

BRA0187	histidyl-tRNA synthetase (502 aa)

BU288	histidyl-tRNA synthetase (423 aa)

CP0084	histidyl-tRNA synthetase (430 aa)

CBU1248	histidyl-tRNA synthetase (421 aa)

ECH_0291	histidyl-tRNA synthetase (413 aa)

ERGA_CDS_07280	Histidyl-tRNA synthetase (414 aa)

Erum7010	histidyl-tRNA synthetase (414 aa)

NSE_0781	histidyl-tRNA synthetase (413 aa)

RC0412	histidyl-tRNA synthetase [EC:6.1.1.21] (439 aa)

RP308	HISTIDYL-TRNA SYNTHETASE (hisS) (416 aa)

RT0298	histidine--tRNA ligase (412 aa)

TW785	histidyl-tRNA synthetase (426 aa)

hisS	Protein synthesis (432 aa)

WD1076	histidyl-tRNA synthetase (408 aa)

Wbm0412	histidyl-tRNA synthetase (413 aa)

Cluster 163

Number of proteins: 20

Average blastp percent identity: 38%

Average blastp sequence coverage: 92%

AM791	translation elongation factor EF-Ts (291 aa)

APH_0398	translation elongation factor Ts (287 aa)

BH06230	Elongation factor ts (EF-ts) (307 aa)

Bfl272	elongation factor Ts (EF-Ts) (269 aa)

BR1161	translation elongation factor Ts (305 aa)

BU232	elongation factor Ts (268 aa)

CP0049	translation elongation factor Ts (282 aa)

CBU1385	translation elongation factor Ts (296 aa)

ECH_0515	translation elongation factor Ts (288 aa)

ERGA_CDS_05230	Elongation factor Ts (EF-TS) (288 aa)

Erum5080	elongation factor Ts (288 aa)

NSE_0947	translation elongation factor Ts (290 aa)

RC0113	elongation factor EF-Ts (309 aa)

RP087	ELONGATION FACTOR TS (tsf) (309 aa)

RT0049	elongation factor Ts (309 aa)

TW448	elongation factor EF-Ts (273 aa)

tsf	Protein synthesis (270 aa)

WD0531	translation elongation factor Ts (286 aa)

Wbm0807	translation elongation factor Ts (289 aa)

Cluster 164

Number of proteins: 20

Average blastp percent identity: 38%

Average blastp sequence coverage: 81%

AM860	ribonuclease-diphosphate reductase alpha chain (606 aa)

APH_0331	ribonucleoside-diphosphate reductase, alpha subunit (603 aa)

BH01940	Ribonucleotide reductase alpha-chain (716 aa)

Bfl478	ribonucleoside-diphosphate reductase 1 alpha chain (761 aa)

BRA0316	ribonucleoside-diphosphate reductase, alpha subunit (713 aa)

BU179	ribonucleoside-diphosphate reductase 1 alpha chain (761 aa)

CP0872	ribonucleoside-diphosphate reductase, alpha subunit (1044 aa)

CBU1553	ribonucleoside-diphosphate reductase, alpha subunit (941 aa)

ECH_0459	ribonucleoside-diphosphate reductase, alpha subunit (595 aa)

ERGA_CDS_05830	Ribonucleoside-diphosphate reductase alpha chain (595 aa)

Erum5650	putative ribonucleoside-diphosphate reductase alpha chain (595 aa)

NSE_0411	ribonucleoside-diphosphate reductase, alpha subunit (605 aa)

RC0647	ribonucleoside-diphosphate reductase alpha chain [EC:1.17.4.1] (607 aa)

RP513	RIBONUCLEOSIDE-DIPHOSPHATE REDUCTASE ALPHA CHAIN (nrdA) (607 aa)

RT0498	ribonucleoside-diphosphate reductase alpha subunit (607 aa)

TW683	ribonucleotide reductase alpha chain (792 aa)

nrdE	Purines, pyrimidines, nucleosides, and nucleotides (704 aa)

WD0197	ribonucleoside-diphosphate reductase, alpha subunit (594 aa)

Wbm0695	ribonucleoside-diphosphate reductase, alpha subunit (594 aa)

Cluster 165

Number of proteins: 20

Average blastp percent identity: 37%

Average blastp sequence coverage: 89%

AM162	ribosomal protein L9 (220 aa)

APH_0153	ribosomal protein L9 (205 aa)

BH05290	50S ribosomal protein l9 (206 aa)

Bfl087	50S ribosomal subunit protein L9 (161 aa)

BR0452	ribosomal protein L9 (189 aa)

BU562	50S ribosomal protein L9 (150 aa)

CP0906	ribosomal protein L9 (169 aa)

CBU0867	ribosomal protein L9 (152 aa)

ECH_0310	ribosomal protein L9 (208 aa)

ERGA_CDS_07120	50S ribosomal protein L9 (207 aa)

Erum6850	50S ribosomal protein L9 (207 aa)

NSE_0060	ribosomal protein L9 (160 aa)

RC0066	50S ribosomal protein L9 (171 aa)

RP041	50S RIBOSOMAL PROTEIN L9 (rplI) (171 aa)

RT0089	50S ribosomal protein L9 (171 aa)

TW105	50S ribosomal protein L9 (153 aa)

rplI	Protein synthesis (150 aa)

WD0783	ribosomal protein L9 (184 aa)

Wbm0502	ribosomal protein L9 (186 aa)

Cluster 166

Number of proteins: 20

Average blastp percent identity: 37%

Average blastp sequence coverage: 86%

AM1081	lipoprotein signal peptidase (170 aa)

APH_1160	signal peptidase II (156 aa)

BH00100	Lipoprotein signal peptidase (163 aa)

Bfl119	lipoprotein signal peptidase (153 aa)

BR0149	signal peptidase II (160 aa)

BU148	lipoprotein signal peptidase (160 aa)

CP0217	signal peptidase II (168 aa)

CBU0397	signal peptidase II (163 aa)

ECH_1060	signal peptidase II (146 aa)

ERGA_CDS_08510	Lipoprotein signal peptidase (149 aa)

Erum8120	lipoprotein signal peptidase (149 aa)

NSE_0915	signal peptidase II (184 aa)

RC0558	lipoprotein signal peptidase [EC:3.4.23.36] (206 aa)

RP408	LIPOPROTEIN SIGNAL PEPTIDASE (lspA) (194 aa)

RT0394	lipoprotein signal peptidase (196 aa)

TW512	lipoprotein signal peptidase (150 aa)

lspA	Protein fate (153 aa)

WD0760	signal peptidase II (158 aa)

Wbm0417	signal peptidase II (154 aa)

Cluster 167

Number of proteins: 20

Average blastp percent identity: 37%

Average blastp sequence coverage: 91%

AM891	adenylate kinase (216 aa)

APH_0300	adenylate kinase (216 aa)

BH10300	Adenylate kinase (192 aa)

Bfl302	adenylate kinase (226 aa)

BR1212	adenylate kinase (194 aa)

BU484	adenylate kinase (215 aa)

CP0518	adenylate kinase (213 aa)

CBU0454	adenylate kinase (231 aa)

ECH_0429	adenylate kinase (221 aa)

ERGA_CDS_06090	Adenylate kinase (220 aa)

Erum5880	adenylate kinase (220 aa)

NSE_0286	adenylate kinase (209 aa)

RC0985	adenylate kinase [EC:2.7.4.3] (212 aa)

RP638	ADENYLATE KINASE (adk) (213 aa)

RT0630	adenylate kinase (213 aa)

TW534	adenylate kinase (186 aa)

adk	Purines, pyrimidines, nucleosides, and nucleotides (210 aa)

WD0661	adenylate kinase (213 aa)

Wbm0321	adenylate kinase (216 aa)

Cluster 168

Number of proteins: 20

Average blastp percent identity: 36%

Average blastp sequence coverage: 93%

AM755	tRNA isopentenylpyrophosphate transferase (305 aa)

APH_0431	tRNA delta(2)-isopentenylpyrophosphate transferase (312 aa)

BH10930	RNA delta (2)-isopentenylpyrophosphate transferase (290 aa)

Bfl079	tRNA delta-2-isopentenylpyrophosphate (IPP) transferase (323 aa)

BR1390	tRNA delta(2)-isopentenylpyrophosphate transferase (310 aa)

BU569	tRNA delta(2)-isopentenylpyrophosphate transferase (302 aa)

CP0956	tRNA delta-2-isopentenylpyrophosphate transferase (342 aa)

CBU1082	tRNA delta(2)-isopentenylpyrophosphate transferase (311 aa)

ECH_0588	tRNA delta(2)-isopentenylpyrophosphate transferase (303 aa)

ERGA_CDS_04490	tRNA delta(2)-isopentenylpyrophosphate transferase (300 aa)

Erum4370	putative tRNA delta(2)-isopentenylpyrophoshate transferase (300 aa)

NSE_0570	tRNA delta(2)-isopentenylpyrophosphate transferase (305 aa)

RC0656	tRNA delta(2)-isopentenylpyrophosphate transferase [EC:2.5.1.8] (363 aa)

RP510	TRNA DELTA(2)-ISOPENTENYLPYROPHOSPHATE TRANSFERASE (miaA) (311 aa)

RT0495	tRNA isopentenyltransferase (311 aa)

TW609	tRNA isopentenylpyrophosphate transferase (301 aa)

miaA	Protein synthesis (322 aa)

WD0822	tRNA delta(2)-isopentenylpyrophosphate transferase (297 aa)

Wbm0636	tRNA delta(2)-isopentenylpyrophosphate transferase (297 aa)

Cluster 169

Number of proteins: 20

Average blastp percent identity: 36%

Average blastp sequence coverage: 91%

AM859	hypothetical protein (164 aa)

APH_0332	conserved hypothetical protein TIGR00250 (166 aa)

BH08220	hypothetical protein (159 aa)

Bfl250	putative endonuclease involved in recombination (142 aa)

BRA0597	conserved hypothetical protein TIGR00250 (162 aa)

BU548	hypothetical protein (135 aa)

CP0525	conserved hypothetical protein (154 aa)

CBU2094	conserved hypothetical protein TIGR00250 (143 aa)

ECH_0460	conserved hypothetical protein TIGR00250 (156 aa)

ERGA_CDS_05820	Putative Holliday junction resolvase (156 aa)

Erum5640	putative Holliday junction resolvase (156 aa)

NSE_0006	conserved hypothetical protein (159 aa)

RC0455	unknown (154 aa)

RP330	unknown (156 aa)

RT0321	possible Holliday junction resolvase (156 aa)

TW375	unknown (145 aa)

yqgF	Hypothetical proteins (144 aa)

WD0143	conserved hypothetical protein TIGR00250 (155 aa)

Wbm0696	conserved hypothetical protein TIGR00250 (155 aa)

Cluster 170

Number of proteins: 20

Average blastp percent identity: 35%

Average blastp sequence coverage: 78%

AM488	DNA primase (612 aa)

APH_0571	DNA primase (607 aa)

BH11540	DNA primase (645 aa)

Bfl057	DNA primase (588 aa)

BR1480	DNA primase (655 aa)

BU056	DNA primase (577 aa)

CP0919	DNA primase, putative (594 aa)

CBU1595	DNA primase (553 aa)

ECH_0761	DNA primase (588 aa)

ERGA_CDS_03340	DNA primase (592 aa)

Erum3310	putative DNA primase (592 aa)

NSE_0961	DNA primase (579 aa)

RC1330	DNA primase [EC:2.7.7.-] (595 aa)

RP859	DNA PRIMASE (dnaG) (616 aa)

RT0848	DNA primase (593 aa)

TW288	DNA primase (603 aa)

dnaG	DNA metabolism (582 aa)

WD0348	DNA primase (582 aa)

Wbm0194	DNA primase (586 aa)

Cluster 171

Number of proteins: 20

Average blastp percent identity: 35%

Average blastp sequence coverage: 95%

AM885	phenylalanyl-tRNA synthetase beta chain (787 aa)

APH_0305	phenylalanyl-tRNA synthetase, beta subunit (792 aa)

BH00850	Phenylalanyl-tRNA synthetase beta chain (804 aa)

Bfl356	phenylalanyl-tRNA synthetase beta chain (803 aa)

BR2123	phenylalanyl-tRNA synthetase, beta subunit (804 aa)

BU130	phenylalanyl-tRNA synthetase beta chain (795 aa)

CP0154	phenylalanyl-tRNA synthetase beta chain (792 aa)

CBU1321	phenylalanyl-tRNA synthetase, beta subunit (792 aa)

ECH_0434	phenylalanyl-tRNA synthetase, beta subunit (788 aa)

ERGA_CDS_06040	Phenylalanyl-tRNA synthetase beta chain (789 aa)

Erum5830	phenylalanyl-tRNA synthetase beta chain (789 aa)

NSE_0426	phenylalanyl-tRNA synthetase, beta subunit, putative (876 aa)

RC0582	phenylalanyl-tRNA synthetase beta chain [EC:6.1.1.20] (818 aa)

RP418	PHENYLALANYL-TRNA SYNTHETASE BETA CHAIN (pheT) (815 aa)

RT0404	phenylalanine--tRNA ligase beta subunit (815 aa)

TW168	phenylalanyl-tRNA synthetase beta chain (962 aa)

pheT	Protein synthesis (803 aa)

WD0425	phenylalanyl-tRNA synthetase, beta subunit (779 aa)

Wbm0461	phenylalanyl-tRNA synthetase, beta subunit (758 aa)

Cluster 172

Number of proteins: 20

Average blastp percent identity: 35%

Average blastp sequence coverage: 87%

AM734	ribosome-binding factor A (117 aa)

APH_0447	ribosome-binding factor A (118 aa)

BH02140	Ribosomal-binding factor A (135 aa)

Bfl105	ribosome-binding factor A (P15B protein) (117 aa)

BR2166	ribosome-binding factor A (150 aa)

BU376	ribosome-binding factor A (120 aa)

CP0439	ribosome-binding factor A (141 aa)

CBU1431	ribosome-binding factor A (119 aa)

ECH_0564	ribosome-binding factor A (114 aa)

ERGA_CDS_04800	Putative Ribosome-binding factor A (115 aa)

Erum4680	putative ribosome-binding factor A (115 aa)

NSE_0523	ribosome-binding factor A, putative (97 aa)

RC0606	ribosome-binding factor A (120 aa)

RP435	RIBOSOME-BINDING FACTOR A (rbfA) (128 aa)

RT0422	ribosome-binding factor A (127 aa)

TW159	ribosome-binding factor A (196 aa)

rbfA	Transcription (125 aa)

WD1317	ribosomal binding factor A (132 aa)

Wbm0536	Ribosome-binding factor A (114 aa)

Cluster 173

Number of proteins: 20

Average blastp percent identity: 35%

Average blastp sequence coverage: 91%

AM260	ribosomal protein L10 (163 aa)

APH_1026	ribosomal protein L10 (160 aa)

BH06080	50S ribosomal protein l10 (172 aa)

Bfl559	50S ribosomal subunit protein L10 (165 aa)

BR1246	ribosomal protein L10 (172 aa)

BU036	50S ribosomal protein L10 (165 aa)

CP0696	ribosomal protein L10 (170 aa)

CBU0228	ribosomal protein L10 (174 aa)

ECH_0954	ribosomal protein L10 (160 aa)

ERGA_CDS_01630	50S ribosomal protein L10 (160 aa)

Erum1700	50S ribosomal protein L10 (160 aa)

NSE_0680	50S ribosomal protein L10 (174 aa)

RC0179	50S ribosomal protein L10 (169 aa)

RP138	50S RIBOSOMAL PROTEIN L10 (rplJ) (169 aa)

RT0127	50S ribosomal protein L10 (169 aa)

TW712	50S ribosomal protein L10 (166 aa)

rplJ	Protein synthesis (161 aa)

WD0022	ribosomal protein L10 (172 aa)

Wbm0649	ribosomal protein L10 (172 aa)

Cluster 174

Number of proteins: 42

Average blastp percent identity: 32%

Average blastp sequence coverage: 87%

AM167	ABC efflux transporter (582 aa)

APH_0157	ABC transporter, ATP-binding protein/permease protein, putative (578 aa)

BH00480	ABC transporter, ATP-binding protein (588 aa)

BH05200	ABC transporter, ATP-binding protein (627 aa)

BH10210	ABC transporter, ATP-binding protein (595 aa)

BH15040	ABC transporter, ATP-binding protein (596 aa)

Bfl379	probable transport ATP-binding protein MsbA (583 aa)

BR0442	ABC transporter, ATP-binding/permease protein (628 aa)

BR1715	ABC transporter, ATP binding/permease protein (599 aa)

BR1544	ABC transporter, ATP-binding/permease protein, authentic frameshift (622 aa)

BR0998	beta-(1--2)glucan export ATP-binding protein (599 aa)

BRA1050	ABC transporter, ATP-binding/permease protein (609 aa)

BU479	multidrug resistance-like ATP-binding protein mdl (589 aa)

BU480	mdlB (590 aa)

CP0341	ABC transporter, ATP-binding protein (659 aa)

CBU0856	ABC transporter, ATP-binding/permease protein (589 aa)

ECH_0313	ABC transporter, permease/ATP-binding protein (580 aa)

ERGA_CDS_07090	Lipid A export ATP-binding protein (583 aa)

Erum6820	putative ABC transporter, ATP-binding and membrane-spanning protein (583 aa)

NSE_0865	ABC transporter, ATP-binding protein/permease protein, putative (587 aa)

NSE_0410	ABC transporter, ATP-binding/permease protein, putative (557 aa)

RC0270	similarity to multidrug resistance protein (Atm1) (185 aa)

RC0286	multidrug resistance ABC transporter ATP-binding protein (589 aa)

RC0534	multidrug resistance protein (587 aa)

RC1073	multidrug resistance protein (576 aa)

RP205	MITOCHONDRIAL TRANSPORTER ATM1 PRECURSOR (atm1) (609 aa)

RP214	ABC TRANSPORTER ATP-BINDING PROTEIN (abcT3) (548 aa)

RP387	PROBABLE TRANSPORT ATP-BINDING PROTEIN MSBA (msbA1) (476 aa)

RP696	PROBABLE TRANSPORT ATP-BINDING PROTEIN MSBA (msbA2) (576 aa)

RT0195	probable ATP binding cassette transporter (592 aa)

RT0205	ABC transporter ATP-binding protein (589 aa)

RT0376	lipid A export ATP-binding/permease protein MsbA (587 aa)

RT0691	lipid A export ATP-binding/permease protein MsbA (576 aa)

TW600	ABC transporter ATP-binding protein (623 aa)

TW601	ABC transporter ATP-binding protein (589 aa)

mdlB	Transport and binding proteins (577 aa)

mdl	Transport and binding proteins (582 aa)

msbA	Transport and binding proteins (581 aa)

WD0400	ABC transporter, HlyB/MsbA family, putative (568 aa)

WD0616	ABC transporter, permease/ATP-binding protein, putative (591 aa)

Wbm0203	ABC-type multidrug transport system, ATPase and permease component (568 aa)

gbw.ORF00617	multidrug resistance ABC transporter ATP binding protein predicted from 534648-533362 between Wbm0409 and Wbm0410 (428 aa)

Cluster 175

Number of proteins: 26

Average blastp percent identity: 39%

Average blastp sequence coverage: 88%

AM1277	aspartate-semialdehyde dehydrogenase (336 aa)

APH_1346	aspartate-semialdehyde dehydrogenase, putative (337 aa)

BH07530	N-acetyl-gamma-glutamyl-phosphate reductase (311 aa)

BH12890	Aspartate-semialdehyde dehydrogenase (338 aa)

Bfl574	aspartate-semialdehyde dehydrogenase (371 aa)

BR0788	N-acetyl-gamma-glutamyl-phosphate reductase (310 aa)

BRA0887	aspartate-semialdehyde dehydrogenase (344 aa)

BU048	N-acetyl-gamma-glutamyl-phosphate reductase (334 aa)

BU448	aspartate-semialdehyde dehydrogenase (371 aa)

CP0804	aspartate-semialdehyde dehydrogenase (333 aa)

CBU0875	aspartate-semialdehyde dehydrogenase (340 aa)

ECH_0016	aspartate-semialdehyde dehydrogenase (336 aa)

ECH_1017	N-acetyl-gamma-glutamyl-phosphate reductase (346 aa)

ERGA_CDS_08180	N-acetyl-gamma-glutamyl-phosphate reductase (347 aa)

ERGA_CDS_08950	Aspartate-semialdehyde dehydrogenase (88 aa)

ERGA_CDS_09430	Aspartate-semialdehyde dehydrogenase (337 aa)

Erum0060	aspartate-semialdehyde dehydrogenase (337 aa)

Erum7830	N-acetyl-gamma-glutamyl-phosphate reductase (347 aa)

NSE_0260	aspartate-semialdehyde dehydrogenase (334 aa)

RC0430	aspartate-semialdehyde dehydrogenase [EC:1.2.1.11] (338 aa)

RP316	ASPARTATE-SEMIALDEHYDE DEHYDROGENASE (asd) (338 aa)

RT0306	aspartate-semialdehyde dehydrogenase (338 aa)

TW707	aspartate-semialdehyde dehydrogenase (337 aa)

asd	Cell envelope (367 aa)

WD0954	aspartate-semialdehyde dehydrogenase (344 aa)

Wbm0042	aspartate-semialdehyde dehydrogenase (353 aa)

Cluster 176

Number of proteins: 19

Average blastp percent identity: 36%

Average blastp sequence coverage: 83%

AM042	hypothetical protein (185 aa)

APH_0075	methyltransferase, putative (182 aa)

BH02620	hypothetical protein (189 aa)

Bfl628	putative methyltransferase (196 aa)

BRA0207	conserved hypothetical protein TIGR00095 (187 aa)

BU023	hypothetical protein (191 aa)

CP0142	conserved hypothetical protein (186 aa)

CBU1899	methyltransferase, putative (193 aa)

ECH_0211	methyltransferase, putative (183 aa)

ERGA_CDS_07910	Putative Methylase (194 aa)

Erum7590	conserved hypothetical protein (194 aa)

NSE_0201	methyltransferase, putative (198 aa)

RC0809	unknown (245 aa)

RP545	unknown (236 aa)

RT0533	probable methylase (236 aa)

TW636	putative methyltransferase (187 aa)

yhhF	Hypothetical proteins (193 aa)

WD0852	methyltransferase, putative (182 aa)

Wbm0791	methyltransferase, putative (157 aa)
