# Supplementary material for: Comparative Genomics of Emerging Human Ehrlichiosis Agents
Source: PLoS Genet. 2006 Feb 17;2(2):e21. doi: 10.1371/journal.pgen.0020021 (PMC1366493; doi:10.1371/journal.pgen.0020021)
Supplement: Supplementary file 7 [file pgen.0020021.st002.doc]

**Supplementary Table 2.** Ortholog clusters (see methods) present in all the Rickettsiales but not in any other intracellular bacterium examined.

| Common Name | AMA^[1]^ | APH | ECH | ERU | WOL | WBM | NSE | RCO | RPR | RTY |
| --- | --- | --- | --- | --- | --- | --- | --- | --- | --- | --- |
| Conserved hypothetical protein | AM778  AM779  AM780 | APH_0404  APH_0405  APH_0406  APH_0407 | ECH_0523  ECH_0525  ECH_0526  ECH_0593  ECH_0606 | Erum4470  Erum5000  Erum5010 | WD0082  WD0745 | Wbm0010  Wbm0352 | NSE_0242 | RC0098 | RP068 | RT0064 |
| Conserved hypothetical protein | AM486 | APH_0565 | ECH_0764  ECH_0765 | Erum3280 | WD0715 | Wbm0450 | NSE_0938 | RC0324 | RP240 | RT0232 |
| RDD family protein | AM562 | APH_0627 | ECH_0643 | Erum4070 | WD0355 | Wbm0300 | NSE_0542 | RC0608 | RP437 | RT0424 |
| Type I secretion system ATPase | AM929 | APH_0256 | ECH_0383 | Erum6270 | WD0770 | Wbm0216 | NSE_0042 | RC0429 | RP315 | RT0305 |
| Pyridine nucleotide-disulphide oxidoreductase family protein | AM617 | APH_0734 | ECH_0649 | Erum4020 | WD0982 | Wbm0685 | NSE_0779 | RC0637 | RP514 | RT0500 |
| Transporter, putative | AM092 | APH_0077 | ECH_0212 | Erum7580 | WD0249 | Wbm0762 | NSE_0173 | RC0616 | RP443 | RT0430 |
| Type IV secretion system protein VirB9 | AM1315 | APH_1405 | ECH_0043 | Erum0290 | WD0005 | Wbm0280 | NSE_0742 | RC0388 | RP290 | RT0281 |
| Conserved hypothetical protein | AM862 | APH_0328 | ECH_0454 | Erum5670 | WD1191 | Wbm0736 | NSE_0971 | RC0542 | RP395 | RT0383 |
| Conserved hypothetical protein | AM242 | APH_1042 | ECH_0967 | Erum1600 | WD0850 | Wbm0509 | NSE_0760 | RC0950 | RP616 | RT0605 |
| Type IV secretion system protein VirB8 | AM757 | APH_0430 | ECH_0579 | Erum4410 | WD0817 | Wbm0641 | NSE_0211 | RC0385 | RP287 | RT0278 |
| Conserved hypothetical protein | AM1122 | APH_1394 | ECH_0069 | Erum0470 | WD1053 | Wbm0440 | NSE_0937 | RC0124 | RP094 | RT0043 |

1. AMA, *Anaplasma marginale*; APH, *Anaplasma phagocytophilum*; ECH, *Ehrlichia chaffeensis*; ERU, *Ehrlichia ruminantium* Welgevonden; WOL, *Wolbachia pipientis* *w*Mel; WBM, *Wolbachia* endosymbiont *w*Bm; NSE, *Neorickettsia sennetsu*; RPR, *Rickettsia prowazekii*; RCO *Rickettsia conorii*; RPR *Rickettsia prowazekii*.
