# Supplementary material for: Comparative Genomics of Emerging Human Ehrlichiosis Agents
Source: PLoS Genet. 2006 Feb 17;2(2):e21. doi: 10.1371/journal.pgen.0020021 (PMC1366493; doi:10.1371/journal.pgen.0020021)
Supplement: Supplementary file 8 [file pgen.0020021.st003.doc]

**Supplementary Table 3.** Ortholog clusters (see methods) present in all Anaplasmataceae but not the Rickettsiales or other intracellular bacterium examined.

| Common Name | AMA^[1]^ | APH | ECH | ERU | WOL | WBM | NSE |
| --- | --- | --- | --- | --- | --- | --- | --- |
| Hypothetical protein | AM578 | APH_0615  APH_0641 | ECH_0663  ECH_0664 | Erum3890  Erum3900 | WD0079 | gbw.ORF00519^[2]^ | NSE_0647 |
| Hypothetical protein | AM1051 | APH_1153 | ECH_1036  ECH_1038 | Erum7950  Erum7970 | WD1174 | Wbm0749 | NSE_0765 |
| Conserved hypothetical protein | AM445 | APH_0515 | ECH_0795 | Erum3000 | WD1304  WD0931 | Wbm0386  Wbm0498 | NSE_0038 |
| Conserved hypothetical protein | AM218 | APH_1067  APH_1102 | ECH_0987  ECH_0988 | Erum1450 | WD0772 | Wbm0214 | NSE_0788 |
| Conserved hypothetical protein | AM981 | APH_0206 | ECH_0345 | Erum6560 | WD0083 | Wbm0353 | NSE_0126 |
| Conserved hypothetical protein | AM119 | APH_0103 | ECH_0237 | Erum7400 | WD0183 | Wbm0260 | NSE_0189 |
| Conserved hypothetical protein | AM836 | APH_0351 | ECH_0477 | Erum5410 | WD0744 | Wbm0008 | NSE_0735 |
| Conserved hypothetical protein | AM500 | APH_0585 | ECH_0753 | Erum3380 | WD1056 | Wbm0437 | NSE_0571 |
| Conserved hypothetical protein | AM753 | APH_0432 | ECH_0587 | Erum4360 | WD0821 | Wbm0637 | NSE_0331 |
| Conserved hypothetical protein | AM508 | APH_0648 | ECH_0739 | Erum3450 | WD0523 | Wbm0800 | NSE_0205 |
| Acetyltransferase, GNAT family | AM303 | APH_0975 | ECH_0906 | Erum1970 | WD0729 | Wbm0598 | NSE_0658 |
| Conserved hypothetical protein | AM967 | APH_0220 | ECH_0354 | Erum6490 | WD0863 | Wbm0659 | NSE_0389 |
| Hypothetical protein | AM1292 | APH_0011 | ECH_0021 | Erum0090 | WD0827 | Wbm0765 | NSE_0564 |

1. AMA, *Anaplasma marginale*; APH, *Anaplasma phagocytophilum*; ECH, *Ehrlichia chaffeensis*; ERU, *Ehrlichia ruminantium* Welgevonden; WOL, *Wolbachia pipientis* *w*Mel; WBM, *Wolbachia* endosymbiont *w*Bm; NSE, *Neorickettsia sennetsu*.
2. An ORF predicted from 435402-435301 between Wbm0346 and Wbm0347
