# Supplementary material for: Comparative Genomics of Emerging Human Ehrlichiosis Agents
Source: PLoS Genet. 2006 Feb 17;2(2):e21. doi: 10.1371/journal.pgen.0020021 (PMC1366493; doi:10.1371/journal.pgen.0020021)
Supplement: Supplementary file 9 [file pgen.0020021.st004.doc]

**Supplementary Table 4.** Five genera in the Rickettsiales order have at least one representative sequenced. In order to compare these five genera, the ortholog clusters (see methods) from the following genomes were compared: *Rickettsia prowazekii*, *Neorickettsia sennetsu*, *Wolbachia pipientis*, *Anaplasma phagocytophilum*, and *Ehrlichia chaffeensis.*

Cluster 1

APH_0004	ABC transporter, ATP-binding protein (241 aa)

APH_0041	ABC transporter, ATP-binding protein (225 aa)

APH_0313	phosphate ABC transporter, ATP-binding protein (252 aa)

APH_0983	cation ABC transporter, ATP-binding protein, putative (220 aa)

APH_0986	ABC transporter, ATP-binding protein (342 aa)

ECH_0085	ABC transporter, ATP-binding protein (238 aa)

ECH_0174	ABC transporter, ATP-binding protein (235 aa)

ECH_0444	phosphate ABC transporter, ATP-binding protein (253 aa)

ECH_0841	cation ABC transporter, ATP-binding protein, putative (242 aa)

ECH_0845	ABC transporter, ATP-binding protein (340 aa)

NSE_0808	phosphate ABC transporter, ATP-binding protein, putative (255 aa)

NSE_0698	ABC transporter, ATP-binding protein (242 aa)

NSE_0480	ABC transporter, ATP-binding protein (337 aa)

NSE_0315	lipoprotein releasing system ATP-binding protein LolD, putative (224 aa)

RP060	ABC TRANSPORTER ATP-BINDING PROTEIN (abcT1) (555 aa)

RP097	POSSIBLE RIBONUCLEOTIDE TRANSPORT ATP-BINDING PROTEIN MKL (mkl) (252 aa)

RP508	ABC TRANSPORTER ATP-BINDING PROTEIN (abcT2) (254 aa)

RP700	GLUTAMINE TRANSPORT ATP-BINDING PROTEIN GLNQ (glnQ1) (221 aa)

RP834	FERRIC TRANSPORT ATP-BINDING PROTEIN (afuC) (233 aa)

RP868	GLUTAMINE TRANSPORT ATP-BINDING PROTEIN GLNQ (glnQ2) (240 aa)

WD0990	ABC transporter, ATP-binding protein (233 aa)

WD0153	ABC transporter, ATP-binding protein (257 aa)

WD0371	phosphate ABC transporter, ATP-binding protein (252 aa)

WD0455	amino acid ABC transporter, ATP-binding protein, putative (213 aa)

WD0707	ABC transporter, ATP-binding protein (224 aa)

WD0938	cation ABC transporter, ATP-binding protein, putative (231 aa)

Cluster 2

APH_1373	1-acyl-sn-glycerol-3-phosphate acyltransferase family protein (241 aa)

APH_0094	membrane protein, putative (391 aa)

APH_0164	bicyclomycin resistance protein (389 aa)

APH_0878	membrane protein, putative (419 aa)

ECH_0072	1-acyl-sn-glycerol-3-phosphate acyltransferase family protein (236 aa)

ECH_0230	membrane protein, putative (411 aa)

ECH_0318	drug resistance transporter, Bcr/CflA family (402 aa)

ECH_0838	conserved hypothetical protein (405 aa)

NSE_0064	drug resistance transporter, Bcr/CflA family (397 aa)

NSE_0846	membrane protein, putative (399 aa)

NSE_0407	1-acyl-sn-glycerol-3-phosphate acyltransferase family protein (264 aa)

RP054	GLYCEROL-3-PHOSPHATE TRANSPORTER (glpT) (429 aa)

RP157	MULTIDRUG RESISTANCE PROTEIN B (emrB) (519 aa)

RP375	PROLINE/BETAINE TRANSPORTER (proP3) (406 aa)

RP469	POSSIBLE 1-ACYL-SN-GLYCEROL-3-PHOSPHATE ACYLTRANSFERASE (plsC) (243 aa)

RP603	BICYCLOMYCIN RESISTANCE PROTEIN (bcr1) (407 aa)

RP620	2-ACYLGLYCEROPHOSPHOETHANOLAMINE ACYLTRANSFERASE (aas) (1152 aa)

RP698	BICYCLOMYCIN RESISTANCE PROTEIN (bcr2) (391 aa)

WD1033	permease, putative (409 aa)

WD1034	membrane protein, putative (406 aa)

WD1149	1-acyl-sn-glycerol-3-phosphate acyltransferase family protein (241 aa)

WD0056	major facilitator family transporter (395 aa)

WD0248	drug resistance transporter (387 aa)

WD0384	bicyclomycin resistance protein (400 aa)

WD0619	GlpT/PgpT/UhpT transporter family protein (396 aa)

Cluster 3

APH_0110	acetamidase/formamidase family protein (999 aa)

APH_0441	hypothetical protein (597 aa)

APH_1170	pentapeptide repeat family protein (564 aa)

ECH_0264	conserved hypothetical protein (995 aa)

ECH_0558	lipoprotein, putative (583 aa)

ECH_1083	pentapeptide repeat protein (607 aa)

NSE_0181	hypothetical protein (943 aa)

NSE_0725	pentapeptide repeat domain protein (568 aa)

NSE_0591	hypothetical protein (490 aa)

RP018	190 KD ANTIGEN PRECURSOR (sca1) (347 aa)

RP278	unknown (371 aa)

RP498	CELL SURFACE ANTIGEN (sca4) (785 aa)

RP563	unknown (588 aa)

RP674	unknown (711 aa)

RP780	unknown (835 aa)

WD1039	hypothetical protein (474 aa)

WD0073	ankyrin repeat domain protein (800 aa)

WD0117	hypothetical protein (142 aa)

WD0305	hypothetical protein (1040 aa)

WD0335	hypothetical protein (825 aa)

WD0424	hypothetical protein (792 aa)

WD0440	pentapeptide repeat domain protein (601 aa)

WD0462	hypothetical protein (440 aa)

Cluster 4

APH_0009	ubiquinone biosynthesis hydroxylase, UbiH/UbiF/VisC/COQ6 family (392 aa)

APH_0566	conserved hypothetical protein (326 aa)

APH_0769	tRNA pseudouridine synthase B (300 aa)

ECH_0020	ubiquinone biosynthesis hydroxylase, UbiH/UbiF/VisC/COQ6 family (390 aa)

ECH_0728	tRNA pseudouridine synthase B (296 aa)

ECH_0762	conserved hypothetical protein (335 aa)

NSE_0101	tRNA pseudouridine synthase B (235 aa)

NSE_0601	conserved hypothetical protein (323 aa)

NSE_0384	ubiquinone biosynthesis hydroxylase, UbiH/UbiF/VisC/COQ6 family (377 aa)

RP045	unknown (358 aa)

RP501	TRNA PSEUDOURIDINE 55 SYNTHASE (truB) (172 aa)

RP561	unknown (302 aa)

WD0177	monoxygenase family protein (382 aa)

WD0717	conserved hypothetical protein (349 aa)

WD0904	hypothetical protein (86 aa)

Cluster 5

APH_0021	hemolysin, putative (289 aa)

APH_0449	integral membrane protein, TerC family (236 aa)

APH_0957	CBS/transporter associated domain protein (422 aa)

ECH_0031	hemolysin (295 aa)

ECH_0566	membrane protein, TerC family (228 aa)

ECH_0892	CBS domain protein (433 aa)

NSE_0794	membrane protein, TerC family (224 aa)

NSE_0404	CBS domain protein (430 aa)

NSE_0327	CBS/transporter associated domain protein (252 aa)

RP702	unknown (424 aa)

RP740	HEMOLYSIN C (tlyC) (303 aa)

RP786	TELLURIUM RESISTANCE PROTEIN TERC (terC) (297 aa)

WD1027	CBS domain protein (272 aa)

WD0194	membrane protein, TerC family (235 aa)

WD0558	CBS domain protein (427 aa)

Cluster 6

APH_1300	amidophosphoribosyltransferase (468 aa)

APH_1345	membrane protein, putative (233 aa)

ECH_0139	amidophosphoribosyltransferase (462 aa)

ECH_0009	membrane protein, putative (233 aa)

NSE_0194	amidophosphoribosyltransferase (463 aa)

NSE_0050	membrane proetin, putative (251 aa)

RP147	unknown (236 aa)

WD0950	membrane protein, putative (240 aa)

WD1109	amidophosphoribosyltransferase (461 aa)

WD0535	glucosamine--fructose-6-phosphate aminotransferase (isomerizing) (606 aa)

Cluster 7

APH_1224	valyl-tRNA synthetase (806 aa)

APH_0796	isoleucyl-tRNA synthetase (1114 aa)

APH_0249.1	valyl-tRNA synthetase, truncation (92 aa)

ECH_0136	valyl-tRNA synthetase (808 aa)

ECH_0538	isoleucyl-tRNA synthetase (1122 aa)

NSE_0603	valyl-tRNA synthetase, putative (877 aa)

NSE_0537	isoleucyl-tRNA synthetase (1045 aa)

RP617	PROBABLE ISOLEUCYL-TRNA SYNTHETASE (ileS) (1086 aa)

RP687	VALYL-TRNA SYNTHETASE (valS) (814 aa)

WD0423	isoleucyl-tRNA synthetase (1111 aa)

WD0464	valyl-tRNA synthetase (864 aa)

Cluster 8

APH_0249	carbamoyl-phosphate synthase, large subunit (1086 aa)

APH_0364	propionyl-CoA carboxylase, alpha subunit (659 aa)

ECH_0378	carbamoyl-phosphate synthase, large subunit (1076 aa)

ECH_0487	propionyl-CoA carboxylase, alpha subunit (655 aa)

NSE_0222	carbamoyl-phosphate synthase, large subunit (1073 aa)

NSE_0815	propionyl-CoA carboxylase, alpha subunit (652 aa)

RP618	PROPIONYL-COA CARBOXYLASE ALPHA CHAIN PRECURSOR (pccA) (665 aa)

WD1295	carbamoyl-phosphate synthase, large subunit (1151 aa)

WD0433	propionyl-CoA carboxylase, alpha subunit (691 aa)

Cluster 9

APH_1397	heme exporter protein CcmB, putative (214 aa)

APH_1030	preprotein translocase, SecE subunit (66 aa)

ECH_0065	heme exporter protein CcmB, putative (220 aa)

ECH_0958	preprotein translocase, SecE subunit, putative (65 aa)

NSE_0161	CcmB-related protein (203 aa)

NSE_0683.1	preprotein translocase, SecE subunit (60 aa)

RP134	unknown (secE) (66 aa)

RP268	HAEM EXPORTER PROTEIN B (ccmB) (215 aa)

WD1093	heme exporter protein CcmB (215 aa)

WD0018	protein translocase subunit SecE (69 aa)

Cluster 10

APH_0258	ATP-dependent DNA helicase, UvrD/Rep family (788 aa)

APH_0903	helicase, UvrD/Rep family (659 aa)

ECH_0387	ATP-dependent DNA helicase, UvrD/Rep family (860 aa)

ECH_0860	helicase, UvrD/Rep family (639 aa)

NSE_0467	ATP-dependent DNA helicase, UvrD/REP family (907 aa)

NSE_0356	helicase, UvrD/Rep family (630 aa)

RP447	DNA HELICASE II (uvrD) (658 aa)

RP734	ATP-DEPENDENT NUCLEASE SUBUNIT A (addA) (822 aa)

WD0963	helicase II - UvrD/PcrA (638 aa)

WD0359	helicase, UvrD/Rep/AddA family (1089 aa)

Cluster 11

APH_0362	ribosomal large subunit pseudouridine synthase, RluA family (324 aa)

APH_0549	ribosomal large subunit pseudouridine synthase C (297 aa)

ECH_0486	ribosomal large subunit pseudouridine synthase, RluA family (337 aa)

ECH_0774	ribosomal large subunit pseudouridine synthase C (307 aa)

NSE_0146	ribosomal large subunit pseudouridine synthases, RluA family (330 aa)

NSE_0138	RNA pseudouridylate synthase, putative, truncation (119 aa)

NSE_0556	ribosomal large subunit pseudouridine synthase C (305 aa)

RP258	unknown (303 aa)

RP846	SFHB PROTEIN HOMOLOG (sfhB) (313 aa)

WD1162	ribosomal large subunit pseudouridine synthase, RluD subfamily (276 aa)

WD0415	ribosomal large subunit pseudouridine synthase C, putative (307 aa)

Cluster 12

APH_1023	DNA-directed RNA polymerase, beta' subunit (1409 aa)

APH_1024	DNA-directed RNA polymerase, beta subunit (1383 aa)

ECH_0951	DNA-directed RNA polymerase, beta' subunit (1410 aa)

ECH_0952	DNA-directed RNA polymerase, beta subunit (1380 aa)

NSE_0678	DNA-directed RNA polymerase, beta subunit (1358 aa)

NSE_0677	DNA-directed RNA polymerase, beta' subunit (1375 aa)

RP140	DNA-DIRECTED RNA POLYMERASE BETA CHAIN (rpoB) (1374 aa)

RP141	DNA-DIRECTED RNA POLYMERASE BETA PRIME CHAIN (rpoC) (1372 aa)

WD0024	DNA-directed RNA polymerase, beta/beta' subunits (2837 aa)

Cluster 13

APH_1197	hexapeptide transferase family protein (170 aa)

ECH_1066	hexapeptide transferase family protein (170 aa)

NSE_0045	hexapeptide transferase family protein (185 aa)

RP009	UDP-3-O-[3-HYDROXYMYRISTOYL] GLUCOSAMINE N-ACYLTRANSFERASE (lpxD) (346 aa)

RP454	UDP-N-ACETYLGLUCOSAMINE PYROPHOSPHORYLASE (glmU) (249 aa)

RP516	unknown (185 aa)

WD0133	UDP-N-acetylglucosamine pyrophosphorylase (430 aa)

WD0466	hexapeptide transferase family protein (171 aa)

Cluster 14

APH_1111	ATP-binding protein, Mrp/Nbp35 family (342 aa)

ECH_1023	conserved hypothetical protein (352 aa)

ECH_1156	chromosome partitioning ATPase, ParA family (256 aa)

NSE_0888	ATP-binding protein, Mrp/Nbp35 family (246 aa)

RP058	SOJ PROTEIN (soj) (255 aa)

RP121	unknown (318 aa)

WD1217	ParA family protein (280 aa)

WD0179	GTP/ATP binding protein, putative (340 aa)

Cluster 15

APH_0065	dihydrolipoamide dehydrogenase (471 aa)

APH_0393	dihydrolipoamide dehydrogenase (468 aa)

APH_1070	dihydrolipoamide dehydrogenase (471 aa)

ECH_0509	dihydrolipoamide dehydrogenase (463 aa)

ECH_0992	dihydrolipoamide dehydrogenase (468 aa)

NSE_0671	dihydrolipoamide dehydrogenase (468 aa)

NSE_0463	dihydrolipoamide dehydrogenase (457 aa)

RP460	DIHYDROLIPOAMIDE DEHYDROGENASE PRECURSOR (pdhD) (453 aa)

RP805	DIHYDROLIPOAMIDE DEHYDROGENASE (pdhD) (459 aa)

WD0325	alpha keto acid dehydrogenase complex, E3 component, lipoamide dehydrogenase (457 aa)

WD0751	alpha keto acid dehydrogenase complex, E3 component, lipoamide dehydrogenase (459 aa)

Cluster 16

APH_0310	4-hydroxybenzoate octaprenyltransferase (292 aa)

APH_1086	protoheme IX farnesyltransferase (296 aa)

ECH_0441	4-hydroxybenzoate octaprenyltransferase (291 aa)

ECH_1004	protoheme IX farnesyltransferase (295 aa)

NSE_0806	4-hydroxybenzoate octaprenyltransferase (280 aa)

NSE_0623	protoheme IX farnesyltransferase (302 aa)

RP346	CYTOCHROME C OXIDASE ASSEMBLY FACTOR (ctaB) (310 aa)

RP686	4-HYDROXYBENZOATE OCTAPRENYLTRANSFERASE (ubiA) (282 aa)

WD1316	4-hydroxybenzoate octaprenyltransferase (285 aa)

WD0302	protoheme IX farnesyltransferase (295 aa)

Cluster 17

APH_0080	thioredoxin (110 aa)

APH_0141	thiol:disulfide oxidoreductase, putative (166 aa)

APH_0561	thiol-disulfide oxidoreductase, putative (210 aa)

ECH_0218	thioredoxin (107 aa)

ECH_0304	thiol:disulfide oxidoreductase, putative (165 aa)

NSE_0894	thioredoxin 1 (102 aa)

RP002	THIOREDOXIN (trxA) (130 aa)

RP397	THIOL:DISULFIDE INTERCHANGE PROTEIN TLPA (tlpA) (221 aa)

WD0879	thioredoxin (107 aa)

Cluster 18

APH_0673	rrf2 family protein /cysteine desulfurase (486 aa)

APH_0674	cysteine desulfurase (412 aa)

ECH_0628	rrf2/aminotransferase, class V family protein (492 aa)

ECH_0629	cysteine desulfurase (410 aa)

NSE_0304	rrf2/aminotransferase, class V family protein (530 aa)

NSE_0303	cysteine desulfurase (402 aa)

RP486	NIFS PROTEIN HOMOLOG (spl1) (410 aa)

RP487	NIFS PROTEIN HOMOLOG (spl1) (376 aa)

WD0997	cysteine desulfurase (415 aa)

WD0705	aminotransferase, class V (378 aa)

Cluster 19

APH_0072	glutamyl-tRNA synthetase (450 aa)

APH_0408	glutamyl-tRNA synthetase (468 aa)

ECH_0208	glutamyl-tRNA synthetase (443 aa)

ECH_0605	glutamyl-tRNA synthetase (469 aa)

NSE_0737	glutamyl-tRNA synthetase (409 aa)

NSE_0518	glutamyl-tRNA synthetase (433 aa)

RP325	GLUTAMYL-TRNA SYNTHETASE (gltX1) (447 aa)

RP623	GLUTAMYL-TRNA SYNTHETASE (gltX2) (470 aa)

WD0435	glutamyl-tRNA synthetase (473 aa)

WD0778	glutamyl-tRNA synthetase (440 aa)

Cluster 20

APH_0659	antioxidant, AhpC/Tsa family (203 aa)

APH_0795	antioxidant, AhpC/Tsa family (154 aa)

ECH_0539	antioxidant, AhpC/TSA family (147 aa)

ECH_0734	antioxidant, AhpC/Tsa family (205 aa)

NSE_0723	bacterioferritin comigratory protein (162 aa)

NSE_0559	antioxidant, AhpC/Tsa family (200 aa)

RP327	THIOREDOXIN PEROXIDASE 1 (tdpX1) (200 aa)

WD1285	bacterioferritin comigratory protein (155 aa)

WD0756	antioxidant, AhpC/Tsa family (199 aa)

Cluster 21

APH_1247	octaprenyl-diphosphate synthase (316 aa)

APH_0329	geranyltranstransferase (280 aa)

ECH_0088	octaprenyl-diphosphate synthase (325 aa)

ECH_0455	geranyltranstransferase, putative (267 aa)

NSE_0238	polyprenyl synthetase family protein (321 aa)

NSE_0694	geranyltranstransferase, putative (256 aa)

RP479	OCTAPRENYL-DIPHOSPHATE SYNTHASE (ispB) (327 aa)

WD1192	geranyltranstransferase (335 aa)

WD0799	octaprenyl-diphosphate synthase (328 aa)

Cluster 22

APH_0278	translation elongation factor Tu (393 aa)

APH_1032	translation elongation factor Tu (393 aa)

ECH_0960	translation elongation factor Tu (395 aa)

ECH_0407	translation elongation factor Tu (395 aa)

NSE_0686	translation elongation factor Tu (430 aa)

RP661	ELONGATION FACTOR TU (tuf) (394 aa)

WD0017	translation elongation factor Tu (390 aa)

WD0683	translation elongation factor Tu (390 aa)

Cluster 23

APH_1001	3-phosphoshikimate 1-carboxyvinyltransferase, putative (440 aa)

ECH_0920	3-phosphoshikimate 1-carboxyvinyltransferase, putative (428 aa)

NSE_0180	3-phosphoshikimate 1-carboxyvinyltransferase, putative (435 aa)

RP579	UDP-N-ACETYLGLUCOSAMINE 1-CARBOXYVINYLTRANSFERASE (murA) (419 aa)

WD1197	UDP-N-acetylglucosamine 1-carboxyvinyltransferase (425 aa)

Cluster 24

APH_1200	modification methylase, HemK family (282 aa)

ECH_1063	modification methylase, HemK family (280 aa)

NSE_0128	modification methylase, HemK family (289 aa)

NSE_0922	tRNA (guanine-N(7)-)-methyltransferase, putative (217 aa)

RP847	POSSIBLE PROTOPORPHYRINOGEN OXIDASE (hemK) (518 aa)

WD1091	methyltransferase, putative (221 aa)

WD0010	modification methylase, HemK family (279 aa)

Cluster 25

APH_1308	pyruvate dehydrogenase complex, E1 component, beta subunit, putative (332 aa)

ECH_0149	pyruvate dehydrogenase complex, E1 component, beta subunit, putative (332 aa)

NSE_0746	pyruvate dehydrogenase complex, E1 component, beta subunit, putative (332 aa)

RP262	PYRUVATE DEHYDROGENASE E1 COMPONENT, BETA SUBUNIT PRECURSOR (326 aa)

WD0473	pyruvate dehydrogenase complex, E1 component, pyruvate dehydrogenase beta subunit, putative (332 aa)

Cluster 26

APH_1198	2-oxoglutarate dehydrogenase, E2 component, dihydrolipoamide succinyltransferase (406 aa)

ECH_1065	2-oxoglutarate dehydrogenase, E2 component, dihydrolipoamide succinyltransferase (404 aa)

NSE_0548	2-oxoglutarate dehydrogenase, E2 component, dihydrolipoamide succinyltransferase (427 aa)

RP179	DIHYDROLIPOAMIDE ACETYLTRANSFERASE COMPONENT (sucB) (401 aa)

WD0544	2-oxoglutarate dehydrogenase, E2 component, dihydrolipoamide succinyltransferase (390 aa)

Cluster 27

APH_0064	transcription termination factor Rho (452 aa)

APH_1071	transcription termination factor Rho (443 aa)

ECH_0994	transcription termination factor Rho (422 aa)

ECH_0200	transcription termination factor Rho (422 aa)

NSE_0067	transcription termination factor Rho (439 aa)

RP526	TRANSCRIPTION TERMINATION FACTOR RHO (rho) (457 aa)

WD0795	transcription termination factor Rho (435 aa)

Cluster 28

APH_0190	aspartyl-tRNA synthetase (598 aa)

ECH_0334	aspartyl-tRNA synthetase (590 aa)

NSE_0477	aspartyl-tRNA synthetase (588 aa)

RP145	ASPARAGINYL-TRNA SYNTHETASE (aspS) (605 aa)

WD0413	aspartyl-tRNA synthetase (600 aa)

Cluster 29

APH_1148	protease DO family protein (490 aa)

ECH_1052	serine protease, DO/DeqQ family (471 aa)

NSE_0166	periplasmic serine protease, DO/DeqQ family (473 aa)

RP124	PROBABLE PERIPLASMIC SERINE PROTEASE DO-LIKE PRECURSOR (htrA) (513 aa)

WD0833	protease DO (497 aa)

Cluster 30

APH_0610	glycoprotease family protein (195 aa)

ECH_0730	glycoprotease family protein (195 aa)

NSE_0844	glycoprotease family protein (196 aa)

RP551	unknown (186 aa)

WD0210	endopeptidase-related protein (191 aa)

Cluster 31

APH_0494	ATP synthase F1, beta subunit (479 aa)

ECH_0573	ATP synthase F1, beta subunit (507 aa)

NSE_0763	ATP synthase F1, beta subunit (474 aa)

RP801	ATP SYNTHASE BETA CHAIN (atpD) (474 aa)

WD0203	ATP synthase F1, beta subunit (475 aa)

Cluster 32

APH_0668	3-oxoacyl-(acyl-carrier-protein) reductase (245 aa)

ECH_0669	3-oxoacyl-(acyl-carrier-protein) reductase (247 aa)

NSE_0665	3-oxoacyl-[acyl-carrier protein] reductase (246 aa)

RP035	ACETOACETYL-COA REDUCTASE (phbB) (241 aa)

RP762	3-OXOACYL REDUCTASE (fabG) (241 aa)

WD0650	3-oxoacyl-(acyl-carrier-protein) reductase (244 aa)

Cluster 33

APH_1216	polyA polymerase/tRNA nucleotidyltransferase family protein (409 aa)

ECH_1116	polyA polymerase/tRNA nucleotidyltransferase family protein (401 aa)

NSE_0646	polyA polymerase family protein (392 aa)

RP015	POLY(A) POLYMERASE (pcnB) (387 aa)

WD0444	poly A polymerase family protein (391 aa)

Cluster 34

APH_0930	3-oxoacyl-(acyl-carrier-protein) synthase II (423 aa)

ECH_0882	3-oxoacyl-(acyl-carrier-protein) synthase II (422 aa)

NSE_0453	3-oxoacyl-(acyl-carrier-protein) synthase II (415 aa)

RP764	3-OXOACYL-[ACYL-CARRIER-PROTEIN] SYNTHASE II (fabF) (426 aa)

WD1194	3-oxoacyl-(acyl-carrier-protein) synthase II (423 aa)

Cluster 35

APH_0564	arginyl-tRNA synthetase FRAMESHIFT (57 aa)

APH_0700	arginyl-tRNA synthetase (580 aa)

APH_0701	hypothetical protein (40 aa)

ECH_0537	arginyl-tRNA synthetase (576 aa)

NSE_0049	arginyl-tRNA synthetase (570 aa)

RP065	ARGINYL-TRNA SYNTHETASE (argS) (576 aa)

WD0402	arginyl-tRNA synthetase (568 aa)

Cluster 36

APH_0448	translation elongation factor G, truncation (75 aa)

APH_1033	translation elongation factor G (690 aa)

ECH_0961	translation elongation factor G (690 aa)

NSE_0687	translation elongation factor G (692 aa)

RP132	ELONGATION FACTOR G (fusA) (699 aa)

WD0016	translation elongation factor G (691 aa)

Cluster 37

APH_0970	ATP-dependent Clp protease, proteolytic subunit ClpP (197 aa)

ECH_0901	ATP-dependent Clp protease, proteolytic subunit ClpP (199 aa)

NSE_0752	ATP-dependent Clp protease, proteolytic subunit ClpP (211 aa)

RP520	ATP-DEPENDENT CLP PROTEASE PROTEOLYTIC SUBUNIT (clpP) (201 aa)

WD0319	ATP-dependent Clp protease, proteolytic subunit ClpP (208 aa)

Cluster 38

APH_1199	methionine aminopeptidase, type I (264 aa)

ECH_1064	methionine aminopeptidase, type I (266 aa)

NSE_0337	methionine aminopeptidase, type I (262 aa)

RP824	METHIONINE AMINOPEPTIDASE (map) (259 aa)

WD0167	methionine aminopeptidase, type I (260 aa)

Cluster 39

APH_0154	serine hydroxymethyltransferase (425 aa)

ECH_0311	serine hydroxymethyltransferase (420 aa)

ECH_0333	serine hydroxymethyltransferase domain protein (68 aa)

NSE_0218	serine hydroxymethyltransferase (419 aa)

RP743	SERINE HYDROXYMETHYLTRANSFERASE (glyA) (420 aa)

WD1035	serine hydroxymethyltransferase (425 aa)

Cluster 40

APH_0013	glycyl-tRNA synthetase, alpha subunit (283 aa)

APH_0165	glycyl-tRNA synthetase, alpha subunit (283 aa)

ECH_0023	glycyl-tRNA synthetase, alpha subunit (280 aa)

NSE_0757	glycyl-tRNA synthetase, alpha subunit (305 aa)

RP850	GLYCYL-TRNA SYNTHETASE ALPHA SUBUNIT (glyQ) (289 aa)

WD0156	glycyl-tRNA synthetase, alpha subunit (279 aa)

Cluster 41

APH_0658	thioredoxin-disulfide reductase (321 aa)

ECH_0735	thioredoxin-disulfide reductase (318 aa)

NSE_0558	thioredoxin-disulfide reductase (314 aa)

RP445	THIOREDOXIN REDUCTASE (trxB1) (310 aa)

WD0755	thioredoxin-disulfide reductase (315 aa)

Cluster 42

APH_1101	inorganic pyrophosphatase (178 aa)

ECH_1014	inorganic pyrophosphatase (172 aa)

NSE_0734	inorganic pyrophosphatase (172 aa)

RP589	INORGANIC PYROPHOSPHATASE (ppa) (172 aa)

WD1263	inorganic pyrophosphatase (168 aa)

Cluster 43

APH_0694	GTP-binding protein, GTP1/Obg family (352 aa)

ECH_0543	GTP-binding protein, GTP1/Obg family (340 aa)

NSE_0444	GTP-binding protein, GTP1/Obg family (341 aa)

RP843	unknown (331 aa)

WD0493	GTP-binding protein (340 aa)

Cluster 44

APH_0716	DNA gyrase, B subunit (793 aa)

ECH_0620	DNA gyrase, B subunit (798 aa)

NSE_0721	DNA gyrase, B subunit (783 aa)

RP580	DNA GYRASE SUBUNIT B (gyrB2) (807 aa)

WD0112	DNA gyrase, B subunit (795 aa)

Cluster 45

APH_1263	translation initiation factor IF-3 (172 aa)

ECH_0007	translation initiation factor IF-3 (173 aa)

NSE_0293	translation initiation factor IF-3 (189 aa)

RP531	TRANSLATION INITIATION FACTOR IF-3 (infC) (185 aa)

WD0977	translation initiation factor IF-3 (172 aa)

Cluster 46

APH_0647	prolyl-tRNA synthetase (426 aa)

ECH_0624	conserved domain protein (41 aa)

ECH_0740	prolyl-tRNA synthetase (424 aa)

NSE_0367	prolyl-tRNA synthetase (436 aa)

RP384	PROLYL-TRNA SYNTHETASE (proS) (432 aa)

WD0813	prolyl-tRNA synthetase (422 aa)

Cluster 47

APH_0626	metalloendopeptidase, putative, glycoprotease family (342 aa)

ECH_0644	metalloendopeptidase, putative, glycoprotease family (349 aa)

NSE_0236	metalloendopeptidase, putative, glycoprotease family (329 aa)

RP037	PROBABLE O-SIALOGLYCOPROTEIN ENDOPEPTIDASE (gcp) (387 aa)

WD0699	peptidase, M22 family protein (335 aa)

Cluster 48

APH_0321	cytidine and deoxycytidylate deaminase family protein (147 aa)

ECH_0449	cytidine and deoxycytidylate deaminase family protein (140 aa)

NSE_0085	cytidine/deoxycytidylate deaminase family protein (144 aa)

RP831	unknown (148 aa)

WD0469	cytidine and deoxycytidylate deaminase family protein (135 aa)

Cluster 49

APH_0528	hydrolase, TatD family (270 aa)

APH_0530	hydrolase, TatD family, truncation (79 aa)

ECH_0793	hydrolase, TatD family (260 aa)

NSE_0357	hydrolase, TatD family (268 aa)

RP682	unknown (293 aa)

WD1120	deoxyribonuclease, TatD family (255 aa)

Cluster 50

APH_0532	hypothetical protein (44 aa)

APH_0534	leucyl-tRNA synthetase (837 aa)

ECH_0794	leucyl-tRNA synthetase (829 aa)

NSE_0506	leucyl-tRNA synthetase (851 aa)

RP421	LEUCYL-TRNA SYNTHETASE (leuS) (828 aa)

WD0060	leucyl-tRNA synthetase (838 aa)

Cluster 51

APH_1059	alanyl-tRNA synthetase (876 aa)

APH_1096	alanyl-tRNA synthetase domain protein (94 aa)

ECH_0981	alanyl-tRNA synthetase (887 aa)

NSE_0156	alanyl-tRNA synthetase (848 aa)

RP856	ALANYL-TRNA SYNTHETASE (alaS) (877 aa)

WD0862	alanyl-tRNA synthetase (877 aa)

Cluster 52

APH_0147	guanylate kinase, truncation (95 aa)

APH_0170	guanylate kinase (210 aa)

ECH_0322	guanylate kinase (209 aa)

NSE_0726	guanylate kinase (206 aa)

RP765	GUANYLATE KINASE (gmk) (197 aa)

WD0439	guanylate kinase (205 aa)

Cluster 53

APH_0802	signal peptidase I (243 aa)

ECH_0690	signal peptidase I (235 aa)

NSE_0784	signal peptidase I (252 aa)

RP116	SIGNAL PEPTIDASE I (lepB) (264 aa)

WD0893	signal peptidase I (248 aa)

Cluster 54

APH_1372	peptide deformylase (187 aa)

APH_1375	peptide deformylase, truncation (91 aa)

ECH_0073	peptide deformylase (188 aa)

NSE_0392	peptide deformylase (186 aa)

RP208	METHIONYL-TRNA DEFORMYLASE (def) (175 aa)

WD0165	peptide deformylase (179 aa)

Cluster 55

APH_0231	cytosol aminopeptidase (510 aa)

ECH_0369	cytosol aminopeptidase (500 aa)

NSE_0475	cytosol aminopeptidase (506 aa)

RP142	AMINOPEPTIDASE A (pepA) (500 aa)

WD0054	cytosol aminopeptidase (500 aa)

Cluster 56

APH_0446	translation initiation factor IF-2 (823 aa)

ECH_0563	translation initiation factor IF-2 (830 aa)

NSE_0522	translation initiation factor IF-2 (779 aa)

RP552	TRANSLATION INITIATION FACTOR IF-2 (infB) (831 aa)

WD1318	translation initiation factor IF-2 (774 aa)

Cluster 57

APH_0540	inner membrane protein, 60 kDa (568 aa)

APH_0541	membrane protein, putative (122 aa)

ECH_0782	inner-membrane protein, 60 kDa (579 aa)

NSE_0402	inner membrane protein, 60 kDa (566 aa)

RP048	60 KD INNER-MEMBRANE PROTEIN (yidC) (560 aa)

WD0237	60 kd inner-membrane protein (569 aa)

Cluster 58

APH_1097	DNA polymerase III, beta subunit (375 aa)

ECH_1009	DNA polymerase III, beta subunit (369 aa)

ECH_1010	DNA polymerase III, beta subunit domain protein (182 aa)

NSE_0170	DNA polymerase III, beta subunit (372 aa)

RP419	DNA POLYMERASE III, BETA CHAIN (dnaN) (381 aa)

WD1067	DNA polymerase III, beta subunit (365 aa)

Cluster 59

APH_0036	co-chaperone GrpE (211 aa)

ECH_0168	co-chaperone GrpE (203 aa)

NSE_0212	co-chaperone GrpE (187 aa)

RP629	GRPE PROTEIN (grpE) (178 aa)

WD0800	heat shock protein GrpE (189 aa)

Cluster 60

APH_0782	Sua5/YciO/YrdC/YwlC family protein (200 aa)

ECH_0802	Sua5/YciO/YrdC/YwlC family protein (194 aa)

NSE_0579	Sua5/YciO/YrdC/YwlC family protein (195 aa)

RP848	unknown (313 aa)

WD0820	Sua5/YciO/YrdC/YwlC family protein (195 aa)

Cluster 61

APH_1035	ribosomal protein S12 (124 aa)

ECH_0963	ribosomal protein S12 (123 aa)

NSE_0689	ribosomal protein S12 (123 aa)

RP130	30S RIBOSOMAL PROTEIN S12 (rpsL) (129 aa)

WD0014	ribosomal protein S12 (124 aa)

Cluster 62

APH_0346	chaperone protein DnaK (645 aa)

ECH_0471	chaperone protein DnaK (635 aa)

NSE_0019	chaperone protein DnaK (636 aa)

RP185	DNAK PROTEIN (dnaK) (627 aa)

WD0928	dnaK protein (640 aa)

Cluster 63

APH_0698	ribosomal protein L27 (91 aa)

ECH_0546	ribosomal protein L27 (88 aa)

NSE_0897	ribosomal protein L27 (82 aa)

RP752	50S RIBOSOMAL PROTEIN L27 (rpmA) (86 aa)

WD0135	ribosomal protein L27 (89 aa)

Cluster 64

APH_0290	ribosomal protein L14 (119 aa)

ECH_0419	ribosomal protein L14 (119 aa)

NSE_0276	ribosomal protein L14 (119 aa)

RP649	50S RIBOSOMAL PROTEIN L14 (rplN) (122 aa)

WD0671	ribosomal protein L14 (119 aa)

Cluster 65

APH_0969	ATP-dependent Clp protease, ATP-binding subunit ClpX (415 aa)

ECH_0900	ATP-dependent Clp protease, ATP-binding subunit ClpX (406 aa)

NSE_0753	ATP-dependent Clp protease, ATP-binding subunit ClpX (400 aa)

RP692	ATP-DEPENDENT CLP PROTEASE ATP-BINDING SUBUNIT CLPX (clpX) (425 aa)

WD0318	ATP-dependent Clp protease, ATP-binding subunit ClpX (425 aa)

Cluster 66

APH_0283	ribosomal protein L2 (276 aa)

ECH_0412	ribosomal protein L2 (276 aa)

NSE_0269	ribosomal protein L2 (279 aa)

RP656	50S RIBOSOMAL PROTEIN L2 (rplB) (273 aa)

WD0678	ribosomal protein L2 (274 aa)

Cluster 67

APH_0765	GTP-binding protein LepA (598 aa)

ECH_0724	GTP-binding protein LepA (598 aa)

NSE_0771	GTP-binding protein LepA (596 aa)

RP275	GTP-BINDING PROTEIN LEPA (lepA) (600 aa)

WD0477	GTP-binding protein LepA (598 aa)

Cluster 68

APH_0240	60 kDa chaperonin (550 aa)

ECH_0365	chaperonin, 60 kd (550 aa)

NSE_0642	60 kDa chaperonin (548 aa)

RP626	60 KD CHAPERONIN (groEL) (550 aa)

WD0307	chaperonin, 60 kDa (549 aa)

Cluster 69

APH_1179	ATP-dependent metalloprotease FtsH (611 aa)

ECH_1098	ATP-dependent metalloprotease FtsH (610 aa)

NSE_0423	ATP-dependent metalloprotease FtsH (636 aa)

RP043	CELL DIVISION PROTEIN FTSH (ftsH) (637 aa)

WD1253	cell division protein FtsH (613 aa)

Cluster 70

APH_0301	ribosomal protein S13 (123 aa)

ECH_0430	ribosomal protein S13 (123 aa)

NSE_0287	ribosomal protein S13 (123 aa)

RP637	30S RIBOSOMAL PROTEIN S13 (rpsM) (125 aa)

WD0660	ribosomal protein S13 (122 aa)

Cluster 71

APH_0279	ribosomal protein S10 (105 aa)

ECH_0408	ribosomal protein S10 (108 aa)

NSE_0265	ribosomal protein S10 (103 aa)

RP660	30S RIBOSOMAL PROTEIN S10 (rpsJ) (105 aa)

WD0682	ribosomal protein S10 (109 aa)

Cluster 72

APH_0060	ribosomal protein L20 (128 aa)

ECH_0197	ribosomal protein L20 (124 aa)

NSE_0215	ribosomal protein L20 (117 aa)

RP609	CYANELLE 50S RIBOSOMAL PROTEIN L20 (rplT) (117 aa)

WD0865	ribosomal protein L20 (121 aa)

Cluster 73

APH_0286	ribosomal protein S3 (211 aa)

ECH_0415	ribosomal protein S3 (211 aa)

NSE_0272	ribosomal protein S3 (211 aa)

RP653	30S RIBOSOMAL PROTEIN S3 (rpsC) (217 aa)

WD0675	ribosomal protein S3 (204 aa)

Cluster 74

APH_0287	ribosomal protein L16 (137 aa)

ECH_0416	ribosomal protein L16 (136 aa)

NSE_0273	ribosomal protein L16 (139 aa)

RP652	50S RIBOSOMAL PROTEIN L16 (rplP) (136 aa)

WD0674	ribosomal protein L16 (137 aa)

Cluster 75

APH_0284	ribosomal protein S19 (94 aa)

ECH_0413	ribosomal protein S19 (93 aa)

NSE_0270	ribosomal protein S19 (91 aa)

RP655	30S RIBOSOMAL PROTEIN S19 (rpsS) (92 aa)

WD0677	ribosomal protein S19 (94 aa)

Cluster 76

APH_0292	ribosomal protein L5 (177 aa)

ECH_0421	ribosomal protein L5 (177 aa)

NSE_0278	ribosomal protein L5 (177 aa)

RP647	50S RIBOSOMAL PROTEIN L5 (rplE) (179 aa)

WD0669	ribosomal protein L5 (177 aa)

Cluster 77

APH_0395	translation initiation factor IF-1 (81 aa)

ECH_0511	translation initiation factor IF-1 (82 aa)

NSE_0882	translation initiation factor IF-1 (93 aa)

RP814	TRANSLATION INITIATION FACTOR IF-1 (infA) (71 aa)

WD0334	translation initiation factor IF-1 (87 aa)

Cluster 78

APH_0420	peptide chain release factor 1 (359 aa)

ECH_0597	peptide chain release factor 1 (359 aa)

NSE_0406	peptide chain release factor 1 (367 aa)

RP529	PEPTIDE CHAIN RELEASE FACTOR 1 (prfA) (355 aa)

WD0247	peptide chain release factor 1 (359 aa)

Cluster 79

APH_1178	preprotein translocase, SecA subunit (873 aa)

ECH_1149	preprotein translocase, SecA subunit (862 aa)

NSE_0232	preprotein translocase, SecA subunit (804 aa)

RP575	PREPROTEIN TRANSLOCASE SECA SUBUNIT (secA) (906 aa)

WD0549	preprotein translocase, SecA subunit (886 aa)

Cluster 80

APH_0302	ribosomal protein S11 (124 aa)

ECH_0431	ribosomal protein S11 (126 aa)

NSE_0288	ribosomal protein S11 (120 aa)

RP636	30S RIBOSOMAL PROTEIN S11 (rpsK) (127 aa)

WD0659	ribosomal protein S11 (128 aa)

Cluster 81

APH_1107	ribosomal protein S9 (149 aa)

ECH_1018	ribosomal protein S9 (152 aa)

NSE_0839	ribosomal protein S9 (145 aa)

RP234	30S RIBOSOMAL PROTEIN S9 (rpsI) (161 aa)

WD0066	ribosomal protein S9 (151 aa)

Cluster 82

APH_0297	ribosomal protein S5 (173 aa)

ECH_0426	ribosomal protein S5 (174 aa)

NSE_0283	ribosomal protein S5 (172 aa)

RP642	30S RIBOSOMAL PROTEIN S5 (rpsE) (175 aa)

WD0664	ribosomal protein S5 (170 aa)

Cluster 83

APH_0925	ribosomal protein L33 (56 aa)

ECH_0876	ribosomal protein L33 (56 aa)

NSE_0257	ribosomal protein L33 (59 aa)

RP879	50S RIBOSOMAL PROTEIN L33 (rpmG) (56 aa)

WD0930	ribosomal protein L33 (66 aa)

Cluster 84

APH_1034	ribosomal protein S7 (160 aa)

ECH_0962	ribosomal protein S7 (160 aa)

NSE_0688	ribosomal protein S7 (175 aa)

RP131	30S RIBOSOMAL PROTEIN S7 (rpsG) (160 aa)

WD0015	ribosomal protein S7 (159 aa)

Cluster 85

APH_0152	ribosomal protein S18 (96 aa)

ECH_0309	ribosomal protein S18, putative (95 aa)

NSE_0061	ribosomal protein S18 (73 aa)

RP040	30S RIBOSOMAL PROTEIN S18 (rpsR) (95 aa)

WD0782	ribosomal protein S18 (91 aa)

Cluster 86

APH_0038	CTP synthase (541 aa)

ECH_0171	CTP synthase (532 aa)

NSE_0877	CTP synthase (548 aa)

RP378	CTP SYNTHASE (pyrG) (586 aa)

WD0468	CTP synthase (529 aa)

Cluster 87

APH_0397	ribosomal protein S2 (296 aa)

ECH_0514	ribosomal protein S2 (288 aa)

NSE_0948	ribosomal protein S2 (298 aa)

RP086	30S RIBOSOMAL PROTEIN S2 (rpsB) (296 aa)

WD0532	ribosomal protein S2 (282 aa)

Cluster 88

APH_1108	ribosomal protein L13 (158 aa)

ECH_1019	ribosomal protein L13 (156 aa)

NSE_0840	ribosomal protein L13 (152 aa)

RP233	50S RIBOSOMAL PROTEIN L13 (rplM) (155 aa)

WD0067	ribosomal protein L13 (152 aa)

Cluster 89

APH_0054	ribosomal protein S16 (96 aa)

ECH_0192	ribosomal protein S16 (87 aa)

NSE_0123	ribosomal protein S16 (85 aa)

RP878	30S RIBOSOMAL PROTEIN S16 (rpsP) (111 aa)

WD0798	ribosomal protein S16 (106 aa)

Cluster 90

APH_0768	ribosomal protein S15 (93 aa)

ECH_0727	ribosomal protein S15 (93 aa)

NSE_0058	ribosomal protein S15 (86 aa)

RP503	30S RIBOSOMAL PROTEIN S15 (rpsO) (91 aa)

WD0905	ribosomal protein S15 (90 aa)

Cluster 91

APH_0304	ribosomal protein L17 (132 aa)

ECH_0433	ribosomal protein L17 (133 aa)

NSE_0290	ribosomal protein L17 (117 aa)

RP634	50S RIBOSOMAL PROTEIN L17 (rplQ) (136 aa)

WD0657	ribosomal protein L17 (142 aa)

Cluster 92

APH_0293	ribosomal protein S14p/S29e (101 aa)

ECH_0422	ribosomal protein S14p (101 aa)

NSE_0279	ribosomal protein S14 (101 aa)

RP646	CYANELLE 30S RIBOSOMAL PROTEIN S14 (rpsN) (101 aa)

WD0668	ribosomal protein S14 (102 aa)

Cluster 93

APH_0061	ribosomal protein L35 (66 aa)

ECH_0198	ribosomal protein L35 (66 aa)

NSE_0214	ribosomal protein L35 (68 aa)

RP608	50S RIBOSOMAL PROTEIN L35 (rpmI) (67 aa)

WD0864	ribosomal protein L35 (68 aa)

Cluster 94

APH_0289	ribosomal protein S17 (76 aa)

ECH_0418	ribosomal protein S17 (75 aa)

NSE_0275	ribosomal protein S17 (74 aa)

RP650	30S RIBOSOMAL PROTEIN S17 (rpsQ) (77 aa)

WD0672	ribosomal protein S17 (78 aa)

Cluster 95

APH_1285	GTP-binding protein YchF (363 aa)

ECH_0154	GTP-binding protein YchF (362 aa)

NSE_0910	GTP-binding protein YchF (350 aa)

RP604	unknown (365 aa)

WD0442	GTP-binding protein YchF (364 aa)

Cluster 96

APH_0465	single-strand binding protein (159 aa)

ECH_0815	single-strand binding protein (157 aa)

NSE_0759	single-strand binding protein (151 aa)

RP836	SINGLE-STRAND BINDING PROTEIN (ssb) (152 aa)

WD0774	single-strand binding protein (160 aa)

Cluster 97

APH_1266	ribosomal protein L19 (128 aa)

ECH_0004	ribosomal protein L19 (125 aa)

NSE_0895	ribosomal protein L19 (120 aa)

RP112	50S RIBOSOMAL PROTEIN L19 (rplS) (138 aa)

WD1021	ribosomal protein L19 (125 aa)

Cluster 98

APH_0379	deoxyuridine 5'-triphosphate nucleotidohydrolase (146 aa)

ECH_0501	deoxyuridine 5'triphosphate nucleotidohydrolase (155 aa)

NSE_0957	deoxyuridine 5'triphosphate nucleotidohydrolase (149 aa)

RP399	DEOXYURIDINE 5-TRIPHOSPHATE NUCLEOTIDOHYDROLASE (dut) (148 aa)

WD0243	deoxyuridine 5'-triphosphate nucleotidohydrolase (153 aa)

Cluster 99

APH_1333	transcription elongation factor GreA (166 aa)

ECH_0133	transcription elongation factor GreA (162 aa)

NSE_0669	transcription elongation factor GreA (159 aa)

RP861	TRANSCRIPTION ELONGATION FACTOR GREA (greA) (162 aa)

WD0654	transcription elongation factor GreA (164 aa)

Cluster 100

APH_1264	threonyl-tRNA synthetase (615 aa)

ECH_0006	threonyl-tRNA synthetase (633 aa)

NSE_0294	threonyl-tRNA synthetase (637 aa)

RP221	THREONYL-TRNA SYNTHETASE (thrS) (635 aa)

WD0978	threonyl-tRNA synthetase (633 aa)

Cluster 101

APH_0303	DNA-directed RNA polymerase, alpha subunit (344 aa)

ECH_0432	DNA-directed RNA polymerase, alpha subunit (341 aa)

NSE_0289	DNA-directed RNA polymerase, alpha subunit (333 aa)

RP635	DNA-DIRECTED RNA POLYMERASE ALPHA CHAIN (rpoA) (340 aa)

WD0658	DNA-directed RNA polymerase, alpha subunit (355 aa)

Cluster 102

APH_0015	chaperone protein DnaJ (382 aa)

ECH_0025	chaperone protein DnaJ (380 aa)

NSE_0535	chaperone protein DnaJ (379 aa)

RP184	DNAJ PROTEIN (dnaJ) (370 aa)

WD0040	dnaJ protein (372 aa)

Cluster 103

APH_0175	methylenetetrahydrofolate dehydrogenase/methenyltetrahydrofolate cyclohydrolase (304 aa)

ECH_0324	FolD bifunctional protein (300 aa)

NSE_0821	FolD bifunctional protein (298 aa)

RP515	METHYLENETETRAHYDROFOLATE DEHYDROGENASE (folD) (310 aa)

WD0555	methylenetetrahydrofolate dehydrogenase/methenyltetrahydrofolate cyclohydrolase (292 aa)

Cluster 104

APH_0899	DNA gyrase, A subunit (906 aa)

ECH_0858	DNA gyrase, A subunit (898 aa)

NSE_0958	DNA gyrase, A subunit (869 aa)

RP206	DNA GYRASE SUBUNIT A (gyrA) (905 aa)

WD1202	DNA gyrase, A subunit (900 aa)

Cluster 105

APH_0767	polyribonucleotide nucleotidyltransferase (838 aa)

ECH_0726	polyribonucleotide nucleotidyltransferase (796 aa)

NSE_0057	polyribonucleotide nucleotidyltransferase (757 aa)

RP504	POLYRIBONUCLEOTIDE NUCLEOTIDYLTRANSFERASE (pnp) (745 aa)

WD0906	polyribonucleotide nucleotidyltransferase (757 aa)

Cluster 106

APH_0090	50S ribosomal protein L31, putative (73 aa)

ECH_0226	ribosomal protein L31 (75 aa)

NSE_0503	conserved hypothetical protein (76 aa)

RP100	50S RIBOSOMAL PROTEIN L31 (rpmE) (78 aa)

WD0870	ribosomal protein L31, putative (67 aa)

Cluster 107

APH_0241	10 kDa chaperonin (94 aa)

ECH_0364	chaperonin, 10 kDa (94 aa)

NSE_0643	10 kDa chaperonin (98 aa)

RP627	10 KD CHAPERONIN (groES) (95 aa)

WD0308	chaperonin, 10 kDa (96 aa)

Cluster 108

APH_0427	seryl-tRNA synthetase (428 aa)

ECH_0584	seryl-tRNA synthetase (426 aa)

NSE_0569	seryl-tRNA synthetase (415 aa)

RP783	SERYL-TRNA SYNTHETASE (serS) (425 aa)

WD0028	seryl-tRNA synthetase (425 aa)

Cluster 109

APH_0294	ribosomal protein S8 (133 aa)

ECH_0423	ribosomal protein S8 (132 aa)

NSE_0280	ribosomal protein S8 (131 aa)

RP645	30S RIBOSOMAL PROTEIN S8 (rpsH) (132 aa)

WD0667	ribosomal protein S8 (131 aa)

Cluster 110

APH_1028	ribosomal protein L11 (148 aa)

ECH_0956	ribosomal protein L11 (147 aa)

NSE_0682	ribosomal protein L11 (140 aa)

RP136	50S RIBOSOMAL PROTEIN L11 (rplK) (145 aa)

WD0020	ribosomal protein L11 (146 aa)

Cluster 111

APH_0299	preprotein translocase, SecY subunit (434 aa)

ECH_0428	preprotein translocase, SecY subunit (432 aa)

NSE_0285	preprotein translocase, SecY subunit (436 aa)

RP639	PREPROTEIN TRANSLOCASE SECY SUBUNIT (secY) (433 aa)

WD0662	preprotein translocase, SecY subunit (442 aa)

Cluster 112

APH_0285	ribosomal protein L22 (112 aa)

ECH_0414	ribosomal protein L22 (110 aa)

NSE_0271	ribosomal protein L22 (113 aa)

RP654	50S RIBOSOMAL PROTEIN L22 (rplV) (119 aa)

WD0676	ribosomal protein L22 (115 aa)

Cluster 113

APH_0280	ribosomal protein L3 (220 aa)

ECH_0409	ribosomal protein L3 (231 aa)

NSE_0266	ribosomal protein L3 (210 aa)

RP659	50S RIBOSOMAL PROTEIN L3 (rplC) (216 aa)

WD0681	ribosomal protein L3 (240 aa)

Cluster 114

APH_1331	SsrA-binding protein (149 aa)

ECH_0135	SsrA-binding protein (149 aa)

NSE_0811	SsrA-binding protein (147 aa)

RP430	SMALL PROTEIN B HOMOLOG (smpB) (152 aa)

WD0767	SsrA-binding protein (149 aa)

Cluster 115

APH_0989	ribosomal protein S4 (204 aa)

ECH_0912	ribosomal protein S4 (202 aa)

NSE_0583	ribosomal protein S4 (205 aa)

RP345	30S RIBOSOMAL PROTEIN S4 (rpsD) (205 aa)

WD0388	ribosomal protein S4 (204 aa)

Cluster 116

APH_0291	ribosomal protein L24 (104 aa)

ECH_0420	ribosomal protein L24 (109 aa)

NSE_0277	ribosomal protein L24 (104 aa)

RP648	50S RIBOSOMAL PROTEIN L24 (rplX) (113 aa)

WD0670	ribosomal protein L24 (105 aa)

Cluster 117

APH_0576	RNA polymerase sigma factor RpoD (627 aa)

ECH_0760	RNA polymerase sigma factor RpoD (622 aa)

NSE_0312	RNA polymerase sigma factor RpoD (620 aa)

RP858	RNA POLYMERASE SIGMA FACTOR RPOD (rpoD) (635 aa)

WD1298	RNA polymerase sigma factor RpoD (642 aa)

Cluster 118

APH_0929	acyl carrier protein, putative (84 aa)

ECH_0881	acyl carrier protein (91 aa)

NSE_0452	acyl carrier protein (144 aa)

RP763	ACYL CARRIER PROTEIN (acpP) (86 aa)

WD1193	acyl carrier protein (86 aa)

Cluster 119

APH_1027	ribosomal protein L1 (216 aa)

ECH_0955	ribosomal protein L1 (220 aa)

NSE_0681	ribosomal protein L1 (209 aa)

RP137	50S RIBOSOMAL PROTEIN L1 (rplA) (238 aa)

WD0021	ribosomal protein L1 (217 aa)

Cluster 120

APH_1227	signal recognition particle-docking protein FtsY (305 aa)

ECH_1111	signal recognition particle-docking protein FtsY (310 aa)

NSE_0545	signal recognition particle-docking protein FtsY (309 aa)

RP775	CELL DIVISION PROTEIN FTSY (ftsY) (303 aa)

WD0888	cell division ABC transporter, periplasmic substrate-binding protein FtsY (303 aa)

Cluster 121

APH_0059	phenylalanyl-tRNA synthetase, alpha subunit (342 aa)

ECH_0196	phenylalanyl-tRNA synthetase, alpha subunit (346 aa)

NSE_0216	phenylalanyl-tRNA synthetase, alpha subunit (325 aa)

RP417	PHENYLALANYL-TRNA SYNTHETASE ALPHA CHAIN (pheS) (350 aa)

WD0132	phenylalanyl-tRNA synthetase, alpha subunit (347 aa)

Cluster 122

APH_0359	ribosomal protein L28 (103 aa)

ECH_0484	50S ribosomal protein L28 (100 aa)

NSE_0321	ribosomal protein L28 (102 aa)

RP099	50S RIBOSOMAL PROTEIN L28 (rpmB) (97 aa)

WD0391	ribosomal protein L28 (104 aa)

Cluster 123

APH_0349	signal recognition particle protein (449 aa)

ECH_0475	signal recognition particle protein (448 aa)

NSE_0540	signal recognition particle protein (449 aa)

RP173	SIGNAL RECOGNITION PARTICLE PROTEIN (ffh) (449 aa)

WD1080	signal recognition particle protein (447 aa)

Cluster 124

APH_1025	ribosomal protein L7/L12 (134 aa)

ECH_0953	ribosomal protein L7/L12 (133 aa)

NSE_0679	ribosomal protein L7/L12 (137 aa)

RP139	50S RIBOSOMAL PROTEIN L7/L12 (rplL) (125 aa)

WD0023	ribosomal protein L7/L12 (133 aa)

Cluster 125

APH_0323	replicative DNA helicase (485 aa)

ECH_0451	replicative DNA helicase (488 aa)

NSE_0551.1	replicative DNA helicase (494 aa)

RP542	PROBABLE REPLICATIVE DNA HELICASE (dnaB) (486 aa)

WD0354	replicative DNA helicase (483 aa)

Cluster 126

APH_0273	ribosomal protein S1 (542 aa)

ECH_0402	ribosomal protein S1, putative (567 aa)

NSE_0408	ribosomal protein S1 (553 aa)

RP521	30S RIBOSOMAL PROTEIN S1 (rpsA) (568 aa)

WD1090	ribosomal protein S1, putative (550 aa)

Cluster 127

APH_0298	ribosomal protein L15 (145 aa)

ECH_0427	ribosomal protein L15 (156 aa)

NSE_0284	ribosomal protein L15 (149 aa)

RP640	50S RIBOSOMAL PROTEIN L15 (rplO) (150 aa)

WD0663	ribosomal protein L15 (156 aa)

Cluster 128

APH_0910	tRNA (5-methylaminomethyl-2-thiouridylate)-methyltransferase (378 aa)

ECH_0872	tRNA (5-methylaminomethyl-2-thiouridylate)-methyltransferase (367 aa)

NSE_0468	tRNA (5-methylaminomethyl-2-thiouridylate)-methyltransferase (371 aa)

RP306	unknown (358 aa)

WD1259	tRNA (5-methylaminomethyl-2-thiouridylate)-methyltransferase (370 aa)

Cluster 129

APH_0035	tryptophanyl-tRNA synthetase (331 aa)

ECH_0167	tryptophanyl-tRNA synthetase (333 aa)

NSE_0213	tryptophanyl-tRNA synthetase (330 aa)

RP468	TRYPTOPHANYL-TRNA SYNTHETASE (trpS) (330 aa)

WD0801	tryptophanyl-tRNA synthetase (332 aa)

Cluster 130

APH_0690	S-adenosyl-methyltransferase MraW (295 aa)

ECH_0542	S-adenosyl-methyltransferase MraW (297 aa)

NSE_0921	S-adenosyl-methyltransferase MraW (296 aa)

RP569	unknown (306 aa)

WD0170	S-adenosyl-methyltransferase MraW (314 aa)

Cluster 131

APH_0860	HIT family protein (124 aa)

ECH_0826	HIT family protein (116 aa)

NSE_0413	HIT domain protein (117 aa)

RP317	PROTEIN KINASE C INHIBITOR 1 (pkcI) (120 aa)

WD1125	HIT family protein (122 aa)

Cluster 132

APH_1374	ribosomal protein S20 (96 aa)

ECH_0071	ribosomal protein S20 (95 aa)

NSE_0792	ribosomal protein S20 (87 aa)

RP633	30S RIBOSOMAL PROTEIN S20 (rpsT) (90 aa)

WD1150	ribosomal protein S20 (89 aa)

Cluster 133

APH_0371	Fe superoxide dismutase (206 aa)

ECH_0493	superoxide dismutase, Fe (205 aa)

NSE_0843	superoxide dismutase, Fe (205 aa)

RP535	SUPEROXIDE DISMUTASE (sodB) (209 aa)

WD0738	superoxide dismutase, Fe (206 aa)

Cluster 134

APH_0555	cysteinyl-tRNA synthetase (462 aa)

ECH_0768	cysteinyl-tRNA synthetase (459 aa)

NSE_0333	cysteinyl-tRNA synthetase (507 aa)

RP085	CYSTEINYL-TRNA SYNTHETASE (cysS) (457 aa)

WD0149	cysteinyl-tRNA synthetase (457 aa)

Cluster 135

APH_0502	tRNA pseudouridine synthase A (246 aa)

ECH_0622	tRNA pseudouridine synthase A (245 aa)

NSE_0020	tRNA pseudouridine synthase A (243 aa)

RP857	PSEUDOURIDYLATE SYNTHASE I (truA) (253 aa)

WD1068	tRNA pseudouridine synthase A (245 aa)

Cluster 136

APH_0296	50S ribosomal protein L18 (121 aa)

ECH_0425	ribosomal protein L18 (121 aa)

NSE_0282	ribosomal protein L18 (117 aa)

RP643	50S RIBOSOMAL PROTEIN L18 (rplR) (118 aa)

WD0665	ribosomal protein L18 (123 aa)

Cluster 137

APH_1244	tyrosyl-tRNA synthetase (412 aa)

ECH_0091	tyrosyl-tRNA synthetase (418 aa)

NSE_0345	tyrosyl-tRNA synthetase (391 aa)

RP556	TYROSYL-TRNA SYNTHETASE (tyrS) (411 aa)

WD1115	tyrosyl-tRNA synthetase (416 aa)

Cluster 138

APH_1376	prolipoprotein diacylglyceryl transferase (277 aa)

ECH_1101	prolipoprotein diacylglyceryl transferase (280 aa)

NSE_0854	prolipoprotein diacylglyceryl transferase (266 aa)

RP046	PROLIPOPROTEIN DIACYLGLYCERYL TRANSFERASE (lgt) (268 aa)

WD0768	prolipoprotein diacylglyceryl transferase (263 aa)

Cluster 139

APH_0697	ribosomal protein L21 (99 aa)

ECH_0545	ribosomal protein L21 (102 aa)

NSE_0898	ribosomal protein L21 (99 aa)

RP751	50S RIBOSOMAL PROTEIN L21 (rplU) (105 aa)

WD0134	ribosomal protein L21 (100 aa)

Cluster 140

APH_0445	N utilization substance protein A (514 aa)

ECH_0562	N utilization substance protein A (517 aa)

NSE_0521	N utilization substance protein A (537 aa)

RP553	N UTILIZATION SUBSTANCE PROTEIN A (nusA) (503 aa)

WD1319	N utilization substance protein A (520 aa)

Cluster 141

APH_0345	ribonuclease, Rne/Rng family (636 aa)

ECH_0470	ribonuclease, Rne/Rng family (606 aa)

NSE_0120	ribonuclease, Rne/Rng family (549 aa)

RP256	RIBONUCLEASE E (rne) (683 aa)

WD0929	ribonuclease, Rne/Rng family protein (587 aa)

Cluster 142

APH_0295	ribosomal protein L6 (180 aa)

ECH_0424	ribosomal protein L6 (144 aa)

NSE_0281	ribosomal protein L6 (176 aa)

RP644	50S RIBOSOMAL PROTEIN L6 (rplF) (177 aa)

WD0666	ribosomal protein L6 (181 aa)

Cluster 143

APH_0281	ribosomal protein L4 (208 aa)

ECH_0410	ribosomal protein L4 (205 aa)

NSE_0267	ribosomal protein L4 (214 aa)

RP658	50S RIBOSOMAL PROTEIN L4 (rplD) (207 aa)

WD0680	ribosomal protein L4 (204 aa)

Cluster 144

APH_0282	ribosomal protein L23 (96 aa)

ECH_0411	ribosomal protein L23 (96 aa)

NSE_0268	ribosomal protein L23 (94 aa)

RP657	50S RIBOSOMAL PROTEIN L23 (rplW) (98 aa)

WD0679	ribosomal protein L23 (95 aa)

Cluster 145

APH_0112	ribosome recycling factor (184 aa)

ECH_0267	ribosome recycling factor (185 aa)

NSE_0945	ribosome recycling factor (185 aa)

RP154	RIBOSOME RECYCLING FACTOR (rrf) (186 aa)

WD0529	ribosome recycling factor (185 aa)

Cluster 146

APH_1149	ribonuclease III (236 aa)

ECH_1054	ribonuclease III (226 aa)

NSE_0918	ribonuclease III (222 aa)

RP117	RIBONUCLEASE III (rnc) (225 aa)

WD1240	ribonuclease III, putative (232 aa)

Cluster 147

APH_0001	DNA polymerase I (853 aa)

ECH_0080	DNA polymerase I (944 aa)

NSE_0675	DNA polymerase I (829 aa)

RP776	DNA POLYMERASE I (polA) (867 aa)

WD1003	DNA polymerase I (858 aa)

Cluster 148

APH_1029	transcription termination/antitermination factor NusG (186 aa)

ECH_0957	transcription antitermination protein NusG (179 aa)

NSE_0683	transcription termination/antitermination factor NusG, putative (176 aa)

RP135	TRANSCRIPTION ANTITERMINATION PROTEIN NUSG (nusG) (192 aa)

WD0019	transcription antitermination protein NusG, putative (196 aa)

Cluster 149

APH_0547	translation elongation factor P (188 aa)

ECH_0777	translation elongation factor P (189 aa)

NSE_0196	translation elongation factor P (191 aa)

RP238	ELONGATION FACTOR P (efp) (188 aa)

WD1281	translation elongation factor P (188 aa)

Cluster 150

APH_1003	DNA polymerase III, alpha subunit (1127 aa)

ECH_0922	DNA polymerase III, alpha subunit (1120 aa)

NSE_0167	DNA polymerase III, alpha subunit (1077 aa)

RP778	DNA POLYMERASE III, ALPHA CHAIN (dnaE) (1182 aa)

WD0780	DNA polymerase III, alpha subunit (1109 aa)

Cluster 151

APH_0138	DNA ligase, NAD-dependent (677 aa)

ECH_0301	DNA ligase, NAD-dependent (676 aa)

NSE_0500	DNA ligase, NAD-dependent (670 aa)

RP720	DNA LIGASE (lig) (689 aa)

WD0776	DNA ligase, NAD-dependent (662 aa)

Cluster 152

APH_0560	ribonucleoside-diphosphate reductase, beta subunit (323 aa)

ECH_0766	ribonucleoside-diphosphate reductase, beta subunit (324 aa)

NSE_0520	ribonucleoside-diphosphate reductase, beta subunit (322 aa)

RP512	RIBONUCLEOSIDE-DIPHOSPHATE REDUCTASE BETA CHAIN (nrdB) (324 aa)

WD0212	ribonucleoside-diphosphate reductase, beta subunit (329 aa)

Cluster 153

APH_1301	peptidyl-tRNA hydrolase (183 aa)

ECH_0141	peptidyl-tRNA hydrolase (198 aa)

NSE_0307	peptidyl-tRNA hydrolase (194 aa)

RP605	PEPTIDYL-TRNA HYDROLASE (pth) (185 aa)

WD0175	peptidyl-tRNA hydrolase (182 aa)

Cluster 154

APH_1348	DNA polymerase III, subunits gamma and tau (456 aa)

ECH_0014	DNA polymerase III, subunits gamma and tau (505 aa)

NSE_0492	DNA polymerase III, gamma/tau subunit, putative (396 aa)

RP865	DNA POLYMERASE III SUBUNITS GAMMA AND TAU (541 aa)

WD1060	DNA polymerase III, gamma and tau subunits (533 aa)

Cluster 155

APH_0622	dimethyladenosine transferase (269 aa)

ECH_0648	dimethyladenosine transferase (277 aa)

NSE_0362	dimethyladenosine transferase (262 aa)

RP672	DIMETHYLADENOSINE TRANSFERASE (ksgA) (268 aa)

WD0090	dimethyladenosine transferase (286 aa)

Cluster 156

APH_1290	conserved hypothetical protein TIGR00043 (154 aa)

ECH_1155	conserved hypothetical protein TIGR00043 (154 aa)

NSE_0249	conserved hypothetical protein TIGR00043 (143 aa)

RP741	unknown (180 aa)

WD0726	conserved hypothetical protein TIGR00043 (150 aa)

Cluster 157

APH_0965	methionyl-tRNA formyltransferase (301 aa)

ECH_0897	methionyl-tRNA formyltransferase (303 aa)

NSE_0094	methionyl-tRNA formyltransferase (307 aa)

RP209	METHIONYL-TRNA FORMYLTRANSFERASE (fmt) (303 aa)

WD0866	methionyl-tRNA formyltransferase (299 aa)

Cluster 158

APH_0093	thymidylate kinase (219 aa)

ECH_0229	thymidylate kinase (202 aa)

NSE_0547	thymidylate kinase (199 aa)

RP684	THYMIDYLATE KINASE (tmk) (203 aa)

WD1251	thymidylate kinase (212 aa)

Cluster 159

APH_1079	methionyl-tRNA synthetase (513 aa)

ECH_1000	methionyl-tRNA synthetase (506 aa)

NSE_0893	methionyl-tRNA synthetase (503 aa)

RP683	METHIONYL-TRNA SYNTHETASE (metS) (508 aa)

WD0352	methionyl-tRNA synthetase (545 aa)

Cluster 160

APH_0390	GTP-binding protein EngA, putative (440 aa)

ECH_0504	GTP-binding protein EngA (442 aa)

NSE_0536	GTP-binding protein EngA, putative (473 aa)

RP677	unknown (447 aa)

WD1098	GTP-binding protein (441 aa)

Cluster 161

APH_0151	ribosomal protein S6 (109 aa)

ECH_0308	ribosomal protein S6 (109 aa)

NSE_0062	ribosomal protein S6 (114 aa)

RP039	30S RIBOSOMAL PROTEIN S6 (rpsF) (121 aa)

WD0781	ribosomal protein S6 family protein (120 aa)

Cluster 162

APH_0120	histidyl-tRNA synthetase (420 aa)

ECH_0291	histidyl-tRNA synthetase (413 aa)

NSE_0781	histidyl-tRNA synthetase (413 aa)

RP308	HISTIDYL-TRNA SYNTHETASE (hisS) (416 aa)

WD1076	histidyl-tRNA synthetase (408 aa)

Cluster 163

APH_0398	translation elongation factor Ts (287 aa)

ECH_0515	translation elongation factor Ts (288 aa)

NSE_0947	translation elongation factor Ts (290 aa)

RP087	ELONGATION FACTOR TS (tsf) (309 aa)

WD0531	translation elongation factor Ts (286 aa)

Cluster 164

APH_0331	ribonucleoside-diphosphate reductase, alpha subunit (603 aa)

ECH_0459	ribonucleoside-diphosphate reductase, alpha subunit (595 aa)

NSE_0411	ribonucleoside-diphosphate reductase, alpha subunit (605 aa)

RP513	RIBONUCLEOSIDE-DIPHOSPHATE REDUCTASE ALPHA CHAIN (nrdA) (607 aa)

WD0197	ribonucleoside-diphosphate reductase, alpha subunit (594 aa)

Cluster 165

APH_0153	ribosomal protein L9 (205 aa)

ECH_0310	ribosomal protein L9 (208 aa)

NSE_0060	ribosomal protein L9 (160 aa)

RP041	50S RIBOSOMAL PROTEIN L9 (rplI) (171 aa)

WD0783	ribosomal protein L9 (184 aa)

Cluster 166

APH_1160	signal peptidase II (156 aa)

ECH_1060	signal peptidase II (146 aa)

NSE_0915	signal peptidase II (184 aa)

RP408	LIPOPROTEIN SIGNAL PEPTIDASE (lspA) (194 aa)

WD0760	signal peptidase II (158 aa)

Cluster 167

APH_0300	adenylate kinase (216 aa)

ECH_0429	adenylate kinase (221 aa)

NSE_0286	adenylate kinase (209 aa)

RP638	ADENYLATE KINASE (adk) (213 aa)

WD0661	adenylate kinase (213 aa)

Cluster 168

APH_0431	tRNA delta(2)-isopentenylpyrophosphate transferase (312 aa)

ECH_0588	tRNA delta(2)-isopentenylpyrophosphate transferase (303 aa)

NSE_0570	tRNA delta(2)-isopentenylpyrophosphate transferase (305 aa)

RP510	TRNA DELTA(2)-ISOPENTENYLPYROPHOSPHATE TRANSFERASE (miaA) (311 aa)

WD0822	tRNA delta(2)-isopentenylpyrophosphate transferase (297 aa)

Cluster 169

APH_0332	conserved hypothetical protein TIGR00250 (166 aa)

ECH_0460	conserved hypothetical protein TIGR00250 (156 aa)

NSE_0006	conserved hypothetical protein (159 aa)

RP330	unknown (156 aa)

WD0143	conserved hypothetical protein TIGR00250 (155 aa)

Cluster 170

APH_0571	DNA primase (607 aa)

ECH_0761	DNA primase (588 aa)

NSE_0961	DNA primase (579 aa)

RP859	DNA PRIMASE (dnaG) (616 aa)

WD0348	DNA primase (582 aa)

Cluster 171

APH_0305	phenylalanyl-tRNA synthetase, beta subunit (792 aa)

ECH_0434	phenylalanyl-tRNA synthetase, beta subunit (788 aa)

NSE_0426	phenylalanyl-tRNA synthetase, beta subunit, putative (876 aa)

RP418	PHENYLALANYL-TRNA SYNTHETASE BETA CHAIN (pheT) (815 aa)

WD0425	phenylalanyl-tRNA synthetase, beta subunit (779 aa)

Cluster 172

APH_0447	ribosome-binding factor A (118 aa)

ECH_0564	ribosome-binding factor A (114 aa)

NSE_0523	ribosome-binding factor A, putative (97 aa)

RP435	RIBOSOME-BINDING FACTOR A (rbfA) (128 aa)

WD1317	ribosomal binding factor A (132 aa)

Cluster 173

APH_1026	ribosomal protein L10 (160 aa)

ECH_0954	ribosomal protein L10 (160 aa)

NSE_0680	50S ribosomal protein L10 (174 aa)

RP138	50S RIBOSOMAL PROTEIN L10 (rplJ) (169 aa)

WD0022	ribosomal protein L10 (172 aa)

Cluster 174

APH_0660	aspartate aminotransferase (394 aa)

ECH_0732	aspartate aminotransferase (398 aa)

NSE_0758	aspartate aminotransferase (397 aa)

RP091	ASPARTATE AMINOTRANSFERASE A (aatA) (412 aa)

WD1029	aspartate aminotransferase (397 aa)

Cluster 175

APH_0104	uridylate kinase, truncation (97 aa)

APH_0111	uridylate kinase (244 aa)

ECH_0266	uridylate kinase (244 aa)

NSE_0946	uridylate kinase (233 aa)

RP155	URIDYLATE KINASE (pyrH) (242 aa)

WD0530	uridylate kinase (247 aa)

Cluster 176

APH_0314	queuine tRNA-ribosyltransferase (394 aa)

ECH_0445	queuine tRNA-ribosyltransferase (396 aa)

NSE_0016	queuine tRNA-ribosyltransferase (402 aa)

RP721	QUEUINE TRNA-RIBOSYLTRANSFERASE (tgt) (361 aa)

WD0735	queuine tRNA-ribosyltransferase (404 aa)

Cluster 177

APH_1267	tRNA (guanine-N1)-methyltransferase (232 aa)

ECH_0003	tRNA (guanine-N1)-methyltransferase (235 aa)

NSE_0896	tRNA (guanine-N1)-methyltransferase (225 aa)

RP111	TRNA (GUANINE-N1)-METHYLTRANSFERASE (trmD) (234 aa)

WD1022	tRNA (guanine-N1)-methyltransferase (236 aa)

Cluster 178

APH_0308	ribonuclease P protein component (120 aa)

ECH_0439	hypothetical protein (119 aa)

NSE_0857	ribonuclease P protein component (113 aa)

RP611	RIBONUCLEASE P (rnpA) (121 aa)

WD0200	hypothetical protein (45 aa)

Cluster 179

APH_1302	ribosomal 5S rRNA E-loop binding protein Ctc/L25/TL5 (211 aa)

ECH_0142	ribosomal 5S rRNA E-loop binding protein Ctc/L25/TL5 (210 aa)

NSE_0306	ribosomal 5S rRNA E-loop binding protein Ctc/L25/TL5 (194 aa)

RP606	PROBABLE 50S RIBOSOMAL PROTEIN L25 (rplY) (203 aa)

WD0174	ribosomal 5S rRNA E-loop binding protein Ctc/L25/TL5 (203 aa)

Cluster 180

APH_0157	ABC transporter, ATP-binding protein/permease protein, putative (578 aa)

ECH_0313	ABC transporter, permease/ATP-binding protein (580 aa)

NSE_0865	ABC transporter, ATP-binding protein/permease protein, putative (587 aa)

NSE_0410	ABC transporter, ATP-binding/permease protein, putative (557 aa)

RP205	MITOCHONDRIAL TRANSPORTER ATM1 PRECURSOR (atm1) (609 aa)

RP214	ABC TRANSPORTER ATP-BINDING PROTEIN (abcT3) (548 aa)

RP387	PROBABLE TRANSPORT ATP-BINDING PROTEIN MSBA (msbA1) (476 aa)

RP696	PROBABLE TRANSPORT ATP-BINDING PROTEIN MSBA (msbA2) (576 aa)

WD0400	ABC transporter, HlyB/MsbA family, putative (568 aa)

WD0616	ABC transporter, permease/ATP-binding protein, putative (591 aa)

Cluster 181

APH_1346	aspartate-semialdehyde dehydrogenase, putative (337 aa)

ECH_0016	aspartate-semialdehyde dehydrogenase (336 aa)

ECH_1017	N-acetyl-gamma-glutamyl-phosphate reductase (346 aa)

NSE_0260	aspartate-semialdehyde dehydrogenase (334 aa)

RP316	ASPARTATE-SEMIALDEHYDE DEHYDROGENASE (asd) (338 aa)

WD0954	aspartate-semialdehyde dehydrogenase (344 aa)

Cluster 182

APH_0075	methyltransferase, putative (182 aa)

ECH_0211	methyltransferase, putative (183 aa)

NSE_0201	methyltransferase, putative (198 aa)

RP545	unknown (236 aa)

WD0852	methyltransferase, putative (182 aa)

Cluster 183

APH_0676	iron-sulfur cluster assembly accessory protein (121 aa)

APH_0803	iron-sulfur cluster assembly accessory protein (148 aa)

ECH_0631	iron-sulfur cluster assembly accessory protein (147 aa)

ECH_0689	iron-sulfur cluster assembly accessory protein (150 aa)

NSE_0458	iron-sulfur cluster assembly accessory protein, putative (178 aa)

NSE_0301	iron-sulfur cluster assembly accessory protein (121 aa)

RP063	HESB PROTEIN (hesB1) (110 aa)

RP484	HESB PROTEIN (hesB2) (110 aa)

WD0708	HesB/YadR/YfhF family protein (153 aa)

WD0890	HesB/YadR/YfhF family protein (137 aa)

Cluster 184

APH_1189	ATP synthase F0, B subunit, putative (163 aa)

APH_1190	ATP synthase F0, B' subunit, putative (167 aa)

ECH_1088	ATP synthase F0, B' subunit (168 aa)

ECH_1089	ATP synthase F0, B chain (161 aa)

NSE_0398	ATP synthase F0, B' chain (176 aa)

RP020	ATP SYNTHASE B CHAIN PRECURSOR (atpF) (167 aa)

RP021	ATP SYNTHASE B CHAIN (atpX) (158 aa)

WD0429	ATP synthase F0, B subunit, putative (159 aa)

WD0430	conserved domain protein (158 aa)

Cluster 185

APH_0747	folylpolyglutamate synthase (429 aa)

ECH_0702	folylpolyglutamate synthase (435 aa)

NSE_0715	folylpolyglutamate synthase (442 aa)

RP536	FOLYLPOLYGLUTAMATE SYNTHASE (folC) (433 aa)

WD1052	folylpolyglutamate synthase (429 aa)

Cluster 186

APH_1292	cell division protein FtsZ (400 aa)

APH_0129	tubulin/FtsZ family, C-terminal domain (225 aa)

ECH_1153	cell division protein FtsZ (421 aa)

NSE_0342	cell division protein FtsZ (372 aa)

RP666	CELL DIVISION PROTEIN FTSZ (ftsZ) (452 aa)

WD0723	cell division protein FtsZ (398 aa)

Cluster 187

APH_0707	ATP synthase F1, gamma subunit (285 aa)

APH_0813	ATP synthase F1, gamma subunit, truncated (183 aa)

ECH_0652	ATP synthase F1, gamma subunit (281 aa)

NSE_0588	ATP synthase F1, gamma subunit (269 aa)

RP802	ATP SYNTHASE GAMMA CHAIN (atpG) (288 aa)

WD1233	ATP synthase F1, gamma subunit (290 aa)

Cluster 188

APH_1334	ATP synthase F1, alpha subunit (506 aa)

ECH_0132	ATP synthase F1, alpha subunit (509 aa)

NSE_0131	ATP synthase F1, alpha subunit (509 aa)

RP803	ATP SYNTHASE ALPHA CHAIN (atpA) (512 aa)

WD0655	ATP synthase F1, alpha subunit (513 aa)

Cluster 189

APH_1191	ATP synthase F0, C chain (74 aa)

ECH_1087	ATP synthase F0, C chain (73 aa)

NSE_0397	ATP synthase F0, C chain (75 aa)

RP022	ATP SYNTHASE C CHAIN (atpE) (74 aa)

WD0428	ATP synthase F0, C subunit (75 aa)

Cluster 190

APH_1192	ATP synthase F0, A subunit (242 aa)

ECH_1086	ATP synthase F0, A subunit (243 aa)

NSE_0396	ATP synthase F0, A subunit (243 aa)

RP023	ATP SYNTHASE A CHAIN (atpB) (242 aa)

WD0427	ATP synthase F0, A subunit (241 aa)

Cluster 191

APH_0089	ATP-NAD kinase (270 aa)

ECH_0225	ATP-NAD kinase, putative (263 aa)

NSE_0310	ATP-NAD kinase, putative (253 aa)

RP440	unknown (255 aa)

WD0871	ATP-NAD kinase (264 aa)

Cluster 192

APH_0493	ATP synthase F1, epsilon subunit (128 aa)

ECH_0574	ATP synthase F1, epsilon subunit (128 aa)

NSE_0762	ATP synthase F1, epsilon subunit (111 aa)

RP800	ATP SYNTHASE EPSILON CHAIN (atpC) (112 aa)

WD0204	ATP synthase F1, epsilon subunit, putative (114 aa)

Cluster 193

APH_1270	aminomethyl transferase family protein (275 aa)

ECH_0138	aminomethyl transferase family protein (278 aa)

NSE_0318	aminomethyl transferase family protein (310 aa)

RP464	unknown (285 aa)

WD0309	aminomethyl transferase family protein (268 aa)

Cluster 194

APH_1268	16S rRNA processing protein RimM (171 aa)

ECH_0002	16S rRNA processing protein RimM (175 aa)

NSE_0124	16S rRNA processing protein RimM, putative (163 aa)

RP348	unknown (165 aa)

WD1282	16S rRNA processing protein RimM (168 aa)

Cluster 195

APH_0308.1	ribosomal protein L34 (44 aa)

ECH_0440	ribosomal protein L34 (44 aa)

NSE_0856	ribosomal protein L34 (44 aa)

RP610	50S RIBOSOMAL PROTEIN L34 (rpmH) (44 aa)

WD0201	ribosomal protein L34 (44 aa)

Cluster 196

APH_0746	porphobilinogen deaminase (304 aa)

ECH_0701	porphobilinogen deaminase (298 aa)

NSE_0385	porphobilinogen deaminase, putative (286 aa)

RP466	PORPHOBILINOGEN DEAMINASE (hemC) (299 aa)

WD0542	porphobilinogen deaminase (292 aa)

Cluster 197

APH_0415	propionyl-CoA carboxylase, beta subunit (509 aa)

ECH_0599	propionyl-CoA carboxylase, beta subunit (510 aa)

NSE_0796	propionyl-CoA carboxylase, beta subunit (518 aa)

RP619	PROPIONYL-COA CARBOXYLASE BETA CHAIN PRECURSOR (pccB) (514 aa)

WD1157	propionyl-CoA carboxylase, beta subunit (508 aa)

Cluster 198

APH_0955	FtsK/SpoIIIE family protein (835 aa)

ECH_0890	cell division protein FtsK, putative (827 aa)

NSE_0650	cell division protein FtsK, putative (809 aa)

RP823	CELL DIVISION PROTEIN FTSK HOMOLOG (ftsK) (744 aa)

WD0120	cell division protein FtsK, putative (704 aa)

Cluster 199

APH_0275	RNA methyltransferase, TrmH family, group 3 (258 aa)

ECH_0404	RNA methyltransferase, TrmH family, group 3 (252 aa)

NSE_0262	RNA methyltransferase, TrmH family, group 3 (243 aa)

RP664	unknown (248 aa)

WD0951	RNA methyltransferase, TrmH family, group 3 (254 aa)

Cluster 200

APH_0856	biotin--acetyl-CoA-carboxylase ligase, putative, degenerate (150 aa)

APH_0876	biotin--acetyl-CoA-carboxylase ligase (241 aa)

ECH_0848	biotin--acetyl-CoA-carboxylase ligase (251 aa)

NSE_0693	biotin--acetyl-CoA-carboxylase ligase (226 aa)

RP533	BIOTIN-PROTEIN LIGASE (birA) (268 aa)

WD0970	biotin protein ligase, putative (347 aa)

Cluster 201

APH_1217	nucleoside diphosphate kinase (140 aa)

ECH_1117	nucleoside diphosphate kinase (142 aa)

NSE_0560	nucleoside diphosphate kinase (139 aa)

RP055	NUCLEOSIDE DIPHOSPHATE KINASE (ndk) (140 aa)

WD1183	nucleoside diphosphate kinase (139 aa)

Cluster 202

APH_1206	CDP-diacylglycerol--glycerol-3-phosphate 3-phosphatidyltransferase (181 aa)

ECH_1078	CDP-diacylglycerol--glycerol-3-phosphate 3-phosphatidyltransferase (175 aa)

NSE_0009	CDP-diacylglycerol--glycerol-3-phosphate 3-phosphatidyltransferase (182 aa)

RP049	CDP-DIACYLGLYCEROL--GLYCEROL-3-PHOSPHATE 3-PHOSPHATIDYLTRANSFERASE (pgsA) (181 aa)

WD0487	CDP-diacylglycerol--glycerol-3-phosphate 3-phosphatidyltransferase (177 aa)

Cluster 203

APH_0092	malonyl CoA-acyl carrier protein transacylase (319 aa)

ECH_0227	malonyl CoA-acyl carrier protein transacylase (323 aa)

NSE_0960	malonyl CoA-acyl carrier protein transacylase (309 aa)

RP735	MALONYL COA-ACYL CARRIER PROTEIN TRANSACYLASE (fabD) (319 aa)

WD0869	malonyl CoA-acyl carrier protein transacylase (326 aa)

Cluster 204

APH_0198	glycerol-3-phosphate dehydrogenase [NAD(P)+] (324 aa)

ECH_0340	glycerol-3-phosphate dehydrogenase [NAD(P)+] (326 aa)

NSE_0228	glycerol-3-phosphate dehydrogenase (NAD(P)+) (334 aa)

RP442	GLYCEROL-3-PHOSPHATE DEHYDROGENASE (gpsA) (316 aa)

WD0731	glycerol-3-phosphate dehydrogenase (NAD+) (327 aa)

Cluster 205

APH_0113	phosphatidate cytidylyltransferase, putative (235 aa)

ECH_0269	phosphatidate cytidylyltransferase, putative (211 aa)

NSE_0942	phosphatidate cytidylyltransferase, putative (205 aa)

RP424	PHOSPHATIDATE CYTIDYLYLTRANSFERASE (cdsA) (228 aa)

WD0526	phosphatidate cytidylyltransferase (208 aa)

Cluster 206

APH_1211	membrane-associated zinc metalloprotease, putative (362 aa)

ECH_1070	membrane-associated zinc metalloprotease, putative (380 aa)

NSE_0719	membrane-associated zinc metalloprotease, putative (366 aa)

RP161	unknown (359 aa)

WD1086	membrane-associated zinc metalloprotease, putative (372 aa)

Cluster 207

APH_1366	protein-export membrane protein SecD (508 aa)

ECH_1106	protein-export membrane protein SecD (505 aa)

NSE_0627	protein-export membrane protein SecD (502 aa)

RP586	PROTEIN-EXPORT MEMBRANE PROTEIN SECD (secD) (518 aa)

WD0086	protein-export membrane protein SecD (501 aa)

Cluster 208

APH_0235	ATP-dependent chaperone protein ClpB (859 aa)

ECH_0367	ATP-dependent Clp protease, ATP-binding subunit ClpB (857 aa)

NSE_0119	ClpB protein (854 aa)

RP036	CLPB PROTEIN (clpB) (858 aa)

WD0224	ATP-dependent Clp protease, ATP-binding subunit ClpB (853 aa)

Cluster 209

APH_0317	3-oxoacyl-(acyl-carrier-protein) synthase III (319 aa)

ECH_0448	3-oxoacyl-(acyl-carrier-protein) synthase III (319 aa)

NSE_0630	3-oxoacyl-(acyl-carrier-protein) synthase III (311 aa)

RP772	3-OXOACYL-[ACYL-CARRIER-PROTEIN] SYNTHASE III (fabH) (317 aa)

WD0985	3-oxoacyl-(acyl-carrier-protein) synthase III (318 aa)

Cluster 210

APH_0693	5-formyltetrahydrofolate cyclo-ligase family protein (174 aa)

ECH_0541	5-formyltetrahydrofolate cyclo-ligase family protein (173 aa)

NSE_0133	5-formyltetrahydrofolate cyclo-ligase family protein (193 aa)

RP461	unknown (172 aa)

WD1268	5-formyltetrahydrofolate cyclo-ligase, putative (183 aa)

Cluster 211

APH_0428	DNA recombination protein RmuC, putative (411 aa)

ECH_0577	RmuC family protein (397 aa)

NSE_0125	RmuC domain protein (408 aa)

RP690	unknown (426 aa)

WD0251	conserved hypothetical protein (443 aa)

Cluster 212

APH_1075	ubiquinone/menaquinone biosynthesis methlytransferase UbiE (231 aa)

ECH_0998	ubiquinone/menaquinone biosynthesis methlytransferase UbiE (230 aa)

NSE_0950	ubiquinone/menaquinone biosynthesis methlytransferase UbiE (230 aa)

RP680	UBIQUINONE/MENAQUINONE BIOSYNTHESIS METHLYTRANSFERASE UBIE (ubiE) (265 aa)

WD0393	ubiquinone/menaquinone biosynthesis methlytransferase UbiE (238 aa)

Cluster 213

APH_0995	membrane protein, putative (363 aa)

ECH_0910	conserved hypothetical protein (379 aa)

NSE_0191	permease, PerM family (327 aa)

RP630	PUTATIVE PERMEASE PERM HOMOLOG (perM) (351 aa)

WD1144	conserved hypothetical protein (354 aa)

Cluster 214

APH_0109	ribonuclease H1 (150 aa)

ECH_0263	ribonuclease HI (146 aa)

NSE_0667	ribonuclease HI (151 aa)

RP726	RIBONUCLEASE H (rnhA) (152 aa)

WD0304	ribonuclease H (145 aa)

Cluster 215

APH_0606	ubiquinone biosynthesis O-methyltransferase (236 aa)

APH_0642	ribose 5-phosphate isomerase B (143 aa)

ECH_0637	3-demethylubiquinone-9 3-methyltransferase (241 aa)

ECH_0638	ribose 5-phosphate isomerase B (148 aa)

NSE_0217	ribose 5-phosphate isomerase B (152 aa)

NSE_0628	3-demethylubiquinone-9 3-methyltransferase (230 aa)

RP299	GALACTOSE-6-PHOSPHATE ISOMERASE LACB SUBUNIT (lacA) (144 aa)

RP622	3-DEMETHYLUBIQUINONE-9 3-METHYLTRANSFERASE (ubiG) (252 aa)

WD0350	3-demethylubiquinone-9 3-methyltransferase/sugar-phosphate isomerase family protein (391 aa)

Cluster 216

APH_1246	glutamine synthetase, type I (472 aa)

APH_1061	glutamine synthetase domain protein (270 aa)

ECH_0089	glutamine synthetase, type I (470 aa)

ECH_0983	glutamine synthetase domain protein (265 aa)

NSE_0696	glutamine synthetase domain protein (272 aa)

NSE_0350	glutamine synthetase, type I (468 aa)

RP495	unknown (276 aa)

WD1322	glutamine synthetase (245 aa)

Cluster 217

APH_0471	exodeoxyribonuclease III, N-terminal truncation (185 aa)

APH_0505	exodeoxyribonuclease III (273 aa)

ECH_0675	exodeoxyribonuclease III (281 aa)

NSE_0415	exodeoxyribonuclease III (265 aa)

RP260	EXODEOXYRIBONUCLEASE III (xthA1) (261 aa)

RP676	EXODEOXYRIBONUCLEASE III (xthA2) (269 aa)

WD1001	exodeoxyribonuclease III (259 aa)

Cluster 218

APH_1299	NADH-ubiquinone oxidoreductase, putative (313 aa)

ECH_0063	NADH-ubiquinone oxidoreductase, homolog (320 aa)

NSE_0386	NADH-ubiquinone oxidoreductase family protein (340 aa)

RP333	CAPD PROTEIN (capD) (341 aa)

WD1129	NADH-ubiquinone oxidoreductase, putative (316 aa)

WD0612	NAD-dependent epimerase/dehydratase family protein (319 aa)

Cluster 219

APH_1326	protein-export membrane protein SecF (305 aa)

ECH_0095	protein-export membrane protein SecF (293 aa)

NSE_0417	protein-export membrane protein SecF, putative (298 aa)

RP114	PROTEIN-EXPORT MEMBRANE PROTEIN SECF (secF) (311 aa)

WD0235	protein-export membrane protein SecF (291 aa)

Cluster 220

APH_1281	integration host factor, alpha subunit (106 aa)

APH_0784	DNA-binding protein HU (91 aa)

ECH_0162	integration host factor, alpha subunit (100 aa)

ECH_0804	DNA-binding protein HU (94 aa)

NSE_0640	DNA-binding protein HU (93 aa)

RP171	DNA-BINDING PROTEIN HU (hupA) (110 aa)

RP708	INTEGRATION HOST FACTOR ALPHA-SUBUNIT (himA) (95 aa)

WD0057	DNA-binding protein, HU family (101 aa)

WD0065	DNA-binding protein, HU family (103 aa)

Cluster 221

APH_1298	ATP-dependent DNA helicase RecG (702 aa)

APH_0107	transcription-repair coupling factor (1139 aa)

ECH_0062	ATP-dependent DNA helicase RecG (679 aa)

ECH_0250	transcription-repair coupling factor (1134 aa)

NSE_0690	ATP-dependent DNA helicase RecG (675 aa)

RP593	ATP-DEPENDENT DNA HELICASE RECG (recG) (724 aa)

RP598	TRANSCRIPTION-REPAIR COUPLING FACTOR (mfd) (1120 aa)

WD0824	ATP-dependent DNA helicase RecG (673 aa)

Cluster 222

APH_0780	dephospho-CoA kinase (224 aa)

ECH_0799	hypothetical protein (46 aa)

ECH_0801	dephospho-CoA kinase (202 aa)

NSE_0309	dephospho-CoA kinase (200 aa)

RP731	unknown (191 aa)

WD0185	kinase, putative (195 aa)

Cluster 223

APH_0367	lipoic acid synthetase (295 aa)

ECH_0490	lipoic acid synthetase (297 aa)

NSE_0322	lipoic acid synthetase (296 aa)

RP742	LIPOIC ACID SYNTHETASE (lipA) (297 aa)

WD0392	lipoic acid synthetase (287 aa)

Cluster 224

APH_0130	deoxycytidine triphosphate deaminase, putative (185 aa)

ECH_0296	deoxycytidine triphosphate deaminase, putative (187 aa)

NSE_0964	deoxycytidine triphosphate deaminase, putative (185 aa)

RP069	DEOXYCYTIDINE TRIPHOSPHATE DEAMINASE (dcd) (188 aa)

WD0379	deoxycytidine triphosphate deaminase, putative (185 aa)

Cluster 225

APH_0971	trigger factor, putative (439 aa)

ECH_0902	trigger factor (439 aa)

NSE_0751	trigger factor, putative (433 aa)

RP842	TRIGGER FACTOR (tig) (445 aa)

WD0320	trigger factor, putative (444 aa)

Cluster 226

APH_0880	tRNA-i(6)A37 thiotransferase enzyme MiaB (444 aa)

ECH_0217	tRNA modification enzyme, MiaB family (412 aa)

ECH_0837	tRNA-i(6)A37 modification enzyme MiaB (442 aa)

NSE_0832	tRNA-i(6)A37 modification enzyme MiaB (471 aa)

NSE_0785	tRNA modification enzyme, MiaB family (429 aa)

RP416	unknown (421 aa)

RP808	unknown (445 aa)

WD0421	TRAM domain protein (439 aa)

WD0503	MiaB-like tRNA modifying enzyme (408 aa)

Cluster 227

APH_0597	DNA topoisomerase I (811 aa)

ECH_0746	topoisomerase DNA-binding C4 zinc finger domain protein (260 aa)

ECH_0750	DNA topoisomerase I (820 aa)

NSE_0594	DNA topoisomerase I (827 aa)

RP326	DNA TOPOISOMERASE I (topA) (776 aa)

WD1133	DNA topoisomerase I (816 aa)

Cluster 228

APH_0337	primosomal protein N truncation (191 aa)

APH_0356	primosomal protein N' (658 aa)

ECH_0483	primosomal protein N' (658 aa)

NSE_0206	primosomal protein N' (709 aa)

RP540	POSSIBLE PRIMOSOMAL PROTEIN N (priA) (648 aa)

WD1200	primosomal protein N (780 aa)

Cluster 229

APH_0196	nitrogen assimilation regulatory protein (462 aa)

APH_0551	response regulator/GGDEF domain protein (456 aa)

APH_1099	DNA-binding response regulator (276 aa)

ECH_0339	nitrogen regulation protein NtrX, putative (465 aa)

ECH_0773	response regulator/diguanylate cyclase, putative (458 aa)

ECH_1012	DNA-binding response regulator (265 aa)

NSE_0234	DNA-binding response regulator (265 aa)

NSE_0512	response regulator/GGDEF domain protein (456 aa)

NSE_0442	EAL domain protein (338 aa)

RP071	TRANSCRIPTIONAL ACTIVATOR PROTEIN CZCR (czcR) (237 aa)

RP237	unknown (450 aa)

RP246	unknown (407 aa)

RP427	PETR PROTEIN (ompR) (225 aa)

RP562	NITROGEN ASSIMILATION REGULATORY PROTEIN NTRX (ntrX) (475 aa)

WD0221	response regulator/GGDEF domain protein (460 aa)

WD0732	cell cycle transcriptional regulator (256 aa)

Cluster 230

APH_0474	chromosomal replication initiator protein DnaA (459 aa)

ECH_0808	chromosomal replication initiator protein DnaA, degenerate (59 aa)

ECH_0809	chromosomal replication initiator protein DnaA (464 aa)

NSE_0259	chromosomal replication initiator protein DnaA (474 aa)

RP601	CHROMOSOMAL REPLICATION INITIATOR PROTEIN DNAA (dnaA) (463 aa)

WD0001	chromosomal replication initiator protein DnaA (460 aa)

Cluster 231

APH_0243	lipoate-protein ligase B (208 aa)

ECH_0371	lipoate-protein ligase B (214 aa)

NSE_0100	lipoate-protein ligase B (209 aa)

RP876	PROBABLE LIPOATE-PROTEIN LIGASE B (lipB) (209 aa)

WD1014	lipoate-protein ligase B (205 aa)

Cluster 232

APH_0350	GTP-binding protein Era (305 aa)

ECH_0476	GTP-binding protein Era (296 aa)

NSE_0959	GTP-binding protein Era (308 aa)

RP118	GTP-BINDING PROTEIN ERA (era) (295 aa)

WD0743	GTP-binding protein Era (294 aa)

Cluster 233

APH_0794	conserved hypothetical protein TIGR00046 (239 aa)

ECH_0540	conserved hypothetical protein (241 aa)

NSE_0036	conserved hypothetical protein (227 aa)

RP168	unknown (198 aa)

WD1212	conserved hypothetical protein TIGR00046 (228 aa)

Cluster 234

APH_0451	GTP-binding protein TypA (612 aa)

ECH_0693	GTP-binding protein TypA (614 aa)

NSE_0673	GTP-binding protein TypA (596 aa)

RP263	unknown (605 aa)

WD0227	elongation factor Tu family protein (609 aa)

Cluster 235

APH_0039	preprotein translocase, SecG subunit (107 aa)

ECH_0172	preprotein translocase, SecG subunit (110 aa)

NSE_0876	preprotein translocase, SecG subunit (107 aa)

RP079	PROTEIN-EXPORT MEMBRANE PROTEIN SECG (secG) (100 aa)

WD0467	preprotein translocase, SecG subunit (106 aa)

Cluster 236

APH_1223	recA protein, truncation (84 aa)

APH_1354	recA protein (354 aa)

ECH_1109	recA protein (357 aa)

NSE_0530	recA protein (327 aa)

RP761	RECA PROTEIN (recA) (340 aa)

WD1050	recA protein (355 aa)

Cluster 237

APH_0018	crossover junction endodeoxyribonuclease RuvC (163 aa)

ECH_0028	crossover junction endodeoxyribonuclease RuvC (156 aa)

NSE_0955	crossover junction endodeoxyribonuclease RuvC (166 aa)

RP119	CROSSOVER JUNCTION ENDODEOXYRIBONUCLEASE RUVC (ruvC) (169 aa)

WD0142	crossover junction endodeoxyribonuclease RuvC (162 aa)

Cluster 238

APH_0166	holliday junction DNA helicase RuvB (329 aa)

ECH_0319	holliday junction DNA helicase RuvB (329 aa)

NSE_0575	holliday junction DNA helicase RuvB (331 aa)

RP386	HOLLIDAY JUNCTION DNA HELICASE RUVB (ruvB) (342 aa)

WD1112	Holliday junction DNA helicase RuvB (326 aa)

Cluster 239

APH_0167	Holliday junction DNA helicase RuvA (191 aa)

ECH_0320	holliday junction DNA helicase RuvA (190 aa)

NSE_0574	holliday junction DNA helicase RuvA (193 aa)

RP385	HOLLIDAY JUNCTION DNA HELICASE RUVA (ruvA) (199 aa)

WD1113	Holliday junction DNA helicase RuvA (190 aa)

Cluster 240

APH_0353	metallopeptidase, M24 family (569 aa)

ECH_0479	metallopeptidase, M24 family (574 aa)

NSE_0471	metallopeptidase, M24 family (545 aa)

RP482	unknown (591 aa)

WD1008	aminopeptidase P (555 aa)

Cluster 241

APH_0259	ankyrin repeat protein (239 aa)

APH_0375	type IV secretion system protein, VirB6 family (928 aa)

APH_0376	type IV secretion systemprotein, VirB6 family (1477 aa)

APH_0377	type IV secretion system VirB6 family protein (2360 aa)

APH_0546	conserved domain protein (1103 aa)

ECH_0166	conserved hypothetical protein (285 aa)

ECH_0247	conserved domain protein (302 aa)

ECH_0389	ankyrin repeat protein (140 aa)

ECH_0497	type IV secretion system protein, VirB6 family (922 aa)

ECH_0498	type IV secretion system protein,VirB6 family (1468 aa)

ECH_0499	type IV secretion system protein,VirB6 family (2758 aa)

NSE_0861	type IV secretion system protein, VirB6 family (839 aa)

NSE_0860	type IV secretion system protein, VirB6 family (1169 aa)

NSE_0859	type IV secretion system protein, VirB6 family (1149 aa)

NSE_0607	hypothetical protein (753 aa)

RP106	unknown (971 aa)

RP107	unknown (888 aa)

RP108	unknown (1155 aa)

RP226	unknown (584 aa)

RP451	CELL SURFACE ANTIGEN (sca3) (2340 aa)

RP704	CELL SURFACE ANTIGEN (sca5) (1643 aa)

WD0441	ankyrin repeat domain protein (402 aa)

WD0733	hypothetical protein (708 aa)

WD0854	membrane protein, putative (1242 aa)

WD0855	membrane protein, putative (992 aa)

WD0856	membrane protein, putative (795 aa)

Cluster 242

APH_0136	nitrogen regulation protein (700 aa)

APH_0582	sensor histidine kinase/response regulator (814 aa)

APH_0944	sensor histidine kinase (470 aa)

ECH_0299	nitrogen regulation protein NtrY, putative (714 aa)

ECH_0755	sensor histidine kinase/response regulator (417 aa)

ECH_0885	sensor histidine kinase (470 aa)

NSE_0003	sensor histidine kinase (447 aa)

NSE_0366	sensor histidine kinase/response regulator (742 aa)

RP229	HISTIDINE KINASE SENSOR PROTEIN (barA) (497 aa)

RP426	OSMOLARITY SENSOR PROTEIN ENVZ (envZ) (437 aa)

RP614	NITROGEN REGULATION PROTEIN NTRY (ntrY) (601 aa)

WD1216	sensor histidine kinase/response regulator (417 aa)

WD1284	sensor histidine kinase (475 aa)

Cluster 243

APH_0040	tyrosine recombinase XerD (311 aa)

APH_0200	tyrosine recombinase XerC (250 aa)

ECH_0173	tyrosine recombinase XerD (309 aa)

ECH_0341	site-specific recombinase, phage integrase family (311 aa)

NSE_0144	tyrosine recombinase XerD (305 aa)

NSE_0447	site-specific recombinase, phage integrase family (307 aa)

RP361	INTEGRASE/RECOMBINASE XERD (xerD) (335 aa)

RP817	PROBABLE INTEGRASE/RECOMBINASE RIPX (xerC) (305 aa)

WD1148	site-specific recombinase, phage integrase family (328 aa)

WD0752	tyrosine recombinase XerC (309 aa)

Cluster 244

APH_0462	glutamyl-tRNA FRAMESHIFT (54 aa)

APH_0470	glutamyl-tRNA(Gln) amidotransferase, B subunit (483 aa)

APH_0478	glutamyl-tRNA FRAMESHIFT (54 aa)

ECH_0813	glutamyl-tRNA(Gln) amidotransferase, B subunit (481 aa)

NSE_0084	glutamyl-tRNA(Gln) amidotransferase, B subunit (488 aa)

RP151	GLUTAMYL-tRNA AMIDOTRANSFERASE SUBUNIT B (gatB) (483 aa)

WD0146	glutamyl-tRNA(Gln) amidotransferase, B subunit (474 aa)

Cluster 245

APH_1403	type IV secretion system protein VirB11 (332 aa)

ECH_0041	type IV secretion system protein VirB11 (332 aa)

NSE_0740	type IV secretion system protein VirB11 (331 aa)

RP292	VIRB11 PROTEIN (virB11) (334 aa)

WD0007	type IV secretion system protein VirB11 (330 aa)

Cluster 246

APH_0748	glutamyl-tRNA(Gln) amidotransferase, A subunit (491 aa)

ECH_0703	glutamyl-tRNA(Gln) amidotransferase, A subunit (487 aa)

NSE_0256	glutamyl-tRNA(Gln) amidotransferase, A subunit (482 aa)

RP152	GLUTAMYL-tRNA AMIDOTRANSFERASE SUBUNIT A (gatA) (493 aa)

WD0505	glutamyl-tRNA(Gln) amidotransferase, A subunit (489 aa)

Cluster 247

APH_0082	pyruvate dehydrogenase complex, E1 component, pyruvate dehydrogenase alpha subunit (345 aa)

ECH_0220	pyruvate dehydrogenase complex, E1 component, pyruvate dehydrogenase alpha subunit (327 aa)

NSE_0802	pyruvate dehydrogenase complex, E1 component, pyruvate dehydrogenase alpha subunit (334 aa)

RP261	PYRUVATE DEHYDROGENASE E1 COMPONENT, ALPHA SUBUNIT PRECURSOR (326 aa)

WD0416	pyruvate dehydrogenase complex, E1 component, pyruvate dehydrogenase alpha subunit (326 aa)

Cluster 248

APH_0988	recombination protein RecR (194 aa)

ECH_0843	recombination protein RecR (195 aa)

NSE_0899	recombination protein RecR (196 aa)

RP438	RECOMBINATION PROTEIN RECR (recR) (226 aa)

WD1180	recombination protein RecR (201 aa)

Cluster 249

APH_0119	phosphoribosylaminoimidazole-succinocarboxamide synthase (244 aa)

ECH_0290	phosphoribosylaminoimidazole-succinocarboxamide synthase (242 aa)

NSE_0852	phosphoribosylaminoimidazole-succinocarboxamide synthase (240 aa)

RP220	PHOSPHORIBOSYLAMINOIMIDAZOLE-SUCCINOCARBOXAMIDE SYNTHASE (purC) (236 aa)

WD1023	phosphoribosylaminoimidazole-succinocarboxamide synthase (240 aa)

Cluster 250

APH_0026.1	ferredoxin (126 aa)

ECH_0038	ferredoxin A (125 aa)

NSE_0330	ferredoxin (139 aa)

RP829	FERREDOXIN (fdxA) (109 aa)

WD0093	ferredoxin, 4Fe-4S (124 aa)

Cluster 251

APH_0168	heme exporter protein CcmC (235 aa)

ECH_0321	heme exporter protein CcmC (234 aa)

NSE_0509	heme exporter protein, CcmC family (233 aa)

RP830	HEME EXPORTER PROTEIN C (ccmC) (230 aa)

WD0340	heme exporter protein CcmC (236 aa)

Cluster 252

APH_1409	DNA replication and repair protein RecF, putative (371 aa)

ECH_0076	DNA replication and repair protein RecF, putative (372 aa)

NSE_0199	DNA replication and repair protein RecF, putative (349 aa)

RP029	RECF PROTEIN (recF) (360 aa)

WD1286	recF protein (365 aa)

Cluster 253

APH_1257	pyruvate dehydrogenase complex, E2 component, dihydrolipoamide acetyltransferase, putative (420 aa)

ECH_0098	pyruvate dehydrogenase complex, E2 component, dihydrolipoamide acetyltransferase (416 aa)

NSE_0953	pyruvate dehydrogenase complex, E2 component, dihydrolipoamide acetyltransferase (403 aa)

RP530	DIHYDROLIPOAMIDE ACETYLTRANSFERASE COMPONENT (pdhC) (408 aa)

WD1177	pyruvate dehydrogenase complex, E2 component, dihydrolipoamide acetyltransferase (454 aa)

Cluster 254

APH_0496	magnesium transporter (448 aa)

ECH_0571	magnesium transporter (456 aa)

NSE_0465	magnesium transporter (437 aa)

RP583	MAGNESIUM TRANSPORTER (mgtE) (456 aa)

WD0375	magnesium transporter (456 aa)

Cluster 255

APH_0137	ribonuclease D, putative (381 aa)

APH_1098	3''-5'' exonuclease family protein (204 aa)

ECH_0300	ribonuclease D, putative (387 aa)

ECH_1011	3'-5' exonuclease family protein (204 aa)

NSE_0529	3'-5' exonuclease family protein (206 aa)

RP422	unknown (203 aa)

RP462	RIBONUCLEASE D (rnd) (281 aa)

WD0186	ribonuclease D, putative (392 aa)

WD0878	3'-5' exonuclease (206 aa)

Cluster 256

APH_0607	lysyl-tRNA synthetase (514 aa)

ECH_0626	lysyl-tRNA synthetase (511 aa)

NSE_0773	lysyl-tRNA synthetase (505 aa)

RP371	LYSYL-tRNA SYNTHETASE (lysS) (528 aa)

WD0860	lysyl-tRNA synthetase (511 aa)

Cluster 257

APH_0443	Sec-independent protein translocase TatC (241 aa)

ECH_0560	Sec-independent protein translocase protein TatC (249 aa)

NSE_0127	Sec-independent protein translocase TatC (263 aa)

RP782	unknown (251 aa)

WD0452	MttB family protein (253 aa)

Cluster 258

APH_1036	metallo-beta-lactamase family, beta-CASP subfamily (543 aa)

ECH_0966	metallo-beta-lactamase family, beta-CASP subfamily (540 aa)

NSE_0829	metallo-beta-lactamase family, beta-CASP subfamily (542 aa)

RP441	unknown (560 aa)

WD0039	metallo-beta-lactamase family protein (544 aa)

Cluster 259

APH_0851	delta-aminolevulinic acid dehydratase (330 aa)

ECH_0821	delta-aminolevulinic acid dehydratase (331 aa)

NSE_0394	porphobilinogen synthase (321 aa)

RP539	DELTA-AMINOLEVULINIC ACID DEHYDRATASE (hemB) (330 aa)

WD0158	delta-aminolevulinic acid dehydratase (331 aa)

Cluster 260

APH_0020	uroporphyrinogen decarboxylase (343 aa)

ECH_0030	uroporphyrinogen decarboxylase (334 aa)

NSE_0968	uroporphyrinogen decarboxylase (324 aa)

RP885	UROPORPHYRINOGEN DECARBOXYLASE (hemE) (345 aa)

WD1028	uroporphyrinogen decarboxylase (338 aa)

Cluster 261

APH_0263	ferrochelatase (320 aa)

ECH_0395	ferrochelatase (343 aa)

NSE_0790	ferrochelatase (325 aa)

RP884	PROBABLE FERROCHELATASE (hemH) (346 aa)

WD1186	ferrochelatase (315 aa)

Cluster 262

APH_0545	conserved hypothetical protein (1123 aa)

ECH_0778	conserved hypothetical protein (1132 aa)

NSE_0661	conserved hypothetical protein (1243 aa)

RP006	unknown (706 aa)

WD1082	conserved hypothetical protein (960 aa)

Cluster 263

APH_0402	ubiquinol-cytochrome c reductase, cytochrome b (408 aa)

ECH_0521	ubiquinol-cytochrome c reductase, cytochrome b (408 aa)

NSE_0625	ubiquinol-cytochrome c reductase, cytochrome b (410 aa)

RP271	CYTOCHROME B (petB) (398 aa)

WD1071	ubiquinol-cytochrome c reductase, cytochrome b (409 aa)

Cluster 264

APH_0160	conserved domain protein (52 aa)

APH_0163	thymidylate synthase, flavin-dependent (288 aa)

ECH_0317	thymidylate synthase, flavin-dependent (253 aa)

NSE_0163	thymidylate synthase, flavin-dependent (292 aa)

RP301	THY1 PROTEIN (thyA) (294 aa)

WD1198	thymidylate synthase, flavin-dependent (284 aa)

Cluster 265

APH_1084	cytochrome c oxidase, subunit II (262 aa)

ECH_1002	cytochrome c oxidase, subunit II (257 aa)

NSE_0621	cytochrome c oxidase, subunit II (255 aa)

RP406	CYTOCHROME C OXIDASE POLYPEPTIDE II (coxB) (313 aa)

WD0300	cytochrome c oxidase, subunit II (254 aa)

Cluster 266

APH_1085	cytochrome c oxidase, subunit I (518 aa)

ECH_1003	cytochrome c oxidase, subunit I (518 aa)

NSE_0622	cytochrome c oxidase, subunit I (519 aa)

RP405	CYTOCHROME C OXIDASE POLYPEPTIDE I (coxA) (534 aa)

WD0301	cytochrome c oxidase, subunit I (516 aa)

Cluster 267

APH_0019	cytochrome c oxidase, subunit III (285 aa)

ECH_0029	cytochrome c oxidase, subunit III (274 aa)

NSE_0676	cytochrome c oxidase, subunit III (274 aa)

RP191	CYTOCHROME C OXIDASE POLYPEPTIDE III (coxC) (278 aa)

WD0141	cytochrome c oxidase, subunit III (275 aa)

Cluster 268

APH_1171	conserved hypothetical protein (225 aa)

ECH_1081	SURF1 family protein (210 aa)

NSE_0066	lipoprotein, putative (222 aa)

RP733	SURFEIT LOCUS PROTEIN 1 (surf1) (244 aa)

WD1248	conserved hypothetical protein (205 aa)

Cluster 269

APH_0067	NifU domain protein (188 aa)

APH_0675	FeS cluster assembly scaffold IscU (130 aa)

ECH_0202	NifU domain protein (186 aa)

ECH_0630	FeS cluster assembly scaffold IscU (137 aa)

NSE_0817	NifU-like domain protein (180 aa)

NSE_0302	FeS cluster assembly scaffold IscU (142 aa)

RP485	NIFU PROTEIN (nifU) (131 aa)

RP667	unknown (190 aa)

WD1075	NifU domain protein (191 aa)

WD0889	NifU domain protein (127 aa)

Cluster 270

APH_0548	inositol monophosphatase, putative (256 aa)

ECH_0776	inositol monophosphatase, putative (244 aa)

NSE_0800	inositol monophosphatase family protein (239 aa)

RP239	EXTRAGENIC SUPPRESSOR PROTEIN SUHB (suhB) (245 aa)

RP297	CYSQ PROTEIN (cysQ) (262 aa)

WD1280	inositol monophosphatase family protein (231 aa)

Cluster 271

APH_0247	fumarate hydratase, class II (462 aa)

ECH_0376	fumarate hydratase, class II (461 aa)

ECH_0937	argininosuccinate lyase (470 aa)

NSE_0056	fumarate hydratase, class II (515 aa)

RP665	FUMARATE HYDRATASE (fumC) (461 aa)

WD0492	fumarate hydratase, class II (464 aa)

Cluster 272

APH_1341	lipoprotein releasing system, transmembrane protein, LolC/E family (399 aa)

ECH_0127	lipoprotein releasing system transmembrane protein, LolC/E family (410 aa)

NSE_0137	permease, putative (407 aa)

RP699	unknown (415 aa)

WD1229	conserved hypothetical protein (409 aa)

Cluster 273

APH_0224	pyridine nucleotide-disulfide oxidoreductase (628 aa)

APH_0412	glucose-inhibited division domain protein (249 aa)

ECH_0359	glucose inhibited division protein A (625 aa)

NSE_0707	glucose inhibited division protein A (627 aa)

RP056	GLUCOSE INHIBITED DIVISION PROTEIN A (gidA) (621 aa)

WD1009	glucose-inhibited division protein A (644 aa)

Cluster 274

APH_0968	ATP-dependent protease La (802 aa)

ECH_0899	ATP-dependent protease La (802 aa)

NSE_0347	ATP-dependent protease La (826 aa)

RP450	ATP-DEPENDENT PROTEASE LA (lon) (784 aa)

WD0317	ATP-dependent protease La (817 aa)

Cluster 275

APH_1051	ribosomal protein S21 (91 aa)

ECH_0978	ribosomal protein S21 (90 aa)

NSE_0827	ribosomal protein S21 (64 aa)

RP615	RIBOSOMAL PROTEIN S21 (rpsU) (66 aa)

WD1211	ribosomal protein S21, putative (66 aa)

Cluster 276

APH_0315	ribosomal protein L32 (58 aa)

ECH_0446	ribosomal protein L32 (61 aa)

NSE_0633	ribosomal protein L32 (63 aa)

RP773	50S RIBOSOMAL PROTEIN L32 (rpmF) (65 aa)

WD0987	ribosomal protein L32 (60 aa)

Cluster 277

APH_1090	preprotein translocase, YajC subunit (124 aa)

ECH_1008	preprotein translocase, YajC subunit (125 aa)

NSE_0319	preprotein translocase, YajC subunit (100 aa)

RP585	unknown (142 aa)

WD0534	preprotein translocase, YajC subunit (123 aa)

Cluster 278

APH_1288	tRNA modification GTPase TrmE (445 aa)

ECH_0060	tRNA modification GTPase TrmE (439 aa)

NSE_0717	tRNA modification GTPase TrmE (550 aa)

RP759	POSSIBLE THIOPHENE AND FURAN OXIDATION PROTEIN THDF (445 aa)

WD0981	tRNA modification GTPase TrmE (508 aa)

Cluster 279

APH_0338	OmpA family protein (205 aa)

ECH_0462	OmpA family protein (175 aa)

NSE_0889	peptidoglycan-associated lipoprotein, putative (208 aa)

RP771	PEPTIDOGLYCAN-ASSOCIATED LIPOPROTEIN PRECURSOR (pal) (155 aa)

WD1255	peptidoglycan-associated lipoprotein, putative (159 aa)

Cluster 280

APH_0538	N utilization substance protein B (282 aa)

ECH_0784	N utilization substance protein B, putative (174 aa)

NSE_0880	N utilization substance protein B, putative (141 aa)

RP162	N UTILIZATION SUBSTANCE PROTEIN B (nusB) (174 aa)

WD0239	N utilization substance protein B, putative (160 aa)

Cluster 281

APH_1210	outer membrane protein, OMP85 family (772 aa)

ECH_1071	outer membrane protein, OMP85 family (773 aa)

NSE_0718	outer membrane protein, OMP85 family (744 aa)

RP160	OUTER MEMBRANE PROTEIN OMP1 (omp1) (768 aa)

WD1085	surface antigen (778 aa)

Cluster 282

APH_0185	pyruvate, phosphate dikinase (874 aa)

ECH_0330	pyruvate, phosphate dikinase (873 aa)

NSE_0369	pyruvate, phosphate dikinase (867 aa)

RP492	PYRUVATE,PHOSPHATE DIKINASE PRECURSOR (ppdK) (880 aa)

WD0690	pyruvate phosphate dikinase (912 aa)

Cluster 283

APH_1398	dnaK suppressor protein, putative (144 aa)

ECH_0064	DnaK suppressor protein (152 aa)

NSE_0162	DnaK suppressor protein (137 aa)

RP816	DNAK SUPPRESSOR PROTEIN HOMOLOG (dksA) (143 aa)

WD1094	dnaK suppressor protein, putative (130 aa)

Cluster 284

APH_0896	hypothetical protein (96 aa)

APH_0897	endonuclease III (209 aa)

ECH_0856	endonuclease III, degenerate (45 aa)

ECH_0857	endonuclease III (210 aa)

NSE_0246	endonuclease III (216 aa)

RP746	ENDONUCLEASE III (nth) (212 aa)

WD0789	endonuclease III (212 aa)

Cluster 285

APH_0863	2-oxoglutarate dehydrogenase, E1 component (905 aa)

ECH_0832	2-oxoglutarate dehydrogenase, E1 component (912 aa)

NSE_0578	2-oxoglutarate dehydrogenase, E1 component (905 aa)

RP180	2-OXOGLUTARATE DEHYDROGENASE E1 COMPONENT (sucA) (936 aa)

WD1309	2-oxoglutarate dehydrogenase, E1 component (884 aa)

Cluster 286

APH_1146	HflK protein (368 aa)

APH_1147	HflC protein (291 aa)

ECH_1050	hflK protein (357 aa)

ECH_1051	hflC protein (289 aa)

NSE_0165	HflC protein (286 aa)

NSE_0164	HflK protein (347 aa)

RP122	HFLK PROTEIN (hflK) (344 aa)

RP123	HFLC PROTEIN (hflC) (286 aa)

RP328	unknown (311 aa)

WD0831	hflK protein (344 aa)

WD0832	hflC protein (290 aa)

Cluster 287

APH_0140	BolA family protein (78 aa)

APH_1004	BolA protein (90 aa)

ECH_0303	BolA family protein (73 aa)

ECH_0930	bolA protein, putative (96 aa)

NSE_0202	BolA family protein (86 aa)

NSE_0782	BolA family protein (95 aa)

RP245	BOLA PROTEIN HOMOLOG (bolA) (80 aa)

RP812	unknown (77 aa)

WD0490	conserved hypothetical protein (72 aa)

WD0926	bolA protein, putative (94 aa)

Cluster 288

APH_0139	glutaredoxin-related protein (109 aa)

ECH_0302	glutaredoxin-related protein (110 aa)

NSE_0203	glutaredoxin-related protein (106 aa)

RP745	GLUTAREDOXIN-LIKE PROTEIN GRLA (grxC2) (107 aa)

WD0491	glutaredoxin-related protein (107 aa)

Cluster 289

APH_0759	RNA polymerase sigma-32 factor (289 aa)

ECH_0655	RNA polymerase sigma-32 factor (297 aa)

NSE_0341	RNA polymerase sigma-32 factor (288 aa)

RP303	RNA POLYMERASE SIGMA-32 FACTOR (rpoH) (293 aa)

WD1064	heat shock sigma factor RpoH (297 aa)

Cluster 290

APH_0783	conserved domain protein (292 aa)

ECH_0803	conserved hypothetical protein (295 aa)

NSE_0377	hypothetical protein (266 aa)

RP172	DNA POLYMERASE III, DELTA SUBUNIT (holB) (269 aa)

WD0819	DNA polymerase III, subunit, putative (273 aa)

Cluster 291

APH_0014	glycyl-tRNA synthetase, beta subunit (701 aa)

ECH_0024	glycyl-tRNA synthetase, beta subunit (703 aa)

NSE_0756	glycyl-tRNA synthetase, beta subunit (696 aa)

RP849	GLYCYL-TRNA SYNTHETASE BETA CHAIN (glyS) (648 aa)

WD0155	glycyl-tRNA synthetase, beta subunit (705 aa)

Cluster 292

APH_1087	competence lipoprotein ComL, putative (233 aa)

ECH_1005	competence protein ComL, putative (250 aa)

NSE_0923	competence protein ComL, putative (219 aa)

RP183	unknown (251 aa)

WD0485	competence lipoprotein ComL, putative (235 aa)

Cluster 293

APH_1335	ATP synthase F1, delta subunit (180 aa)

ECH_0131	ATP synthase F1, delta subunit (185 aa)

NSE_0132	ATP synthase F1, delta subunit (174 aa)

RP804	ATP SYNTHASE DELTA CHAIN (atpH) (183 aa)

WD0656	ATP synthase F1, delta subunit (186 aa)

Cluster 294

APH_0044	NADH-ubiquinone/plastoquinone oxidoreductase family protein (495 aa)

APH_0181	NADH-ubiquinone/plastoquinone oxidoreductase family protein (500 aa)

APH_0439	NADH dehydrogenase I, N subunit (464 aa)

ECH_0179	NADH dehydrogenase I, N subunit, putative (491 aa)

ECH_0328	NADH-ubiquinone/plastoquinone oxidoreductase family protein (489 aa)

ECH_0556	NADH dehydrogenase I, N subunit (469 aa)

NSE_0706	NADH-ubiquinone/plastoquinone oxidoreductase family protein (458 aa)

NSE_0692	NADH dehydrogenase I, N subunit (458 aa)

NSE_0563	NADH-ubiquinone/plastoquinone oxidoreductase family protein (475 aa)

RP283	NADH DEHYDROGENASE I CHAIN L (nuoL2) (486 aa)

RP284	NADH DEHYDROGENASE I CHAIN N (nuoN2) (432 aa)

RP537	NADH DEHYDROGENASE I CHAIN N (nuoN1) (458 aa)

WD0969	NADH dehydrogenase I, N subunit, putative (454 aa)

WD1107	Na+/H+ antiporter subunit, putative (488 aa)

WD0816	monovalent cation/proton antiporter subunit, putative (498 aa)

Cluster 295

APH_0542	conserved domain protein (103 aa)

APH_0712	NADH-quinone oxidoreductase, chain G (692 aa)

ECH_0618	NADH dehydrogenase I, G subunit (683 aa)

NSE_0053	NADH dehydrogenase I, G subunit (679 aa)

RP797	NADH DEHYDROGENASE I CHAIN G (nuoG) (675 aa)

WD0160	NADH dehydrogenase I, G subunit (682 aa)

Cluster 296

APH_0435	NADH dehydrogenase I, J subunit (201 aa)

ECH_0550	hypothetical protein (33 aa)

ECH_0552	NADH dehydrogenase I, J subunit (200 aa)

NSE_0010	NADH dehydrogenase I, J subunit (189 aa)

RP790	NADH DEHYDROGENASE I CHAIN J (nuoJ) (205 aa)

WD0965	NADH dehydrogenase I, J subunit (195 aa)

Cluster 297

APH_0520	NADH dehydrogenase I, B subunit (174 aa)

ECH_0787	NADH dehydrogenase I, B subunit (179 aa)

NSE_0431	NADH dehydrogenase I, B subunit (167 aa)

RP356	NADH DEHYDROGENASE I CHAIN B (nuoB) (174 aa)

WD1123	NADH dehydrogenase I, B subunit (168 aa)

Cluster 298

APH_0732	NADH dehydrogenase I, D subunit (395 aa)

ECH_0616	NADH dehydrogenase I, D subunit (393 aa)

NSE_0566	NADH dehydrogenase I, D subunit (388 aa)

RP354	NADH DEHYDROGENASE I CHAIN D (nuoD) (389 aa)

WD0560	NADH dehydrogenase I, D subunit (390 aa)

Cluster 299

APH_0434	NADH dehydrogenase I, F subunit (426 aa)

ECH_0548	NADH dehydrogenase I, F subunit (427 aa)

NSE_0714	NADH dehydrogenase I, F subunit (413 aa)

RP115	NADH DEHYDROGENASE I CHAIN F (nuoF) (421 aa)

WD0976	NADH dehydrogenase I, F subunit, putative (417 aa)

Cluster 300

APH_0801	NADH dehydrogenase I, I subunit (164 aa)

ECH_0691	NADH dehydrogenase I, I subunit (169 aa)

NSE_0820	NADH dehydrogenase I, I subunit (160 aa)

RP795	NADH DEHYDROGENASE I CHAIN I (nuoI) (159 aa)

WD0980	NADH dehydrogenase I, I subunit (169 aa)

Cluster 301

APH_0711	NADH dehydrogenase I, H subunit (340 aa)

ECH_0617	NADH dehydrogenase I, H subunit (367 aa)

NSE_0054	NADH dehydrogenase I, H subunit (336 aa)

RP796	NADH DEHYDROGENASE I CHAIN H (nuoH) (339 aa)

WD0159	NADH dehydrogenase I, H subunit, putative (341 aa)

Cluster 302

APH_0436	NADH dehydrogenase I, K subunit (108 aa)

ECH_0553	NADH dehydrogenase I, K subunit (108 aa)

NSE_0012	NADH dehydrogenase I, K subunit (99 aa)

RP791	NADH DEHYDROGENASE I CHAIN K (nuoK) (110 aa)

WD0966	NADH dehydrogenase I, K subunit (102 aa)

Cluster 303

APH_0519	NADH dehydrogenase I, A subunit (120 aa)

ECH_0786	NADH dehydrogenase I, A subunit (123 aa)

NSE_0429	NADH dehydrogenase I, A subunit (151 aa)

RP357	NADH DEHYDROGENASE I CHAIN A (nuoA) (123 aa)

WD1124	NADH dehydrogenase I, A subunit (117 aa)

Cluster 304

APH_0731	NADH dehydrogenase I, E subunit (171 aa)

ECH_0615	NADH dehydrogenase I, E subunit (181 aa)

NSE_0636	NADH dehydrogenase I, E subunit (181 aa)

RP353	NADH DEHYDROGENASE I CHAIN E (nuoE) (177 aa)

WD0734	NADH dehydrogenase I, E subunit (166 aa)

Cluster 305

APH_0438	NADH dehydrogenase I, M subunit (491 aa)

ECH_0555	NADH dehydrogenase I, M subunit (483 aa)

NSE_0691	NADH dehydrogenase I, M subunit (453 aa)

RP793	NADH DEHYDROGENASE I CHAIN M (nuoM) (491 aa)

WD0968	NADH dehydrogenase I, M subunit (480 aa)

Cluster 306

APH_0437	NADH dehydrogenase I, L subunit (625 aa)

ECH_0554	NADH dehydrogenase I, L subunit (621 aa)

NSE_0014	NADH dehydrogenase I, L subunit (655 aa)

RP792	NADH DEHYDROGENASE I CHAIN L (nuoL1) (653 aa)

WD0967	NADH dehydrogenase I, L subunit (614 aa)

Cluster 307

APH_0601	conserved hypothetical protein (87 aa)

ECH_0625	lipoprotein, putative (133 aa)

NSE_0197	lipoprotein, putative (132 aa)

RP774	unknown (153 aa)

WD0213	lipoprotein, putative (112 aa)

Cluster 308

APH_0504	cell division protein ftsJ FRAMESHIFT (247 aa)

ECH_0533	ribosomal RNA large subunit methyltransferase J (212 aa)

NSE_0546	ribosomal RNA large subunit methyltransferase J (213 aa)

RP163	CELL DIVISION PROTEIN FTSJ (ftsJ) (227 aa)

WD0070	ribosomal RNA large subunit methyltransferase J, putative (192 aa)

Cluster 309

APH_1183	cell division protein FtsA (406 aa)

APH_1164	cell division protein FtsA (394 aa)

ECH_1090	cell division protein FtsA (415 aa)

NSE_0427	cell division protein FtsA (400 aa)

RP251	CELL DIVISION PROTEIN FTSA (ftsA) (411 aa)

WD0166	cell division protein FtsA (412 aa)

Cluster 310

APH_1117	aconitate hydratase 1 (873 aa)

ECH_1030	hypothetical protein (62 aa)

ECH_1031	aconitate hydratase 1 (875 aa)

NSE_0600	aconitate hydratase 1 (905 aa)

RP799	ACONITATE HYDRATASE (acnA) (878 aa)

WD0105	aconitate hydratase (861 aa)

Cluster 311

APH_1166	dehydrogenase, isocitrate/isopropylmalate family (477 aa)

ECH_1114	dehydrogenase, isocitrate/isopropylmalate family (482 aa)

NSE_0172	dehydrogenase, isocitrate/isopropylmalate family (471 aa)

RP265	ISOCITRATE DEHYDROGENASE (icd) (483 aa)

WD0791	isocitrate dehydrogenase, NAD-dependent (472 aa)

Cluster 312

APH_0473	enoyl-(acyl-carrier-protein) reductase (275 aa)

ECH_0811	enoyl-(acyl-carrier-protein) reductase (266 aa)

NSE_0043	enoyl-(acyl-carrier-protein) reductase (265 aa)

RP365	PUTATIVE ENOYL-[ACYL-CARRIER-PROTEIN] REDUCTASE (fabI) (261 aa)

WD0085	enoyl-(acyl-carrier-protein) reductase (261 aa)

Cluster 313

APH_0646	holo-(acyl-carrier-protein) synthase (122 aa)

ECH_0623	holo-(acyl-carrier protein) synthase, truncation (82 aa)

ECH_0741	holo-(acyl-carrier-protein) synthase (123 aa)

NSE_0962	holo-(acyl-carrier protein) synthase (121 aa)

RP577	HOLO-[ACYL-CARRIER PROTEIN] SYNTHASE (acpS) (126 aa)

WD0814	holo-(acyl-carrier-protein) synthase (123 aa)

Cluster 314

APH_0272	signal peptide peptidase SppA, 36K type (298 aa)

ECH_0401	signal peptide peptidase SppA (288 aa)

NSE_0409	signal peptide peptidase SppA, 36K type (308 aa)

RP398	POSSIBLE PROTEASE SOHB (sohB) (308 aa)

RP525	PROTEASE IV (sppA) (305 aa)

WD0602	prophage LambdaWp4, minor capsid protein C, putative (353 aa)

Cluster 315

APH_0158	succinate dehydrogenase, flavoprotein subunit (604 aa)

APH_0161	succinate dehydrogenase, flavoprotein subunit (604 aa)

ECH_0315	succinate dehydrogenase, flavoprotein subunit (598 aa)

NSE_0376	succinate dehydrogenase, flavoprotein subunit (597 aa)

RP128	SUCCINATE DEHYDROGENASE FLAVOPROTEIN SUBUNIT (sdhA) (596 aa)

WD0437	succinate dehydrogenase, flavoprotein subunit (599 aa)

Cluster 316

APH_1053	succinyl-CoA synthetase, alpha subunit (295 aa)

ECH_0980	succinyl-CoA synthetase, alpha subunit (293 aa)

NSE_0250	succinyl-CoA synthetase, alpha subunit (290 aa)

RP432	SUCCINYL-COA LIGASE (sucD) (292 aa)

WD1209	succinyl-CoA synthetase, alpha subunit (291 aa)

Cluster 317

APH_1052	succinyl-CoA synthetase, beta subunit (388 aa)

ECH_0979	succinyl-CoA synthetase, beta subunit (387 aa)

NSE_0251	succinyl-CoA synthetase, beta subunit (392 aa)

RP433	SUCCINYL-COA SYNTHETASE BETA CHAIN (sucC) (386 aa)

WD1210	succinyl-CoA synthase, beta subunit (383 aa)

Cluster 318

APH_1208	beta-hydroxyacyl-(acyl-carrier-protein) dehydratase FabZ (145 aa)

ECH_1073	beta-hydroxyacyl-(acyl-carrier-protein) dehydratase FabZ (143 aa)

NSE_0663	beta-hydroxyacyl-[acyl carrier protein] dehydratase FabZ (151 aa)

RP008	(3R)-HYDROXYMYRISTOYL-[ACYL CARRIER PROTEIN] DEHYDRATASE (fabZ) (145 aa)

WD1083	(3R)-hydroxymyristoyl-(acyl-carrier-protein) dehydratase (143 aa)

Cluster 319

APH_0159	succinate dehydrogenase, iron-sulfur protein (262 aa)

APH_0162	succinate dehydrogenase, iron-sulfur protein (262 aa)

ECH_0316	succinate dehydrogenase and fumarate reductase iron-sulfur protein (258 aa)

NSE_0534	succinate dehydrogenase and fumarate reductase iron-sulfur protein (254 aa)

RP044	SUCCINATE DEHYDROGENASE IRON-SULFUR PROTEIN (sdhB) (261 aa)

WD0727	succinate dehydrogenase, iron-sulfur protein (262 aa)

Cluster 320

APH_1022	ribonuclease HII (210 aa)

ECH_0946	ribonuclease HII (209 aa)

NSE_0701	ribonuclease HII (195 aa)

RP202	RIBONUCLEASE HII (rnhB) (193 aa)

WD1103	ribonuclease HII (198 aa)

Cluster 321

APH_0207	iojap-related protein (127 aa)

ECH_0346	iojap-related protein (123 aa)

NSE_0919	iojap-related protein (110 aa)

RP811	unknown (108 aa)

WD1117	iojap-related protein (105 aa)

Cluster 322

APH_0514	DNA-directed RNA polymerase, omega subunit (135 aa)

ECH_0796	DNA-directed RNA polymerase, omega subunit (138 aa)

NSE_0039	DNA-directed RNA polymerase, omega subunit (128 aa)

RP578	unknown (127 aa)

WD1303	DNA-directed RNA polymerase, omega subunit (135 aa)

Cluster 323

APH_1162	glutaredoxin 3 (80 aa)

APH_1175	glutaredoxin 3, degenerate (37 aa)

ECH_1062	glutaredoxin 3 (81 aa)

NSE_0311	glutaredoxin 3 (89 aa)

RP204	GLUTAREDOXIN 3 (grxC1) (95 aa)

WD0758	glutaredoxin family protein (112 aa)

Cluster 324

APH_0984	2-polyprenylphenol 6-hydroxylase (483 aa)

ECH_0840	2-polyprenylphenol 6-hydroxylase (480 aa)

NSE_0637	ubiquinone biosynthesis protein, putative (458 aa)

RP678	unknown (379 aa)

WD0939	ubiquinone biosynthesis protein AarF, putative (476 aa)

Cluster 325

APH_0347	aromatic-rich protein family (152 aa)

ECH_0473	aromatic-rich protein family (154 aa)

NSE_0323	aromatic rich family protein (159 aa)

RP166	unknown (146 aa)

WD1054	aromatic-rich protein family (153 aa)

Cluster 326

APH_0195	cell division protein FtsQ (258 aa)

ECH_0337	cell division protein FtsQ, putative (271 aa)

NSE_0585	cell division protein, putative (178 aa)

RP250	CELL DIVISION PROTEIN FTSQ (ftsQ) (267 aa)

WD0096	cell division protein FtsQ, putative (252 aa)

Cluster 327

APH_0923	disulfide bond formation protein, DsbB family (157 aa)

ECH_0874	disulfide bond formation family protein (157 aa)

NSE_0340	disulfide bond formation protein, DsbB family (165 aa)

RP370	unknown (172 aa)

WD1099	disulfide bond formation protein, DsbB family (173 aa)

Cluster 328

APH_0998.1	succinate dehydrogenase, cytochrome b556 subunit (127 aa)

ECH_0918	succinate dehydrogenase, cytochrome b556 subunit (128 aa)

NSE_0047	succinate dehydrogenase, cytochrome b556 subunit (140 aa)

RP126	SUCCINATE DEHYDROGENASE CYTOCHROME B560 SUBUNIT (sdhC) (124 aa)

WD1221	succinate dehydrogenase, cytochrome b556 subunit (125 aa)

Cluster 329

APH_0998	succinate dehydrogenase, hydrophobic membrane anchor protein (109 aa)

ECH_0917	succinate dehydrogenase, hydrophobic membrane anchor protein, putative (118 aa)

NSE_0048	succinate dehydrogenase, hydrophobic membrane anchor protein, putative (109 aa)

RP127	SUCCINATE DEHYDROGENASE HYDROPHOBIC MEMBRANE ANCHOR PROTEIN (sdhD) (125 aa)

WD1222	succinate dehydrogenase, hydrophobic membrane anchor protein, putative (118 aa)

Cluster 330

APH_0424	sodium:dicarboxylate symporter family protein (396 aa)

APH_0594	membrane protein, putative (210 aa)

APH_0611	membrane protein, putative (394 aa)

APH_0612	membrane protein, putative (399 aa)

ECH_0581	sodium:dicarboxylate symporter family protein (378 aa)

NSE_0198	transporter, putative (382 aa)

RP176	PROTON/SODIUM-GLUTAMATE SYMPORT PROTEIN (gltP) (399 aa)

WD0211	membrane protein, putative (381 aa)

WD0229	sodium/glutamate symporter family protein, putative (394 aa)

Cluster 331

APH_1209	conserved hypothetical protein (185 aa)

ECH_1072	outer membrane protein, OmpH family (182 aa)

NSE_0664	hypothetical protein (207 aa)

RP264	unknown (184 aa)

WD1084	conserved hypothetical protein (183 aa)

Cluster 332

APH_0192	osmotically inducible protein, putative (198 aa)

ECH_0335	osmotically inducible protein, putative (202 aa)

NSE_0055	osmotically inducible protein, putative (219 aa)

RP391	unknown (213 aa)

WD0321	membrane protein, putative (198 aa)

Cluster 333

APH_0924	membrane protein, putative (242 aa)

APH_0974	phosphatidylglycerophosphatase A (166 aa)

ECH_0875	phosphatidylglycerophosphatase A, putative (226 aa)

ECH_0905	phosphatidylglycerophosphatase A (168 aa)

NSE_0226	phosphatidylglycerophosphatase A, putative (215 aa)

NSE_0292	phosphatidylglycerophosphatase A (169 aa)

RP750	PHOSPHATIDYLGLYCEROPHOSPHATASE A (pgpA) (201 aa)

WD0157	hypothetical protein (242 aa)

WD0730	phosphatidylglycerophosphatase A, putative (160 aa)

Cluster 334

APH_0962	rhodanese domain protein (279 aa)

ECH_0896	rhodanese domain protein (275 aa)

NSE_0255	rhodanese domain protein (302 aa)

RP125	unknown (247 aa)

WD1135	rhodanese domain protein (275 aa)

Cluster 335

APH_1347	conserved hypothetical protein (102 aa)

ECH_0015	conserved hypothetical protein (101 aa)

NSE_0490	conserved hypothetical protein TIGR00103 (105 aa)

RP866	unknown (107 aa)

WD1061	conserved hypothetical protein TIGR00103 (110 aa)

Cluster 336

APH_0572	MutS domain V protein (134 aa)

APH_0857	DNA mismatch repair protein MutS (820 aa)

APH_0883	hypothetical protein (39 aa)

ECH_0824	DNA mismatch repair protein MutS (804 aa)

NSE_0335	DNA mismatch repair protein MutS (815 aa)

RP298	DNA MISMATCH REPAIR PROTEIN MUTS (mutS) (891 aa)

WD0190	DNA mismatch repair protein MutS (831 aa)

Cluster 337

APH_0939	DNA mismatch repair protein MutL (634 aa)

ECH_0884	DNA mismatch repair protein, MutL/HexB family (673 aa)

NSE_0609	DNA mismatch repair protein, MutL/HexB family (652 aa)

RP880	DNA MISMATCH REPAIR PROTEIN MUTL (mutL) (595 aa)

WD1306	DNA mismatch repair protein MutL-1 (608 aa)

WD0509	DNA mismatch repair protein MutL-2 (598 aa)

Cluster 338

APH_0042	malate dehydrogenase (751 aa)

ECH_0175	malate dehydrogenase (758 aa)

NSE_0420	malate dehydrogenase (750 aa)

RP373	MALIC ENZYME (tme) (767 aa)

WD0488	NADP-dependent malic enzyme (440 aa)

Cluster 339

APH_1073	ATP-dependent protease HslV (189 aa)

ECH_0996	ATP-dependent protease HslV (189 aa)

NSE_0176	heat shock protein HslVU, HslV subunit (188 aa)

RP319	HEAT SHOCK PROTEIN HSLV (hslV) (182 aa)

WD1189	heat shock protein HslV (184 aa)

Cluster 340

APH_1074	ATP-dependent Hsl protease, ATP-binding subunit HslU - POINT MUTATION (491 aa)

ECH_0997	heat shock protein HslVU, ATPase subunit HslU (487 aa)

NSE_0177	heat shock protein HslVU, ATPase subunit HslU (472 aa)

RP320	HEAT SHOCK PROTEIN HSLU (hslU) (450 aa)

WD1190	heat shock protein HslVU, ATPase subunit HslU (437 aa)

Cluster 341

APH_0043	conserved hypothetical protein (908 aa)

ECH_0176	conserved hypothetical protein (880 aa)

NSE_0498	hypothetical protein (819 aa)

RP347	OUTER MEMBRANE ASSEMBLY PROTEIN (asmA) (835 aa)

RP349	unknown (868 aa)

WD1011	conserved hypothetical protein (847 aa)

Cluster 342

APH_0608	ppiC/parvulin rotamase family protein (601 aa)

ECH_0731	rotamase family protein (634 aa)

NSE_0645	rotamase family protein (594 aa)

RP822	unknown (528 aa)

WD0797	peptidyl-prolyl cis-trans isomerse D, putative (602 aa)

Cluster 343

APH_0097	protein-export protein SecB (158 aa)

ECH_0233	protein-export protein SecB (175 aa)

NSE_0359	protein-export protein SecB, putative (164 aa)

RP070	PROTEIN-EXPORT PROTEIN SECB (secB) (152 aa)

WD0106	protein-export protein SecB (167 aa)

Cluster 344

APH_0679	iron-sulfur cluster-binding protein (116 aa)

ECH_0634	iron-sulfur cluster binding protein (116 aa)

NSE_0297	iron-sulfur cluster binding protein (111 aa)

RP199	ADRENODOXIN PRECURSOR (adx1) (112 aa)

WD0846	ferredoxin, iron-sulfur cluster assembly system (119 aa)

Cluster 345

APH_0893	chaperone protein HtpG (637 aa)

ECH_0853	chaperone protein HtpG (637 aa)

NSE_0495	heat shock protein HtpG (620 aa)

RP840	HEAT SHOCK PROTEIN HTPG (htpG) (621 aa)

WD1277	heat shock protein HtpG (635 aa)

Cluster 346

APH_0678	chaperone protein HscA (592 aa)

ECH_0633	chaperone protein HscA (617 aa)

NSE_0298	chaperone protein HscA, putative (593 aa)

RP200	HEAT SHOCK PROTEIN HSCA (hscA) (593 aa)

WD0892	chaperone protein HscA (582 aa)

Cluster 347

APH_0677	co-chaperone protein hscB (146 aa)

ECH_0632	co-chaperone Hsc20 (147 aa)

NSE_0299	chaperone protein HscB, putative (159 aa)

RP201	unknown (139 aa)

WD0891	co-chaperone Hsc20 (152 aa)

Cluster 348

APH_0083	hydrolase, alpha/beta fold family (292 aa)

APH_0179	conserved hypothetical protein (227 aa)

ECH_0221	hydrolase, putative (284 aa)

ECH_0326	hydrolase, alpha/beta fold family (260 aa)

NSE_0871	hydrolase, alpha/beta fold family (285 aa)

NSE_0472	hydrolase, putative (279 aa)

RP343	unknown (252 aa)

RP444	unknown (293 aa)

WD0802	hydrolase, alpha/beta fold family (253 aa)

Cluster 349

APH_1165	single-stranded-DNA-specific exonuclease RecJ (585 aa)

ECH_1115	single-stranded-DNA-specific exonuclease RecJ (589 aa)

NSE_0712	single-stranded-DNA-specific exonuclease RecJ (577 aa)

RP528	SINGLE-STRANDED-DNA-SPECIFIC EXONUCLEASE RECJ (recJ) (586 aa)

WD0312	single-stranded-DNA-specific exonuclease RecJ (579 aa)

Cluster 350

APH_0101	peptidase, M16 family (423 aa)

APH_1159	peptidase, M16 family (513 aa)

ECH_0235	peptidase, M16 family (421 aa)

ECH_1057	peptidase, M16 family (439 aa)

NSE_0175	peptidase, M16 family (423 aa)

NSE_0914	peptidase, M16 family (437 aa)

RP219	MITOCHONDRIAL PROTEASE (mpp) (412 aa)

WD0737	peptidase, M16 family, putative (423 aa)

WD0761	protease, insulinase family (446 aa)

Cluster 351

APH_1316	TPR domain protein (342 aa)

ECH_0048	TPR domain protein (356 aa)

NSE_0543	hypothetical protein (354 aa)

RP092	unknown (334 aa)

WD0198	TPR domain protein (358 aa)

Cluster 352

APH_0575	tRNA-dihydrouridine synthase, putative (315 aa)

ECH_0758	tRNA-dihydrouridine synthase, putative (317 aa)

NSE_0373	tRNA-dihydrouridine synthase, putative (331 aa)

RP011	NIFR3-LIKE PROTEIN (nifR3) (341 aa)

WD0025	NifR3 family protein (320 aa)

Cluster 353

APH_0629	malate dehydrogenase, NAD-dependent (321 aa)

ECH_0641	malate dehydrogenase, NAD-dependent (313 aa)

NSE_0956	malate dehydrogenase, NAD-dependent (315 aa)

RP376	MALATE DEHYDROGENASE (mdh) (314 aa)

WD1121	malate dehydrogenase (316 aa)

Cluster 354

APH_0543	CDP-diacylglycerol--serine O-phosphatidyltransferase (264 aa)

ECH_0780	CDP-diacylglycerol--serine O-phosphatidyltransferase, putative (258 aa)

NSE_0247	CDP-diacylglycerol--serine O-phosphatidyltransferase, putative (245 aa)

RP242	CDP-DIACYLGLYCEROL--SERINE O-PHOSPHATIDYLTRANSFERASE (pssA) (224 aa)

WD1048	CDP-diacylglycerol--serine O-phosphatidyltransferase, putative (256 aa)

Cluster 355

APH_0481	ribonuclease BN, putative (277 aa)

ECH_0529	ribonuclease BN, putative (274 aa)

NSE_0395	ribonuclease BN, putative (279 aa)

RP496	unknown (285 aa)

WD1292	ribonuclease, BN family (271 aa)

Cluster 356

APH_0098	conserved hypothetical protein (227 aa)

ECH_0234	conserved hypothetical protein (240 aa)

NSE_0900	conserved hypothetical protein (230 aa)

RP025	unknown (272 aa)

WD1055	conserved hypothetical protein (234 aa)

Cluster 357

APH_0150	CvpA family protein (199 aa)

ECH_0307	CvpA family protein (206 aa)

NSE_0841	CvpA family protein (223 aa)

RP033	unknown (218 aa)

WD0129	membrane protein CvpA, putative (196 aa)

Cluster 358

APH_0394	efflux transporter, RND family, MFP subunit (342 aa)

APH_1043	type I secretion membrane fusion protein, HlyD family (509 aa)

ECH_0510	efflux transporter, RND family, MFP subunit (358 aa)

ECH_0970	type I secretion membrane fusion protein, HlyD family (505 aa)

NSE_0151	efflux transporter, RND family, MFP subunit (348 aa)

NSE_0939	type I secretion membrane fusion protein, HlyD family (494 aa)

RP243	MULTIDRUG RESISTANCE PROTEIN A (emrA) (350 aa)

RP314	ALKALINE PROTEASE SECRETION PROTEIN APRE (aprE) (511 aa)

RP341	unknown (341 aa)

WD0345	efflux transporter, RND family, MFP subunit (360 aa)

WD0649	secretion protein, HlyD family (506 aa)

Cluster 359

APH_0053	major facilitator family transporter (427 aa)

APH_0848	major facilitator family transporter (425 aa)

APH_0895	membrane protein, putative (141 aa)

ECH_0193	major facilitator family transporter (422 aa)

ECH_0818	major facilitator family transporter (413 aa)

NSE_0783	major facilitator family transporter (415 aa)

RP077	PROLINE/BETAINE TRANSPORTER (proP1) (418 aa)

RP852	PROLINE/BETAINE TRANSPORTER (proP6) (415 aa)

RP881	PROLINE/BETAINE TRANSPORTER (proP7) (423 aa)

WD0168	major facilitator family transporter (415 aa)

WD0414	major facilitator family transporter (420 aa)

WD0470	major facilitator family transporter (420 aa)

Cluster 360

APH_1212	TPR repeat protein (88 aa)

ECH_1069	TPR repeat protein (98 aa)

NSE_0941	conserved hypothetical protein (82 aa)

RP599	unknown (87 aa)

WD1087	conserved hypothetical protein (92 aa)

Cluster 361

APH_0444	transporter, AcrB/AcrD/AcrF family (1032 aa)

ECH_0561	AcrB/AcrD/AcrF family protein (1032 aa)

NSE_0192	transporter, AcrB/AcrD/AcrF family (1025 aa)

RP170	ACRIFLAVIN RESISTANCE PROTEIN D (acrD) (1008 aa)

WD1320	multidrug resistance protein D (1024 aa)

Cluster 362

APH_0521	NADH dehydrogenase I, C subunit (188 aa)

APH_0510.1	NADH dehydrogenase I, C subunit, truncation (71 aa)

ECH_0788	NADH dehydrogenase I, C subunit (192 aa)

NSE_0432	NADH dehydrogenase I, C subunit (186 aa)

RP355	NADH DEHYDROGENASE I CHAIN C (nuoC) (197 aa)

WD1122	NADH dehydrogenase I, C subunit (190 aa)

Cluster 363

APH_1305	glutathione-regulated potassium-efflux system protein (571 aa)

ECH_0146	glutathione-regulated potassium-efflux system protein KefB, putative (568 aa)

NSE_0885	glutathione-regulated potassium-efflux system protein (578 aa)

RP810	GLUTATHIONE-REGULATED POTASSIUM-EFFLUX SYSTEM PROTEIN KEFB (kefB) (575 aa)

WD1249	glutathione-regulated potassium-efflux system protein (572 aa)

Cluster 364

APH_0553	NAD-glutamate dehydrogenase family protein (1628 aa)

ECH_0759	hypothetical protein (56 aa)

ECH_0771	NAD-glutamate dehydrogenase family protein (1583 aa)

NSE_0514	NAD-glutamate dehydrogenase family protein (1586 aa)

RP758	unknown (1581 aa)

WD0223	conserved hypothetical protein (1574 aa)

Cluster 365

APH_1113	glutamyl-tRNA(Gln) amidotransferase, C subunit (106 aa)

ECH_1025	glutamyl-tRNA(Gln) amidotransferase subunit C (111 aa)

ECH_1027	hypothetical protein (34 aa)

NSE_0516	glutamyl-tRNA(Gln) amidotransferase, C subunit, putative (91 aa)

RP153	GLUTAMYL-tRNA AMIDOTRANSFERASE SUBUNIT C (gatC) (100 aa)

WD0037	glutamyl-tRNA(Gln) amidotransferase, C subunit, putative (117 aa)

Cluster 366

APH_1110	outer membrane efflux protein (424 aa)

ECH_1020	outer membrane protein TolC, putative (423 aa)

NSE_0798	outer membrane efflux protein (436 aa)

RP224	OUTER MEMBRANE PROTEIN TOLC PRECURSOR (tolC) (456 aa)

WD0068	outer membrane protein TolC, putative (407 aa)

Cluster 367

APH_1232	citrate synthase I (411 aa)

ECH_0124	citrate synthase I (416 aa)

NSE_0730	citrate synthase (398 aa)

RP844	CITRATE SYNTHASE (gltA) (436 aa)

WD1151	citrate synthase (416 aa)

Cluster 368

APH_0671	conserved hypothetical protein (236 aa)

ECH_0627	conserved hypothetical protein (240 aa)

NSE_0892	conserved hypothetical protein (246 aa)

RP471	unknown (238 aa)

WD0706	conserved hypothetical protein (232 aa)

Cluster 369

APH_0344	monovalent cation/proton antiporter, putative (101 aa)

ECH_0469	Na(+)/H(+) antiporter subunit C (111 aa)

NSE_0032	Na(+)/H(+) antiporter subunit C (108 aa)

RP285	unknown (146 aa)

WD1299	monovalent cation/proton antiporter, MnhC/PhaC subunit family (103 aa)

Cluster 370

APH_0342	monovalent cation/proton antiporter, MnhG/PhaG subunit (103 aa)

ECH_0467	monovalent cation/proton antiporter, MnhG/PhaG subunit family (99 aa)

NSE_0035	monovalent cation/proton antiporter, MnhG/PhaG subunit (91 aa)

RP266	unknown (106 aa)

WD1301	monovalent cation/proton antiporter, MnhG/PhaG subunit family (95 aa)

Cluster 371

APH_0736	DNA repair protein RecO, putative (245 aa)

ECH_0536	DNA repair protein RecO, putative (244 aa)

NSE_0461	DNA repair protein RecO, putative (237 aa)

RP549	unknown (135 aa)

WD0219	recO-related protein (240 aa)

Cluster 372

APH_0994	conserved hypothetical protein (220 aa)

ECH_0913	conserved hypothetical protein (218 aa)

NSE_0190	conserved hypothetical protein (223 aa)

RP631	unknown (221 aa)

WD1146	chromosomal DNA replication initiator-related protein (211 aa)

Cluster 373

APH_1015	Na+/H+ ion antiporter family protein (166 aa)

ECH_0944	Na+/H+ ion antiporter family protein (163 aa)

NSE_0519	conserved hypothetical protein (158 aa)

RP156	unknown (103 aa)

WD0765	Na+/H+ ion antiporter family protein (176 aa)

Cluster 374

APH_0122	thiol:disulfide oxidoreductase (248 aa)

ECH_0293	disulfide oxidoreductase (246 aa)

NSE_0901	disulfide oxidoreductase (257 aa)

RP431	unknown (266 aa)

WD1312	DsbA-like disulfide oxidoreductase (252 aa)

Cluster 375

APH_0396	maf protein (193 aa)

ECH_0512	maf protein (192 aa)

NSE_0881	maf protein (209 aa)

RP815	MAF PROTEIN (maf) (222 aa)

WD0333	maf protein (198 aa)

Cluster 376

APH_0508	ribosomal protein L36 (55 aa)

ECH_0656	ribosomal protein L36 (42 aa)

NSE_0927	conserved hypothetical protein (44 aa)

RP456	RIBOSOMAL PROTEIN L36 (rpmJ) (41 aa)

WD0342	ribosomal protein L36 (42 aa)

Cluster 377

APH_1218	DNA-binding protein (183 aa)

ECH_1118	transcriptional regulator, putative (213 aa)

NSE_0230	DNA-binding protein (144 aa)

RP497	unknown (130 aa)

WD0255	transcriptional regulator, putative (308 aa)

WD0508	transcriptional regulator, putative (312 aa)

WD0623	transcriptional regulator, putative (302 aa)

WD0626	transcriptional regulator, putative (303 aa)

Cluster 378

APH_0373	type IV secretion system protein VirB4 (801 aa)

APH_0417	type IV secretion system protein VirB4, N-terminal truncation (43 aa)

APH_0419	type IV secretion system protein VirB4, truncation (277 aa)

ECH_0495	type IV secretion system protein VirB4 (800 aa)

NSE_0863	type IV secretion system protein VirB4 (803 aa)

RP103	VIRB4 PROTEIN PRECURSOR (virB4) (805 aa)

WD0858	type IV secretion system protein VirB4 (801 aa)

Cluster 379

APH_0081	type IV secretion system protein VirB9 (271 aa)

ECH_0219	type IV secretion system protein, VirB9 (270 aa)

NSE_0210	type IV secretion system protein VirB9 (266 aa)

RP286	PROBABLE CONJUGAL TRANSFER PROTEIN TRBG PRECURSOR (trbG) (250 aa)

WD1104	conserved hypothetical protein (260 aa)

Cluster 380

APH_1204	cytochrome c-type biogenesis protein, truncation (122 aa)

APH_1271	cytochrome c-type biogenesis protein, CcmF/CycK/CcsA family (637 aa)

ECH_0137	cytochrome c-type biogenesis protein CcmF (645 aa)

NSE_0572	cytochrome c-type biogenesis protein, CcmF/CycK/CcsA family (611 aa)

RP703	CYTOCHROME C-TYPE BIOGENESIS PROTEIN CCMF (ccmF) (670 aa)

WD0232	cytochrome c-type biogenesis protein CcmF, putative (708 aa)

Cluster 381

APH_1406	type IV secretion protein VirB8 (238 aa)

ECH_0044	type IV secretion system protein VirB8 (237 aa)

NSE_0743	type IV secretion system protein VirB8 (225 aa)

RP289	unknown (243 aa)

WD0004	type IV secretion system protein VirB8 (226 aa)

Cluster 382

APH_1404	type IV secretion system protein VirB10 (434 aa)

ECH_0042	type IV secretion system protein VirB10 (447 aa)

NSE_0741	type IV secretion system protein VirB10 (457 aa)

RP291	VIRB10 PROTEIN (virB10) (483 aa)

WD0006	type IV secretion system protein VirB10 (486 aa)

Cluster 383

APH_0849	conserved hypothetical protein (936 aa)

ECH_0820	conserved hypothetical protein (914 aa)

NSE_0524	conserved domain protein (779 aa)

RP403	unknown (870 aa)

WD0912	conserved hypothetical protein (924 aa)

Cluster 384

APH_0062	conserved hypothetical protein (188 aa)

ECH_0199	conserved hypothetical protein (213 aa)

NSE_0505	conserved hypothetical protein (183 aa)

RP073	unknown (186 aa)

WD0796	conserved hypothetical protein (200 aa)

Cluster 385

APH_1371	uracil-DNA glycosylase, family 4 (290 aa)

ECH_0074	uracil-DNA glycosylase, family 4 (261 aa)

NSE_0935	uracil-DNA glycosylase, family 4 (255 aa)

RP845	unknown (258 aa)

WD0164	phage SPO1 DNA polymerase-related protein (273 aa)

Cluster 386

APH_0544	phosphatidylserine decarboxylase, putative (227 aa)

ECH_0779	phosphatidylserine decarboxylase (226 aa)

NSE_0248	phosphatidylserine decarboxylase-related protein (225 aa)

RP241	unknown (231 aa)

WD1049	phosphatidylserine decarboxylase-related protein (230 aa)

Cluster 387

APH_0961	conserved hypothetical protein (280 aa)

ECH_0895	conserved hypothetical protein (273 aa)

NSE_0324	conserved hypothetical protein (270 aa)

RP001	unknown (273 aa)

WD0341	conserved hypothetical protein (276 aa)

Cluster 388

APH_0180	cytochrome C, membrane-bound (174 aa)

ECH_0327	cytochrome C, membrane-bound (172 aa)

NSE_0472.1	cytochrome c (179 aa)

RP253	CYTOCHROME C (cycM) (175 aa)

WD0803	cytochrome c family protein (158 aa)

Cluster 389

APH_0526	cytochrome c-type biogenesis protein CcmE (141 aa)

ECH_0789	cytochrome c-type biogenesis protein CcmE (133 aa)

NSE_0828	cytochrome c-type biogenesis protein CcmE, putative (135 aa)

RP588	CYTOCHROME C-TYPE BIOGENESIS PROTEIN CCME (ccmE) (128 aa)

WD0972	cytochrome c-type biogenesis protein CcmE (130 aa)

Cluster 390

APH_0125	heme exporter protein CcmA (206 aa)

ECH_0295	heme exporter protein CcmA, putative (217 aa)

NSE_0699	ABC transporter, ATP-binding protein (305 aa)

RP794	HEME EXPORTER PROTEIN A (ccmA) (197 aa)

WD0411	heme exporter protein CcmA (207 aa)

Cluster 391

APH_0096	Tim44-like domain protein (241 aa)

ECH_0232	tim44-like domain protein (212 aa)

NSE_0360	Tim44-like domain protein (208 aa)

RP434	unknown (212 aa)

WD0107	conserved domain protein (216 aa)

Cluster 392

APH_1049	major surface protein MSP5 (209 aa)

ECH_0976	major antigenic protein (205 aa)

NSE_0551	major surface protein (186 aa)

RP587	SCO2 PROTEIN PRECURSOR (sco2) (205 aa)

WD0109	SCO1/SenC family protein (202 aa)

Cluster 393

APH_0775	conserved hypothetical protein (157 aa)

ECH_0744	conserved hypothetical protein (157 aa)

NSE_0462	conserved hypothetical protein (166 aa)

RP727	unknown (159 aa)

WD0128	conserved hypothetical protein (145 aa)

Cluster 394

APH_0266	conserved hypothetical protein (122 aa)

ECH_0398	conserved hypothetical protein (121 aa)

NSE_0749	hypothetical protein (107 aa)

RP756	unknown (105 aa)

WD1088	conserved hypothetical protein (108 aa)

Cluster 395

APH_0045.1	conserved domain protein (101 aa)

ECH_0184	NADH:ubiquinone oxidoreductase family protein (97 aa)

NSE_0528	hypothetical protein (103 aa)

RP725	unknown (100 aa)

WD1106	conserved hypothetical protein (104 aa)

Cluster 396

APH_0987	twin-arginine translocation protein, TatA/E family (56 aa)

ECH_0844	twin-arginine translocation protein, TatA (56 aa)

NSE_0481	twin-arginine translocation protein, TatA/E family (51 aa)

RP749	unknown (54 aa)

WD0152	mttA/Hcf106 family protein (55 aa)

Cluster 397

APH_1243	5-aminolevulinic acid synthase (414 aa)

APH_0979	8-amino-7-oxononanoate synthase, truncation (273 aa)

APH_1016	8-amino-7-oxononanoate synthase (378 aa)

ECH_0092	5-aminolevulinic acid synthase (401 aa)

ECH_0950	8-amino-7-oxononanoate synthase (367 aa)

NSE_0613	8-amino-7-oxononanoate synthase (393 aa)

NSE_0824.1	5-aminolevulinic acid synthase (406 aa)

RP841	5-AMINOLEVULINIC ACID SYNTHASE (hemA) (414 aa)

WD1279	5-aminolevulinic acid synthase (402 aa)

Cluster 398

APH_1219	Omp-1X (306 aa)

APH_1240	major surface 4 (282 aa)

ECH_1119	major outer membrane protein OMP-1M (298 aa)

ECH_1127	major outer membrane protein OMP-1V (279 aa)

ECH_1133	major outer membrane protein OMP-1H (298 aa)

ECH_1135	major outer membrane protein OMP-1A (293 aa)

ECH_1136	major outer membrane protein OMP-1B (283 aa)

ECH_1137	major outer membrane protein OMP-1C (280 aa)

ECH_1140	major outer membrane protein OMP-1E (278 aa)

ECH_1143	major outer membrane protein P28 (281 aa)

NSE_0875	outer surface protein, putative (246 aa)

RP827	unknown (248 aa)

WD1063	surface antigen Wsp (237 aa)

WD0009	surface antigen Wsp paralog (282 aa)

WD0489	surface antigen, Wsp paralog (308 aa)

Cluster 399

APH_1307	conserved hypothetical protein (147 aa)

ECH_0148	membrane protein, putative (144 aa)

NSE_0349	membrane protein, putative (136 aa)

RP883	unknown (145 aa)

WD0417	membrane protein, putative (142 aa)

Cluster 400

APH_0003	ABC transporter, permease protein, putative (244 aa)

ECH_0084	conserved hypothetical protein (275 aa)

NSE_0697	ABC transporter, permease protein, putative (250 aa)

RP096	unknown (259 aa)

WD0989	membrane protein, putative (260 aa)

Cluster 401

APH_0045	mce-related protein (150 aa)

ECH_0183	mce-related protein (149 aa)

NSE_0527	mce-related protein (145 aa)

RP724	unknown (149 aa)

WD1105	ABC transporter, periplasmic substrate-binding protein, putative (151 aa)

Cluster 402

APH_0401	ubiquinol-cytochrome c reductase, iron-sulfur subunit (189 aa)

ECH_0520	ubiquinol-cytochrome c reductase, iron-sulfur subunit (187 aa)

NSE_0624	ubiquinol-cytochrome c reductase, iron-sulfur subunit (173 aa)

RP270	CYTOCHROME B6-F COMPLEX IRON-SULFUR SUBUNIT (petA) (177 aa)

WD1201	ubiquinol-cytochrome c reductase, iron-sulfur subunit (199 aa)

Cluster 403

APH_0403	ubiquinol-cytochrome c reductase, cytochrome c1 (250 aa)

ECH_0522	ubiquinol-cytochrome c reductase, cytochrome c1 (252 aa)

NSE_0626	ubiquinol-cytochrome c reductase, cytochrome c1 (256 aa)

RP272	CYTOCHROME C1, HEME PROTEIN PRECURSOR (fbcH) (253 aa)

WD1070	ubiquinol-cytochrome c reductase, cytochrome c1 (252 aa)

Cluster 404

APH_0352	conserved hypothetical protein (165 aa)

ECH_0478	conserved hypothetical protein (172 aa)

NSE_0455	conserved hypothetical protein (193 aa)

RP867	unknown (231 aa)

WD0236	conserved hypothetical protein (171 aa)

Cluster 405

APH_0354	uroporphyrinogen-III synthase, putative (230 aa)

ECH_0480	uroporphyrinogen-III synthase (229 aa)

NSE_0441	hypothetical protein (236 aa)

RP474	unknown (269 aa)

WD1000	uroporphyrinogen-III synthase, putative (235 aa)

Cluster 406

APH_1136	conserved hypothetical protein (144 aa)

APH_1144	conserved hypothetical protein (123 aa)

ECH_1042	conserved hypothetical protein (124 aa)

ECH_1043	conserved hypothetical protein (101 aa)

ECH_1044	conserved hypothetical protein (118 aa)

NSE_0770	type IV secretion system protein VirB2 (115 aa)

RP192	unknown (128 aa)

WD1288	hypothetical protein (59 aa)

WD1289	hypothetical protein (117 aa)

WD1290	hypothetical protein (74 aa)

WD0474	hypothetical protein (124 aa)

WD0651	hypothetical protein (107 aa)

Cluster 407

APH_1402	type IV secretion system protein VirD4 (740 aa)

ECH_0040	type IV secretion system protein VirD4 (714 aa)

NSE_0739	type IV secretion system protein VirD4 (641 aa)

RP293	VIRD4 PROTEIN (virD4) (591 aa)

WD0008	type IV secretion system protein VirD4 (648 aa)

Cluster 408

APH_0911	Coq7 family protein (164 aa)

ECH_0873	Coq7 family protein (170 aa)

NSE_0339	Coq7 family protein (175 aa)

RP190	UBIQUINONE BIOSYNTHESIS PROTEIN COQ7 (coq7) (178 aa)

WD1100	Coq7 family protein (180 aa)

Cluster 409

APH_0404	hypothetical protein (574 aa)

APH_0405	hypothetical protein (536 aa)

APH_0406	hypothetical protein (559 aa)

APH_0407	hypothetical protein (65 aa)

ECH_0523	conserved domain protein (489 aa)

ECH_0525	conserved domain protein (666 aa)

ECH_0526	conserved domain protein (495 aa)

ECH_0593	conserved hypothetical protein (382 aa)

ECH_0606	hypothetical protein (49 aa)

NSE_0242	51 kDa major antigen (511 aa)

RP068	unknown (440 aa)

WD0082	conserved hypothetical protein (375 aa)

WD0745	hypothetical protein (659 aa)

Cluster 410

APH_0565	conserved hypothetical protein (93 aa)

ECH_0764	conserved hypothetical protein (90 aa)

ECH_0765	hypothetical protein (79 aa)

NSE_0938	conserved hypothetical protein (100 aa)

RP240	unknown (107 aa)

WD0715	conserved hypothetical protein (94 aa)

Cluster 411

APH_0627	RDD family protein (201 aa)

ECH_0643	RDD family protein (195 aa)

NSE_0542	RDD family protein (216 aa)

RP437	unknown (173 aa)

WD0355	conserved domain protein (198 aa)

Cluster 412

APH_0256	type I secretion system ATPase/hypothetical protein (995 aa)

ECH_0383	type I secretion system ATPase (590 aa)

NSE_0042	type I secretion system ATPase (577 aa)

RP315	ALKALINE PROTEASE SECRETION ATP-BINDING PROTEIN APRD (aprD) (583 aa)

WD0770	type I secretion system ATPase (581 aa)

Cluster 413

APH_0734	pyridine nucleotide-disulphide oxidoreductase family protein (338 aa)

ECH_0649	pyridine nucleotide-disulphide oxidoreductase family protein (338 aa)

NSE_0779	pyridine nucleotide-disulphide oxidoreductase family protein (333 aa)

RP514	THIOREDOXIN REDUCTASE (trxB2) (334 aa)

WD0982	pyridine nucleotide-disulphide oxidoreductase family protein (338 aa)

Cluster 414

APH_0077	auxin efflux carrier family protein (307 aa)

ECH_0212	transporter, putative (304 aa)

NSE_0173	transporter, putative (304 aa)

RP443	unknown (309 aa)

WD0249	membrane protein, putative (309 aa)

Cluster 415

APH_1405	type IV secretion system protein VirB9 (281 aa)

ECH_0043	type IV secretion system protein VirB9 (273 aa)

NSE_0742	type IV secretion system protein VirB9 (240 aa)

RP290	VIRB9 PROTEIN PRECURSOR (virB9) (157 aa)

WD0005	type IV secretion system protein VirB9 (264 aa)

Cluster 416

APH_0328	conserved domain protein (315 aa)

ECH_0454	conserved hypothetical protein (302 aa)

NSE_0971	conserved hypothetical protein (274 aa)

RP395	unknown (333 aa)

WD1191	conserved domain protein (313 aa)

Cluster 417

APH_1042	conserved hypothetical protein (234 aa)

ECH_0967	conserved hypothetical protein (213 aa)

NSE_0760	conserved hypothetical protein (196 aa)

RP616	unknown (203 aa)

WD0850	rpsU-divergently transcribed protein (196 aa)

Cluster 418

APH_0430	conserved hypothetical protein (207 aa)

ECH_0579	type IV secretion system protein VirB8 (229 aa)

NSE_0211	type IV secretion system protein VirB8 (215 aa)

RP287	VIRB8 PROTEIN (virB8) (247 aa)

WD0817	type IV secretion system protein VirB8, putative (220 aa)

Cluster 419

APH_1394	conserved hypothetical protein (202 aa)

ECH_0069	conserved hypothetical protein (209 aa)

NSE_0937	conserved hypothetical protein (202 aa)

RP094	unknown (199 aa)

WD1053	conserved hypothetical protein (196 aa)

Cluster 420

APH_0123	cytochrome oxidase assembly protein, putative (328 aa)

ECH_0294	cytochrome oxidase assembly protein, putative (344 aa)

NSE_0855	cytochrome oxidase assembly protein, putative (325 aa)

RP257	CYTOCHROME C OXIDASE ASSEMBLY PROTEI (coxW) (334 aa)

WD0432	cytochrome c oxidase assembly protein (350 aa)

Cluster 421

APH_1150	cytochrome c oxidase assembly protein CtaG (183 aa)

ECH_1055	cytochrome c oxidase assembly protein CtaG (175 aa)

NSE_0258	cytochrome c oxidase assembly protein CtaG (181 aa)

RP304	CYTOCHROME C OXIDASE ASSEMBLY PROTEIN COX11 (cox11) (178 aa)

WD0949	cytochrome c oxidase assembly protein CtaG, putative (178 aa)

Cluster 422

APH_0740	ankyrin (1232 aa)

ECH_0684	ankyrin repeat protein (1463 aa)

NSE_0015	ankyrin repeat protein (201 aa)

RP714	ANKYRIN, BRAIN VARIANT 2 (ank2) (107 aa)

WD0147	ankyrin repeat domain protein (954 aa)

WD0294	ankyrin repeat domain protein (541 aa)

WD0385	ankyrin repeat domain protein (542 aa)

WD0550	ankyrin repeat domain protein (329 aa)

WD0766	ankyrin repeat domain protein (474 aa)
