# Supplementary material for: Comparative Genomics of Emerging Human Ehrlichiosis Agents
Source: PLoS Genet. 2006 Feb 17;2(2):e21. doi: 10.1371/journal.pgen.0020021 (PMC1366493; doi:10.1371/journal.pgen.0020021)
Supplement: Supplementary file 10 [file pgen.0020021.st005.doc]

**Supplementary Table 5.** Ortholog clusters (see methods) present in *Neorickettsia sennetsu*, *Anaplasma phagocytophilum*, and *Ehrlichia chaffeensis*, but not in *Wolbachia pipientis* and *Rickettsia prowazekii*.

| Common Name | APH^[1]^ | ECH | NES |
| --- | --- | --- | --- |
| Glutamine-dependent NAD(+) synthetase, putative | APH_0852 | ECH_0822 | NSE_0097 |
| Nicotinate (nicotinamide) nucleotide adenylyltransferase, putative | APH_0791 | ECH_0806 | NSE_0711 |
| ATP cone domain protein | APH_0047 | ECH_0185 | NSE_0352 |
| Monovalent cation/proton antiporter, MrpF/PhaF subunit, putative | APH_0341 | ECH_0466 | NSE_0183 |
| Dihydroneopterin aldolase | APH_0628 | ECH_0642 | NSE_0501 |
| Biotin synthase | APH_0218 | ECH_0352 | NSE_0612 |
| Dethiobiotin synthetase | APH_1228 | ECH_1110 | NSE_0617 |
| Thiamine-monophosphate kinase | APH_0735 | ECH_0528 | NSE_0253 |
| Thiamine-phosphate pyrophosphorylase | APH_0960  APH_0993 | ECH_0893  ECH_0914 | NSE_0220  NSE_0954 |
| Thiamin biosynthesis ThiG | APH_0070 | ECH_0206 | NSE_0483 |
| Thiamine biosynthesis protein ThiS | APH_0069 | ECH_0204 | NSE_0044 |
| Adenylyltransferase thiF | APH_1174 | ECH_1107 | NSE_0777 |
| TRAP transporter, 4TM/12TM fusion protein | APH_0131  APH_0149 | ECH_0306 | NSE_0842 |
| Oxidoreductase, short chain dehydrogenase/reductase family | APH_0056 | ECH_0195 | NSE_0966 |
| Quinolinate synthetase complex, A subunit | APH_0026 | ECH_0036 | NSE_0969 |
| Nicotinate-nucleotide pyrophosphorylase | APH_0016 | ECH_0026 | NSE_0122 |
| Conserved hypothetical protein | APH_0201  APH_0209 | ECH_0348 | NSE_0507 |
| DNA-binding protein | APH_1100 | ECH_1013 | NSE_0508 |
| Hypothetical protein | APH_0005 | ECH_0010 | NSE_0573 |
| Hypothetical protein | APH_0265 | ECH_0396 | NSE_0791 |

1. APH, *Anaplasma phagocytophilum*; ECH, *Ehrlichia chaffeensis*; NES, *Neorickettsia sennetsu*.
