# Supplementary material for: Comparative Genomics of Emerging Human Ehrlichiosis Agents
Source: PLoS Genet. 2006 Feb 17;2(2):e21. doi: 10.1371/journal.pgen.0020021 (PMC1366493; doi:10.1371/journal.pgen.0020021)
Supplement: Supplementary file 11 [file pgen.0020021.st006.doc]

**Supplementary Table 6.** Ortholog clusters (see methods) present in *Wolbachia pipientis*, *Anaplasma phagocytophilum*, and *Ehrlichia chaffeensis*, but not in *Neorickettsia sennetsu* and *Rickettsia prowazekii.*

| Common Name | APH^[1]^ | ECH | WOL |
| --- | --- | --- | --- |
| Formamidopyrimidine-DNA glycosylase | APH_0411 | ECH_0602 | WD1158 |
| Acetylglutamate kinase | APH_0422 |  | WD0245 |
| Proline dehydrogenase/delta-1-pyrroline-5-carboxylate dehydrogenase | APH_0669 | ECH_0667 | WD0103 |
| Conserved hypothetical protein | APH_1291 | ECH_1154 | WD0724 |
| Conserved hypothetical protein | APH_0894  APH_0907 | ECH_0854 | WD1220 |
| Peptidase, M48 family | APH_0208 | ECH_0347 | WD0652 |
| Hypothetical protein | APH_0203 | ECH_0343 | WD1025 |
| Cytochrome c-type biogenesis protein CcmH, putative | APH_0687 | ECH_0636 | WD0844 |
| Conserved domain protein | APH_1339 | ECH_0129 | WD1232 |
| Conserved hypothetical protein TIGR02217 | APH_0378 | ECH_0500 | WD1311 |
| Phage conserved hypothetical protein BR0599 | APH_0688 | ECH_0835 | WD0748 |
| ApaG protein | APH_1120 | ECH_1033 | WD1141 |
| Phage conserved hypothetical protein, phiE125 gp8 family | APH_0634 | ECH_0660 | WD0076 |
| Hypothetical protein | APH_0049  APH_0050 | ECH_0187  ECH_0188 | WD1041  WD1199 |
| Malonyl-CoA decarboxylase, putative | APH_0469 | ECH_0814 | WD0478 |
| Conserved hypothetical protein | APH_0683  APH_0696 | ECH_0836 | WD0382 |
| SPFH domain/band 7 family protein | APH_0260 | ECH_0390 | WD0482 |
| Es1 family protein | APH_0006 | ECH_0012 | WD1271 |
| Conserved hypothetical protein | APH_1320 | ECH_0052 | WD0764 |
| Conserved hypothetical protein | APH_0976 | ECH_0907 | WD0728 |
| Type IV secretion system protein VirB4 | APH_1129 | ECH_1041 | WD1173 |
| Hypothetical protein | APH_0737 | ECH_0535 | WD0991 |
| Twin-arginine translocation protein TatB, putative | APH_0552 | ECH_0772 | WD0222 |
| Conserved hypothetical protein | APH_0322 | ECH_0450 | WD0412 |
| Hypothetical protein | APH_1066 | ECH_0986 | WD0773 |
| Hypothetical protein | APH_1340 | ECH_0128 | WD1230 |
| Hypothetical protein | APH_0366 | ECH_0489 | WD0835 |
| Conserved hypothetical protein | APH_1378 | ECH_1103 | WD0757 |
| Conserved hypothetical protein | APH_0636 | ECH_0662 | WD0078 |
| Hypothetical protein | APH_0635 | ECH_0661 | WD0077 |

1. APH, *Anaplasma phagocytophilum*; ECH, *Ehrlichia chaffeensis*; WOL, *Wolbachia pipientis* *w*Mel.
