# Supplementary material for: Comparative Genomics of Emerging Human Ehrlichiosis Agents
Source: PLoS Genet. 2006 Feb 17;2(2):e21. doi: 10.1371/journal.pgen.0020021 (PMC1366493; doi:10.1371/journal.pgen.0020021)
Supplement: Supplementary file 12 [file pgen.0020021.st007.doc]

**Supplementary Table 7.** Ortholog clusters (see methods) present in *Rickettsia prowazekii* and *Wolbachia pipientis* but not in *Neorickettsia sennetsu*, *Anaplasma phagocytophilum*, or *Ehrlichia chaffeensis.*

| Common Name | RPR^[1]^ | WOL |
| --- | --- | --- |
| Rod shape-determining protein RodA | RP280  RP411 | WD1108  WD0394 |
| Penicillin-binding protein | RP565  RP567 | WD1273  WD0719 |
| Phosphoglucomutase/phosphomannomutase family protein | RP509 | WD0695 |
| UDP-N-acetylmuramate--alanine ligase | RP247 | WD0495 |
| Phospho-N-acetylmuramoyl-pentapeptide-transferase | RP595 | WD1102 |
| UDP-N-acetylmuramoylalanine--D-glutamate ligase | RP410 | WD0849 |
| UDP-N-acetylglucosamine--N-acetylmuramyl-(pentapeptide) pyrophosphoryl-undecaprenol N-acetylglucosamine transferase | RP412 | WD0323 |
| D-alanine--D-alanine ligase | RP249 | WD0095 |
| BioY family protein | RP324 | WD1206 |
| Glycosyl transferase, group 1 family protein / moaA/nifB/pqqE family protein | RP344  RP414 | WD0613 |
| Hypothetical protein | RP081  RP083 | WD0512 |
| (Di)nucleoside polyphosphate hydrolase | RP236 | WD1119 |
| Conserved hypothetical protein | RP423 | WD0343 |
| Rare lipoprotein A, putative | RP390 | WD0496 |
| Amino acid permease family protein | RP307  RP483 | WD0957 |
| Conserved hypothetical protein | RP809 | WD1176 |
| Conserved domain protein | RP568 | WD0171 |
| Cytochrome d ubiquinol oxidase, subunit I | RP216 | WD0740 |
| Cytochrome d ubiquinol oxidase, subunit II | RP217 | WD0741 |
| UDP-glucose 6-dehydrogenase | RP779 | WD0620 |
| Nuclease-related protein | RP819 | WD1243 |
| Conserved hypothetical protein | RP402 | WD1015 |
| Patatin family protein | RP602 | WD0565 |

1. WOL, *Wolbachia pipientis* *w*Mel; RPR *Rickettsia prowazekii*.
