# Supplementary material for: Comparative Genomics of Emerging Human Ehrlichiosis Agents
Source: PLoS Genet. 2006 Feb 17;2(2):e21. doi: 10.1371/journal.pgen.0020021 (PMC1366493; doi:10.1371/journal.pgen.0020021)
Supplement: Supplementary file 13 [file pgen.0020021.st008.doc]

# Supplementary Table 8. Ortholog clusters (see methods) present in *Anaplasma phagocytophilum* and *Ehrlichia chaffeensis* but not in *Rickettsia prowazekii*, *Wolbachia pipientis*, or *Neorickettsia sennetsu.*

| Common Name | APH^[1]^ | ECH |
| --- | --- | --- |
| Conserved hypothetical protein | APH_1213  APH_0985 | ECH_0846  ECH_1068 |
| Conserved hypothetical protein | APH_0052 | ECH_0194 |
| Potassium uptake protein TrkH, putative | APH_1182 | ECH_1093 |
| FAD-dependent oxidoreductase | APH_0325 | ECH_0452 |
| Competence/damage-inducible protein CinA C-terminal domain | APH_1265 | ECH_0005 |
| RDD family protein | APH_0186 | ECH_0331 |
| YjeF family protein | APH_0588 | ECH_0751 |
| Conserved hypothetical protein | APH_1337 | ECH_0130 |
| Thiamin biosynthesis protein ThiC | APH_0586  APH_0600 | ECH_0798 |
| Conserved hypothetical protein | APH_1130 | ECH_1047 |
| Membrane protein, putative | APH_0932  APH_0949 | ECH_0888 |
| Polypeptide deformylase, putative | APH_1012 | ECH_0939 |
| Lipoprotein, putative | APH_0355 | ECH_0482 |
| Transcriptional activator, putative, Baf family | APH_1014 | ECH_0941 |
| Conserved domain protein | APH_0248 | ECH_0377 |
| Hypothetical protein | APH_0755 | ECH_0679 |
| Hypothetical protein | APH_1235 | ECH_0122 |
| Hypothetical protein | APH_1280 | ECH_0161 |
| Hypothetical protein | APH_0384 | ECH_0692 |
| Hypothetical protein | APH_0792 | ECH_0807 |
| Conserved hypothetical protein | APH_0267 | ECH_0399 |
| Hypothetical protein | APH_0506 | ECH_0678 |
| Hypothetical protein | APH_0859 | ECH_0825 |
| Hypothetical protein | APH_0997 | ECH_0916 |
| Hypothetical protein | APH_0365 | ECH_0488 |
| Hypothetical protein | APH_0068 | ECH_0203 |
| Hypothetical protein | APH_1069 | ECH_0991 |
| Hypothetical protein | APH_0643 | ECH_0639 |
| Hypothetical protein | APH_0219 | ECH_0353 |
| Twitching motility protein PilT, putative | APH_0909 | ECH_0871 |
| Ankyrin repeat protein | APH_0928 | ECH_0877 |
| Conserved hypothetical protein | APH_0255 | ECH_0382 |

1. APH, *Anaplasma phagocytophilum*; ECH, *Ehrlichia chaffeensis*.
