# Supplementary material for: Comparative Genomics of Emerging Human Ehrlichiosis Agents
Source: PLoS Genet. 2006 Feb 17;2(2):e21. doi: 10.1371/journal.pgen.0020021 (PMC1366493; doi:10.1371/journal.pgen.0020021)
Supplement: Supplementary file 14 [file pgen.0020021.st009.doc]

**Supplementary Table 9.** Individual genes (based on ortholog cluster analysis, see methods) present in *Anaplasma phagocytophilum* but not in *Ehrlichia chaffeensis,* *Rickettsia prowazekii*, *Wolbachia pipientis*, or *Neorickettsia sennetsu.*

| Locus | Common Name |
| --- | --- |
| APH_0010 | Hypothetical protein |
| APH_0024 | Hypothetical protein |
| APH_0028 | Hypothetical protein |
| APH_0029 | P44-63 outer membrane protein, silent |
| APH_0030 | P44-64 outer membrane protein, silent |
| APH_0031 | Hypothetical protein |
| APH_0032 | 130kDa protein |
| APH_0033 | Hypothetical protein |
| APH_0055 | Hypothetical protein |
| APH_0057 | Hypothetical protein |
| APH_0063 | Hypothetical protein |
| APH_0076 | P44-65 outer membrane protein, silent |
| APH_0095 | Hypothetical protein |
| APH_0099 | Hypothetical protein |
| APH_0100 | Hypothetical protein |
| APH_0102 | Hypothetical protein |
| APH_0105 | Hypothetical protein |
| APH_0108 | Hypothetical protein |
| APH_0114 | Hypothetical protein |
| APH_0115 | P44-66 outer membrane protein, silent |
| APH_0116 | Hypothetical protein |
| APH_0117 | Hypothetical protein |
| APH_0118 | Hypothetical protein |
| APH_0121 | Hypothetical protein |
| APH_0124 | Hypothetical protein |
| APH_0126 | Hypothetical protein |
| APH_0127 | Hypothetical protein |
| APH_0128 | Hypothetical protein |
| APH_0132 | P44-21 outer membrane protein, silent |
| APH_0133 | Hypothetical protein |
| APH_0134 | P44-80 outer membrane protein, truncation |
| APH_0143 | P44-81 outermembrane protein, truncation |
| APH_0144 | Major surface protein 2, P44 family |
| APH_0148 | Hypothetical protein |
| APH_0155 | Hypothetical protein |
| APH_0169 | Hypothetical protein |
| APH_0171 | P44-45 outer membrane protein |
| APH_0172 | P44-69 |
| APH_0173 | Hypothetical protein |
| APH_0174 | P44-55 outer membrane protein, silent |
| APH_0177 | Hypothetical protein |
| APH_0178 | Hydrolase, alpha/beta fold family |
| APH_0182 | Hypothetical protein |
| APH_0183 | Hypothetical protein |
| APH_0184 | Hypothetical protein |
| APH_0188 | Hypothetical protein |
| APH_0189 | Hypothetical protein |
| APH_0191 | Hypothetical protein |
| APH_0194 | Hypothetical protein |
| APH_0197 | Hypothetical protein |
| APH_0204 | Hypothetical protein |
| APH_0214 | P44-88 outer membrane protein, silent |
| APH_0215 | Hypothetical protein |
| APH_0217 | Conserved hypothetical protein |
| APH_0223 | Hypothetical protein |
| APH_0226 | Human granulocytic ehrlichiosis antigen |
| APH_0227 | Hypothetical protein |
| APH_0228 | P44-67 outer membrane protein, silent |
| APH_0229 | P44-68 outer membrane protein, silent |
| APH_0233 | Hypothetical protein |
| APH_0234 | Membrane protein, putative |
| APH_0237 | Hypothetical protein |
| APH_0238 | Hypothetical protein |
| APH_0239 | Hypothetical protein |
| APH_0244 | Hypothetical protein |
| APH_0253 | Hypothetical protein |
| APH_0264 | Hypothetical protein |
| APH_0269 | Hypothetical protein |
| APH_0270 | Hypothetical protein |
| APH_0312 | Hypothetical protein |
| APH_0318 | P44 outer membrane protein, N-terminal fragment |
| APH_0319 | P44-69 outer membrane protein, silent |
| APH_0320 | Hypothetical protein |
| APH_0324 | Hypothetical protein |
| APH_0333 | Hypothetical protein |
| APH_0334 | Hypothetical protein |
| APH_0335 | Hypothetical protein |
| APH_0357 | Hypothetical protein |
| APH_0358 | Hypothetical protein |
| APH_0360 | Hypothetical protein |
| APH_0361 | Hypothetical protein |
| APH_0363 | Hypothetical protein |
| APH_0369 | Hypothetical protein |
| APH_0382 | HGE-14 protein |
| APH_0385 | HGE-14 protein |
| APH_0386 | HGE-14 protein |
| APH_0387 | HGE-14 protein |
| APH_0388 | Hypothetical protein |
| APH_0391 | Hypothetical protein |
| APH_0400 | Hypothetical protein |
| APH_0409 | Hypothetical protein |
| APH_0413 | Hypothetical protein |
| APH_0414 | Hypothetical protein |
| APH_0416 | Hypothetical protein |
| APH_0418 | Type IV secretion system protein VirB6,C-terminal truncation |
| APH_0425 | Hypothetical protein |
| APH_0426 | Hypothetical protein |
| APH_0429 | Hypothetical protein |
| APH_0452 | HGE-14 protein |
| APH_0453 | HGE-14 protein |
| APH_0455 | HGE-14 protein |
| APH_0456 | Hypothetical protein |
| APH_0457 | HGE-14 protein |
| APH_0458 | Hypothetical protein |
| APH_0459 | Hypothetical protein |
| APH_0460 | Hypothetical protein |
| APH_0461 | Hypothetical protein |
| APH_0464 | Hypothetical protein |
| APH_0466 | Hypothetical protein |
| APH_0467 | Hypothetical protein |
| APH_0475 | Hypothetical protein |
| APH_0476 | Hypothetical protein |
| APH_0477 | Hypothetical protein |
| APH_0483 | Hypothetical protein |
| APH_0484 | Hypothetical protein |
| APH_0485 | Hypothetical protein |
| APH_0486 | Hypothetical protein |
| APH_0487 | Hypothetical protein |
| APH_0488 | Hypothetical protein |
| APH_0489 | Hypothetical protein |
| APH_0490 | Hypothetical protein |
| APH_0491 | Hypothetical protein |
| APH_0497 | Hypothetical protein |
| APH_0499 | Hypothetical protein |
| APH_0507 | Hypothetical protein |
| APH_0509 | Hypothetical protein |
| APH_0510 | Lipoprotein, putative |
| APH_0512 | Hypothetical protein |
| APH_0516 | Hypothetical protein |
| APH_0517 | Hypothetical protein |
| APH_0518 | Hypothetical protein |
| APH_0522 | Hypothetical protein |
| APH_0523 | Hypothetical protein |
| APH_0525 | Conserved domain protein |
| APH_0529 | Hypothetical protein |
| APH_0531 | Hypothetical protein |
| APH_0533 | Hypothetical protein |
| APH_0535 | P44-82 outer membrane protein, truncation |
| APH_0550 | Hypothetical protein |
| APH_0556 | Hypothetical protein |
| APH_0557 | Hypothetical protein |
| APH_0558 | Hypothetical protein |
| APH_0559 | Hypothetical protein |
| APH_0562 | Hypothetical protein |
| APH_0563 | Hypothetical protein |
| APH_0567 | Hypothetical protein |
| APH_0568 | Hypothetical protein |
| APH_0569 | Hypothetical protein |
| APH_0570 | Hypothetical protein |
| APH_0577 | Hypothetical protein |
| APH_0578 | Hypothetical protein |
| APH_0580 | Hypothetical protein |
| APH_0583 | Hypothetical protein |
| APH_0587 | Hypothetical protein |
| APH_0589 | Hypothetical protein |
| APH_0590 | Hypothetical protein |
| APH_0591 | Hypothetical protein |
| APH_0592 | Hypothetical protein |
| APH_0593 | Hypothetical protein |
| APH_0595 | Hypothetical protein |
| APH_0596 | Hypothetical protein |
| APH_0598 | Hypothetical protein |
| APH_0599 | Hypothetical protein |
| APH_0602 | Hypothetical protein |
| APH_0603 | Hypothetical protein |
| APH_0604 | Hypothetical protein |
| APH_0605 | Hypothetical protein |
| APH_0609 | Hypothetical protein |
| APH_0618 | Hypothetical protein |
| APH_0619 | Hypothetical protein |
| APH_0620 | Hypothetical protein |
| APH_0621 | Hypothetical protein |
| APH_0630 | Hypothetical protein |
| APH_0631 | Hypothetical protein |
| APH_0633 | Hypothetical protein |
| APH_0637 | Hypothetical protein |
| APH_0638 | Hypothetical protein |
| APH_0640 | Hypothetical protein |
| APH_0645 | Hypothetical protein |
| APH_0652 | Hypothetical protein |
| APH_0653 | Hypothetical protein |
| APH_0654 | Hypothetical protein |
| APH_0655 | Hypothetical protein |
| APH_0656 | Hypothetical protein |
| APH_0657 | Hypothetical protein |
| APH_0661 | Hypothetical protein |
| APH_0662 | P44-70 outer membrane protein |
| APH_0663 | P44-71 outer membrane protein |
| APH_0664 | P44-72 outer membrane protein |
| APH_0665 | Hypothetical protein |
| APH_0666 | Hypothetical protein |
| APH_0667 | Hypothetical protein |
| APH_0670 | Hypothetical protein |
| APH_0672 | Hypothetical protein |
| APH_0680 | Hypothetical protein |
| APH_0681 | Hypothetical protein |
| APH_0682 | Hypothetical protein |
| APH_0684 | Hypothetical protein |
| APH_0685 | Hypothetical protein |
| APH_0686 | Hypothetical protein |
| APH_0689 | Hypothetical protein |
| APH_0691 | Hypothetical protein |
| APH_0692 | Hypothetical protein |
| APH_0702 | Hypothetical protein |
| APH_0708 | Hypothetical protein |
| APH_0710 | Hypothetical protein |
| APH_0714 | Hypothetical protein |
| APH_0715 | Hypothetical protein |
| APH_0717 | Hypothetical protein |
| APH_0718 | Hypothetical protein |
| APH_0719 | Hypothetical protein |
| APH_0720 | Hypothetical protein |
| APH_0721 | Hypothetical protein |
| APH_0722 | Hypothetical protein |
| APH_0723 | Hypothetical protein |
| APH_0724 | Hypothetical protein |
| APH_0725 | Hypothetical protein |
| APH_0726 | Hypothetical protein |
| APH_0727 | Hypothetical protein |
| APH_0728 | Membrane protein, putative |
| APH_0733 | Hypothetical protein |
| APH_0739 | Hypothetical protein |
| APH_0741 | Hypothetical protein |
| APH_0742 | Hypothetical protein |
| APH_0743 | Hypothetical protein |
| APH_0744 | Hypothetical protein |
| APH_0750 | Hypothetical protein |
| APH_0751 | Hypothetical protein |
| APH_0752 | Hypothetical protein |
| APH_0753 | Hypothetical protein |
| APH_0756 | Hypothetical protein |
| APH_0757 | Hypothetical protein |
| APH_0758 | Hypothetical protein |
| APH_0760 | Hypothetical protein |
| APH_0762 | Hypothetical protein |
| APH_0763 | Hypothetical protein |
| APH_0764 | Hypothetical protein |
| APH_0771 | Hypothetical protein |
| APH_0772 | Hypothetical protein |
| APH_0773 | Hypothetical protein |
| APH_0774 | Hypothetical protein |
| APH_0778 | Hypothetical protein |
| APH_0779 | Hypothetical protein |
| APH_0781 | Hypothetical protein |
| APH_0785 | Hypothetical protein |
| APH_0786 | Hypothetical protein |
| APH_0787 | Hypothetical protein |
| APH_0788 | Hypothetical protein |
| APH_0789 | Hypothetical protein |
| APH_0793 | Hypothetical protein |
| APH_0797 | Hypothetical protein |
| APH_0798 | Hypothetical protein |
| APH_0799 | Hypothetical protein |
| APH_0800 | Hypothetical protein |
| APH_0804 | Hypothetical protein |
| APH_0805 | Hypothetical protein |
| APH_0806 | Hypothetical protein |
| APH_0807 | Hypothetical protein |
| APH_0808 | Hypothetical protein |
| APH_0809 | Hypothetical protein |
| APH_0810 | Hypothetical protein |
| APH_0811 | Hypothetical protein |
| APH_0812 | Hypothetical protein |
| APH_0815 | Hypothetical protein |
| APH_0816 | Hypothetical protein |
| APH_0817 | Hypothetical protein |
| APH_0818 | Hypothetical protein |
| APH_0819 | Hypothetical protein |
| APH_0820 | Hypothetical protein |
| APH_0821 | Hypothetical protein |
| APH_0822 | Hypothetical protein |
| APH_0823 | Hypothetical protein |
| APH_0824 | Hypothetical protein |
| APH_0825 | Hypothetical protein |
| APH_0826 | Hypothetical protein |
| APH_0827 | Hypothetical protein |
| APH_0828 | Hypothetical protein |
| APH_0829 | Hypothetical protein |
| APH_0830 | Hypothetical protein |
| APH_0831 | Hypothetical protein |
| APH_0832 | Hypothetical protein |
| APH_0833 | Hypothetical protein |
| APH_0834 | Hypothetical protein |
| APH_0835 | Hypothetical protein |
| APH_0836 | Hypothetical protein |
| APH_0837 | Hypothetical protein |
| APH_0838 | Hypothetical protein |
| APH_0839 | Hypothetical protein |
| APH_0840 | Hypothetical protein |
| APH_0841 | Hypothetical protein |
| APH_0842 | Hypothetical protein |
| APH_0843 | Hypothetical protein |
| APH_0844 | Hypothetical protein |
| APH_0845 | Hypothetical protein |
| APH_0846 | Hypothetical protein |
| APH_0847 | Hypothetical protein |
| APH_0850 | Hypothetical protein |
| APH_0853 | Hypothetical protein |
| APH_0854 | Hypothetical protein |
| APH_0855 | Hypothetical protein |
| APH_0858 | Hypothetical protein |
| APH_0862 | Hypothetical protein |
| APH_0864 | Hypothetical protein |
| APH_0866 | Hypothetical protein |
| APH_0868 | Hypothetical protein |
| APH_0869 | Hypothetical protein |
| APH_0870 | Hypothetical protein |
| APH_0871 | Hypothetical protein |
| APH_0872 | Hypothetical protein |
| APH_0873 | Hypothetical protein |
| APH_0875 | Hypothetical protein |
| APH_0877 | Hypothetical protein |
| APH_0879 | Hypothetical protein |
| APH_0881 | Hypothetical protein |
| APH_0882 | Hypothetical protein |
| APH_0885 | Hypothetical protein |
| APH_0886 | Hypothetical protein |
| APH_0887 | Hypothetical protein |
| APH_0888 | Hypothetical protein |
| APH_0890 | Hypothetical protein |
| APH_0891 | Hypothetical protein |
| APH_0892 | Hypothetical protein |
| APH_0898 | Hypothetical protein |
| APH_0900 | P44-57 outer membrane protein, silent |
| APH_0901 | Hypothetical protein |
| APH_0902 | P44-73 outer membrane protein, silent |
| APH_0904 | Hypothetical protein |
| APH_0905 | Hypothetical protein |
| APH_0906 | Hypothetical protein |
| APH_0908 | Hypothetical protein |
| APH_0912 | Hypothetical protein |
| APH_0913 | Hypothetical protein |
| APH_0914 | Hypothetical protein |
| APH_0915 | Hypothetical protein |
| APH_0916 | Hypothetical protein |
| APH_0917 | Hypothetical protein |
| APH_0918 | Hypothetical protein |
| APH_0919 | Membrane protein, putative |
| APH_0920 | Hypothetical protein |
| APH_0921 | Hypothetical protein |
| APH_0922 | Membrane protein, putative |
| APH_0926 | Hypothetical protein |
| APH_0927 | Hypothetical protein |
| APH_0931 | Hypothetical protein |
| APH_0934 | P44-34 outer membrane protein, silent |
| APH_0935 | P44-85 outer membrane protein, truncation |
| APH_0936 | P44-86 outer membrane protein, truncation |
| APH_0937 | Hypothetical protein |
| APH_0938 | Conserved domain protein |
| APH_0943 | Hypothetical protein |
| APH_0946 | Hypothetical protein |
| APH_0947 | Hypothetical protein |
| APH_0948 | Hypothetical protein |
| APH_0950 | Hypothetical protein |
| APH_0951 | P44 outer membrane protein, C-terminal fragment |
| APH_0952 | Hypothetical protein |
| APH_0953 | P44 outer membrane protein, C-terminal fragment |
| APH_0954 | Hypothetical protein |
| APH_0955.1 | Hypothetical protein |
| APH_0958 | Hypothetical protein |
| APH_0959 | Hypothetical protein |
| APH_0963 | Hypothetical protein |
| APH_0964 | Collagen triple helix repeat protein |
| APH_0967 | Hypothetical protein |
| APH_0978 | Hypothetical protein |
| APH_0980 | Major surface protein |
| APH_0981 | P44-44 outer membrane protein, silent |
| APH_0991 | Hypothetical protein |
| APH_0992 | Hypothetical protein |
| APH_0996 | Hypothetical protein |
| APH_1002 | Hypothetical protein |
| APH_1005 | Hypothetical protein |
| APH_1006 | Hypothetical protein |
| APH_1007 | Hypothetical protein |
| APH_1009 | Hypothetical protein |
| APH_1011 | Hypothetical protein |
| APH_1017 | Major outer membrane protein, P44 family |
| APH_1018 | P44-62 outer membrane protein, silent |
| APH_1019 | Hypothetical protein |
| APH_1038 | Hypothetical protein |
| APH_1039 | Hypothetical protein |
| APH_1041 | Hypothetical protein |
| APH_1044 | Hypothetical protein |
| APH_1046 | Hypothetical protein |
| APH_1047 | Hypothetical protein |
| APH_1054 | Hypothetical protein |
| APH_1055 | Hypothetical protein |
| APH_1057 | Hypothetical protein |
| APH_1063 | P44-38 outer membrane protein, silent |
| APH_1064 | P44-42 outer membrane protein, silent |
| APH_1065 | P44-74 outer membrane protein, silent |
| APH_1072 | Hypothetical protein |
| APH_1076 | Major outer membrane protein, P44 family |
| APH_1077 | P44-41 outer membrane protein, silent |
| APH_1078 | P44-56 outer membrane protein, silent |
| APH_1080 | P44-50 outer membrane protein, silent |
| APH_1081 | P44-28 outer membrane protein, silent |
| APH_1082 | P44-52 outer membrane protein, silent |
| APH_1083 | Hypothetical protein |
| APH_1089 | Hypothetical protein |
| APH_1091 | Hypothetical protein |
| APH_1092 | Hypothetical protein |
| APH_1094 | Hypothetical protein |
| APH_1094.1 | Peptidase domain protein |
| APH_1104 | Hypothetical protein |
| APH_1105 | Hypothetical protein |
| APH_1106 | Hypothetical protein |
| APH_1109 | Hypothetical protein |
| APH_1114 | P44-7 outer membrane protein |
| APH_1115 | P44 outer membrane protein, C-terminal fragment |
| APH_1116 | Hypothetical protein |
| APH_1118 | P44-7b outer membrane protein, silent |
| APH_1121 | P44-6 outer membrane protein, silent |
| APH_1122 | P44-75 outer membrane protein, silent |
| APH_1123 | P44-3 outer membrane protein, silent |
| APH_1124 | P44 outer membrane protein, C-terminal fragment |
| APH_1125 | Human granulocytic ehrlichiosis antigen |
| APH_1126 | Hypothetical protein |
| APH_1127 | Hypothetical protein |
| APH_1128 | P44-76 outer membrane protein, silent |
| APH_1131 | Conserved hypothetical protein |
| APH_1132 | Conserved hypothetical protein |
| APH_1133 | Conserved hypothetical protein |
| APH_1134 | Conserved hypothetical protein |
| APH_1135 | Hypothetical protein |
| APH_1137 | Hypothetical protein |
| APH_1138 | Hypothetical protein |
| APH_1139 | Hypothetical protein |
| APH_1140 | Hypothetical protein |
| APH_1141 | Hypothetical protein |
| APH_1142 | Hypothetical protein |
| APH_1143 | Hypothetical protein |
| APH_1145 | Hypothetical protein |
| APH_1151 | Hypothetical protein |
| APH_1152 | P44-47 outer membrane protein, silent |
| APH_1154 | P44-4 outer membrane protein, silent |
| APH_1155 | P44-53 outer membrane protein |
| APH_1156 | Hypothetical protein |
| APH_1157 | Hypothetical protein |
| APH_1163 | P44-53b outer membrane protein, silent |
| APH_1167 | Hypothetical protein |
| APH_1168 | P44-36 outer membrane protein |
| APH_1169 | P44-19 outer membrane protein, silent |
| APH_1172 | P44 outer membrane protein, C-terminal fragment |
| APH_1176 | P44-2 outer membrane protein |
| APH_1177 | P44-54 outer membrane protein, silent |
| APH_1184 | P44-43 outer membrane protein |
| APH_1185 | P44 outer membrane protein, C-terminal fragment |
| APH_1186 | P44-40 outer membrane protein, silent |
| APH_1187 | Hypothetical protein |
| APH_1188 | Hypothetical protein |
| APH_1194 | P44-18 outer membrane protein, silent |
| APH_1195 | P44-1 outer membrane protein |
| APH_1196 | Hypothetical protein |
| APH_1201 | Hypothetical protein |
| APH_1202 | P44-77 outer membrane protein, silent |
| APH_1203 | P44-34b outer membrane protein, silent |
| APH_1215 | P44-14 outer membrane protein |
| APH_1220 | Omp-1N |
| APH_1221 | P44-18ES, P44 outer membrane protein expression locus with P44-18 |
| APH_1222 | Hypothetical protein |
| APH_1225 | Hypothetical protein |
| APH_1226 | P44-15 outer membrane protein, silent |
| APH_1229 | P44-2b outer membrane protein |
| APH_1230 | P44 outer membrane protein, C-terminal fragment |
| APH_1234 | Hypothetical protein |
| APH_1236 | Hypothetical protein |
| APH_1237 | Hypothetical protein |
| APH_1238 | P44-13 outer membrane protein, silent |
| APH_1239 | P44-15b outer membrane protein |
| APH_1241 | P44-33 outer membrane protein, silent |
| APH_1242 | P44-49 outer membrane protein, silent |
| APH_1248 | P44-27 outer membrane protein, silent |
| APH_1249 | P44-12 outer membrane protein |
| APH_1250 | Major surface protein hypervariable region, authentic point mutation |
| APH_1251 | Hypothetical protein |
| APH_1253 | P44-39 outer membrane protein, silent |
| APH_1254 | Hypothetical protein |
| APH_1255 | P44-10 outer membrane protein, silent |
| APH_1256 | P44-23 outer membrane protein |
| APH_1260 | Hypothetical protein |
| APH_1261 | P44-46 outer membrane protein, silent |
| APH_1262 | Hypothetical protein |
| APH_1269 | P44-16 outer membrane protein |
| APH_1272 | P44-48 outer membrane protein, silent, authentic point mutation |
| APH_1273 | Major outer membrane protein, P44 family |
| APH_1274 | P44-25 outer membrane protein, silent |
| APH_1275 | P44-16b outer membrane protein |
| APH_1278 | Hypothetical protein |
| APH_1284 | P44 outer membrane protein, C-terminal fragment |
| APH_1286 | Hypothetical protein |
| APH_1287 | P44-32 outer membrane protein |
| APH_1293 | Hypothetical protein |
| APH_1294 | Conserved domain protein |
| APH_1294.1 | Hypothetical protein |
| APH_1296 | Hypothetical protein |
| APH_1304 | Hypothetical protein |
| APH_1311 | P44-24 outer membrane protein |
| APH_1312 | P44-5 outer membrane protein, silent |
| APH_1313 | P44-26 outer membrane protein, silent |
| APH_1317 | Hypothetical protein |
| APH_1318 | Hypothetical protein |
| APH_1324 | P44 outer membrane protein, N-terminal fragment |
| APH_1325 | P44-78 outer membrane protein |
| APH_1327 | Hypothetical protein |
| APH_1328 | P44-17 outermembrane protein, silent |
| APH_1329 | P44-60 outer membrane protein, silent |
| APH_1330 | P44-29 outer membrane protein, silent |
| APH_1336 | Hypothetical protein |
| APH_1338 | Hypothetical protein |
| APH_1342 | P44-31 outer membrane protein, silent |
| APH_1343 | P44-58 outer membrane protein, silent |
| APH_1350 | P44-51 outer membrane protein, silent |
| APH_1351 | P44-35 outer membrane protein, silent |
| APH_1352 | P44-30 outer membrane protein, silent |
| APH_1353 | Hypothetical protein |
| APH_1355 | P44-79 outer membrane protein |
| APH_1356 | Hypothetical protein |
| APH_1356.1 | Hypothetical protein |
| APH_1358 | outer membrane protein-1B |
| APH_1359 | Major outer membrane protein OMP-1A, putative |
| APH_1361 | Major surface protein 2 |
| APH_1362 | Hypothetical protein |
| APH_1363 | Hypothetical protein |
| APH_1364 | Hypothetical protein |
| APH_1368 | Hypothetical protein |
| APH_1369 | Hypothetical protein |
| APH_1370 | Hypothetical protein |
| APH_1379 | Hypothetical protein |
| APH_1380 | Hypothetical protein |
| APH_1381 | Hypothetical protein |
| APH_1382 | Hypothetical protein |
| APH_1383 | Hypothetical protein |
| APH_1384 | Hypothetical protein |
| APH_1385 | Hypothetical protein |
| APH_1386 | Hypothetical protein |
| APH_1387 | HGE-2 protein |
| APH_1388 | Hypothetical protein |
| APH_1389 | P44 outer membrane protein, C-terminal fragment |
| APH_1390 | P44-20 outer membrane protein, silent |
| APH_1391 | P44-9 outer membrane protein, silent |
| APH_1392 | P44-11 outer membrane protein |
| APH_1393 | Hypothetical protein |
| APH_1399 | P44 outer membrane protein, C-terminal fragment |
| APH_1400 | P44-37 outer membrane protein, silent |
| APH_1401 | P44-22 outer membrane protein, silent |
| APH_1407 | Hypothetical protein |
| APH_1408 | P44-59 outer membrane protein, silent |
| APH_1410 | Hypothetical protein |
| APH_1411 | Hypothetical protein |
| APH_1412 | Hypothetical protein |
