# Supplementary material for: Comparative Genomics of Emerging Human Ehrlichiosis Agents
Source: PLoS Genet. 2006 Feb 17;2(2):e21. doi: 10.1371/journal.pgen.0020021 (PMC1366493; doi:10.1371/journal.pgen.0020021)
Supplement: Supplementary file 15 [file pgen.0020021.st010.doc]

**Supplementary Table 10.** Individual genes (based on ortholog cluster analysis, see methods) present in *Ehrlichia chaffeensis* but not in *Anaplasma phagocytophilum,* *Rickettsia prowazekii*, *Wolbachia pipientis*, or *Neorickettsia sennetsu.*

| Locus | Common Name |
| --- | --- |
| ECH_0013 | Pyrroline-5-carboxylate reductase, putative |
| ECH_0017 | Hypothetical protein |
| ECH_0034 | Hypothetical protein |
| ECH_0047 | Hypothetical protein |
| ECH_0049 | Hypothetical protein |
| ECH_0051 | Hypothetical protein |
| ECH_0053 | Hypothetical protein |
| ECH_0054 | Hypothetical protein |
| ECH_0059 | Hypothetical protein |
| ECH_0068 | Hypothetical protein |
| ECH_0070 | Hypothetical protein |
| ECH_0075 | Hypothetical protein |
| ECH_0078 | Hypothetical protein |
| ECH_0079 | Hypothetical protein |
| ECH_0081 | Hypothetical protein |
| ECH_0083 | Hypothetical protein |
| ECH_0086 | Hypothetical protein |
| ECH_0087 | Hypothetical protein |
| ECH_0094 | Hypothetical protein |
| ECH_0099 | Hypothetical protein |
| ECH_0100 | Hypothetical protein |
| ECH_0101 | Hypothetical protein |
| ECH_0102 | Hypothetical protein |
| ECH_0103 | Hypothetical protein |
| ECH_0104 | Hypothetical protein |
| ECH_0105 | Hypothetical protein |
| ECH_0106 | Hypothetical protein |
| ECH_0107 | Hypothetical protein |
| ECH_0108 | Hypothetical protein |
| ECH_0109 | Hypothetical protein |
| ECH_0110 | Hypothetical protein |
| ECH_0111 | Hypothetical protein |
| ECH_0112 | Hypothetical protein |
| ECH_0113 | Hypothetical protein |
| ECH_0114 | Hypothetical protein |
| ECH_0115 | Hypothetical protein |
| ECH_0116 | Hypothetical protein |
| ECH_0117 | Hypothetical protein |
| ECH_0118 | Hypothetical protein |
| ECH_0119 | Hypothetical protein |
| ECH_0120 | Hypothetical protein |
| ECH_0121 | Hypothetical protein |
| ECH_0145 | Hypothetical protein |
| ECH_0150 | Hypothetical protein |
| ECH_0153 | Hypothetical protein |
| ECH_0155 | GTP-binding protein, putative |
| ECH_0158 | Hypothetical protein |
| ECH_0159 | Hypothetical protein |
| ECH_0170 | Variable length PCR target protein |
| ECH_0177 | Hypothetical protein |
| ECH_0180 | Hypothetical protein |
| ECH_0181 | Hypothetical protein |
| ECH_0182 | Hypothetical protein |
| ECH_0190 | Hypothetical protein |
| ECH_0191 | Hypothetical protein |
| ECH_0205 | Hypothetical protein |
| ECH_0215 | Hypothetical protein |
| ECH_0216 | Hypothetical protein |
| ECH_0228 | Hypothetical protein |
| ECH_0231 | Hypothetical protein |
| ECH_0236 | Hypothetical protein |
| ECH_0238 | Hypothetical protein |
| ECH_0240 | Hypothetical protein |
| ECH_0241 | Hypothetical protein |
| ECH_0242 | Hypothetical protein |
| ECH_0243 | Hypothetical protein |
| ECH_0244 | Hypothetical protein |
| ECH_0245 | Hypothetical protein |
| ECH_0246 | Hypothetical protein |
| ECH_0248 | Hypothetical protein |
| ECH_0249 | Hypothetical protein |
| ECH_0251 | Hypothetical protein |
| ECH_0252 | Hypothetical protein |
| ECH_0253 | Hypothetical protein |
| ECH_0254 | Hypothetical protein |
| ECH_0255 | Hypothetical protein |
| ECH_0256 | Hypothetical protein |
| ECH_0257 | Hypothetical protein |
| ECH_0258 | Hypothetical protein |
| ECH_0259 | Hypothetical protein |
| ECH_0260 | Hypothetical protein |
| ECH_0261 | Hypothetical protein |
| ECH_0262 | Hypothetical protein |
| ECH_0265 | Hypothetical protein |
| ECH_0268 | Hypothetical protein |
| ECH_0270 | Hypothetical protein |
| ECH_0271 | Hypothetical protein |
| ECH_0272 | Hypothetical protein |
| ECH_0273 | Hypothetical protein |
| ECH_0274 | Hypothetical protein |
| ECH_0275 | Conserved hypothetical protein |
| ECH_0276 | Hypothetical protein |
| ECH_0278 | Hypothetical protein |
| ECH_0279 | Hypothetical protein |
| ECH_0280 | Hypothetical protein |
| ECH_0281 | Hypothetical protein |
| ECH_0282 | Hypothetical protein |
| ECH_0283 | Hypothetical protein |
| ECH_0284 | Hypothetical protein |
| ECH_0285 | Hypothetical protein |
| ECH_0286 | Hypothetical protein |
| ECH_0287 | Hypothetical protein |
| ECH_0288 | Hypothetical protein |
| ECH_0289 | Hypothetical protein |
| ECH_0292 | Conserved domain protein |
| ECH_0314 | Hypothetical protein |
| ECH_0323 | Hypothetical protein |
| ECH_0329 | Hypothetical protein |
| ECH_0338 | Hypothetical protein |
| ECH_0344 | Hypothetical protein |
| ECH_0349 | Hypothetical protein |
| ECH_0357 | Hypothetical protein |
| ECH_0358 | Hypothetical protein |
| ECH_0360 | Hypothetical protein |
| ECH_0361 | Hypothetical protein |
| ECH_0372 | Hypothetical protein |
| ECH_0375 | Hypothetical protein |
| ECH_0379 | Hypothetical protein |
| ECH_0380 | Hypothetical protein |
| ECH_0384 | Hypothetical protein |
| ECH_0386 | Hypothetical protein |
| ECH_0388 | Hypothetical protein |
| ECH_0393 | Hypothetical protein |
| ECH_0394 | Hypothetical protein |
| ECH_0436 | Hypothetical protein |
| ECH_0437 | Hypothetical protein |
| ECH_0453 | Hypothetical protein |
| ECH_0456 | Hypothetical protein |
| ECH_0457 | Hypothetical protein |
| ECH_0463 | Hypothetical protein |
| ECH_0468 | Monovalent cation/proton antiporter subunit, putative, authentic frameshift |
| ECH_0472 | Hypothetical protein |
| ECH_0481 | Hypothetical protein |
| ECH_0485 | Diaminopimelate decarboxylase |
| ECH_0506 | Hypothetical protein |
| ECH_0507 | Hypothetical protein |
| ECH_0513 | Hypothetical protein |
| ECH_0518 | Membrane protein, putative |
| ECH_0519 | Hypothetical protein |
| ECH_0524 | Hypothetical protein |
| ECH_0527 | DNA polymerase III, epsilon subunit, authentic frameshift |
| ECH_0530 | Hypothetical protein |
| ECH_0531 | Hypothetical protein |
| ECH_0549 | Hypothetical protein |
| ECH_0551 | Hypothetical protein |
| ECH_0565 | Hypothetical protein |
| ECH_0568 | Phage minor structural protein, N-terminal domain protein |
| ECH_0569 | Hypothetical protein |
| ECH_0570 | Hypothetical protein |
| ECH_0578 | Hypothetical protein |
| ECH_0580 | Hypothetical protein |
| ECH_0582 | Hypothetical protein |
| ECH_0583 | Hypothetical protein |
| ECH_0586 | Hypothetical protein |
| ECH_0589 | Hypothetical protein |
| ECH_0590 | Hypothetical protein |
| ECH_0596 | Hypothetical protein |
| ECH_0598 | Hypothetical protein |
| ECH_0600 | Hypothetical protein |
| ECH_0601 | Hypothetical protein |
| ECH_0603 | Hypothetical protein |
| ECH_0607 | Hypothetical protein |
| ECH_0608 | Hypothetical protein |
| ECH_0609 | Hypothetical protein |
| ECH_0610 | Hypothetical protein |
| ECH_0611 | Hypothetical protein |
| ECH_0612 | Hypothetical protein |
| ECH_0613 | Conserved domain protein |
| ECH_0614 | Hypothetical protein |
| ECH_0640 | Conserved domain protein |
| ECH_0658 | Hypothetical protein |
| ECH_0668 | Hypothetical protein |
| ECH_0670 | Membrane protein, putative |
| ECH_0672 | Hypothetical protein |
| ECH_0673 | Membrane protein, putative |
| ECH_0674 | Hypothetical protein |
| ECH_0676 | Arginine biosynthesis bifunctional protein ArgJ |
| ECH_0677 | Hypothetical protein |
| ECH_0680 | Argininosuccinate synthase |
| ECH_0681 | Hypothetical protein |
| ECH_0682 | Hypothetical protein |
| ECH_0685 | Hypothetical protein |
| ECH_0686 | Deoxyguanosinetriphosphate triphosphohydrolase, putative, truncation |
| ECH_0694 | Hypothetical protein |
| ECH_0695 | Hypothetical protein |
| ECH_0696 | Hypothetical protein |
| ECH_0697 | Hypothetical protein |
| ECH_0698 | Hypothetical protein |
| ECH_0699 | Hypothetical protein |
| ECH_0700 | Hypothetical protein |
| ECH_0704 | Conserved hypothetical protein |
| ECH_0707 | Hypothetical protein |
| ECH_0710 | Hypothetical protein |
| ECH_0711 | Hypothetical protein |
| ECH_0714 | Hypothetical protein |
| ECH_0715 | Hypothetical protein |
| ECH_0716 | Hypothetical protein |
| ECH_0717 | Hypothetical protein |
| ECH_0718 | Hypothetical protein |
| ECH_0719 | Hypothetical protein |
| ECH_0720 | Hypothetical protein |
| ECH_0721 | Lipoprotein, putative |
| ECH_0722 | Hypothetical protein |
| ECH_0723 | Hypothetical protein |
| ECH_0733 | Hypothetical protein |
| ECH_0736 | Hypothetical protein |
| ECH_0745 | Conserved domain protein |
| ECH_0748 | Hypothetical protein |
| ECH_0749 | Hypothetical protein |
| ECH_0752 | Hypothetical protein |
| ECH_0767 | Conserved hypothetical protein |
| ECH_0770 | Hypothetical protein |
| ECH_0775 | Hypothetical protein |
| ECH_0810 | Hypothetical protein |
| ECH_0816 | Major facilitator family transporter |
| ECH_0817 | Hypothetical protein |
| ECH_0819 | Hypothetical protein |
| ECH_0823 | Hypothetical protein |
| ECH_0827 | Hypothetical protein |
| ECH_0829 | Hypothetical protein |
| ECH_0831 | Conserved domain protein |
| ECH_0833 | Hypothetical protein |
| ECH_0834 | Hypothetical protein |
| ECH_0839 | Hypothetical protein |
| ECH_0851 | Hypothetical protein |
| ECH_0855 | Hypothetical protein |
| ECH_0859 | Hypothetical protein |
| ECH_0861 | Hypothetical protein |
| ECH_0863 | Hypothetical protein |
| ECH_0864 | Conserved domain protein |
| ECH_0865 | Hypothetical protein |
| ECH_0866 | Hypothetical protein |
| ECH_0867 | Hypothetical protein |
| ECH_0868 | Hypothetical protein |
| ECH_0869 | Hypothetical protein |
| ECH_0870 | Hypothetical protein |
| ECH_0878 | Hypothetical protein |
| ECH_0879 | Hypothetical protein |
| ECH_0880 | Hypothetical protein |
| ECH_0887 | Hypothetical protein |
| ECH_0889 | Hypothetical protein |
| ECH_0894 | Conserved domain protein |
| ECH_0909 | Hypothetical protein |
| ECH_0915 | Hypothetical protein |
| ECH_0921 | Hypothetical protein |
| ECH_0923 | Hypothetical protein |
| ECH_0924 | Hypothetical protein |
| ECH_0925 | Hypothetical protein |
| ECH_0926 | Hypothetical protein |
| ECH_0927 | Hypothetical protein |
| ECH_0928 | Hypothetical protein |
| ECH_0929 | Lipoprotein, putative |
| ECH_0932 | Hypothetical protein |
| ECH_0933 | Hypothetical protein |
| ECH_0934 | Patatin-like phospholipase, authentic frameshift |
| ECH_0936 | Hypothetical protein |
| ECH_0938 | Hypothetical protein |
| ECH_0942 | Hypothetical protein |
| ECH_0943 | Hypothetical protein |
| ECH_0945 | Conserved domain protein |
| ECH_0949 | Hypothetical protein |
| ECH_0965 | Hypothetical protein |
| ECH_0968 | Hypothetical protein |
| ECH_0969 | Hypothetical protein |
| ECH_0971 | Hypothetical protein |
| ECH_0975 | Hypothetical protein |
| ECH_0984 | Hypothetical protein |
| ECH_0990 | Hypothetical protein |
| ECH_0993 | Hypothetical protein |
| ECH_0995 | Hypothetical protein |
| ECH_0999 | Hypothetical protein |
| ECH_1007 | Seryl-tRNA synthetase, authentic frameshift |
| ECH_1015 | Hypothetical protein |
| ECH_1016 | Hypothetical protein |
| ECH_1022 | Hypothetical protein |
| ECH_1026 | Hypothetical protein |
| ECH_1028 | Hypothetical protein |
| ECH_1029 | Hypothetical protein |
| ECH_1032 | Hypothetical protein |
| ECH_1035 | Conserved hypothetical protein, degenerate |
| ECH_1037 | Hypothetical protein |
| ECH_1039 | Hypothetical protein |
| ECH_1040 | Conserved domain protein |
| ECH_1045 | Hypothetical protein |
| ECH_1046 | Hypothetical protein |
| ECH_1048 | Hypothetical protein |
| ECH_1049 | Hypothetical protein |
| ECH_1056 | Hypothetical protein |
| ECH_1059 | Conserved hypothetical protein |
| ECH_1075 | Hypothetical protein |
| ECH_1076 | Hypothetical protein |
| ECH_1077 | Hypothetical protein |
| ECH_1080 | Hypothetical protein |
| ECH_1082 | Hypothetical protein |
| ECH_1085 | Hypothetical protein |
| ECH_1091 | Hypothetical protein |
| ECH_1092 | Hypothetical protein |
| ECH_1094 | Hypothetical protein |
| ECH_1095 | Hypothetical protein |
| ECH_1099 | Hypothetical protein |
| ECH_1100 | Hypothetical protein |
| ECH_1102 | Hypothetical protein |
| ECH_1104 | Hypothetical protein |
| ECH_1112 | Hypothetical protein |
| ECH_1113 | Hypothetical protein |
| ECH_1120 | Hypothetical protein |
| ECH_1121 | Major outer membrane protein Omp-1N |
| ECH_1122 | Conserved hypothetical protein |
| ECH_1123 | Major outer membrane protein OMP-1Q |
| ECH_1124 | Major outer membrane protein OMP-1P |
| ECH_1125 | Major outer membrane protein OMP-1T |
| ECH_1126 | Major outer membrane protein OMP-1U |
| ECH_1128 | Conserved hypothetical protein |
| ECH_1129 | Major outer membrane protein OMP-1W |
| ECH_1130 | Major outer membrane protein OMP-1X |
| ECH_1131 | Major outer membrane protein OMP-1Y |
| ECH_1132 | Major outer membrane protein OMP-1S |
| ECH_1134 | Major outer membrane protein OMP-1Z |
| ECH_1138 | Hypothetical protein |
| ECH_1139 | Major outer membrane protein OMP-1D |
| ECH_1141 | Hypothetical protein |
| ECH_1142 | Major outer membrane protein OMP-1F |
| ECH_1144 | Major outer membrane protein P28-1 |
| ECH_1145 | Hypothetical protein |
| ECH_1146 | Major outer membrane protein P28-2 |
| ECH_1147 | Conserved hypothetical protein |
| ECH_1148 | Conserved hypothetical protein |
| ECH_1150 | Hypothetical protein |
| ECH_1151 | Hypothetical protein |
| ECH_1152 | Hypothetical protein |
