# Supplementary material for: Comparative Genomics of Emerging Human Ehrlichiosis Agents
Source: PLoS Genet. 2006 Feb 17;2(2):e21. doi: 10.1371/journal.pgen.0020021 (PMC1366493; doi:10.1371/journal.pgen.0020021)
Supplement: Supplementary file 16 [file pgen.0020021.st011.doc]

**Supplementary Table 11.** Individual genes (based on ortholog cluster analysis, see methods) present in *Neorickettsia sennetsu* but not in *Anaplasma phagocytophilum,* *Rickettsia prowazekii*, *Wolbachia pipientis*, or *Ehrlichia chaffeensis*.

| Locus | Common name |
| --- | --- |
| NSE_0002 | Hypothetical protein |
| NSE_0004 | Hypothetical protein |
| NSE_0008 | Hypothetical protein |
| NSE_0011 | Hypothetical protein |
| NSE_0013 | Hypothetical protein |
| NSE_0017 | Hypothetical protein |
| NSE_0018 | Hypothetical protein |
| NSE_0021 | Hypothetical protein |
| NSE_0022 | Hypothetical protein |
| NSE_0023 | Hypothetical protein |
| NSE_0024 | Hypothetical protein |
| NSE_0025 | Hypothetical protein |
| NSE_0026 | Hypothetical protein |
| NSE_0027 | Hypothetical protein |
| NSE_0028 | Hypothetical protein |
| NSE_0029 | Membrane protein, putative |
| NSE_0030 | Hypothetical protein |
| NSE_0033 | Hypothetical protein |
| NSE_0034 | Monovalent cation/proton antiporter, putative authentic frameshift |
| NSE_0037 | Hypothetical protein |
| NSE_0041 | Hypothetical protein |
| NSE_0051 | Hypothetical protein |
| NSE_0069 | Hypothetical protein |
| NSE_0070 | Hypothetical protein |
| NSE_0071 | Hypothetical protein |
| NSE_0072 | Hypothetical protein |
| NSE_0073 | Hypothetical protein |
| NSE_0074 | Hypothetical protein |
| NSE_0075 | Hypothetical protein |
| NSE_0076 | Hypothetical protein |
| NSE_0077 | Conserved domain protein |
| NSE_0078 | Hypothetical protein |
| NSE_0079 | Hypothetical protein |
| NSE_0080 | Hypothetical protein |
| NSE_0081 | Hypothetical protein |
| NSE_0082 | Hypothetical protein |
| NSE_0083 | Hypothetical protein |
| NSE_0086 | Hypothetical protein |
| NSE_0087 | Conserved domain protein |
| NSE_0088 | Hypothetical protein |
| NSE_0089 | Hypothetical protein |
| NSE_0090 | Hypothetical protein |
| NSE_0091 | Hypothetical protein |
| NSE_0092 | Hypothetical protein |
| NSE_0093 | Hypothetical protein |
| NSE_0096 | Hypothetical protein |
| NSE_0098 | Hypothetical protein |
| NSE_0099 | Hypothetical protein |
| NSE_0102 | Hypothetical protein |
| NSE_0103 | Hypothetical protein |
| NSE_0104 | Hypothetical protein |
| NSE_0105 | Hypothetical protein |
| NSE_0106 | Hypothetical protein |
| NSE_0107 | Hypothetical protein |
| NSE_0108 | Hypothetical protein |
| NSE_0109 | Hypothetical protein |
| NSE_0110 | Hypothetical protein |
| NSE_0111 | Hypothetical protein |
| NSE_0112 | Hypothetical protein |
| NSE_0113 | Hypothetical protein |
| NSE_0114 | Hypothetical protein |
| NSE_0115 | Hypothetical protein |
| NSE_0116 | Hypothetical protein |
| NSE_0117 | Hypothetical protein |
| NSE_0118 | Hypothetical protein |
| NSE_0129 | Hypothetical protein |
| NSE_0130 | Hypothetical protein |
| NSE_0135 | Hypothetical protein |
| NSE_0139 | Hypothetical protein |
| NSE_0140 | Hypothetical protein |
| NSE_0141 | Hypothetical protein |
| NSE_0143 | Hypothetical protein |
| NSE_0145 | Hypothetical protein |
| NSE_0147 | Hypothetical protein |
| NSE_0148 | Hypothetical protein |
| NSE_0149 | Hypothetical protein |
| NSE_0152 | Hypothetical protein |
| NSE_0155 | Hypothetical protein |
| NSE_0157 | Hypothetical protein |
| NSE_0158 | Transporter, putative |
| NSE_0159 | Hypothetical protein |
| NSE_0174 | Hypothetical protein |
| NSE_0182 | Hypothetical protein |
| NSE_0184 | Hypothetical protein |
| NSE_0187 | Hypothetical protein |
| NSE_0188 | Hypothetical protein |
| NSE_0193 | Hypothetical protein |
| NSE_0195 | Hypothetical protein |
| NSE_0200 | Hypothetical protein |
| NSE_0207 | Hypothetical protein |
| NSE_0208 | Hypothetical protein |
| NSE_0219 | Hypothetical protein |
| NSE_0221 | Hypothetical protein |
| NSE_0227 | Thioredoxin |
| NSE_0231 | Hypothetical protein |
| NSE_0233 | Hypothetical protein |
| NSE_0239 | Hypothetical protein |
| NSE_0240 | Membrane protein, putative |
| NSE_0241 | Conserved hypothetical protein |
| NSE_0243 | Hypothetical protein |
| NSE_0244 | Hypothetical protein |
| NSE_0261 | Hypothetical protein |
| NSE_0274 | Ribosomal protein L29 |
| NSE_0296 | Hypothetical protein |
| NSE_0300 | Hypothetical protein |
| NSE_0314 | Hypothetical protein |
| NSE_0316 | Hypothetical protein |
| NSE_0326 | Hypothetical protein |
| NSE_0328 | Hypothetical protein |
| NSE_0329 | Hypothetical protein |
| NSE_0332 | Hypothetical protein |
| NSE_0334 | Hypothetical protein |
| NSE_0338 | Hypothetical protein |
| NSE_0344 | Hypothetical protein |
| NSE_0348 | Conserved hypothetical protein |
| NSE_0351 | Hypothetical protein |
| NSE_0353 | Hypothetical protein |
| NSE_0354 | Hypothetical protein |
| NSE_0355 | Hypothetical protein |
| NSE_0358 | Hypothetical protein |
| NSE_0361 | Lipoprotein, putative |
| NSE_0363 | Hypothetical protein |
| NSE_0364 | Hypothetical protein |
| NSE_0365 | Hypothetical protein |
| NSE_0368 | Hypothetical protein |
| NSE_0370 | Hypothetical protein |
| NSE_0372 | Hypothetical protein |
| NSE_0375 | Hypothetical protein |
| NSE_0378 | Hypothetical protein |
| NSE_0379 | Hypothetical protein |
| NSE_0381 | Hypothetical protein |
| NSE_0382 | Hypothetical protein |
| NSE_0387 | Hypothetical protein |
| NSE_0390 | Hypothetical protein |
| NSE_0399 | ATPase F0, B, chain putative |
| NSE_0400 | Hypothetical protein |
| NSE_0403 | Hypothetical protein |
| NSE_0405 | Hypothetical protein |
| NSE_0414 | Hypothetical protein |
| NSE_0418 | Hypothetical protein |
| NSE_0419 | Hypothetical protein |
| NSE_0421 | Hypothetical protein |
| NSE_0424 | Hypothetical protein |
| NSE_0425 | Hypothetical protein |
| NSE_0428 | Hypothetical protein |
| NSE_0430 | Hypothetical protein |
| NSE_0435 | Hypothetical protein |
| NSE_0437 | Hypothetical protein |
| NSE_0439 | Hypothetical protein |
| NSE_0440 | Hypothetical protein |
| NSE_0445 | Hypothetical protein |
| NSE_0449 | Hypothetical protein |
| NSE_0450 | Hypothetical protein |
| NSE_0451 | Hypothetical protein |
| NSE_0456 | Hypothetical protein |
| NSE_0457 | Hypothetical protein |
| NSE_0459 | Hypothetical protein |
| NSE_0482 | Hypothetical protein |
| NSE_0484 | Hypothetical protein |
| NSE_0487 | Hypothetical protein |
| NSE_0489 | Conserved hypothetical protein |
| NSE_0491 | Hypothetical protein |
| NSE_0494 | Hypothetical protein |
| NSE_0504 | Hypothetical protein |
| NSE_0511 | Hypothetical protein |
| NSE_0513 | Conserved hypothetical protein |
| NSE_0515 | Hypothetical protein |
| NSE_0517 | Lipoprotein, putative |
| NSE_0526 | Hypothetical protein |
| NSE_0531 | Sensor histidine kinase |
| NSE_0533 | Hypothetical protein |
| NSE_0539 | Hypothetical protein |
| NSE_0541 | Membrane protein, putative |
| NSE_0550 | Hypothetical protein |
| NSE_0555 | Hypothetical protein |
| NSE_0557 | Hypothetical protein |
| NSE_0562 | Hypothetical protein |
| NSE_0565 | Hypothetical protein |
| NSE_0568 | Hypothetical protein |
| NSE_0576 | Hypothetical protein |
| NSE_0577 | Hypothetical protein |
| NSE_0584 | Hypothetical protein |
| NSE_0586 | Hypothetical protein |
| NSE_0589 | Hypothetical protein |
| NSE_0593 | Hypothetical protein |
| NSE_0595 | Hypothetical protein |
| NSE_0596 | Hypothetical protein |
| NSE_0597 | Hypothetical protein |
| NSE_0599 | Hypothetical protein |
| NSE_0602 | Hypothetical protein |
| NSE_0605 | Hypothetical protein |
| NSE_0606 | Hypothetical protein |
| NSE_0608 | Hypothetical protein |
| NSE_0610 | Hypothetical protein |
| NSE_0611 | Hypothetical protein |
| NSE_0614 | Hypothetical protein |
| NSE_0616 | Hypothetical protein |
| NSE_0620 | Hypothetical protein |
| NSE_0631 | Hypothetical protein |
| NSE_0634 | Hypothetical protein |
| NSE_0639 | Hypothetical protein |
| NSE_0644 | Hypothetical protein |
| NSE_0648 | Hypothetical protein |
| NSE_0651 | Lipoprotein, putative |
| NSE_0652 | Hypothetical protein |
| NSE_0653 | Hypothetical protein |
| NSE_0654 | Hypothetical protein |
| NSE_0655 | Hypothetical protein |
| NSE_0656 | Hypothetical protein |
| NSE_0657 | Hypothetical protein |
| NSE_0659 | Hypothetical protein |
| NSE_0660 | Hypothetical protein |
| NSE_0662 | Hypothetical protein |
| NSE_0666 | Hypothetical protein |
| NSE_0670 | Hypothetical protein |
| NSE_0672 | Hypothetical protein |
| NSE_0674 | Hypothetical protein |
| NSE_0702 | Hypothetical protein |
| NSE_0703 | Hypothetical protein |
| NSE_0705 | Hypothetical protein |
| NSE_0708 | Hypothetical protein |
| NSE_0713 | Hypothetical protein |
| NSE_0716 | Hypothetical protein |
| NSE_0722 | Hypothetical protein |
| NSE_0727 | Hypothetical protein |
| NSE_0728 | Hypothetical protein |
| NSE_0729 | Hypothetical protein |
| NSE_0731 | Hypothetical protein |
| NSE_0732 | Hypothetical protein |
| NSE_0736 | Hypothetical protein |
| NSE_0744 | Hypothetical protein |
| NSE_0747 | Hypothetical protein |
| NSE_0748 | Hypothetical protein |
| NSE_0754 | Hypothetical protein |
| NSE_0764 | Hypothetical protein |
| NSE_0766 | Type IV secretion system protein VirB4 |
| NSE_0767 | Hypothetical protein |
| NSE_0768 | Type IV secretion system protein VirB2, putative |
| NSE_0769 | Hypothetical protein |
| NSE_0772 | Hypothetical protein |
| NSE_0774 | Hypothetical protein |
| NSE_0775 | Hypothetical protein |
| NSE_0776 | Hypothetical protein |
| NSE_0778 | Hypothetical protein |
| NSE_0780 | Hypothetical protein |
| NSE_0786 | Hypothetical protein |
| NSE_0787 | Hypothetical protein |
| NSE_0789 | Hypothetical protein |
| NSE_0795 | Hypothetical protein |
| NSE_0797 | Hypothetical protein |
| NSE_0801 | Hypothetical protein |
| NSE_0805 | Hypothetical protein |
| NSE_0810 | Hypothetical protein |
| NSE_0813 | Hypothetical protein |
| NSE_0819 | Hypothetical protein |
| NSE_0823 | Hypothetical protein |
| NSE_0830 | Hypothetical protein |
| NSE_0831 | Hypothetical protein |
| NSE_0834 | Hypothetical protein |
| NSE_0835 | Hypothetical protein |
| NSE_0836 | Deoxyribodipyrimidine photolyase family protein, authentic point mutation |
| NSE_0837 | Hypothetical protein |
| NSE_0838 | Hypothetical protein |
| NSE_0845 | Branched-chain amino acid transporter |
| NSE_0851 | Peptidyl-prolyl cis-trans isomerase, cyclophilin-type |
| NSE_0858 | Conserved hypothetical protein |
| NSE_0867 | Hypothetical protein |
| NSE_0868 | Hypothetical protein |
| NSE_0869 | Hypothetical protein |
| NSE_0870 | Hypothetical protein |
| NSE_0872 | Hypothetical protein |
| NSE_0873 | Hypothetical protein |
| NSE_0874 | Hypothetical protein |
| NSE_0879 | Hypothetical protein |
| NSE_0883 | Hypothetical protein |
| NSE_0886 | Hypothetical protein |
| NSE_0902 | Hypothetical protein |
| NSE_0903 | Hypothetical protein |
| NSE_0905 | Strain-specific surface antigen, degenerate |
| NSE_0906 | Hypothetical protein |
| NSE_0907 | Hypothetical protein |
| NSE_0908 | Strain-specific surface antigen |
| NSE_0909 | Hypothetical protein |
| NSE_0912 | Hypothetical protein |
| NSE_0917 | Hypothetical protein |
| NSE_0920 | Hypothetical protein |
| NSE_0925 | Membrane protein, putative |
| NSE_0926 | Membrane protein, putative |
| NSE_0928 | Hypothetical protein |
| NSE_0930 | Hypothetical protein |
| NSE_0931 | Hypothetical protein |
| NSE_0933 | Hypothetical protein |
| NSE_0943 | Hypothetical protein |
| NSE_0949 | Hypothetical protein |
| NSE_0951 | Hypothetical protein |
| NSE_0952 | Hypothetical protein |
| NSE_0963 | Hypothetical protein |
