# Supplementary material for: Comparative Genomics of Emerging Human Ehrlichiosis Agents
Source: PLoS Genet. 2006 Feb 17;2(2):e21. doi: 10.1371/journal.pgen.0020021 (PMC1366493; doi:10.1371/journal.pgen.0020021)
Supplement: Supplementary file 17 [file pgen.0020021.st012.doc]

**Supplementary Table 12.** Ortholog clusters (see methods) present only in *Anaplasma phagocytophilum* and *Anaplasma marginale*.

| Common name | AMA^[1]^ | APH |
| --- | --- | --- |
| Major outer membrane protein OMP-1A, putative | AM1255 | APH_1359 |
| P44/Msp2 superfamily proteins | AM1144 | APH_1195 |
|  | AM991 | APH_1215 |
|  |  | APH_1221 |
|  |  | APH_1229 |
|  |  | APH_1249 |
|  |  | APH_1256 |
|  |  | APH_1269 |
|  |  | APH_1275 |
|  |  | APH_1287 |
|  |  | APH_1312 |
|  |  | APH_1351 |
|  |  | APH_1352 |
|  |  | APH_1392 |
|  |  | APH_1155 |
|  |  | APH_1169 |
| Hypothetical protein | AM347 | APH_0914 |
|  |  | APH_1167 |
| Hypothetical protein | AM952 | APH_0233 |
| Outer membrane protein-1B | AM1257 | APH_1358 |
| Hypothetical protein | AM1050 | APH_1127 |
| Membrane protein, putative | AM950 | APH_0234 |
| Omp-1N | AM1140 | APH_1220 |
| Hypothetical protein | AM629 | APH_0756 |
| Hypothetical protein | AM613 | APH_0485 |
| Hypothetical protein | AM136 | APH_0117 |
| Hypothetical protein | AM689 | APH_0793 |
| Hypothetical protein | AM1078 | APH_1157 |
| Hypothetical protein | AM1073 | APH_1151 |
| Hypothetical protein | AM788 | APH_0400 |
| Hypothetical protein | AM616 | APH_0708 |
| HGE-2 protein | AM1226 | APH_1387 |
| Hypothetical protein | AM649 | APH_0680 |
| Hypothetical protein | AM356 | APH_0906 |
| Membrane protein, putative | AM589 | APH_0728 |
| Hypothetical protein | AM675 | APH_0726 |

1. AMA, *Anaplasma marginale*; APH, *Anaplasma phagocytophilum*.
