# Supplementary material for: Comparative Genomics of Emerging Human Ehrlichiosis Agents
Source: PLoS Genet. 2006 Feb 17;2(2):e21. doi: 10.1371/journal.pgen.0020021 (PMC1366493; doi:10.1371/journal.pgen.0020021)
Supplement: Supplementary file 18 [file pgen.0020021.st013.doc]

**Supplementary Table 13.** Ortholog clusters (see methods) present only in *Ehrlichia chaffeensis*, *Ehrlichia ruminantium* Welgevonden and *Ehrlichia ruminantium* Gardel.

| Common name | ECH^[1]^ | ERU | ERUG |
| --- | --- | --- | --- |
| Pyrroline-5-carboxylate reductase, putative | ECH_0013 | Erum0030 | ERGA_CDS_09400 |
| Diaminopimelate decarboxylase | ECH_0485 | Erum5340 | ERGA_CDS_05490 |
| Argininosuccinate synthase | ECH_0680 | Erum3770 | ERGA_CDS_03850 |
| Arginine biosynthesis bifunctional protein ArgJ | ECH_0676 | Erum3800 | ERGA_CDS_03880 |
| Conserved domain protein | ECH_0745 | Erum3410 | ERGA_CDS_03440 |
| Lipoprotein, putative | ECH_0929 | Erum1860 | ERGA_CDS_01810 |
| Major outer membrane protein OMP-1Q | ECH_1123 | Erum8620 | ERGA_CDS_09030 |
| Hypothetical protein | ECH_1104 | Erum8450 | ERGA_CDS_08870 |
| Hypothetical protein | ECH_0121 | Erum8190  Erum8340 | ERGA_CDS_08580  ERGA_CDS_08740 |
| Hypothetical protein | ECH_0707  ECH_0710 | Erum3610 | ERGA_CDS_03660 |
| Major facilitator family transporter | ECH_0816 | Erum2820 | ERGA_CDS_02820 |
| Hypothetical protein | ECH_0049 | Erum0330 | ERGA_CDS_00200 |
| Membrane protein, putative | ECH_0670 | Erum3830 | ERGA_CDS_03910 |
| Major outer membrane protein P28-1 | ECH_1144 | Erum8750 | ERGA_CDS_09170 |
| Major outer membrane protein OMP-1Z | ECH_1134 | Erum8710 | ERGA_CDS_09120 |
| Major outer membrane protein OMP-1U | ECH_1126 | Erum8650 | ERGA_CDS_09060 |
| Major outer membrane protein Omp-1N | ECH_1121 | Erum8600 | ERGA_CDS_09010 |
| Major outer membrane protein OMP-1P | ECH_1124 | Erum8630 | ERGA_CDS_09040 |
| Major outer membrane protein OMP-1S | ECH_1132 | Erum8720 | ERGA_CDS_09130 |
| Membrane protein, putative | ECH_0673 | Erum3820 | ERGA_CDS_03900 |
| Major outer membrane protein OMP-1T | ECH_1125 | Erum8640 | ERGA_CDS_09050 |
| Membrane protein, putative | ECH_0518 | Erum5050 | ERGA_CDS_05190 |
| Hypothetical protein | ECH_0681 | Erum3760 | ERGA_CDS_03840 |
| Hypothetical protein | ECH_0181 | Erum1250 | ERGA_CDS_01170 |
| Hypothetical protein | ECH_0255 | Erum7310 | ERGA_CDS_07620 |
| Hypothetical protein | ECH_0087 | Erum0590 | ERGA_CDS_00490 |
| Conserved hypothetical protein | ECH_1148 | Erum8770 | ERGA_CDS_09190 |
| Lipoprotein, putative | ECH_0721 | Erum3580 | ERGA_CDS_03610 |
| Hypothetical protein | ECH_0943 | Erum1790 | ERGA_CDS_01730 |
| Conserved hypothetical protein | ECH_0275 | Erum7180 | ERGA_CDS_07470 |
| Hypothetical protein | ECH_0079 | Erum0500 | ERGA_CDS_00390 |
| Hypothetical protein | ECH_0578 | Erum4530 | ERGA_CDS_04650 |
| Hypothetical protein | ECH_0965 | Erum1620 | ERGA_CDS_01540 |
| Hypothetical protein | ECH_1152 | Erum8790 | ERGA_CDS_09210 |
| Hypothetical protein | ECH_0379 | Erum6300 | ERGA_CDS_06520 |
| Conserved domain protein | ECH_0864 | Erum2280 | ERGA_CDS_02260 |
| Hypothetical protein | ECH_0289 | Erum7060 | ERGA_CDS_07330 |
| Hypothetical protein | ECH_0699 | Erum4390 | ERGA_CDS_04500 |
| Hypothetical protein | ECH_0865 | Erum2300 | ERGA_CDS_02280 |
| Hypothetical protein | ECH_0150 | Erum0990 | ERGA_CDS_00920 |
| Hypothetical protein | ECH_0879 | Erum2170 | ERGA_CDS_02150 |
| Conserved hypothetical protein | ECH_1059 | Erum8110 | ERGA_CDS_08500 |
| Hypothetical protein | ECH_0159 | Erum1050 | ERGA_CDS_00980 |
| Hypothetical protein | ECH_0609 | Erum4400 | ERGA_CDS_04510 |
| Hypothetical protein | ECH_0866 | Erum2240 | ERGA_CDS_02220 |
| Hypothetical protein | ECH_1037 | Erum7960 | ERGA_CDS_08310 |
| Conserved hypothetical protein | ECH_0767 | Erum3260 | ERGA_CDS_03280 |
| Hypothetical protein | ECH_0270 | Erum7200 | ERGA_CDS_07500 |
| Conserved domain protein | ECH_0945 | Erum1770 | ERGA_CDS_01710 |
| Hypothetical protein | ECH_0108 | Erum0720 | ERGA_CDS_00630 |
| Hypothetical protein | ECH_0113 | Erum0680 | ERGA_CDS_00600 |
| Conserved domain protein | ECH_0292 | Erum7020 | ERGA_CDS_07290 |

1. ECH, *Ehrlichia chaffeensis*; ERU, *Ehrlichia ruminantium* Welgevonden; ERUG, *Ehrlichia ruminantium* Gardel.
