# Supplementary material for: Comparative Genomics of Emerging Human Ehrlichiosis Agents
Source: PLoS Genet. 2006 Feb 17;2(2):e21. doi: 10.1371/journal.pgen.0020021 (PMC1366493; doi:10.1371/journal.pgen.0020021)
Supplement: Supplementary file 19 [file pgen.0020021.st014.doc]

**Supplementary Table 14.** Ortholog clusters (see methods) that are absent in all of the tick-, flea-, and louse-borne Rickettsiales, but are present in *Wolbachia* spp. and *Neorickettsia sennetsu*.

| Common name | NSE^[1]^ | WOL | WBM |
| --- | --- | --- | --- |
| Bacterioferritin | NSE_0346 | WD1247 | Wbm0144 |
| Conserved hypothetical protein | NSE_0709 | WD1007 | Wbm0613 |
| Conserved hypothetical protein/strain-specific antigen | NSE_0904 | WD0975 | Wbm0514 |

1. WOL, *Wolbachia pipientis* *w*Mel; WBM, *Wolbachia* endosymbiont *w*Bm; NSE, *Neorickettsia sennetsu*.
