# Supplementary material for: Comparative Genomics of Emerging Human Ehrlichiosis Agents
Source: PLoS Genet. 2006 Feb 17;2(2):e21. doi: 10.1371/journal.pgen.0020021 (PMC1366493; doi:10.1371/journal.pgen.0020021)
Supplement: Supplementary file 20 [file pgen.0020021.st015.doc]

**Supplementary Table 15.** Ortholog clusters (see methods) present in all the pathogenic Rickettsiales but none of the endosymbionts.

| Common name | AMA^[1]^ | APH | ECH | ERUG | ERU | NES | RCO | RPR | RTY |
| --- | --- | --- | --- | --- | --- | --- | --- | --- | --- |
| Metallo-beta-lactamase family protein | AM220 | APH_1062 | ECH_0985 | ERGA_CDS_01390 | Erum1470 | NSE_0580 | RC1342 | RP869 | RT0860 |
| Flavin reductase family protein | AM875 | APH_0311 | ECH_0442 | ERGA_CDS_05980 | Erum5780 | NSE_0807 | RC0191 | RP149 | RT0138 |
| TRAP transporter solute receptor, TAXI family | AM560  AM573 | APH_0625 | ECH_0645 | ERGA_CDS_04130 | Erum4050 | NSE_0237 | RC0259 | RP197 | RT0187 |
| Conserved hypothetical protein | AM038 | APH_0071 | ECH_0207 | ERGA_CDS_07940 | Erum7620 | NSE_0422 | RC0432 | RP318 | RT0308 |

1. AMA, *Anaplasma marginale*; APH_, *Anaplasma phagocytophilum*; ECH_, *Ehrlichia chaffeensis*; ERU, *Ehrlichia ruminantium* Welgevonden; ERUG, *Ehrlichia ruminantium* Gardel; NES, *Neorickettsia sennetsu*; RPR, *Rickettsia prowazekii*; RCO *Rickettsia conorii*; RPR *Rickettsia prowazekii*.
