# Supplementary material for: Comparative Genomics of Emerging Human Ehrlichiosis Agents
Source: PLoS Genet. 2006 Feb 17;2(2):e21. doi: 10.1371/journal.pgen.0020021 (PMC1366493; doi:10.1371/journal.pgen.0020021)
Supplement: Supplementary file 21 [file pgen.0020021.st016.doc]

**Supplementary Table 16.** *A. phagocytophilum* *p44* genes.

| Locus | 5'-End | 3'-End | Length | Start/Stop | Expression | Common name |
| --- | --- | --- | --- | --- | --- | --- |
| APH_0029 | 26886 | 26311 | 576 |  | Yes | P44-63 outer membrane protein, silent |
| APH_0030 | 27944 | 27328 | 617 |  | Yes | P44-64 outer membrane protein, silent |
| APH_0076 | 82973 | 83492 | 520 |  | Yes | P44-65 outer membrane protein, silent |
| APH_0115 | 121880 | 122397 | 518 |  |  | P44-66 outer membrane protein, silent |
| APH_0132 | 137376 | 136823 | 554 |  | Yes | P44-21 outer membrane protein, silent |
| APH_0134 | 138120 | 137815 | 306 |  |  | P44-80 outer membrane protein, truncation |
| APH_0142 | 147346 | 146561 | 786 |  |  | P44-87 outer membrane protein, truncation |
| APH_0143 | 147821 | 148051 | 231 |  |  | P44-81 outer membrane protein, truncation |
| APH_0144 | 148039 | 147836 | 204 |  |  | P44 outer membrane protein, C-terminal fragment |
| APH_0145 | 149309 | 148302 | 1008 | Yes | Yes | P44-8 outer membrane protein |
| APH_0146 | 149391 | 149266 | 126 |  |  | P44 outer membrane protein, N-terminal fragment |
| APH_0171 | 174024 | 172921 | 1104 | Yes | Yes | P44-45 outer membrane protein |
| APH_0172 | 174288 | 174085 | 204 |  |  | P44 outer membrane protein, C-terminal fragment |
| APH_0174 | 175531 | 174551 | 981 | Yes | Yes | P44-55 outer membrane protein, silent |
| APH_0214 | 214003 | 213533 | 471 |  |  | P44-88 outer membrane protein, silent |
| APH_0226 | 224371 | 224201 | 171 |  |  | P44 outer membrane protein, C-terminal fragment |
| APH_0228 | 226160 | 225599 | 562 |  |  | P44-67 outer membrane protein, silent |
| APH_0229 | 227059 | 226543 | 517 |  |  | P44-68 outer membrane protein, silent |
| APH_0318 | 303552 | 303875 | 324 |  |  | P44 outer membrane protein, N-terminal fragment |
| APH_0319 | 303903 | 304425 | 523 |  |  | P44-69 outer membrane protein, silent |
| APH_0535 | 552485 | 552676 | 192 |  |  | P44-82 outer membrane protein, truncation |
| APH_0632 | 658114 | 656999 | 1116 |  |  | P44-84 outer membrane protein, degenerate |
| APH_0662 | 680648 | 681976 | 1329 | Yes |  | P44-70 outer membrane protein |
| APH_0663 | 682036 | 683325 | 1290 | Yes |  | P44-71 outer membrane protein |
| APH_0664 | 683389 | 684696 | 1308 | Yes |  | P44-72 outer membrane protein |
| APH_0900 | 957413 | 957988 | 576 |  | Yes | P44-57 outer membrane protein, silent |
| APH_0902 | 958566 | 959119 | 554 |  |  | P44-73 outer membrane protein, silent |
| APH_0934 | 999990 | 1000506 | 517 |  | Yes | P44-34 outer membrane protein, silent |
| APH_0935 | 1000509 | 1000841 | 333 |  |  | P44-85 outer membrane protein, truncation |
| APH_0936 | 1000834 | 1001250 | 417 |  |  | P44-86 outer membrane protein, truncation |
| APH_0938 | 1001717 | 1002064 | 348 |  |  | P44 outer membrane protein, C-terminal fragment |
| APH_0951 | 1014245 | 1014418 | 174 |  |  | P44 outer membrane protein, C-terminal fragment |
| APH_0953 | 1014850 | 1015083 | 234 |  |  | P44 outer membrane protein, C-terminal fragment |
| APH_0980 | 1039370 | 1038552 | 819 |  |  | P44 outer membrane protein, C-terminal fragment |
| APH_0981 | 1040022 | 1039484 | 539 |  | Yes | P44-44 outer membrane protein, silent |
| APH_1018 | 1078660 | 1078014 | 647 |  | Yes | P44-62 outer membrane protein, silent |
| APH_1063 | 1126419 | 1126938 | 520 |  | Yes | P44-38 outer membrane protein, silent |
| APH_1064 | 1127667 | 1128217 | 551 |  | Yes | P44-42 outer membrane protein, silent |
| APH_1065 | 1128359 | 1128899 | 541 |  |  | P44-74 outer membrane protein, silent |
| APH_1076 | 1141236 | 1141006 | 231 |  |  | P44 outer membrane protein, C-terminal fragment |
| APH_1077 | 1142182 | 1141512 | 671 |  | Yes | P44-41 outer membrane protein, silent |
| APH_1078 | 1142848 | 1142364 | 485 |  | Yes | P44-56 outer membrane protein, silent |
| APH_1080 | 1146352 | 1146910 | 559 |  | Yes | P44-50 outer membrane protein, silent |
| APH_1081 | 1147139 | 1147928 | 790 |  | Yes | P44-28 outer membrane protein, silent |
| APH_1082 | 1147987 | 1148517 | 531 |  | Yes | P44-52 outer membrane protein, silent |
| APH_1114 | 1173721 | 1174788 | 1068 | Yes | Yes | P44-7 outer membrane protein |
| APH_1115 | 1174886 | 1175182 | 297 |  |  | P44 outer membrane protein, C-terminal fragment |
| APH_1118 | 1180993 | 1180043 | 951 |  | Yes | P44-7b outer membrane protein, silent |
| APH_1121 | 1183639 | 1184171 | 533 |  | Yes | P44-6 outer membrane protein, silent |
| APH_1122 | 1184304 | 1184862 | 559 |  |  | P44-75 outer membrane protein, silent |
| APH_1123 | 1185054 | 1185614 | 561 |  | Yes | P44-3 outer membrane protein, silent |
| APH_1124 | 1185766 | 1185936 | 171 |  |  | P44 outer membrane protein, C-terminal fragment |
| APH_1125 | 1186140 | 1186427 | 288 |  |  | P44 outer membrane protein, C-terminal fragment |
| APH_1128 | 1191435 | 1190963 | 473 |  |  | P44-76 outer membrane protein, silent |
| APH_1152 | 1212931 | 1212141 | 791 |  | Yes | P44-47 outer membrane protein, silent |
| APH_1154 | 1219499 | 1219007 | 493 |  | Yes | P44-4 outer membrane protein, silent |
| APH_1155 | 1220803 | 1219544 | 1260 | Yes | Yes | P44-53 outer membrane protein |
| APH_1163 | 1227712 | 1228654 | 943 |  | Yes | P44-53b outer membrane protein, silent |
| APH_1168 | 1236532 | 1235432 | 1101 | Yes | Yes | P44-36 outer membrane protein |
| APH_1169 | 1237543 | 1236593 | 951 | Yes | Yes | P44-19 outer membrane protein, silent |
| APH_1172 | 1240820 | 1240584 | 237 |  |  | P44 outer membrane protein, C-terminal fragment |
| APH_1176 | 1244325 | 1245404 | 1080 | Yes | Yes | P44-2 outer membrane protein |
| APH_1177 | 1245704 | 1246366 | 663 |  | Yes | P44-54 outer membrane protein, silent |
| APH_1184 | 1256681 | 1256136 | 546 |  | Yes | P44-43 outer membrane protein, silent |
| APH_1185 | 1256883 | 1256713 | 171 |  |  | P44 outer membrane protein, C-terminal fragment |
| APH_1186 | 1256988 | 1257776 | 789 |  | Yes | P44-40 outer membrane protein, silent |
| APH_1194 | 1264166 | 1263405 | 762 |  | Yes | P44-18 outer membrane protein, silent |
| APH_1195 | 1265383 | 1264142 | 1242 | Yes | Yes | P44-1 outer membrane protein |
| APH_1202 | 1270154 | 1269498 | 657 |  |  | P44-77 outer membrane protein, silent |
| APH_1203 | 1270818 | 1270289 | 530 |  | Yes | P44-34b outer membrane protein, silent |
| APH_1215 | 1285256 | 1283955 | 1302 | Yes | Yes | P44-14 outer membrane protein |
| APH_1221 | 1290383 | 1291678 | 1296 | Yes | Yes | P44-18ES, P44 outer membrane protein expression locus with P44-18 |
| APH_1226 | 1296194 | 1295441 | 754 |  | Yes | P44-15 outer membrane protein, silent |
| APH_1229 | 1299025 | 1300302 | 1278 | Yes | Yes | P44-2b outer membrane protein |
| APH_1230 | 1300408 | 1300659 | 252 |  |  | P44 outer membrane protein, C-terminal fragment |
| APH_1238 | 1306915 | 1308022 | 1108 | - | Yes | P44-13 outer membrane protein, silent |
| APH_1239 | 1308083 | 1309177 | 1095 | Yes | Yes | P44-15b outer membrane protein |
| APH_1241 | 1312166 | 1311538 | 629 |  | Yes | P44-33 outer membrane protein, silent |
| APH_1242 | 1313347 | 1312404 | 944 |  | Yes | P44-49 outer membrane protein, silent |
| APH_1248 | 1320843 | 1320158 | 686 |  | Yes | P44-27 outer membrane protein, silent |
| APH_1249 | 1322068 | 1320893 | 1176 | Yes | Yes | P44-12 outer membrane protein |
| APH_1253 | 1324187 | 1324870 | 684 |  | Yes | P44-39 outer membrane protein, silent |
| APH_1255 | 1325391 | 1326197 | 807 |  | Yes | P44-10 outer membrane protein, silent |
| APH_1256 | 1326258 | 1327445 | 1188 | Yes | Yes | P44-23 outer membrane protein |
| APH_1261 | 1330484 | 1331006 | 523 |  | Yes | P44-46 outer membrane protein, silent |
| APH_1269 | 1338106 | 1336817 | 1290 | Yes | Yes | P44-16 outer membrane protein |
| APH_1272 | 1341386 | 1342293 | 908 |  | Yes | P44-48 outer membrane protein, silent, authentic point mutation |
| APH_1273 | 1342590 | 1342694 | 105 |  |  | P44 outer membrane protein, C-terminal fragment |
| APH_1274 | 1342744 | 1343661 | 918 |  | Yes | P44-25 outer membrane protein, silent |
| APH_1275 | 1343722 | 1345008 | 1287 | Yes | Yes | P44-16b outer membrane protein |
| APH_1284 | 1352113 | 1351934 | 180 |  |  | P44 outer membrane protein, C-terminal fragment |
| APH_1287 | 1354240 | 1355508 | 1269 | Yes | Yes | P44-32 outer membrane protein |
| APH_1311 | 1379532 | 1378339 | 1194 | Yes | Yes | P44-24 outer membrane protein |
| APH_1312 | 1380468 | 1379593 | 876 |  | Yes | P44-5 outer membrane protein, silent |
| APH_1313 | 1381367 | 1380593 | 775 |  | Yes | P44-26 outer membrane protein, silent |
| APH_1324 | 1391128 | 1391346 | 219 |  |  | P44 outer membrane protein, N-terminal fragment |
| APH_1328 | 1395781 | 1395185 | 597 |  | Yes | P44-17 outermembrane protein, silent |
| APH_1329 | 1396620 | 1395899 | 722 |  | Yes | P44-60 outer membrane protein, silent |
| APH_1330 | 1397647 | 1397038 | 610 |  | Yes | P44-29 outer membrane protein, silent |
| APH_1342 | 1406760 | 1406235 | 526 |  | Yes | P44-31 outer membrane protein, silent |
| APH_1343 | 1407854 | 1407217 | 638 |  | Yes | P44-58 outer membrane protein, silent |
| APH_1350 | 1414292 | 1415197 | 906 |  | Yes | P44-51 outer membrane protein, silent |
| APH_1351 | 1415643 | 1416459 | 817 |  | Yes | P44-35 outer membrane protein, silent |
| APH_1352 | 1416517 | 1417413 | 897 |  | Yes | P44-30 outer membrane protein, silent |
| APH_1355 | 1420199 | 1418814 | 1386 | Yes |  | P44-79 outer membrane protein |
| APH_1389 | 1451723 | 1451466 | 258 |  |  | P44 outer membrane protein, C-terminal fragment |
| APH_1390 | 1452477 | 1451804 | 674 |  | Yes | P44-20 outer membrane protein, silent |
| APH_1391 | 1453355 | 1452555 | 801 |  | Yes | P44-9 outer membrane protein, silent |
| APH_1392 | 1454572 | 1453433 | 1140 | Yes | Yes | P44-11 outer membrane protein |
| APH_1399 | 1458228 | 1458112 | 117 |  |  | P44 outer membrane protein, C-terminal fragment |
| APH_1400 | 1458734 | 1458201 | 534 |  | Yes | P44-37 outer membrane protein, silent |
| APH_1401 | 1459630 | 1458860 | 771 |  | Yes | P44-22 outer membrane protein, silent |
| APH_1408 | 1467987 | 1467184 | 804 |  | Yes | P44-59 outer membrane protein, silent |
