# Supplementary material for: Comparative Genomics of Emerging Human Ehrlichiosis Agents
Source: PLoS Genet. 2006 Feb 17;2(2):e21. doi: 10.1371/journal.pgen.0020021 (PMC1366493; doi:10.1371/journal.pgen.0020021)
Supplement: Supplementary file 22 [file pgen.0020021.st017.doc]

**Supplementary Table 17.** Putative *A. phagocytophilum* type IV effector motifs in HGE-14 proteins with important arginines highlighted.

| Locus | C-terminus |
| --- | --- |
| APH_0382 | SPELDDAQVLAY**R**P**R**TPSWDIEPPVK**R**A**RS**A |
| APH_0385 | SELQEAQVSSHGS**R**TPSDDELEPPSK**R**S**RS**A |
| APH_0386 | QTSVS**R**SASTSVTTSSELEGAQVTAH**R**S**R**TP**R**GEIADLVH**R**LEEHNLQ |
| APH_0387 | STLEEAQVSTH**R**SQTPDDDEDEQPSK**R**A**R**Y**R** |
| APH_0452 | FTGAPLGTPQITSSQQVFYATDLDLT**R**KI |
| APH_0453 | SVLEEA**R**ISSP**R**SL**R**TPSWDIEPPVK**R**A**RS**A |
| APH_0455 | STLEEAQVSSH**R**P**R**TPSDDDSEPPSKQA**RR**A |
| APH_0457 | SILQDLLVTSPLQSNNESELMQPPQK**R**P**RS**V |
