# Supplementary material for: Comparative Genomics of Emerging Human Ehrlichiosis Agents
Source: PLoS Genet. 2006 Feb 17;2(2):e21. doi: 10.1371/journal.pgen.0020021 (PMC1366493; doi:10.1371/journal.pgen.0020021)
Supplement: Supplementary file 23 [file pgen.0020021.st018.doc]

**Supplementary Table 18. DNA repair genes.**

| **DNA Repair** | | | **ECH^[1]^** | **APH** | **WOL** | **NES** | **RPR** | **RCO** |
| --- | --- | --- | --- | --- | --- | --- | --- | --- |
| **Direct Repair** | | |  |  |  |  |  |  |
|  |  | Photolyase |  |  |  | NSE_0836 |  |  |
|  |  | DNA ligase ligA | ECH_0301 | APH_0138 | WD0776 | NSE_0500 | RP720 | RC1096 |
|  |  | Alkyltransferase |  |  |  |  |  | RC0867 |
| **AP Endonuclease** | | |  |  |  |  |  |  |
|  |  | Xth | ECH_0675 | APH_0505 | WD1001 | NSE_0415 | RP260 | RC0346 |
| **Base Excision Repair Glycosylases** | | | | | |  |  |  |
|  |  | 3mg | ECH_0277 |  | WD1110 |  | RP351 | RC0480 |
|  |  | Ung Family4 | ECH_0074 | APH_1371 |  | NSE_0935 |  |  |
|  |  | Fpg | ECH_0602 | APH_0411 | WD1158 |  |  | RC1038 |
|  |  | Nth | ECH_0857 | APH_0897 | WD0789 | NSE_0246 | RP746 | RC1149 |
| **Nucleotide Excision Repair** | | | |  |  |  |  |  |
|  |  | UvrA | ECH_0785 | APH_0537 | WD0916 |  | RP835 | RC1294 |
|  |  | UvrB |  | APH_1367 | WD0839 |  | RP203 | RC0266 |
|  |  | UvrC |  | APH_0884 | WD0154 |  | RP572 | RC0864 |
|  |  | UvrD | ECH_0858 | APH_0903 | WD0359 | NSE_0356 | RP734 | RC0624 |
|  |  | UvrD family | ECH_0387 | APH_0258 | WD0963 | NSE_0467 |  | RC1114 |
| **Transciption Repair Coupling** | | | |  |  |  |  |  |
|  |  | Mfd | ECH_0250 | APH_0107 |  |  | RP598 | RC0913 |
| **Mismatch Repair** | | |  |  |  |  |  |  |
|  |  | MutL | ECH_0884 | APH_0939 | WD0509 | NSE_0609 | RP880 | RC1361 |
|  |  | MutL2 |  |  | WD1306 |  |  |  |
|  |  | MutS | ECH_0824 | APH_0857 | WD0190 | NSE_0335 | RP298 | RC0401 |
| **Homologous Recombination** | | | |  |  |  |  |  |
|  | RecF Pathway | |  |  |  |  |  |  |
|  |  | RecF | ECH_0076 | APH_1409 | WD1286 | NSE_0199 | RP029 | RC0032 |
|  |  | RecJ | ECH_1115 | APH_1165 | WD0312 | NSE_0712 | RP528 | RC0761 |
|  |  | RecN |  |  |  |  | RP182 | RC0229 |
|  |  | RecO | ECH_0536 | APH_0736 |  | NSE_0461 |  |  |
|  |  | RecR | ECH_0843 | APH_0988 | WD1180 | NSE_0899 | RP438 | RC0609 |
|  |  | RecQ |  |  |  |  |  |  |
|  | Recombinase | |  |  |  |  |  |  |
|  |  | RecA | ECH_1109 | APH_1354 | WD1050 | NSE_0530 | RP761 | RC1182 |
|  | Holliday junction resolution | | |  |  |  |  |  |
|  |  | RuvA | ECH_0320 | APH_0167 | WD1113 | NSE_0574 | RP385 | RC0531 |
|  |  | RuvB | ECH_0319 | APH_0166 | WD1112 | NSE_0575 | RP386 | RC0533 |
|  |  | RuvC | ECH_0028 | APH_0018 | WD0142 | NSE_0955 | RP119 | RC0159 |
|  |  | RecG | ECH_0062 | APH_1298 | WD0824 | NSE_0690 | RP593 | RC0901 |
|  | Other recombination | |  |  |  |  |  |  |
|  |  | RadA | ECH_0305 |  | WD0887 |  | RP546 | RC0810 |
| **Other** | | |  |  |  |  |  |  |
|  |  | RadC | ECH_0363 | APH_0242 | WD0357 | NSE_0229 |  |  |
|  |  | XseL | ECH_0056 | APH_1322 |  |  | RP350 | RC0475 |
|  |  | XseS | ECH_0214 | APH_0079 |  |  | RP675 | RC1026 |
|  |  | Hu |  | APH_0784 | WD1089 | NSE_0640 | RP524 | RC0757 |
|  |  | Hu2 |  |  | WD0057 |  |  |  |
|  |  | Hu3 |  |  | WD0065 |  |  |  |
|  |  | RmuC |  | APH_0428 |  |  | RP690 |  |

1. APH, *Anaplasma phagocytophilum*; ECH, *Ehrlichia chaffeensis*; WOL, *Wolbachia pipientis* *w*Mel; NES, *Neorickettsia sennetsu*; RPR, *Rickettsia prowazekii*; RCO, *Rickettsia conorii*.
