# Supplementary material for: Comparative Genomics of Emerging Human Ehrlichiosis Agents
Source: PLoS Genet. 2006 Feb 17;2(2):e21. doi: 10.1371/journal.pgen.0020021 (PMC1366493; doi:10.1371/journal.pgen.0020021)
Supplement: Supplementary file 24 [file pgen.0020021.st019.doc]

**Supplementary Table 19.** Sequences of oligonucleotides used for RT-PCR.

| Target Gene | | Forward | Reverse |
| --- | --- | --- | --- |
| *E. chaffeensis* | |  |  |
|  | *BioA* | TACGCTTACCGCAGACAGAA | TATTATCCCAGTTGCACCTTTTAC |
|  | *BioB* | TACGATCTCATTTAACACATAG | ATCTTCAATACTTTCACCAA |
|  | *BioD* | GCGGTACTGATATAGGAAAAACT | AGAGCCTATAACCAAAATCACAT |
|  | *BioF* | ATGGCTTTAGTATTTAGTAGTGG | GGTTCAGCAAGATTCAGATTTTTA |
| *A. phagocytophilum* | |  |  |
|  | *BioA* | CAGTGGCCCCGATGAGAGTGG | ACCTGCAGCCTGTAAAAGTGG |
|  | *BioB* | AGAGCGCACATAACAAAACTGG | CTGGCATCATTATACGAGCAACC |
|  | *BioD* | CTACAGAGGGTGAGTCCGATACG | ATGGGTTCTCTTCCTCTTTCTCAG |
|  | *BioF* | TCCCGTAGACGAGTTTATCCA | CACGCTTTCAATGCATTACACAGT |
